# Supplementary figures and images for: Evaluation of surface-based hippocampal registration using ground-truth subfield definitions
Source: eLife. 2023 Nov 13;12:RP88404. doi: 10.7554/eLife.88404 (PMC10642966; doi:10.7554/eLife.88404)

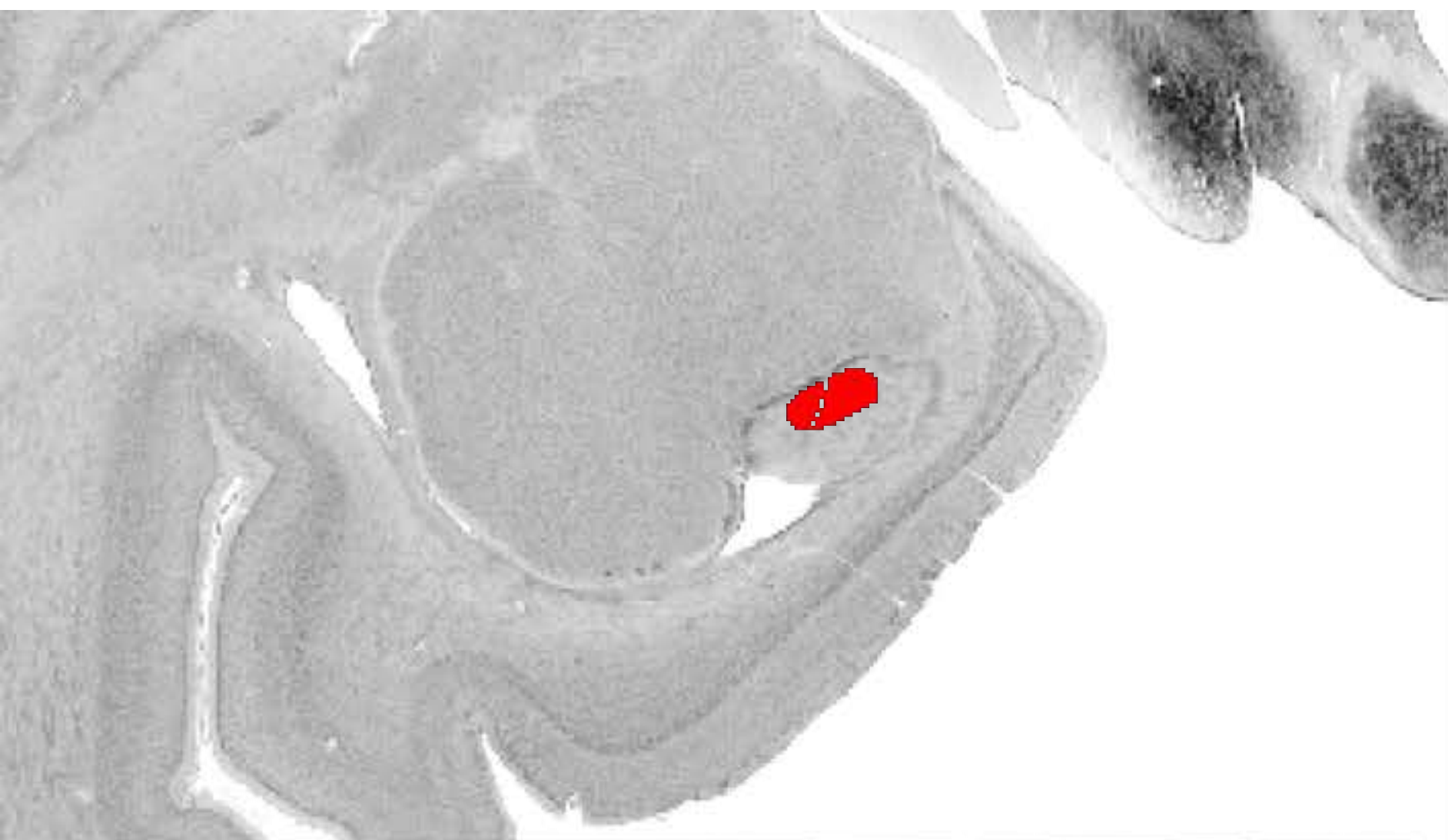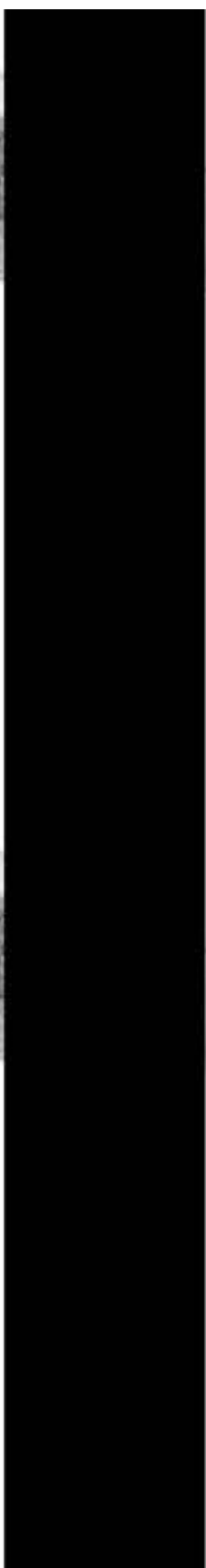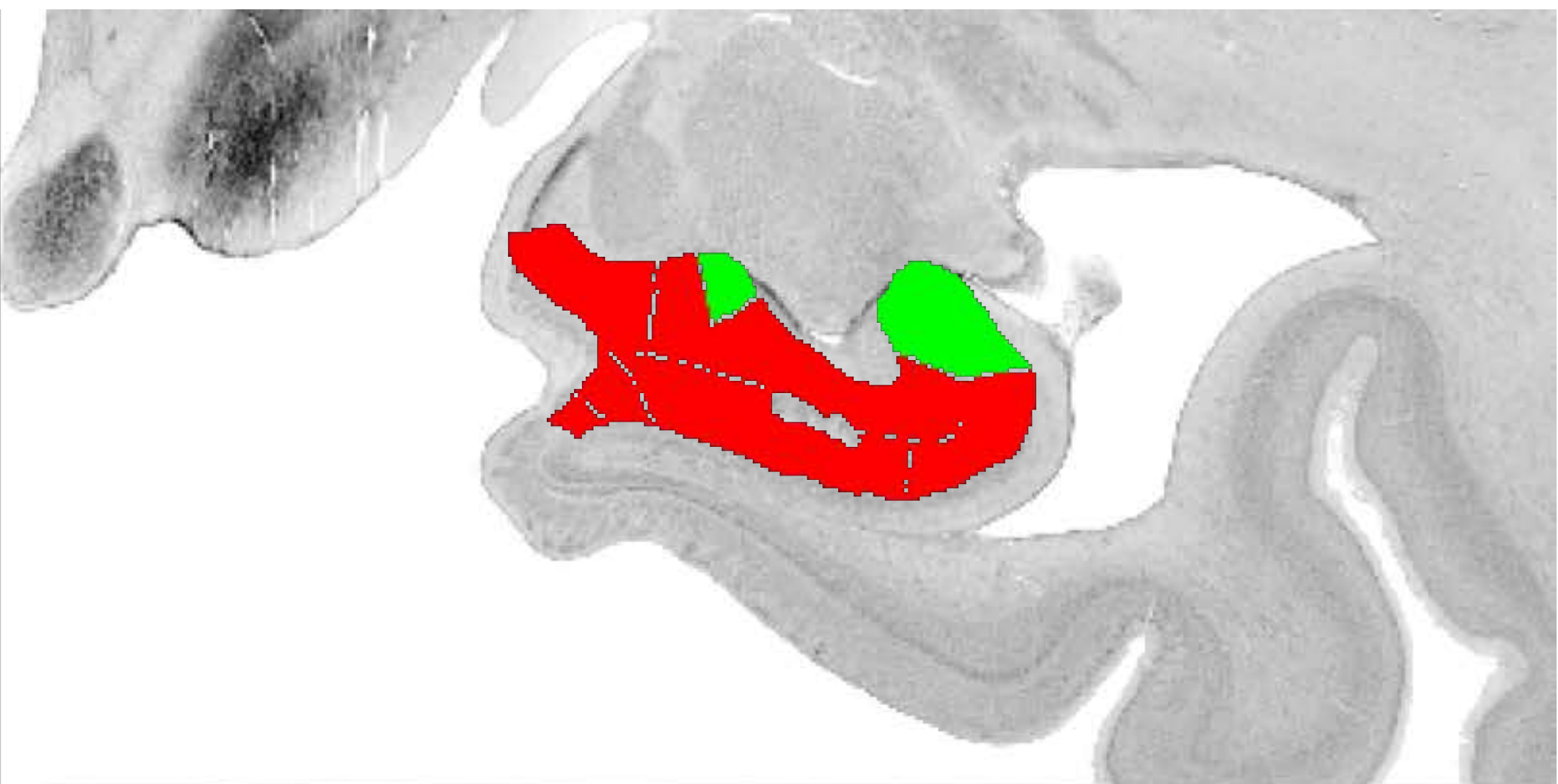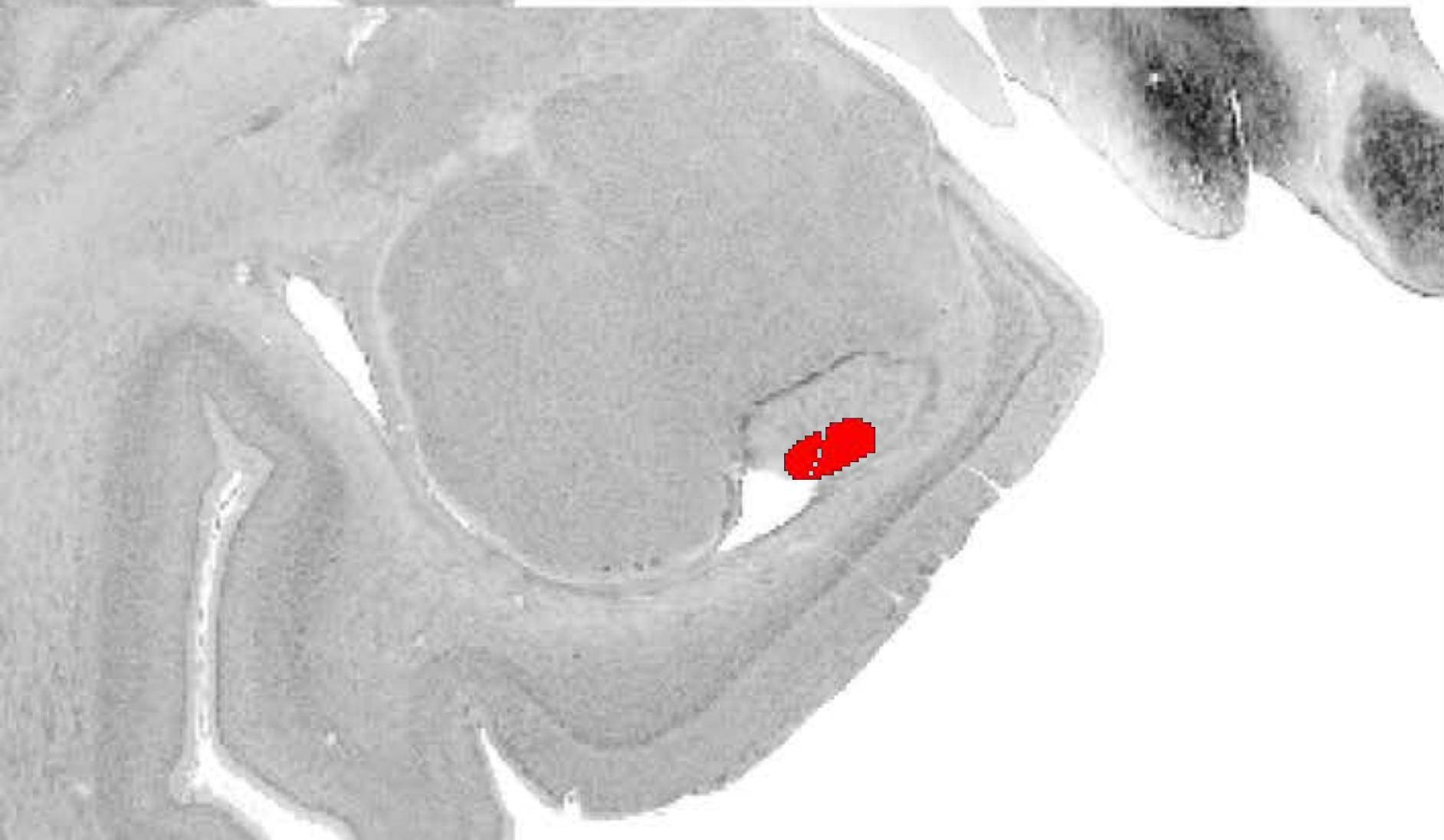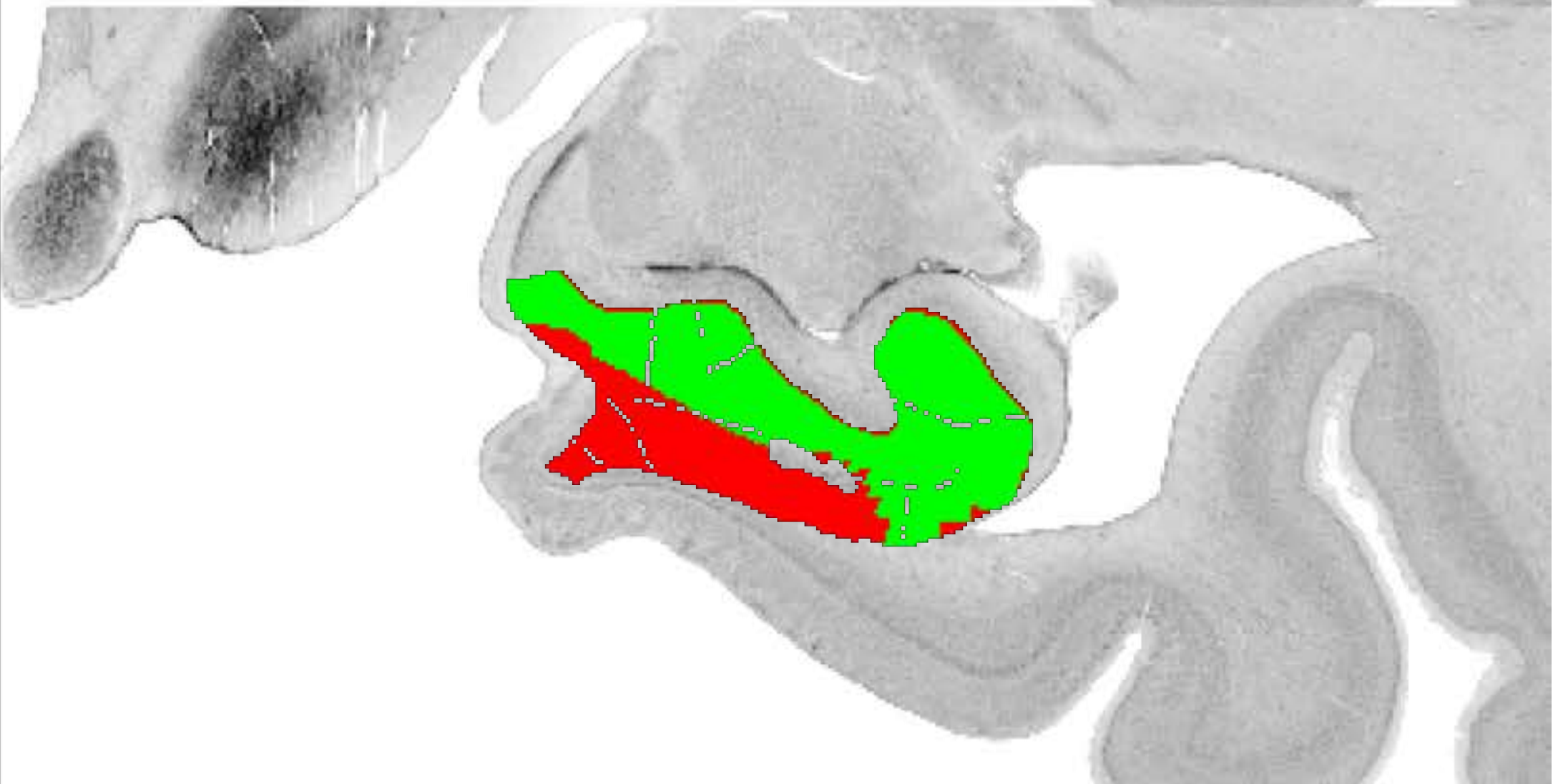

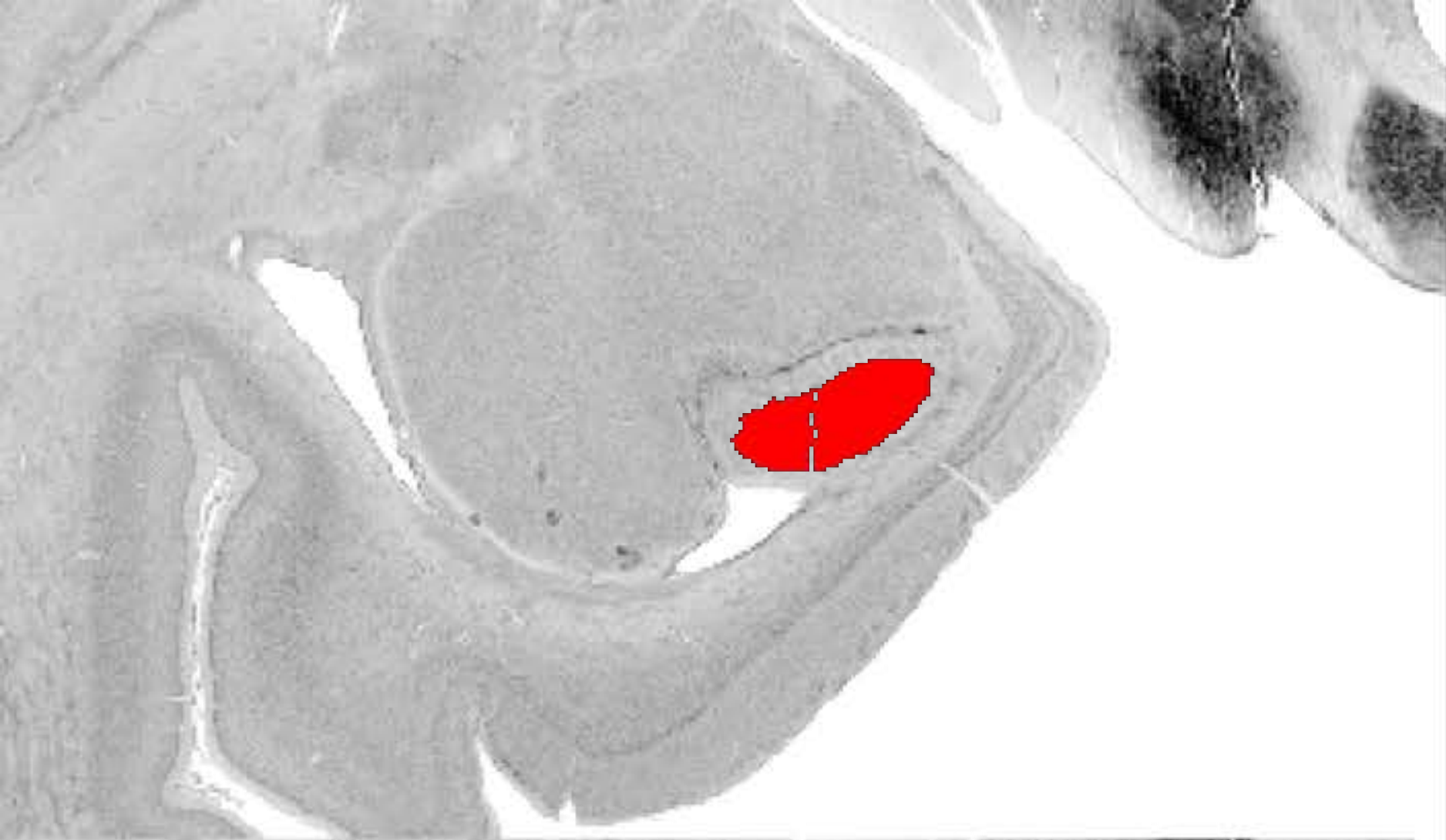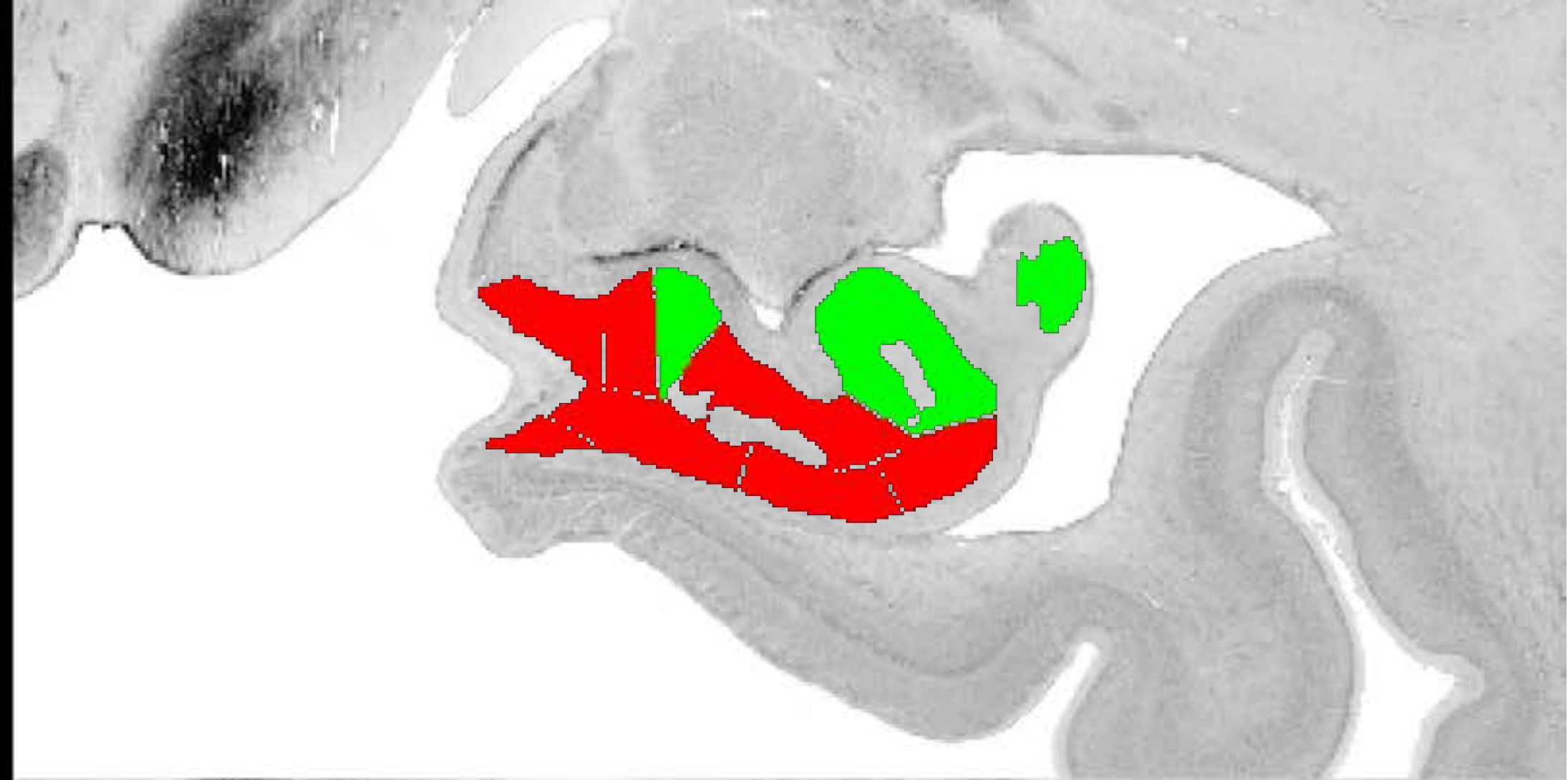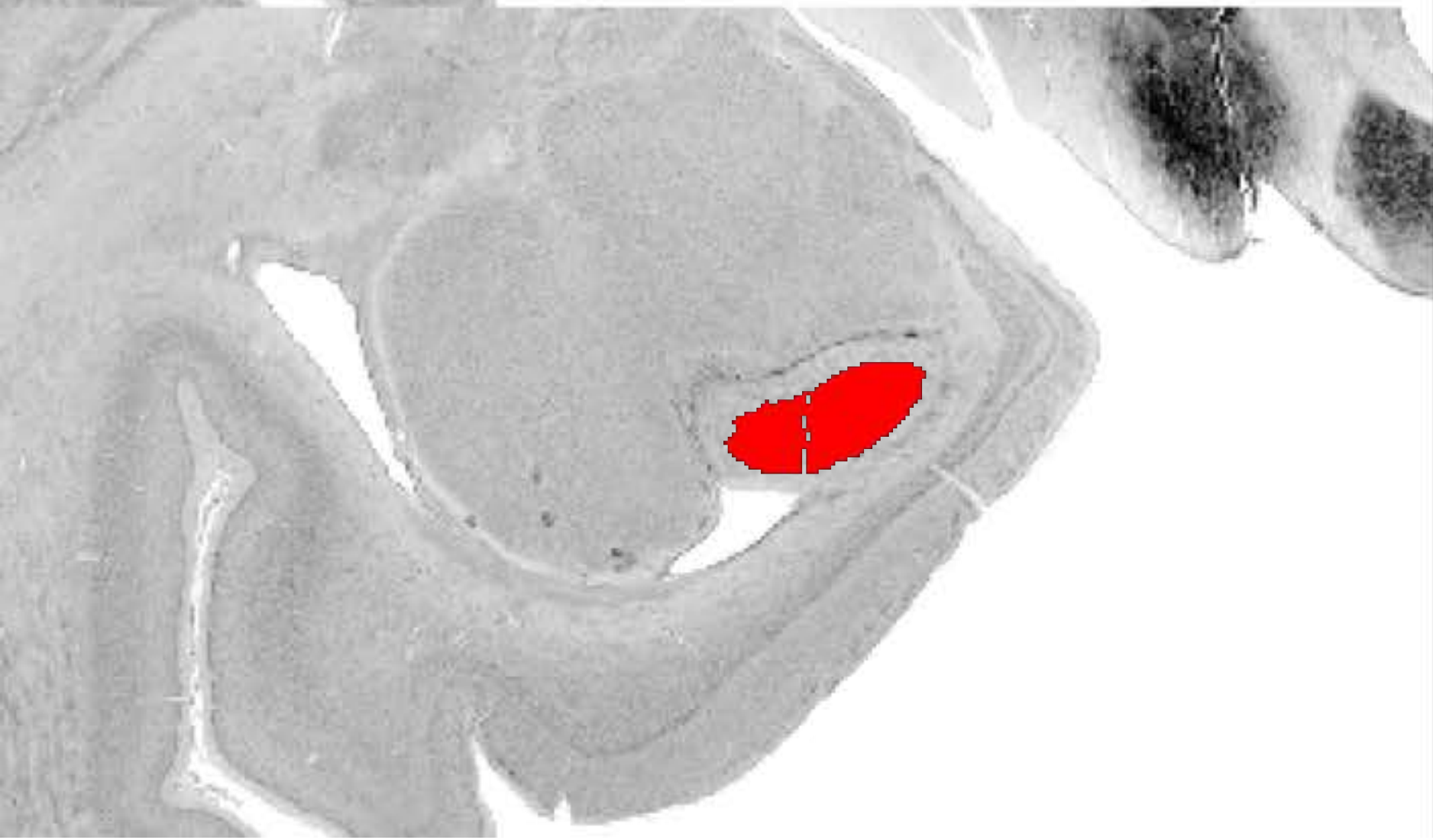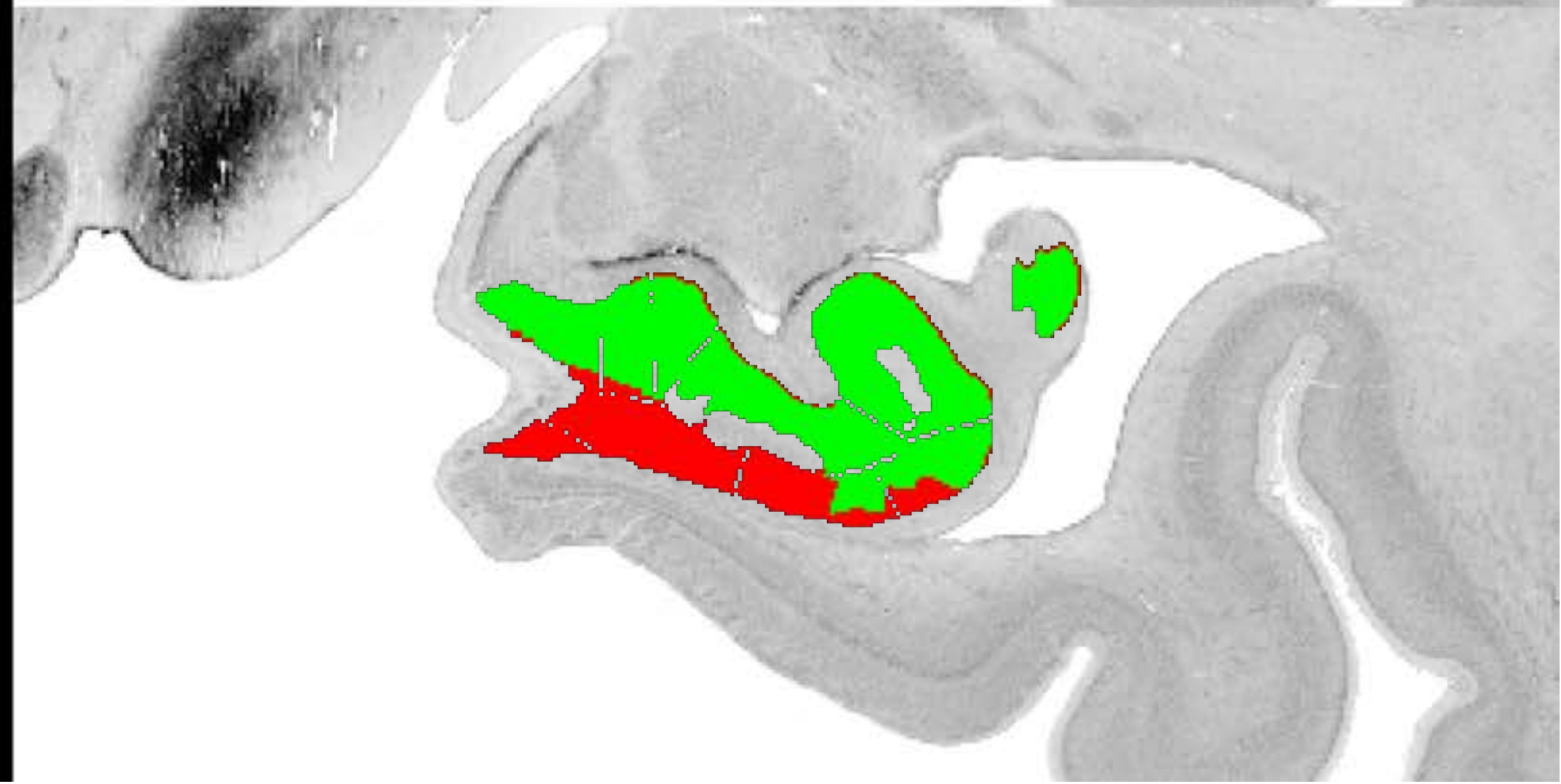

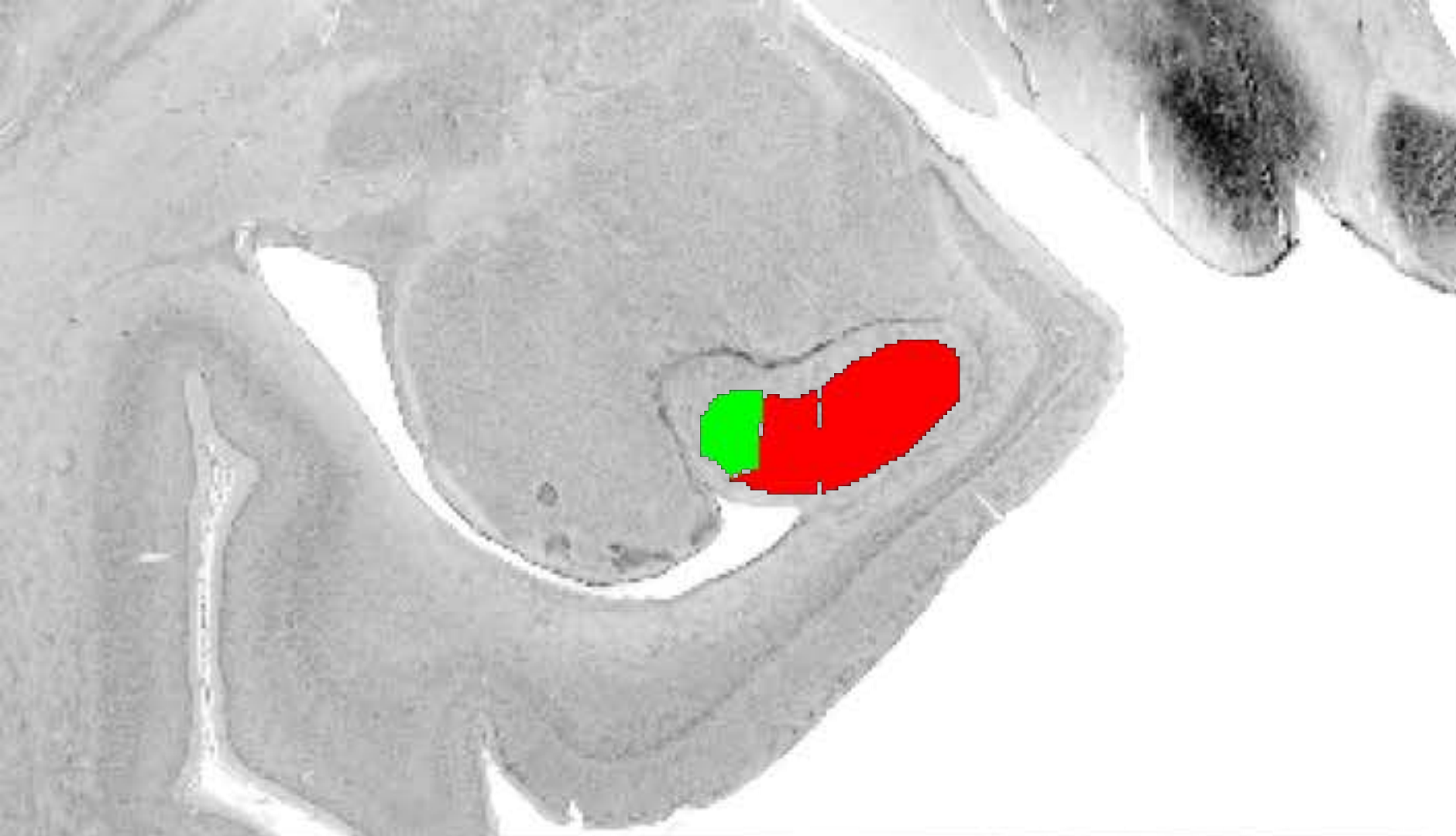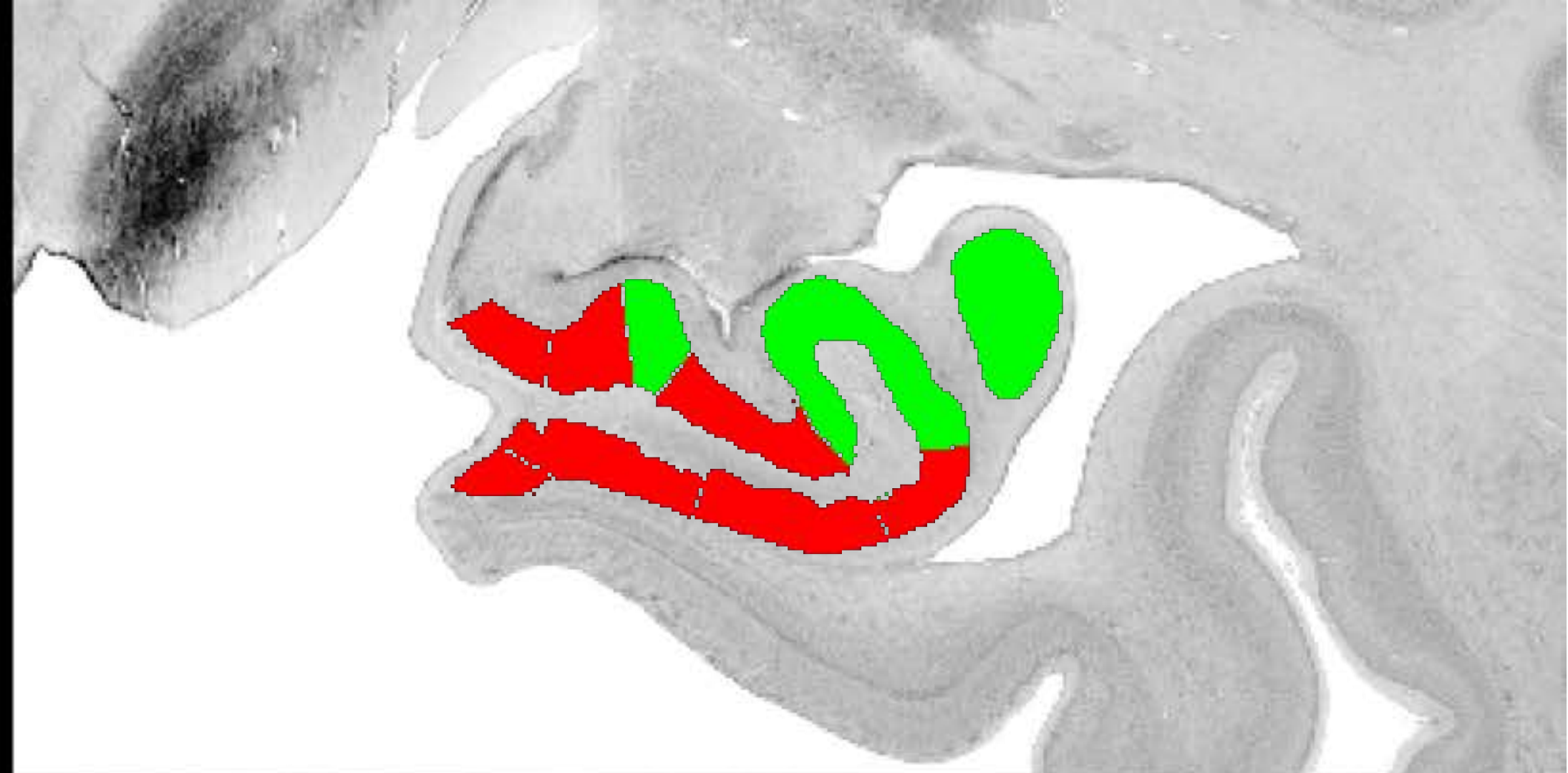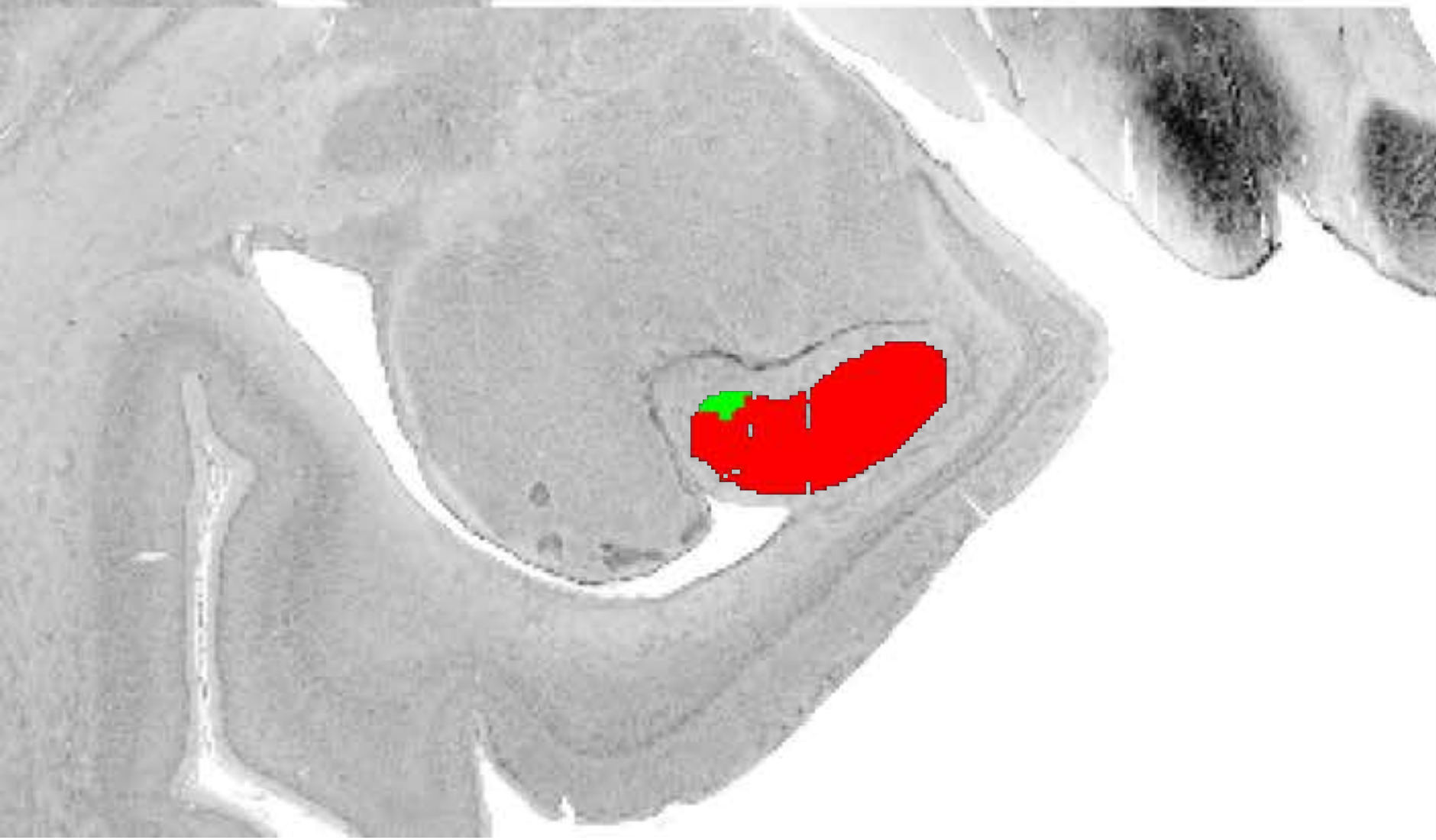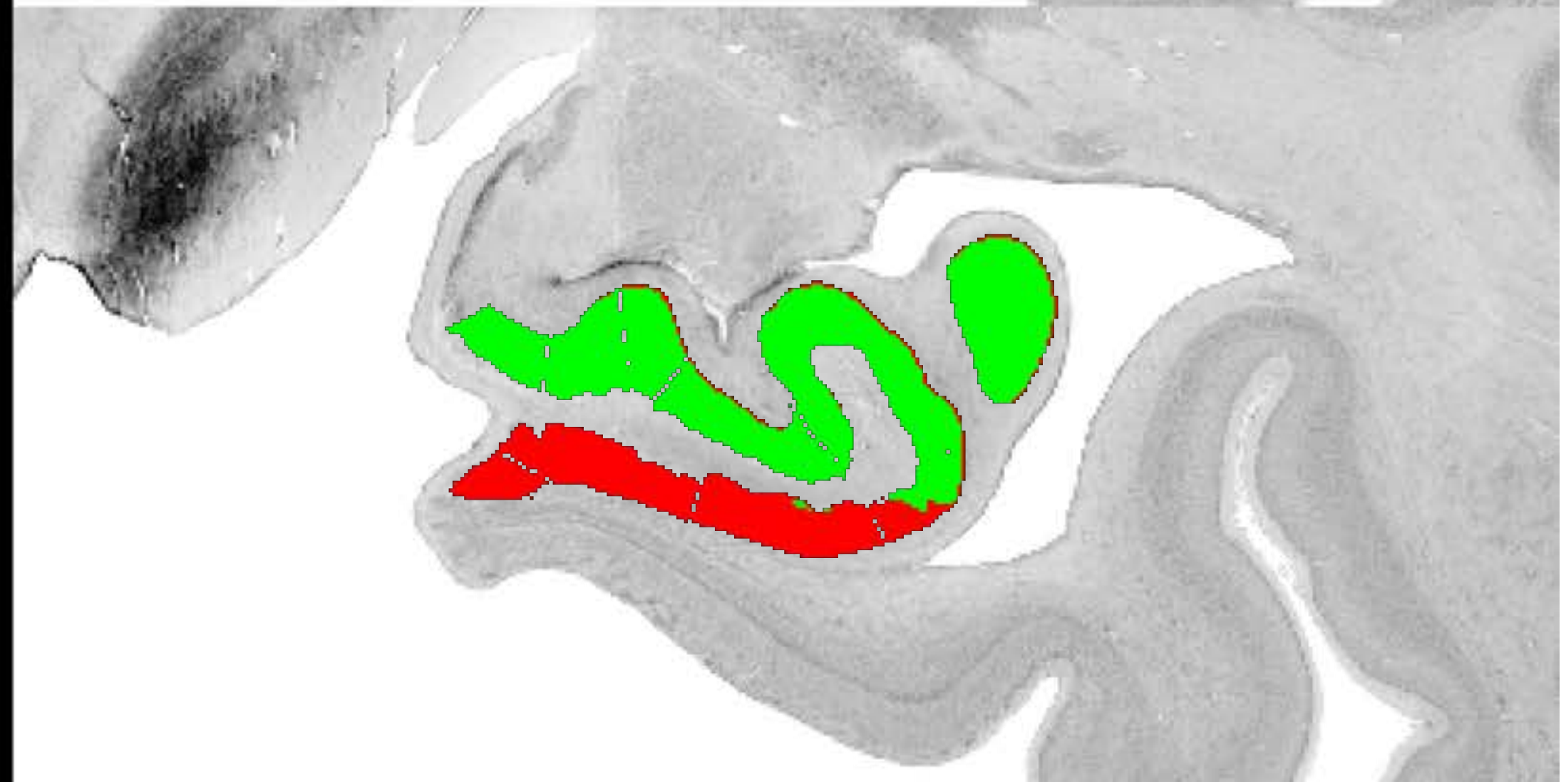

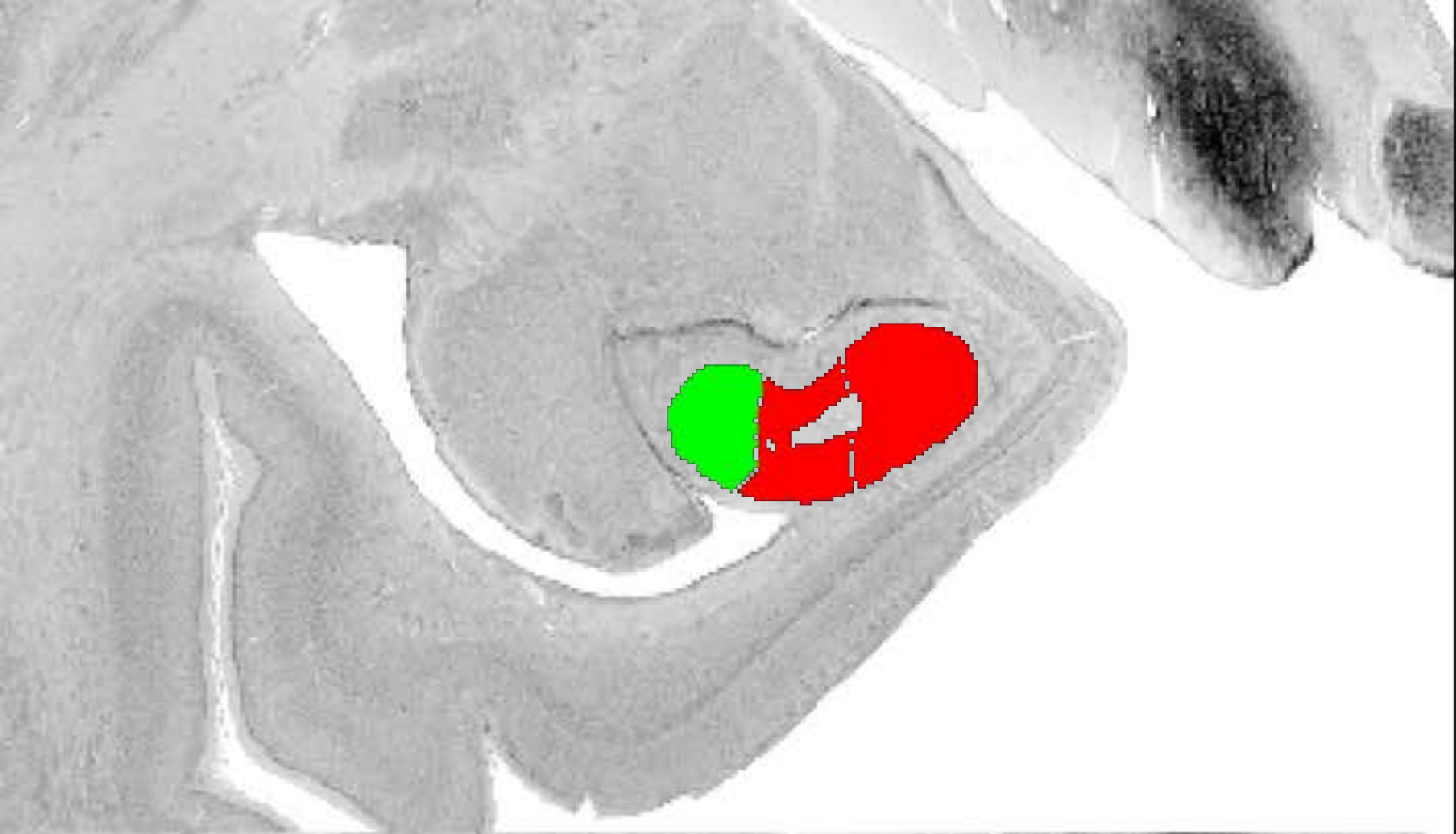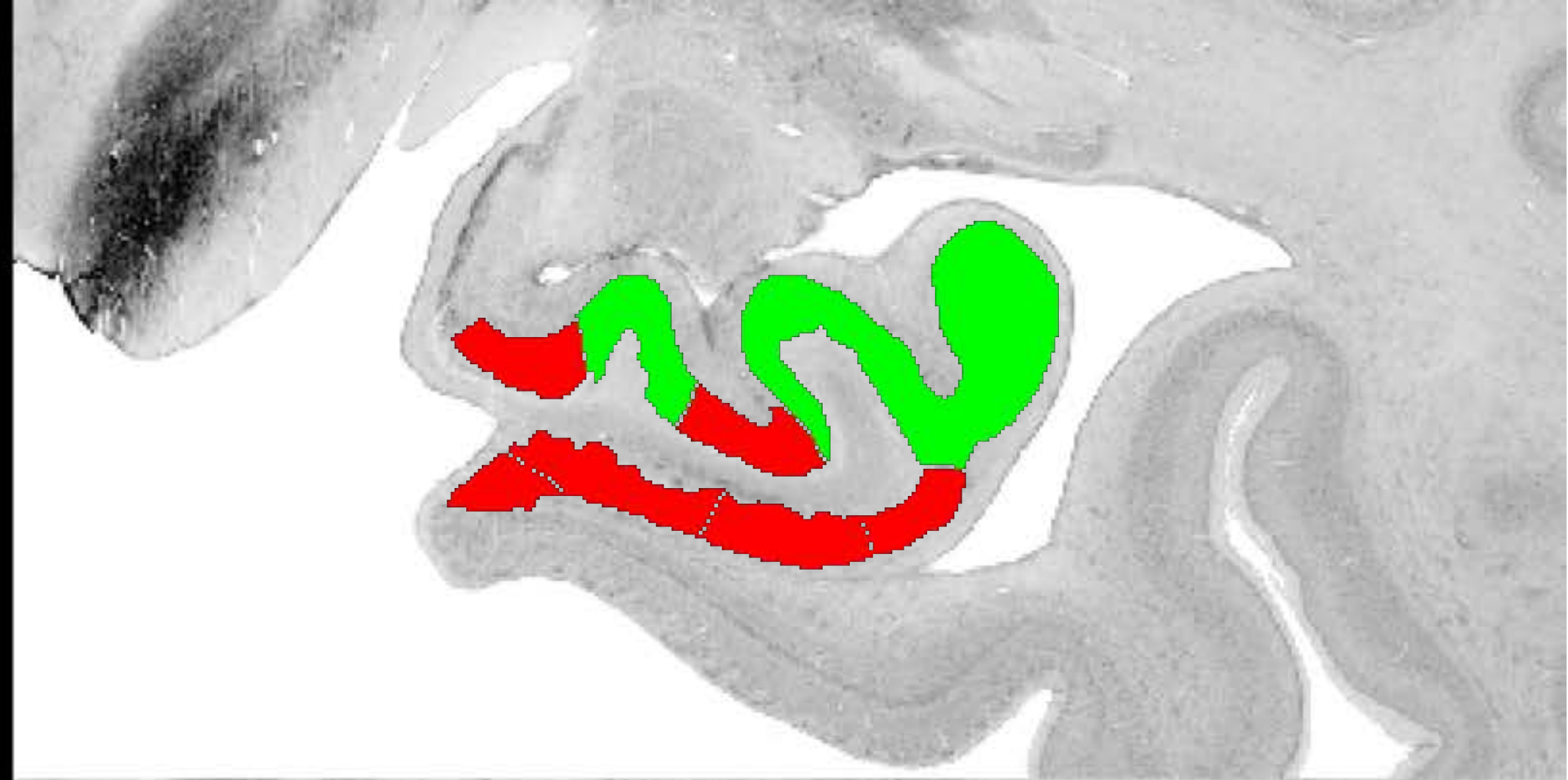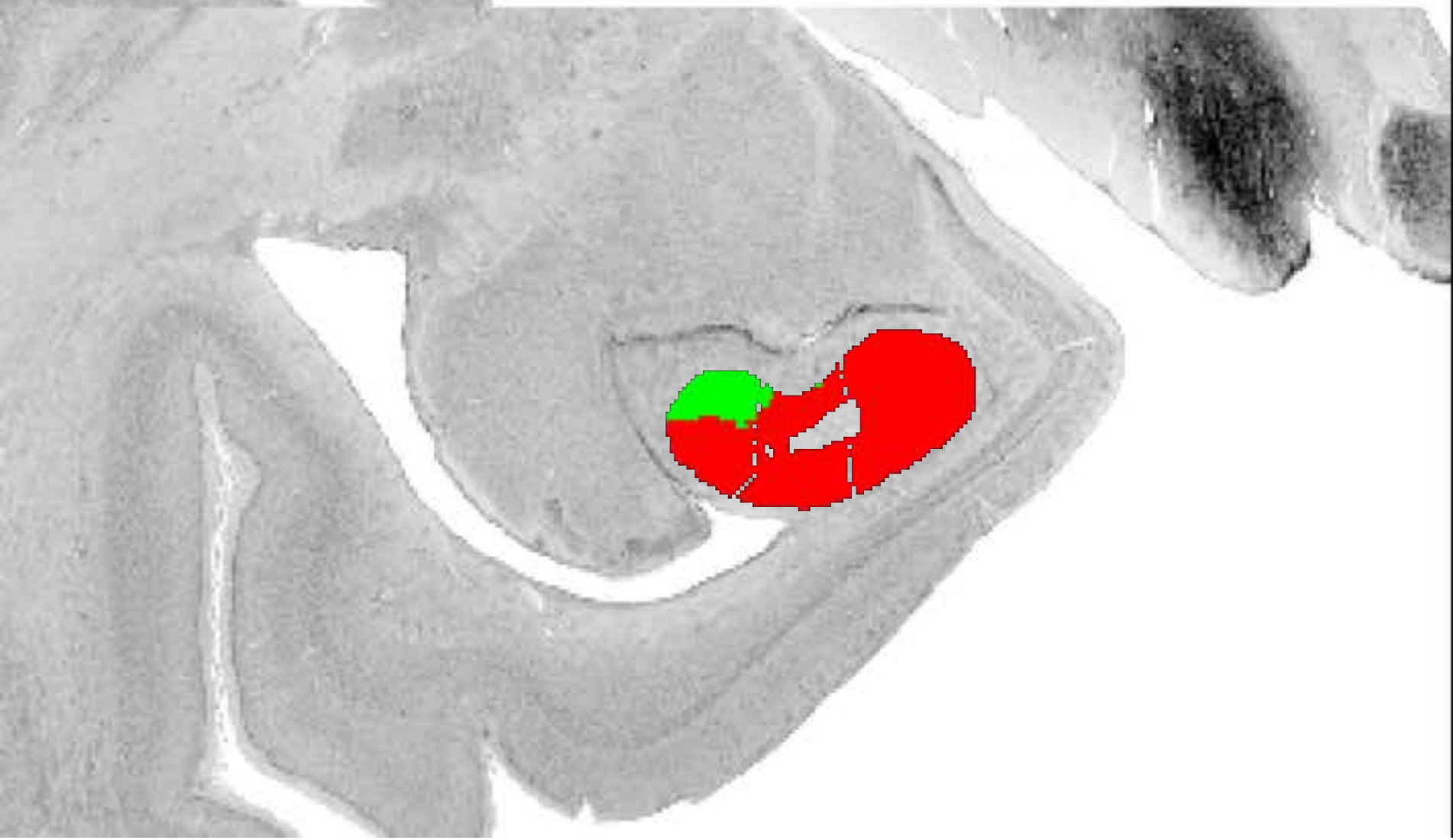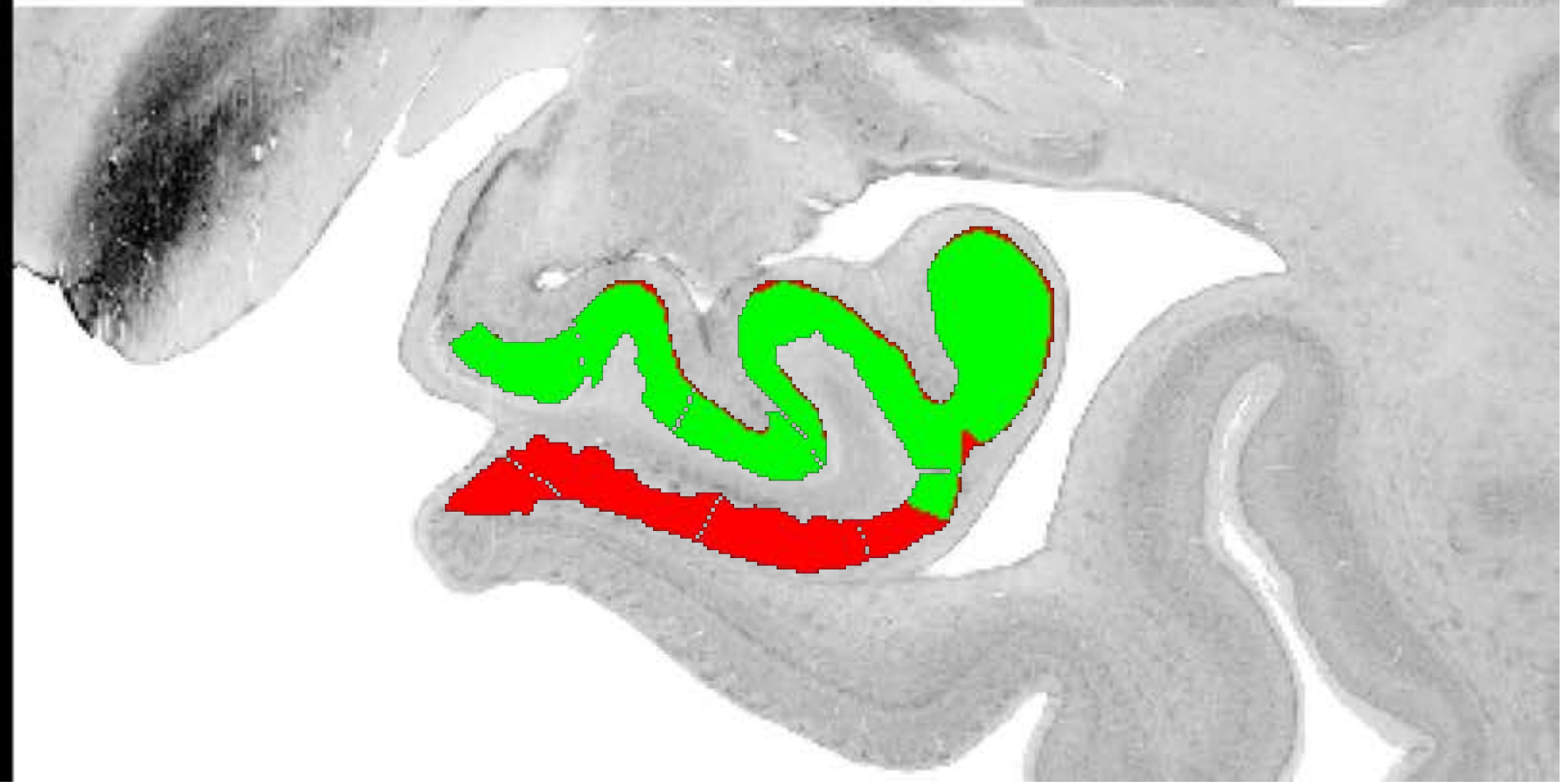

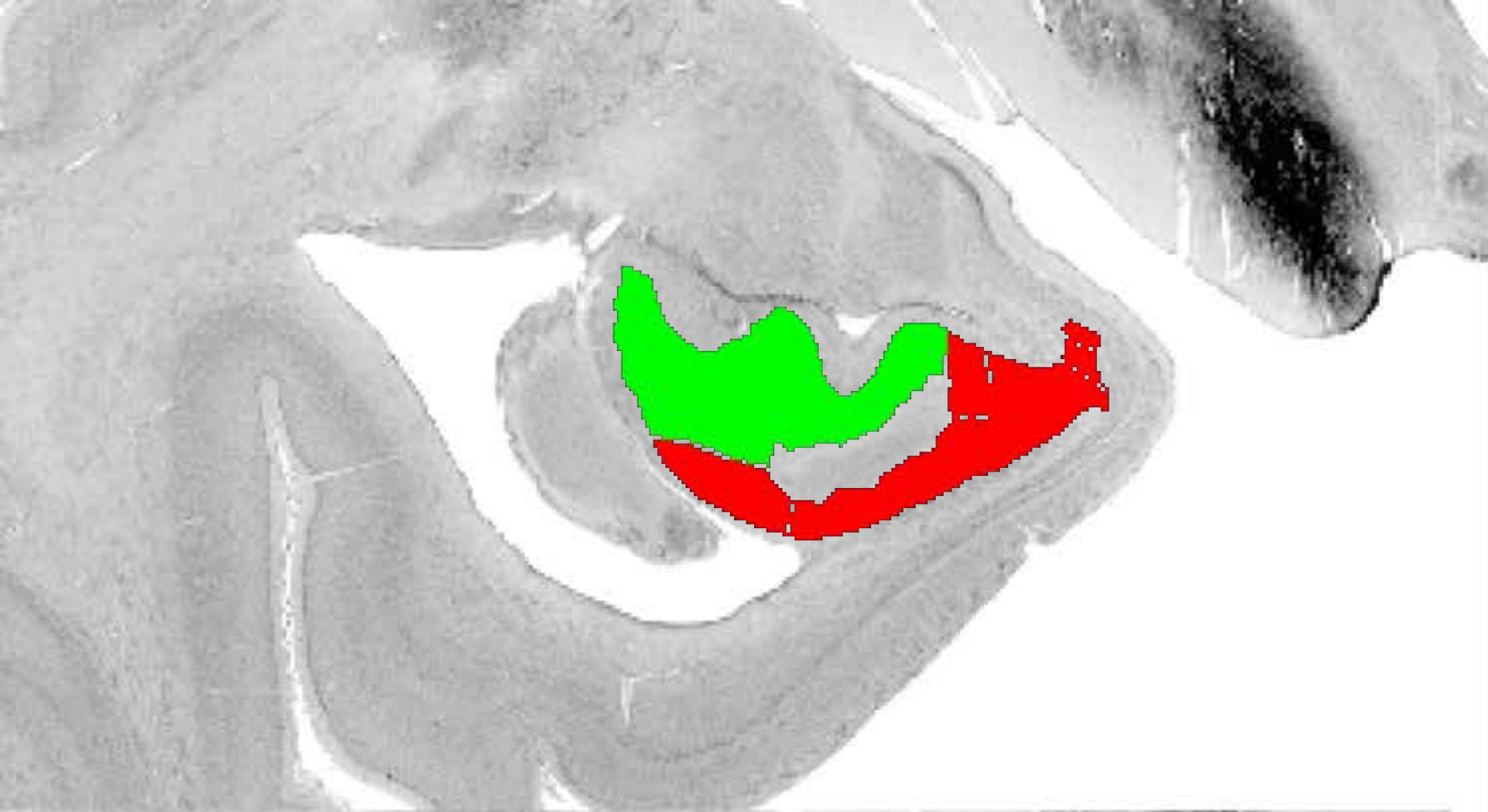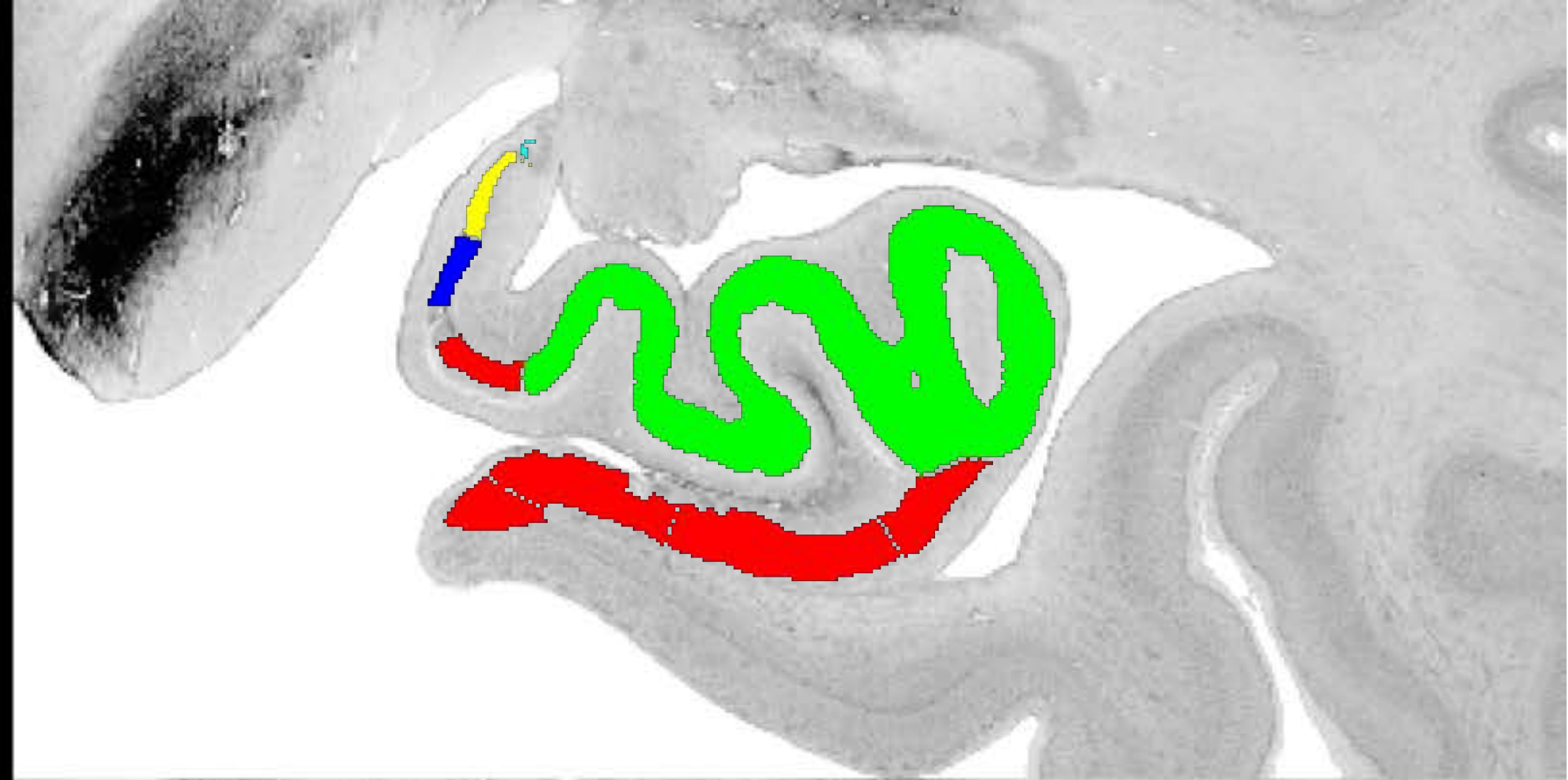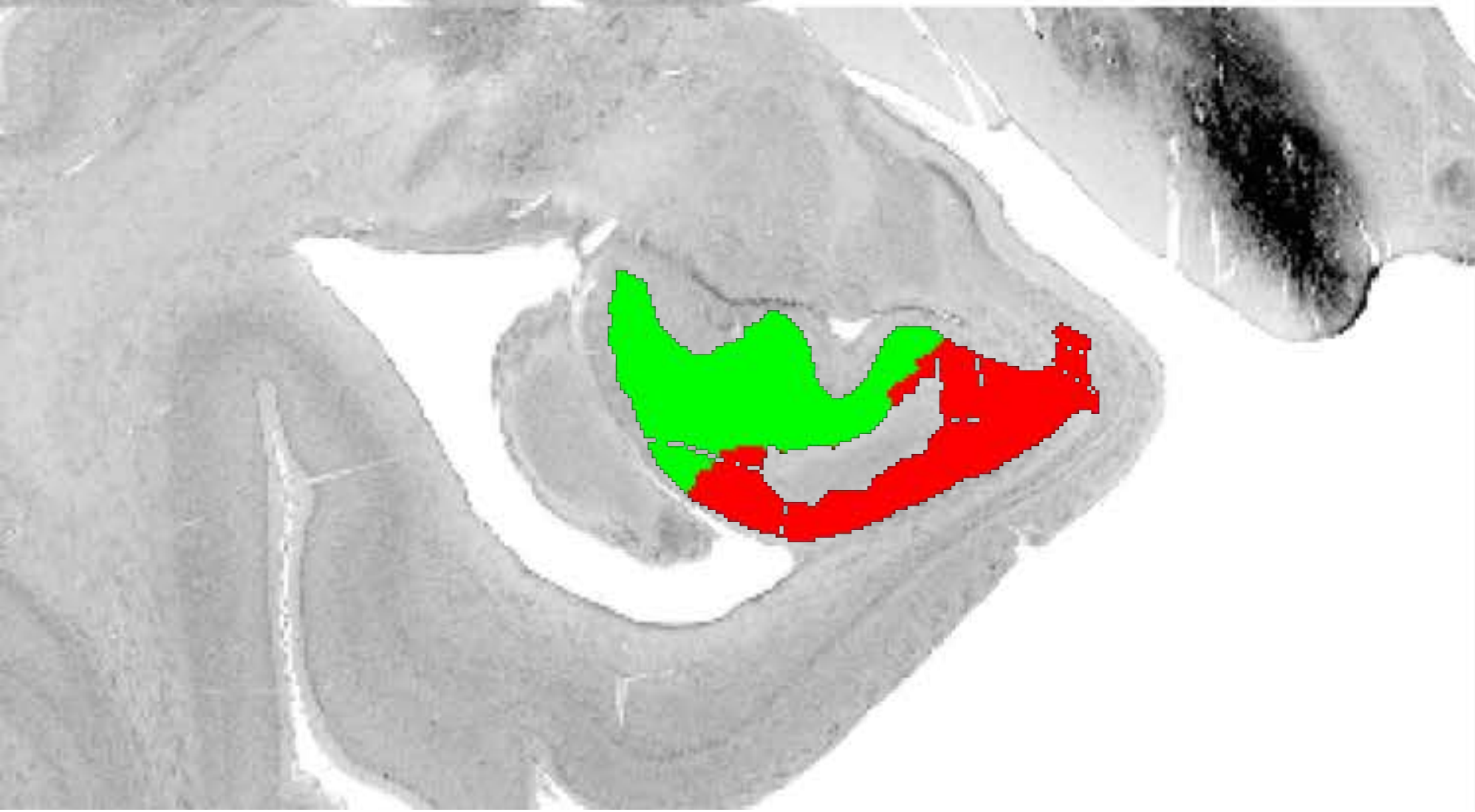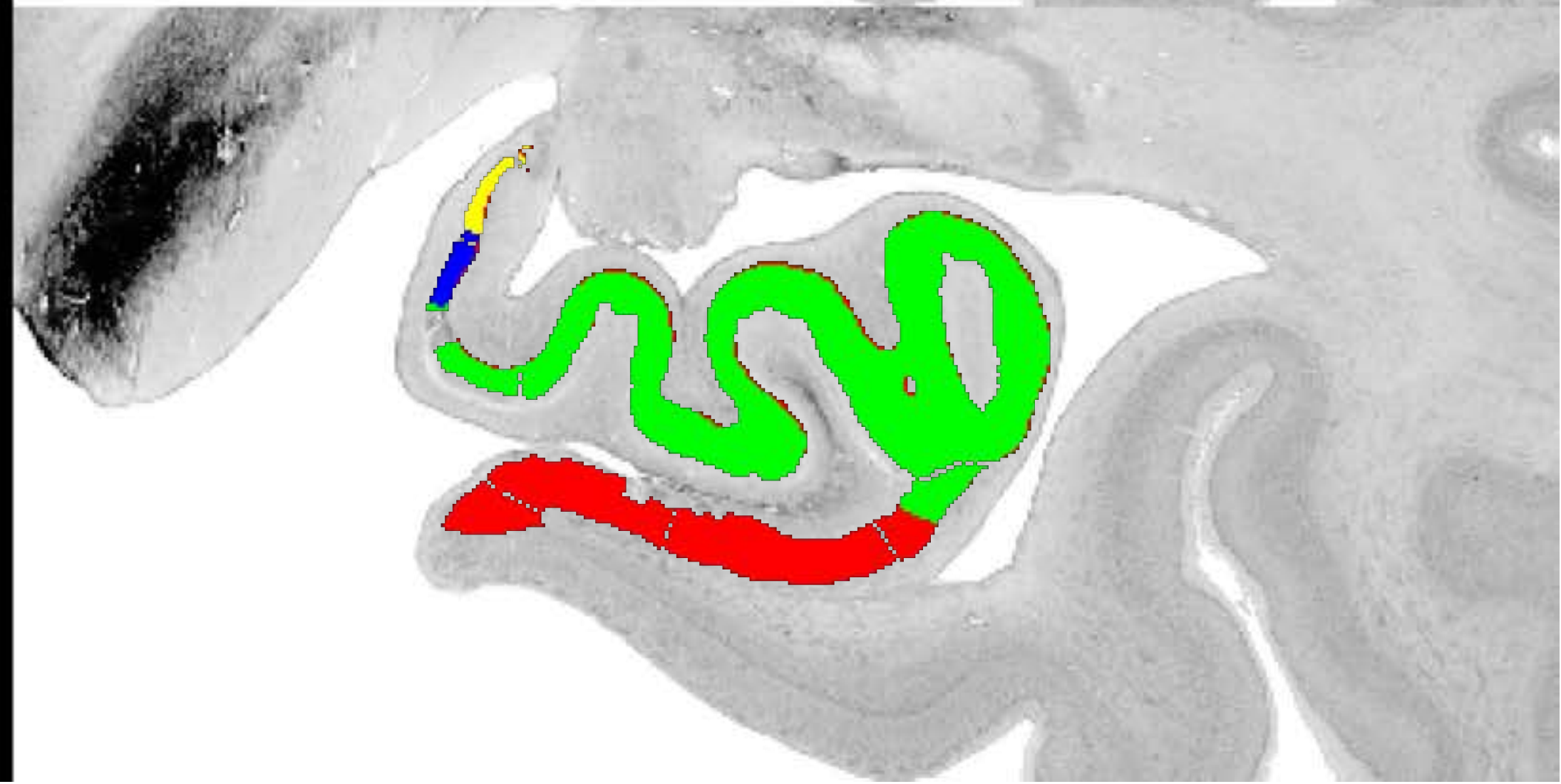

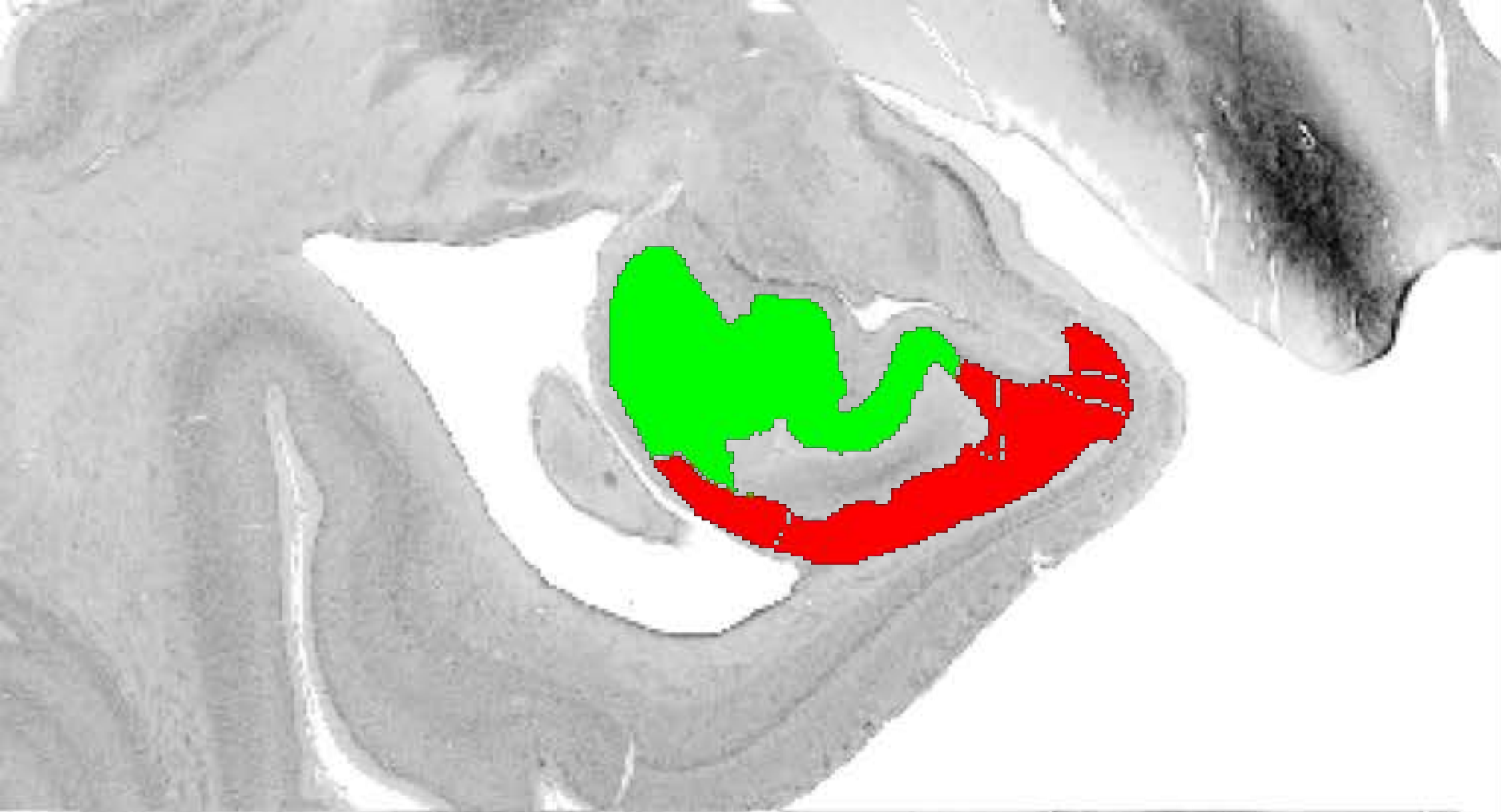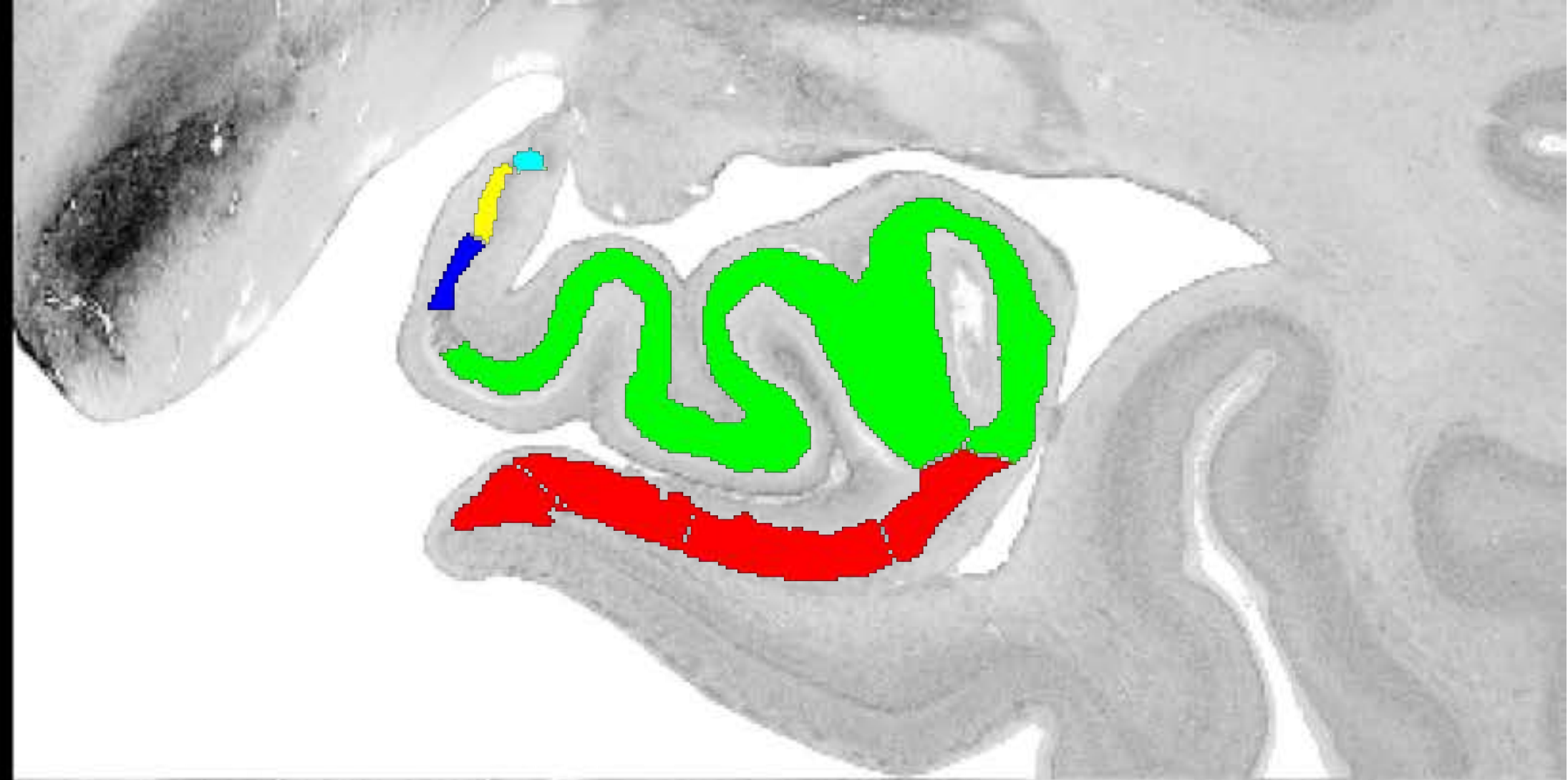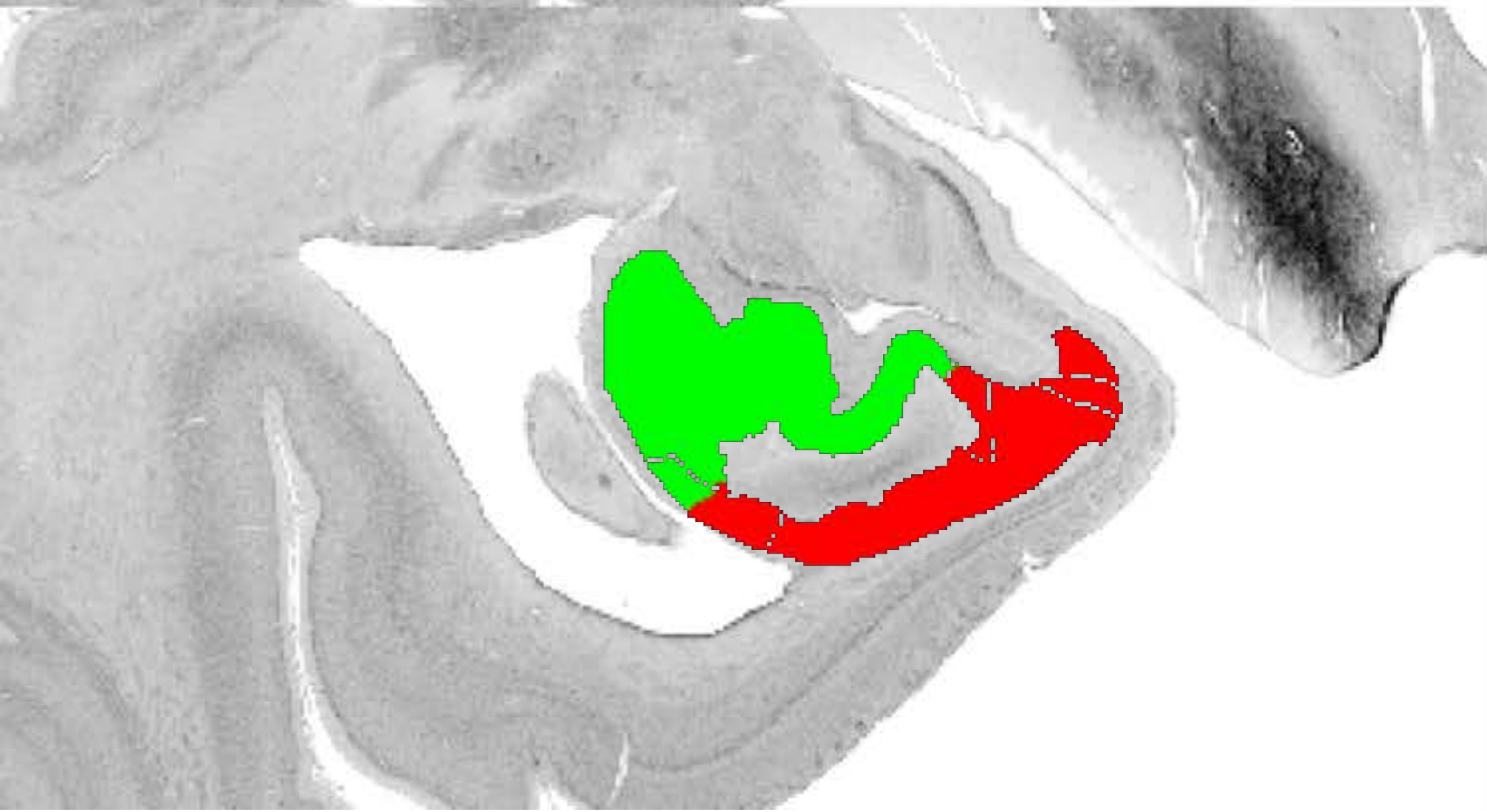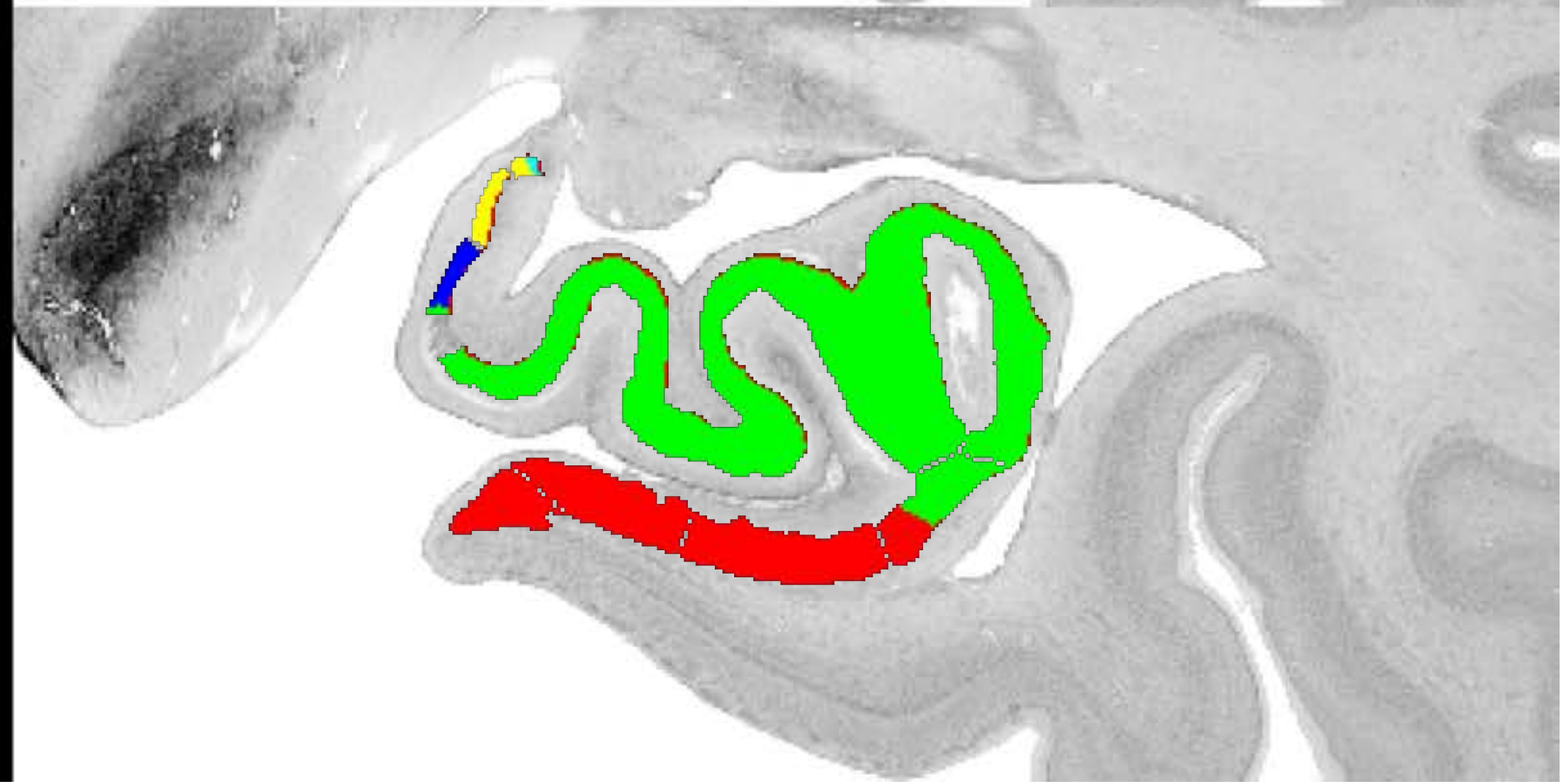

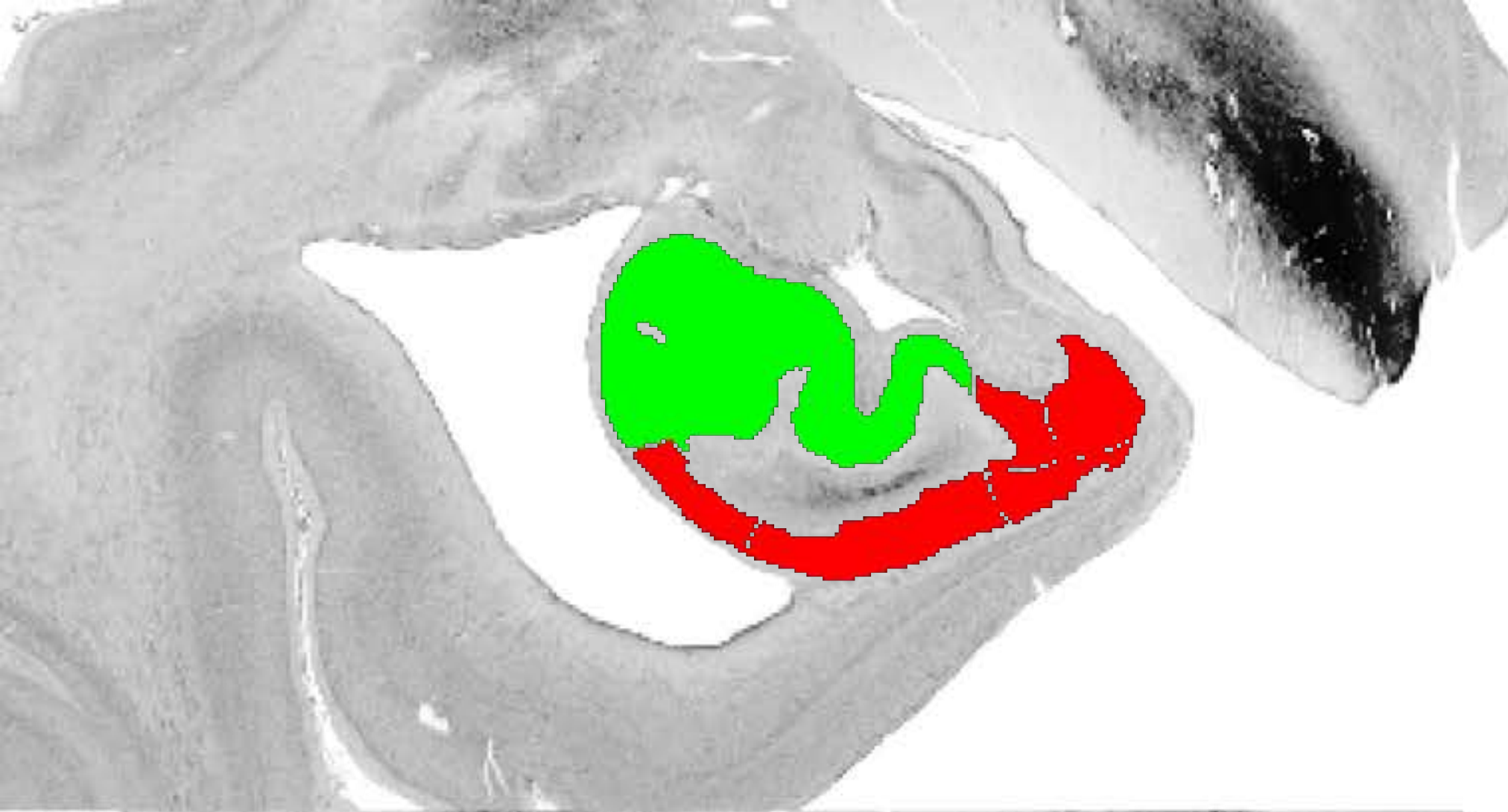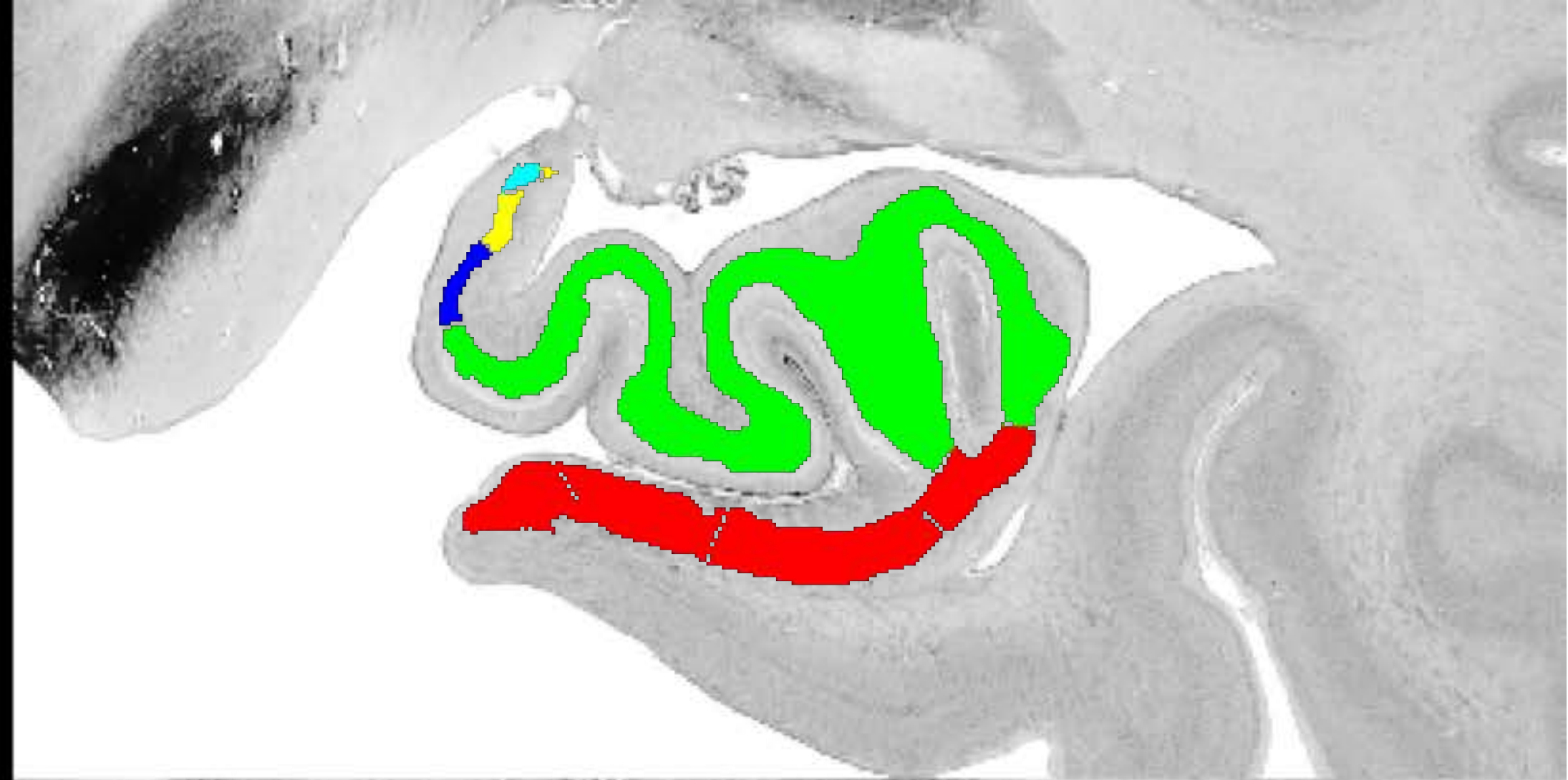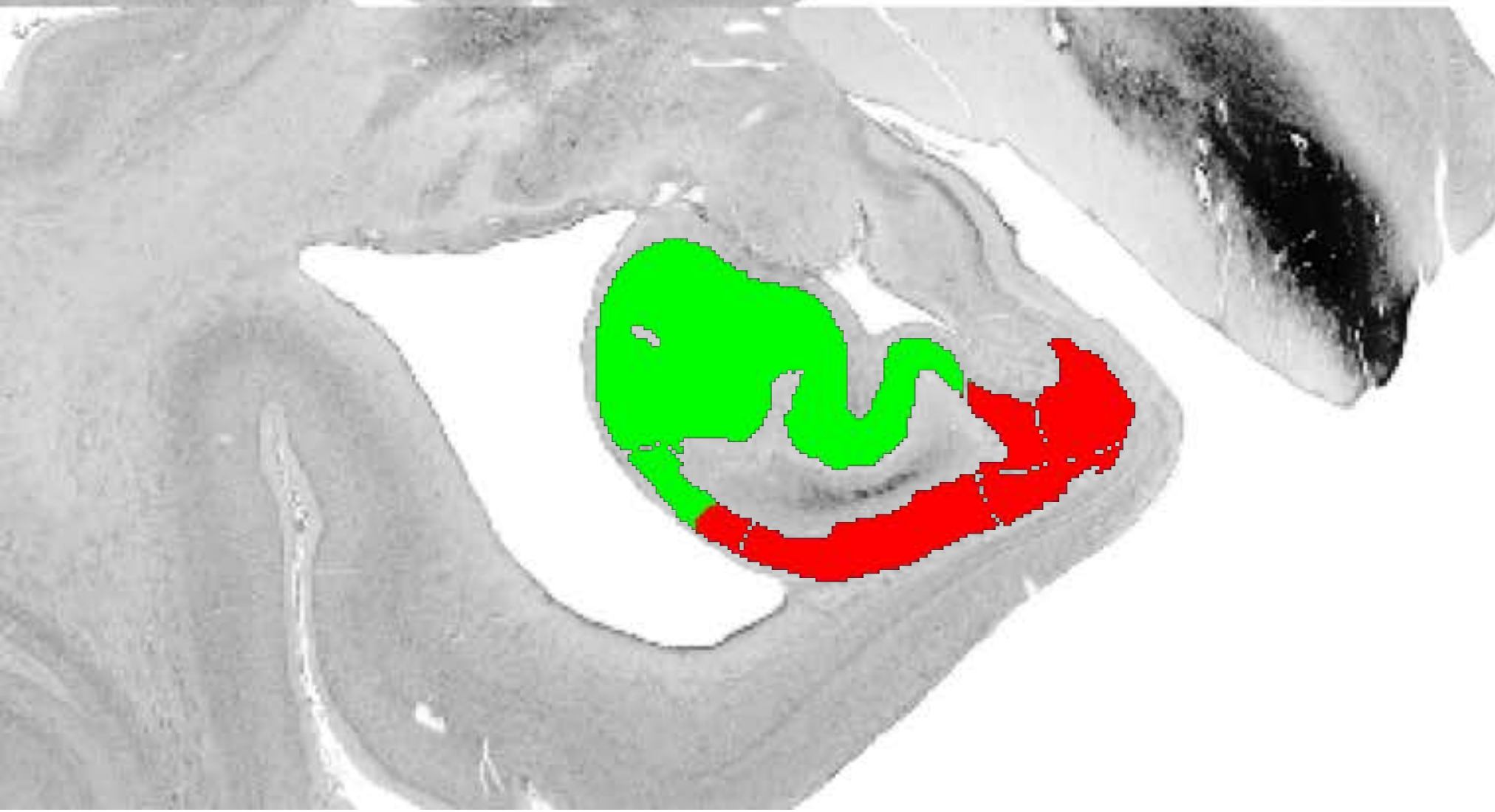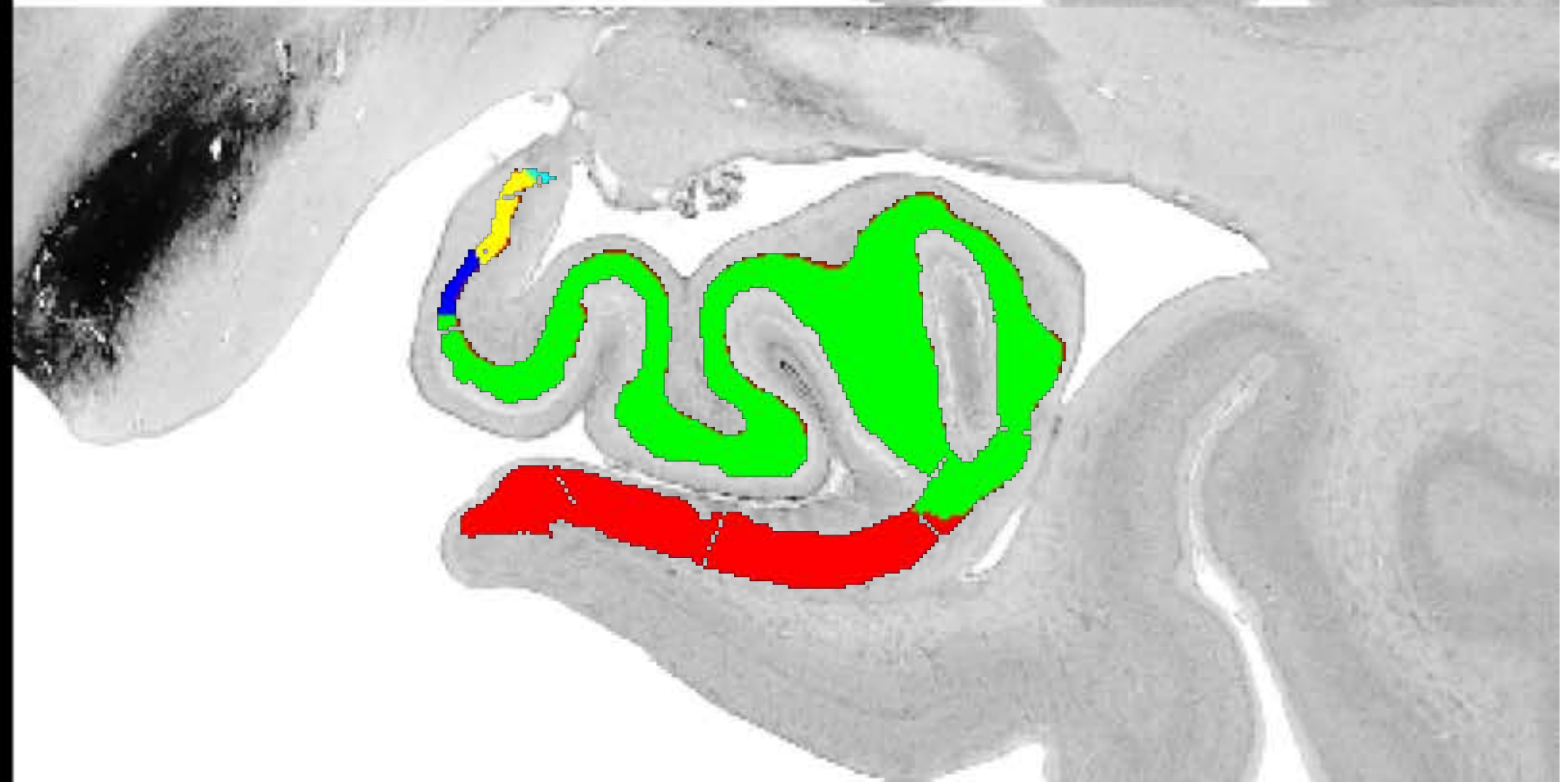

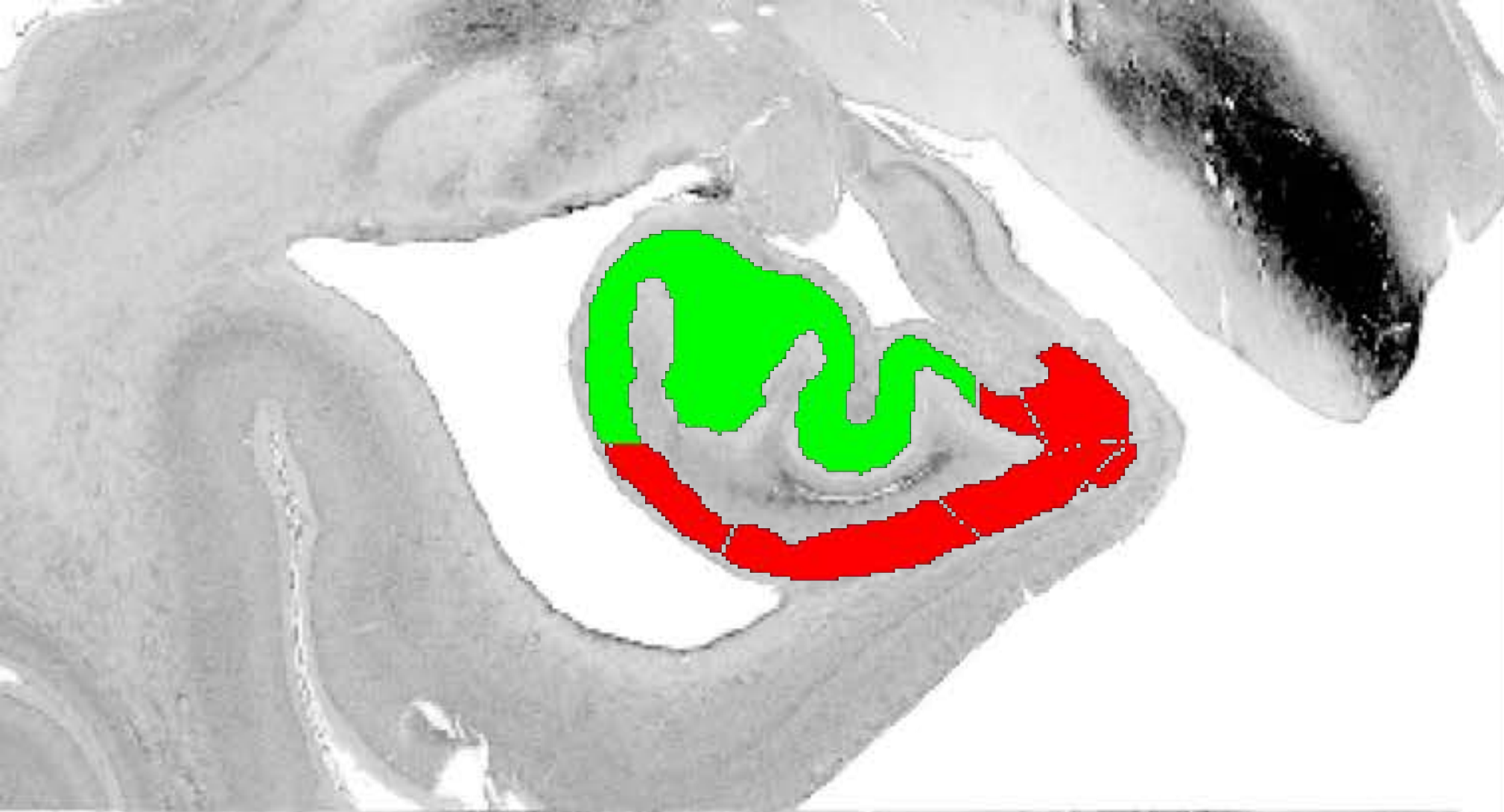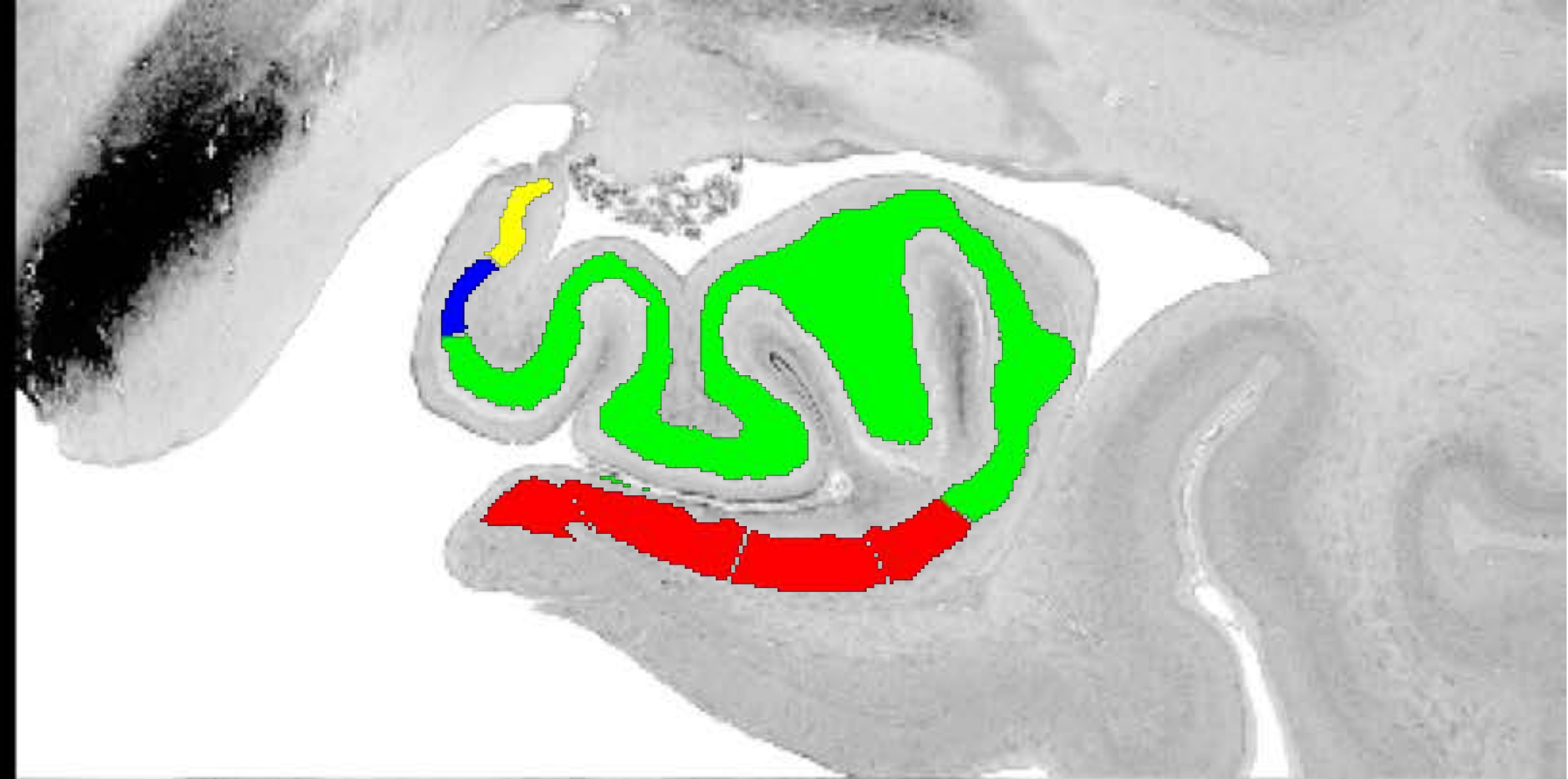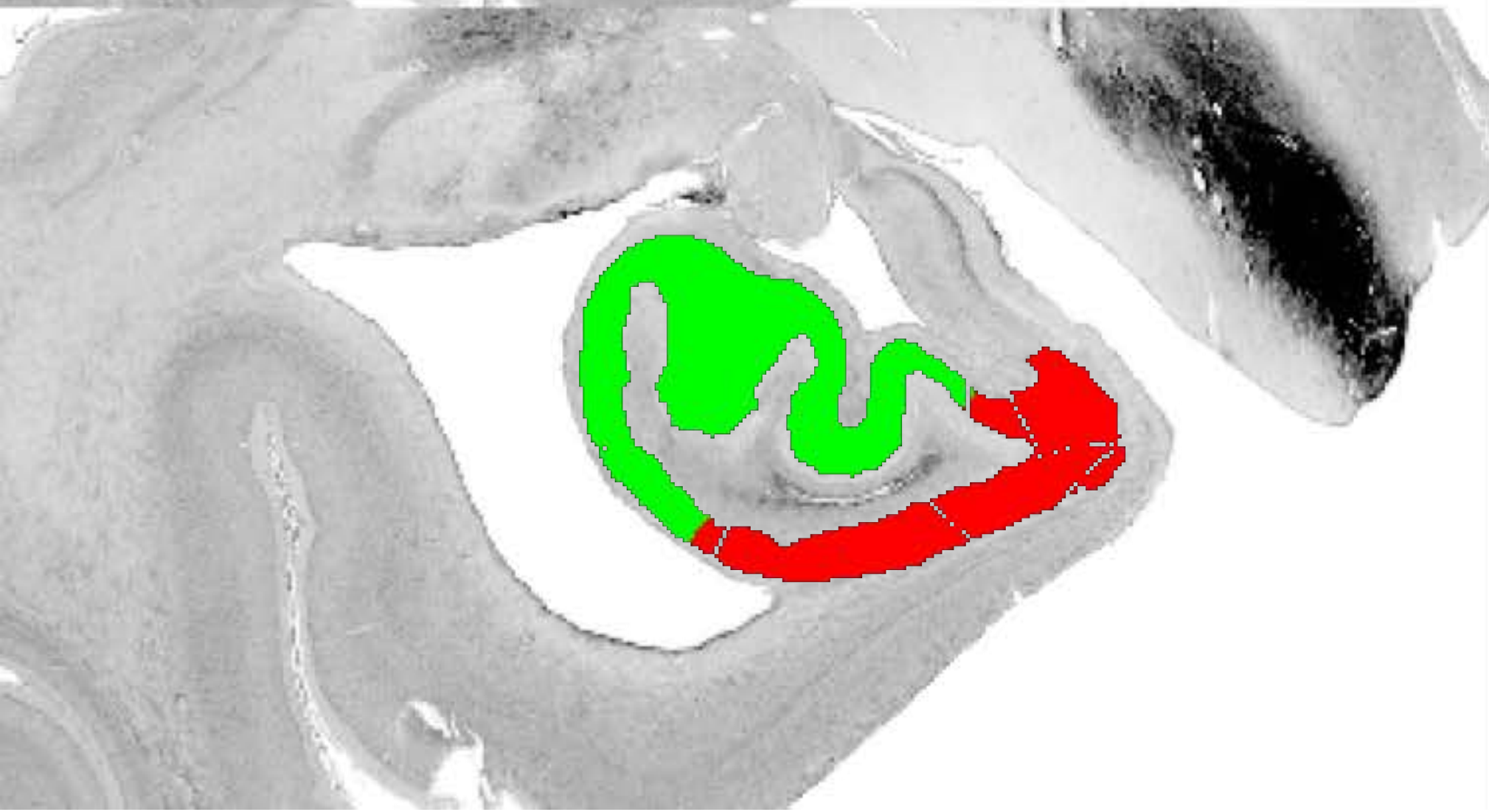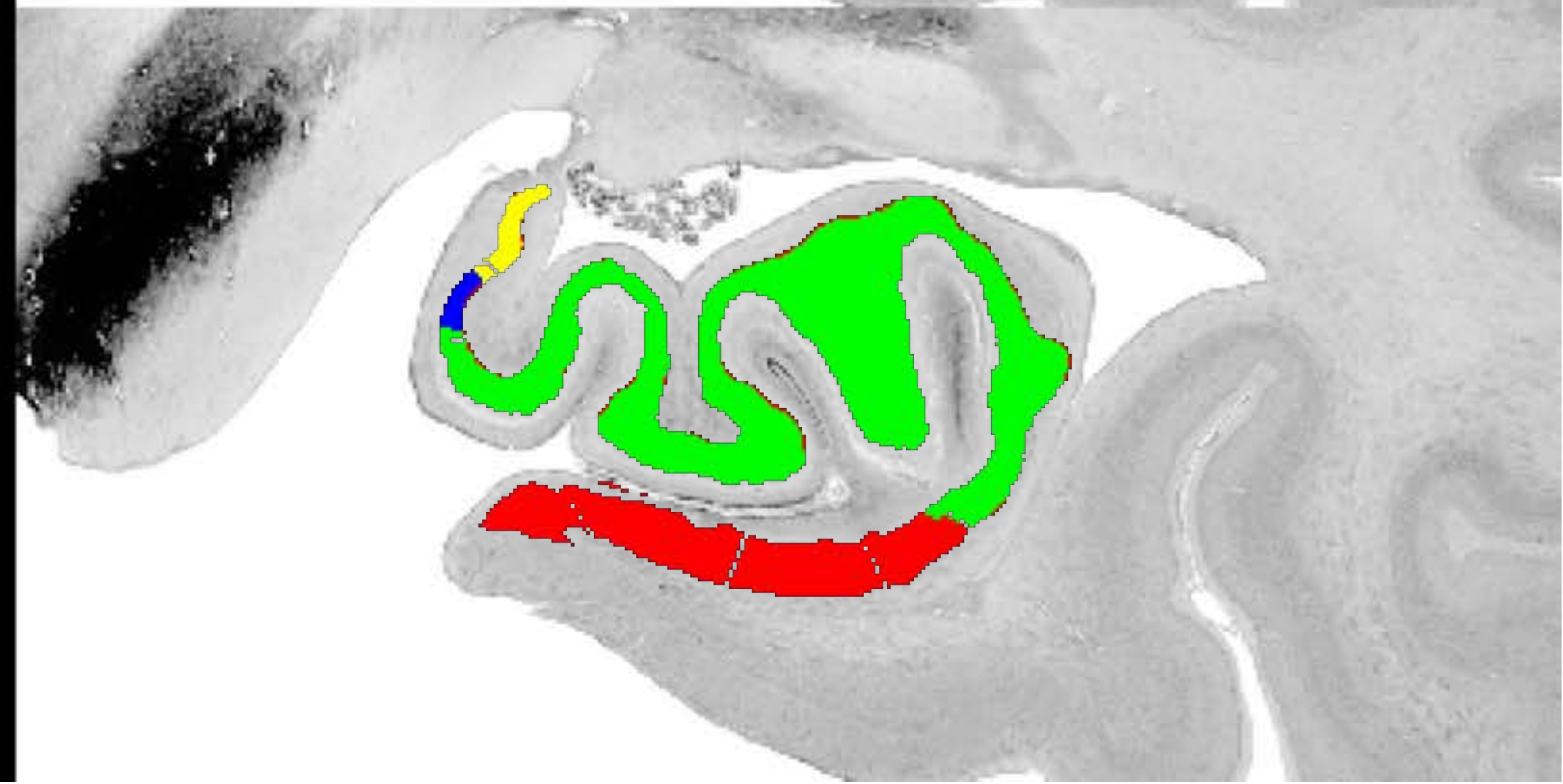

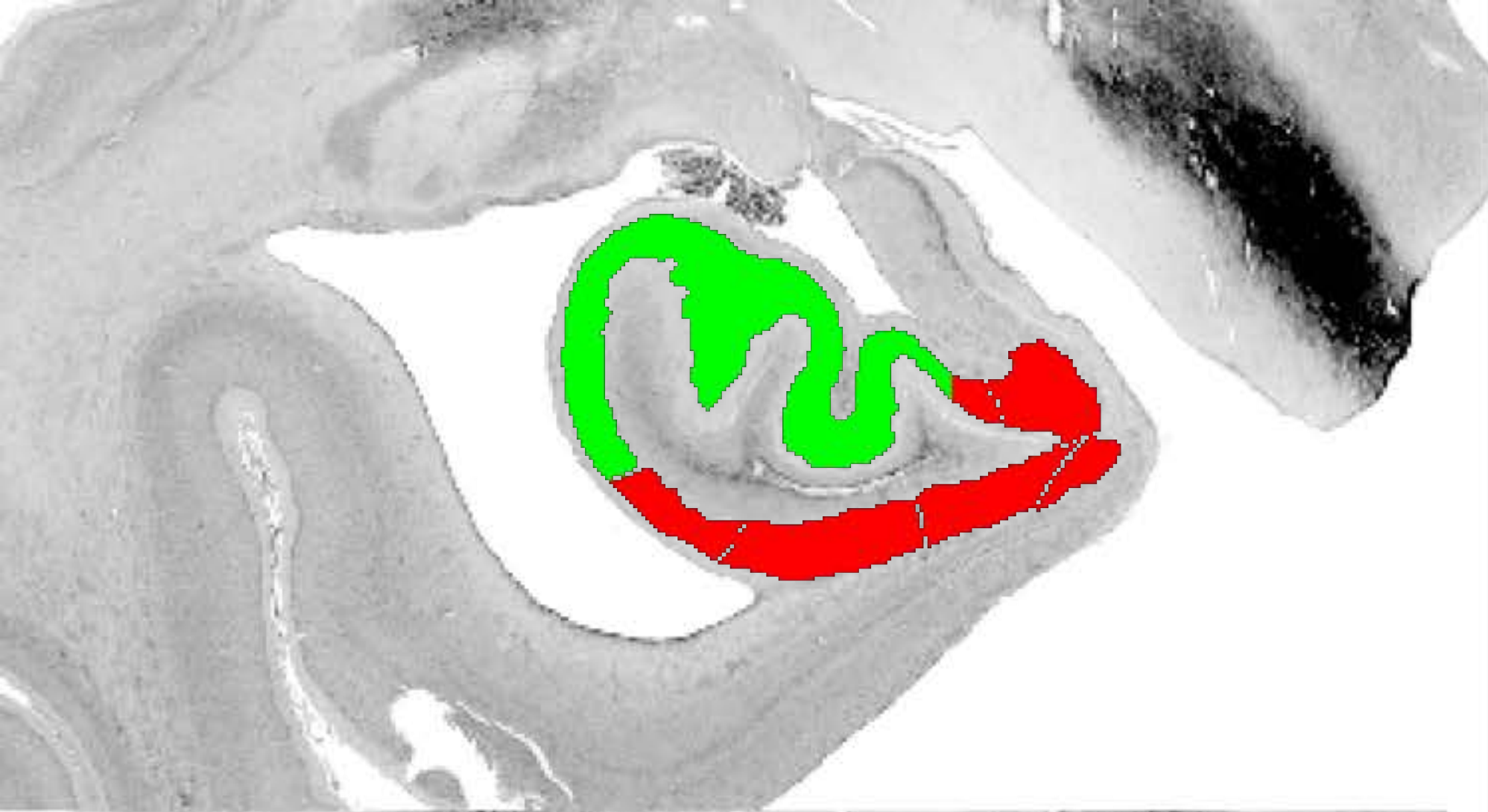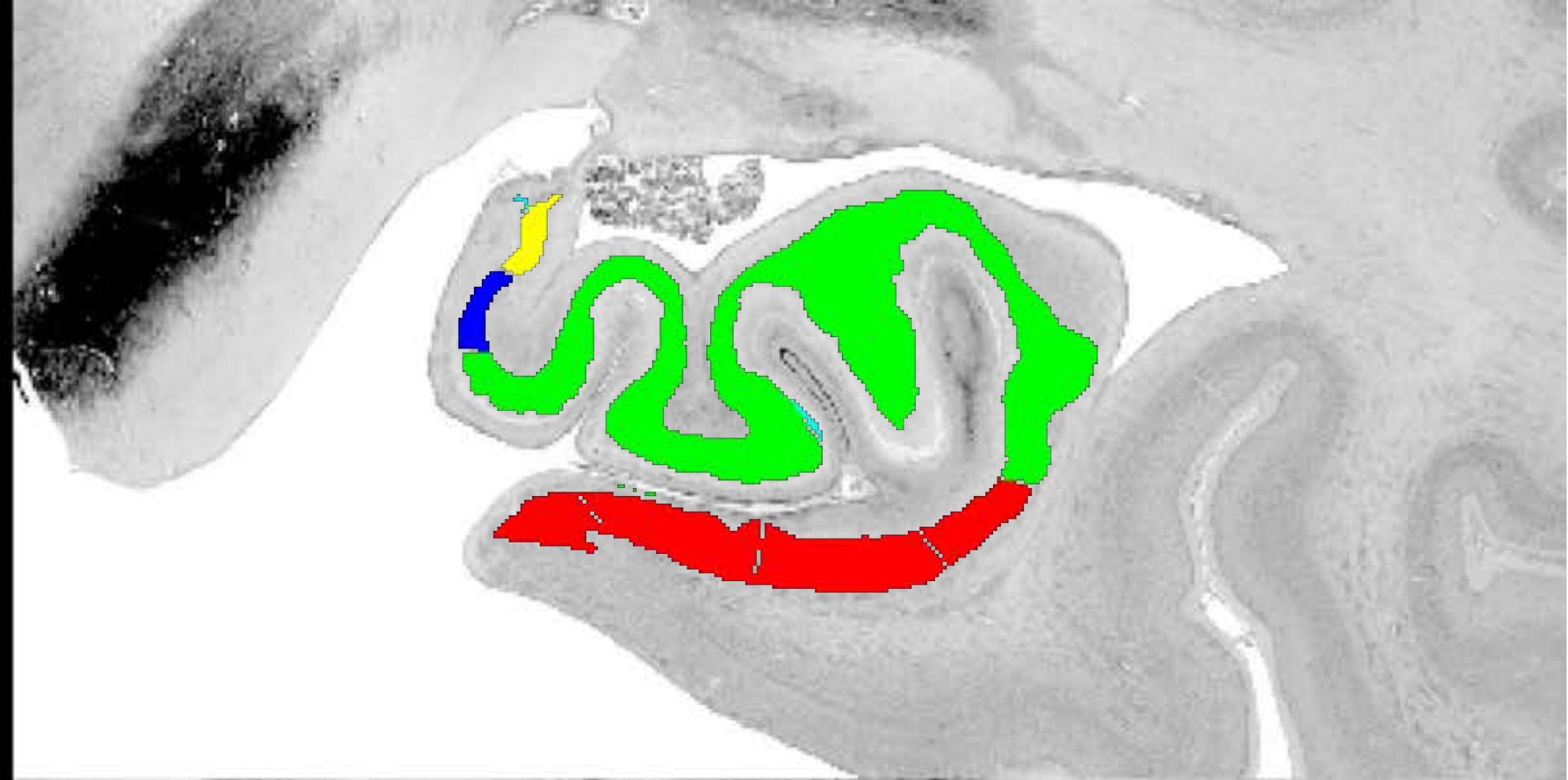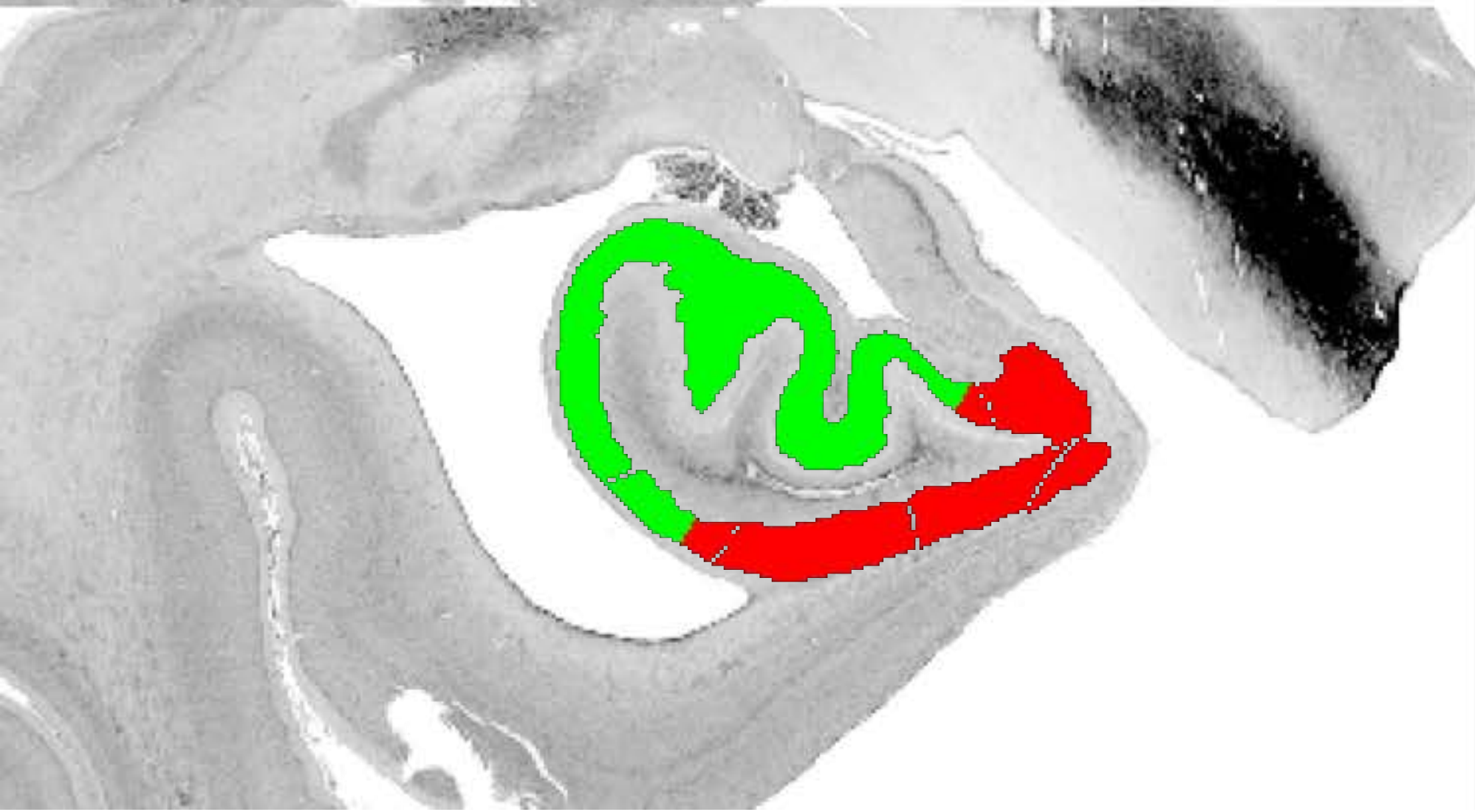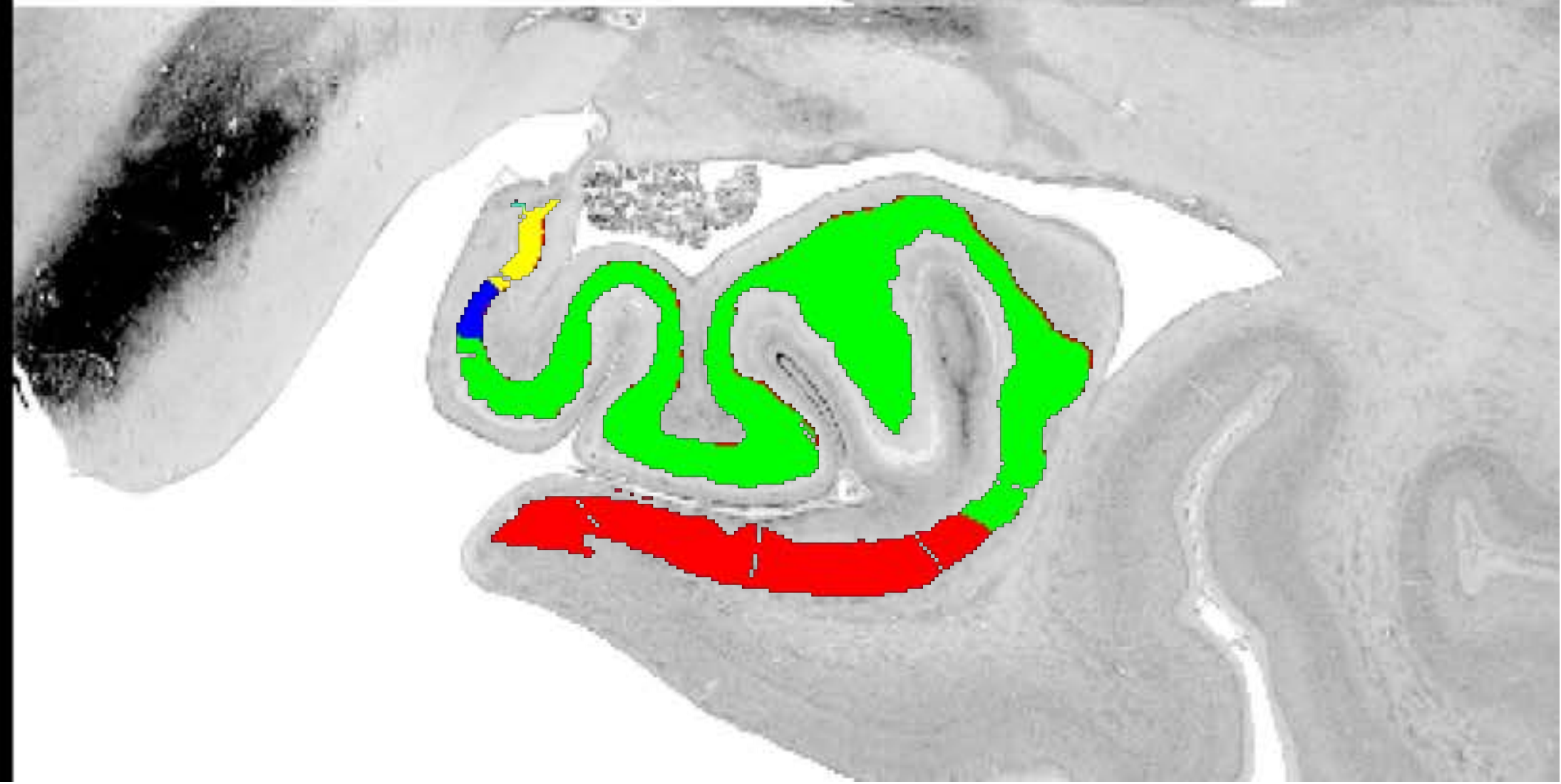

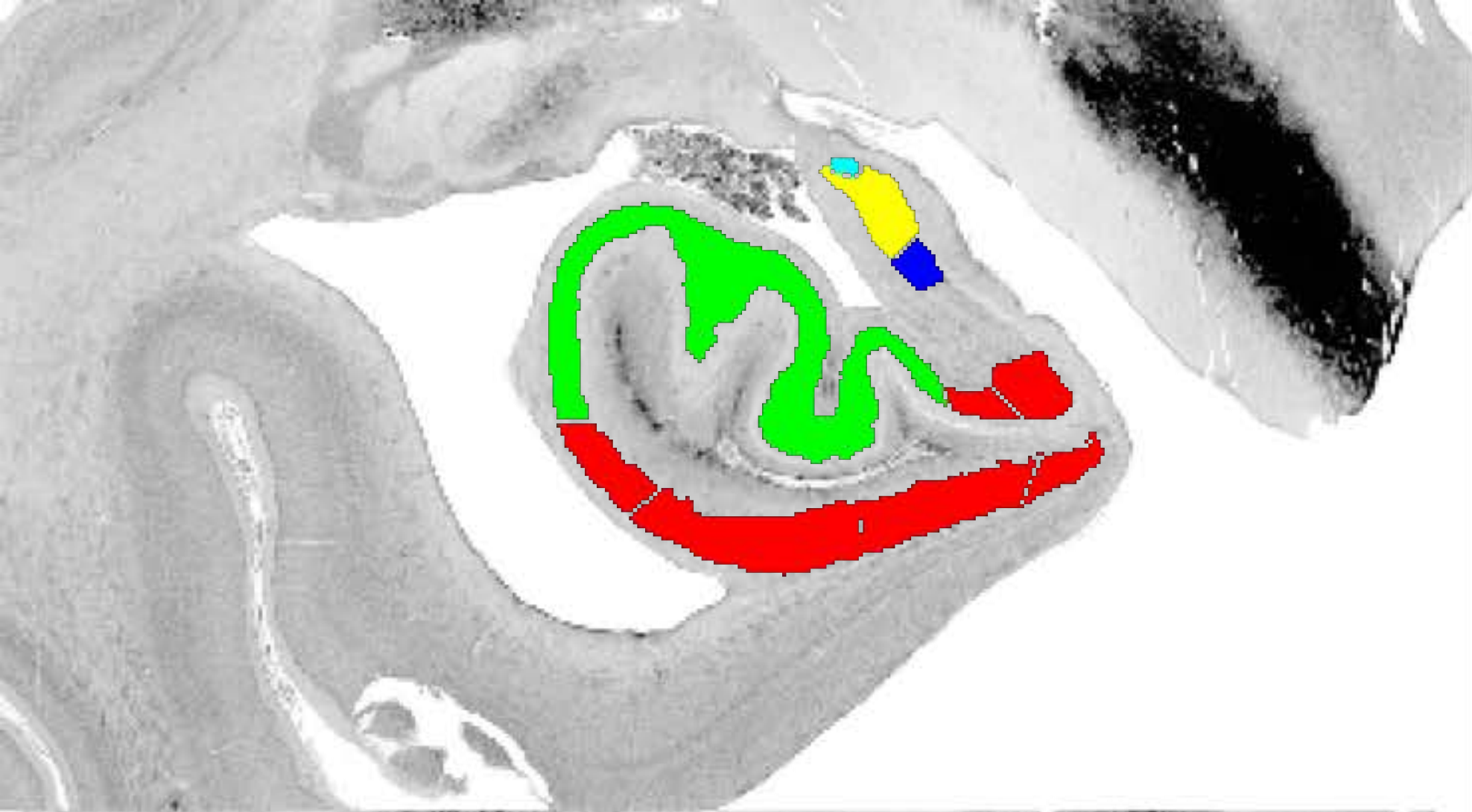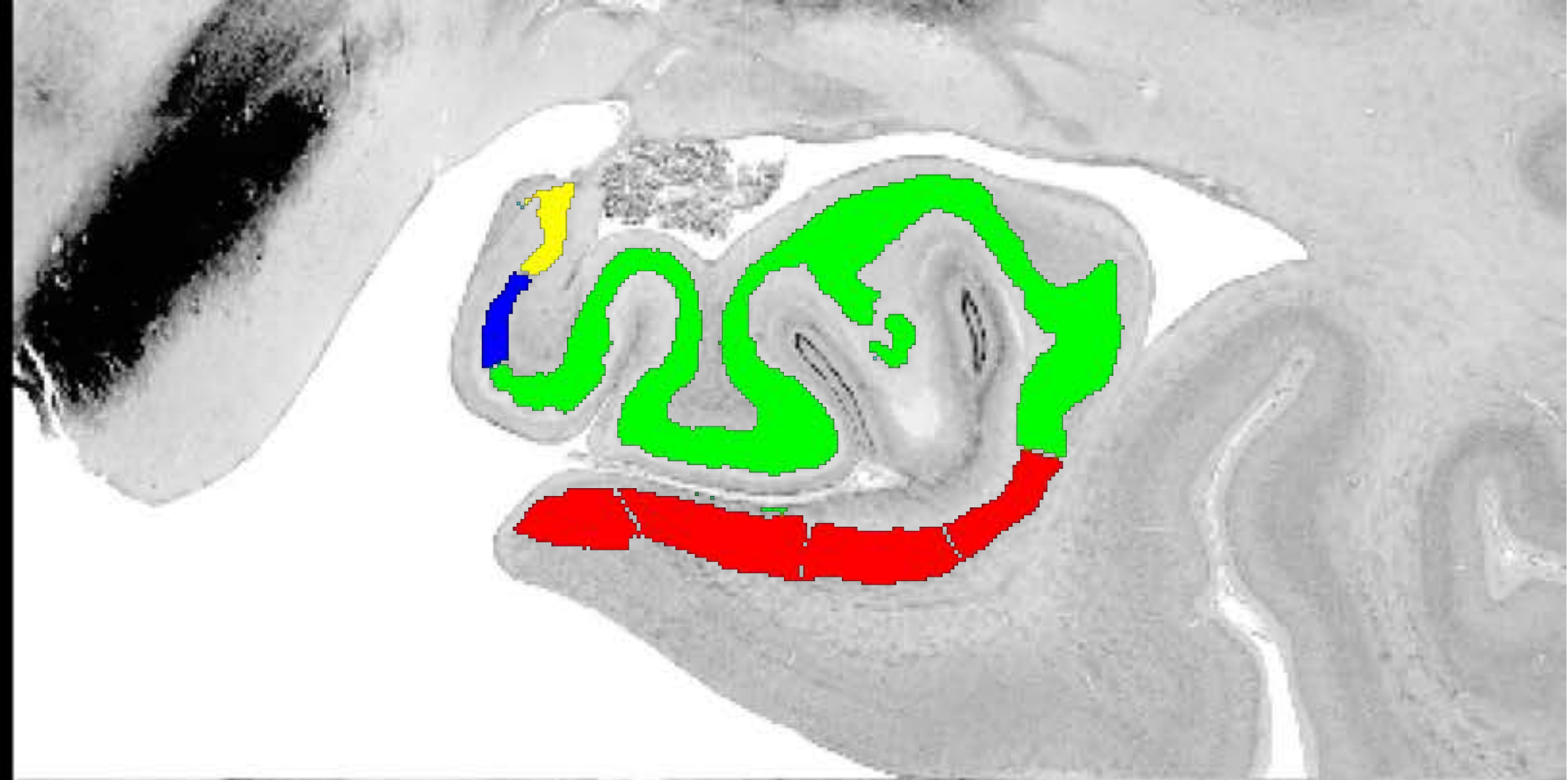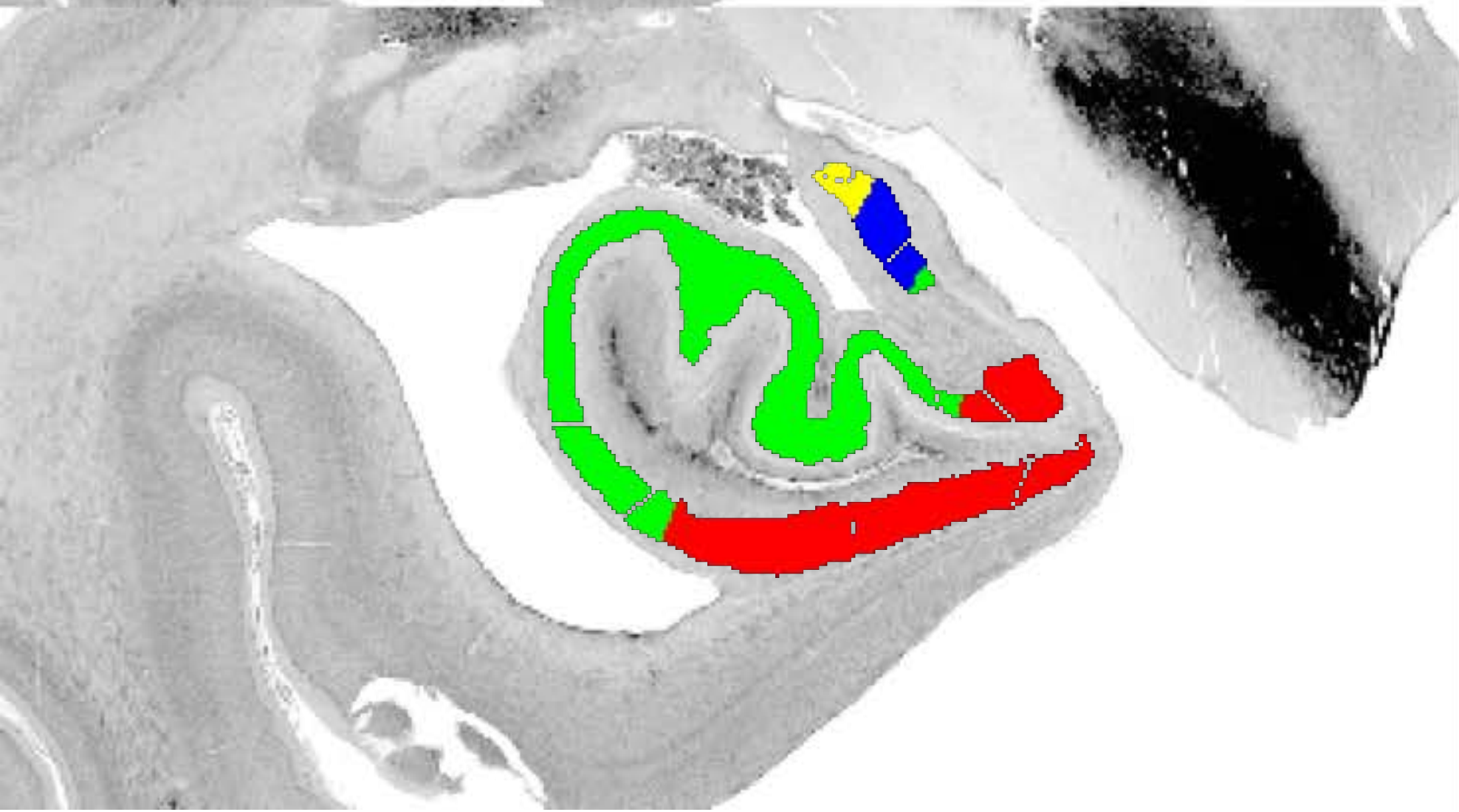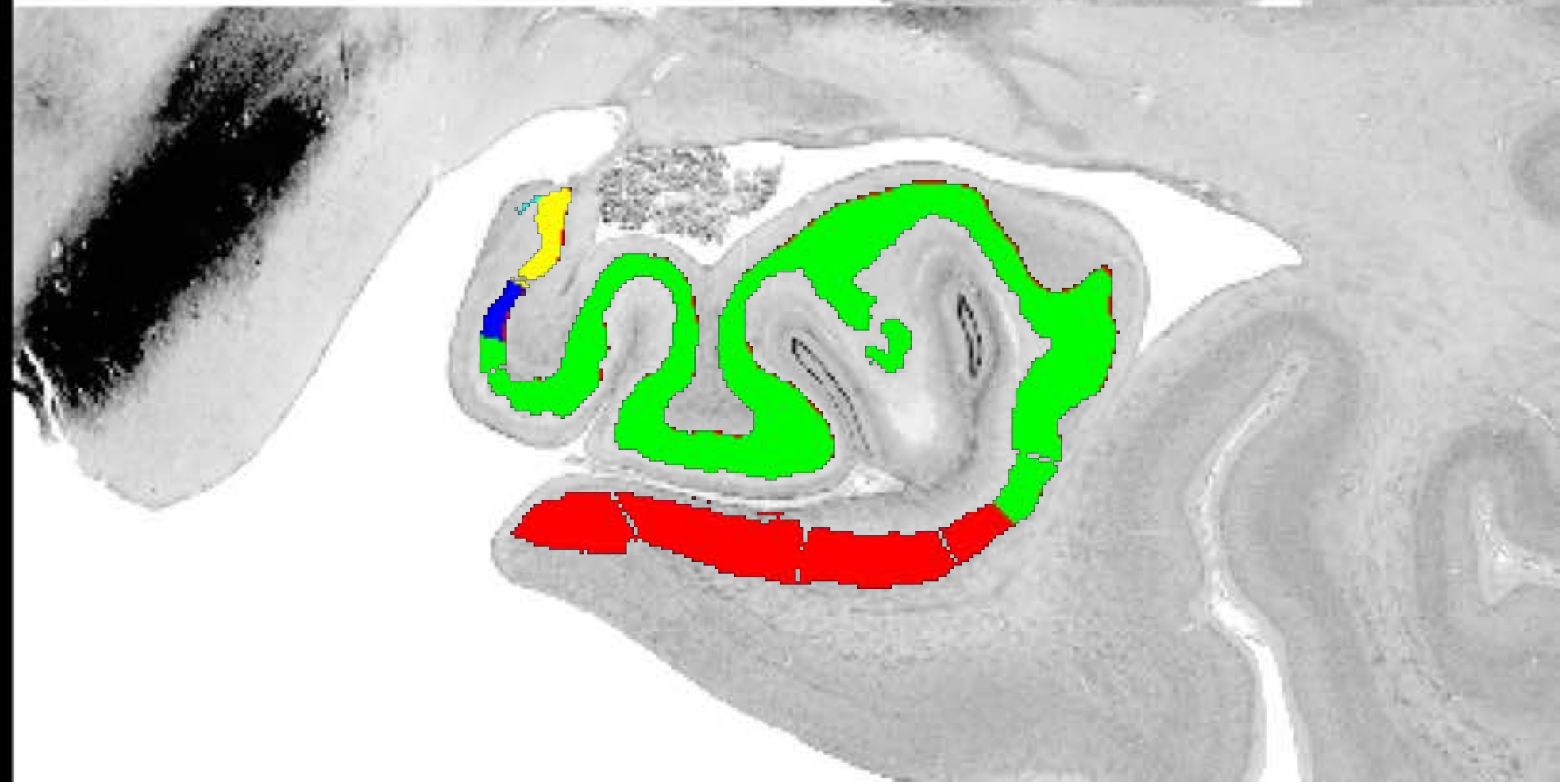

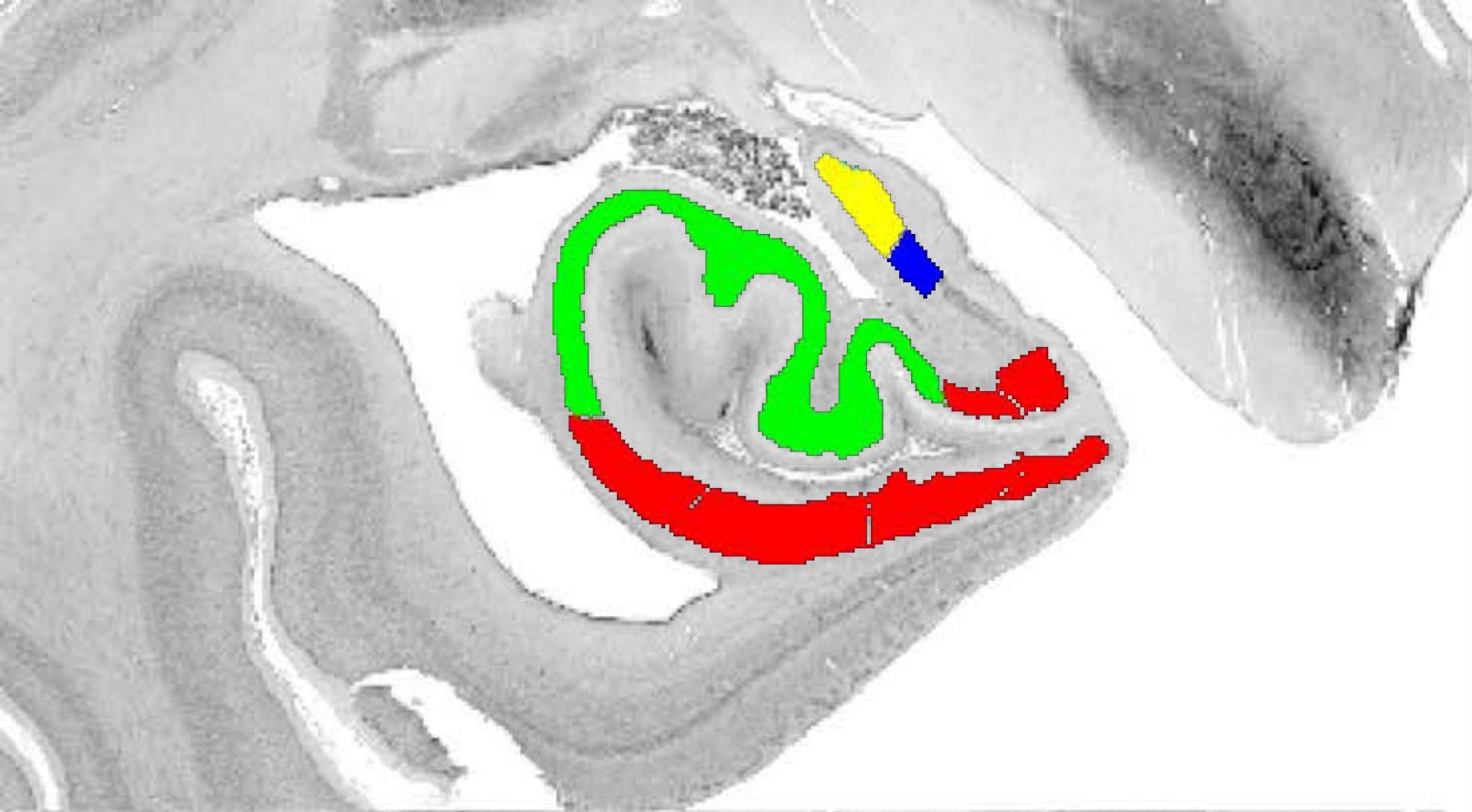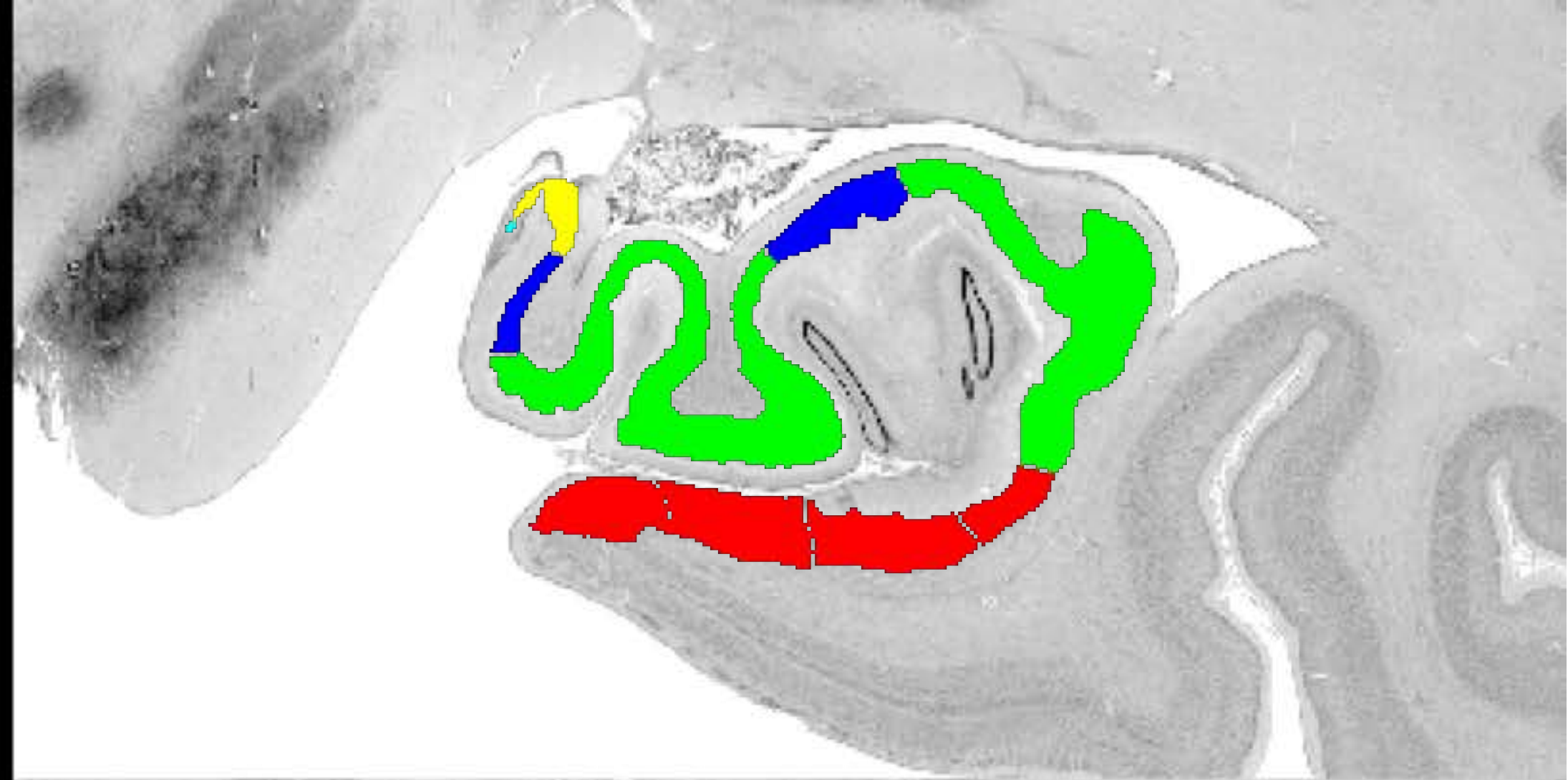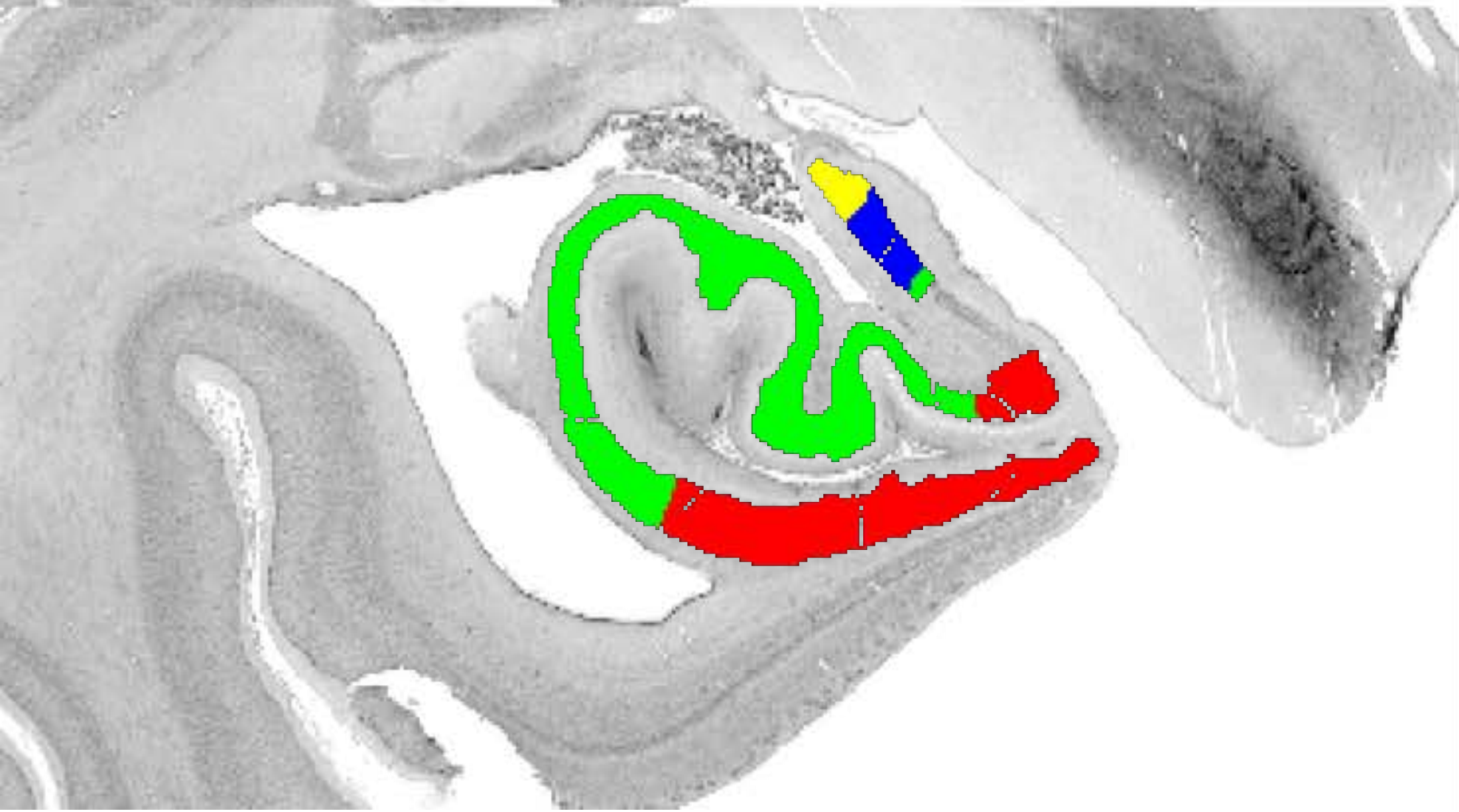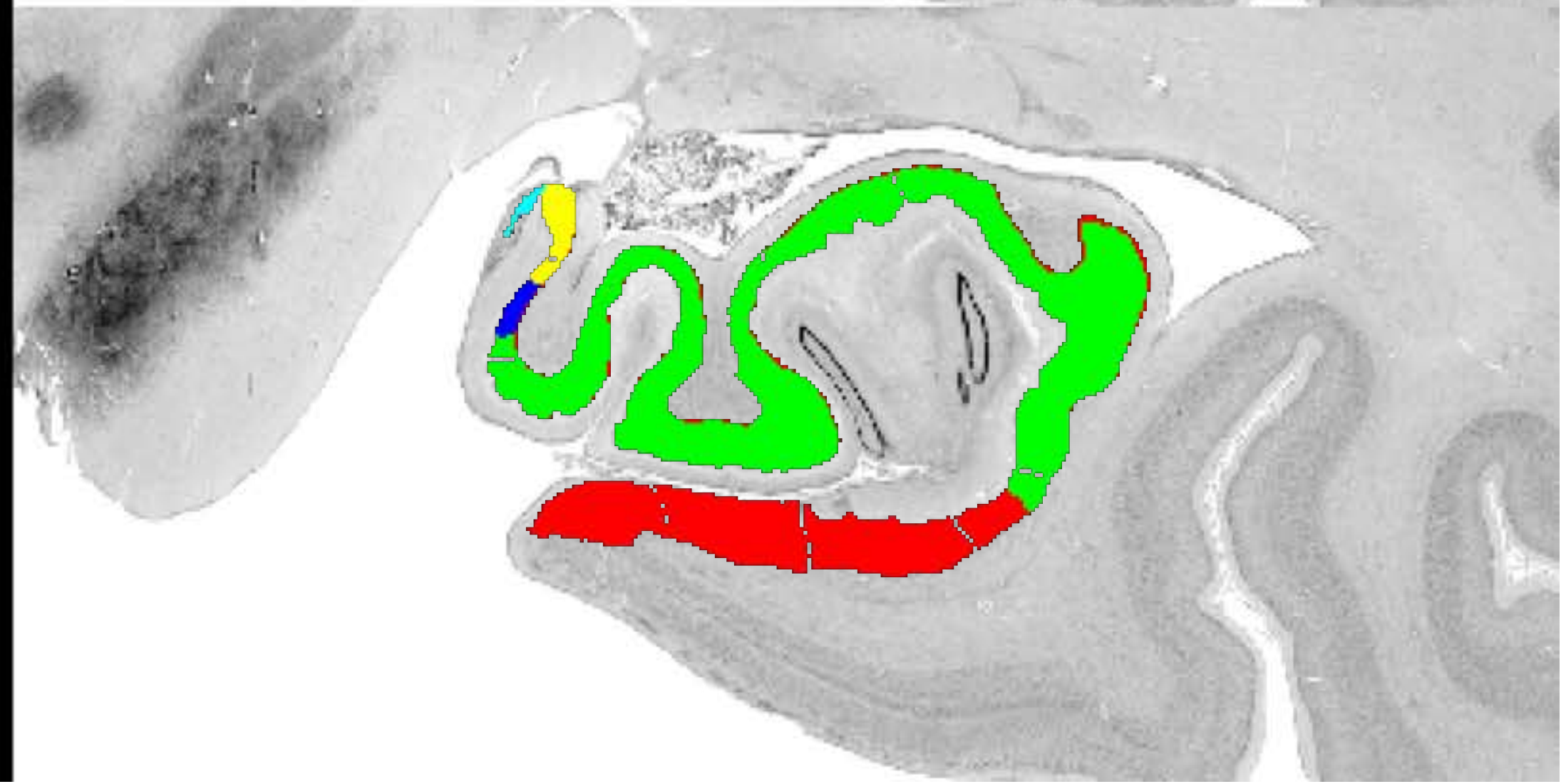

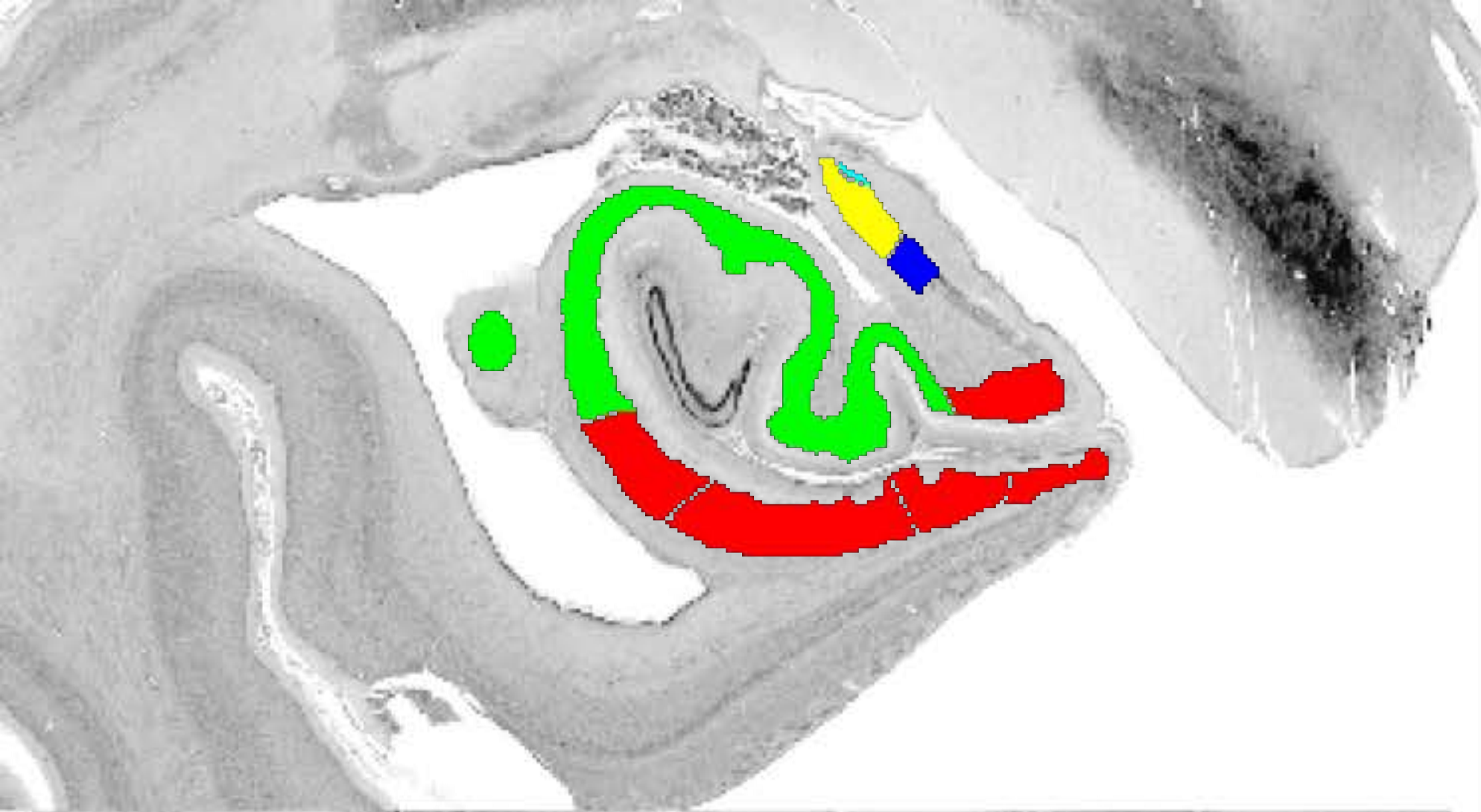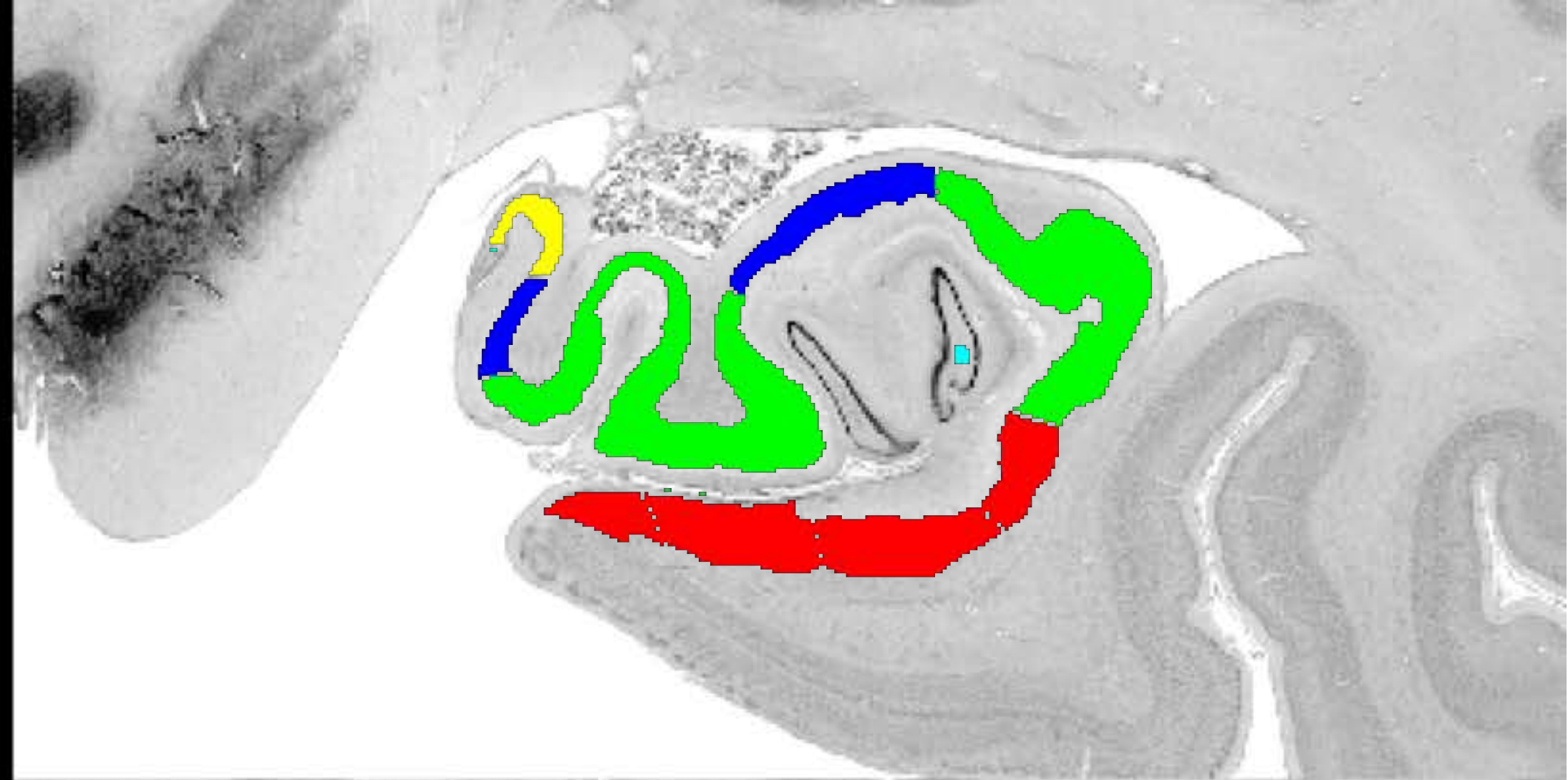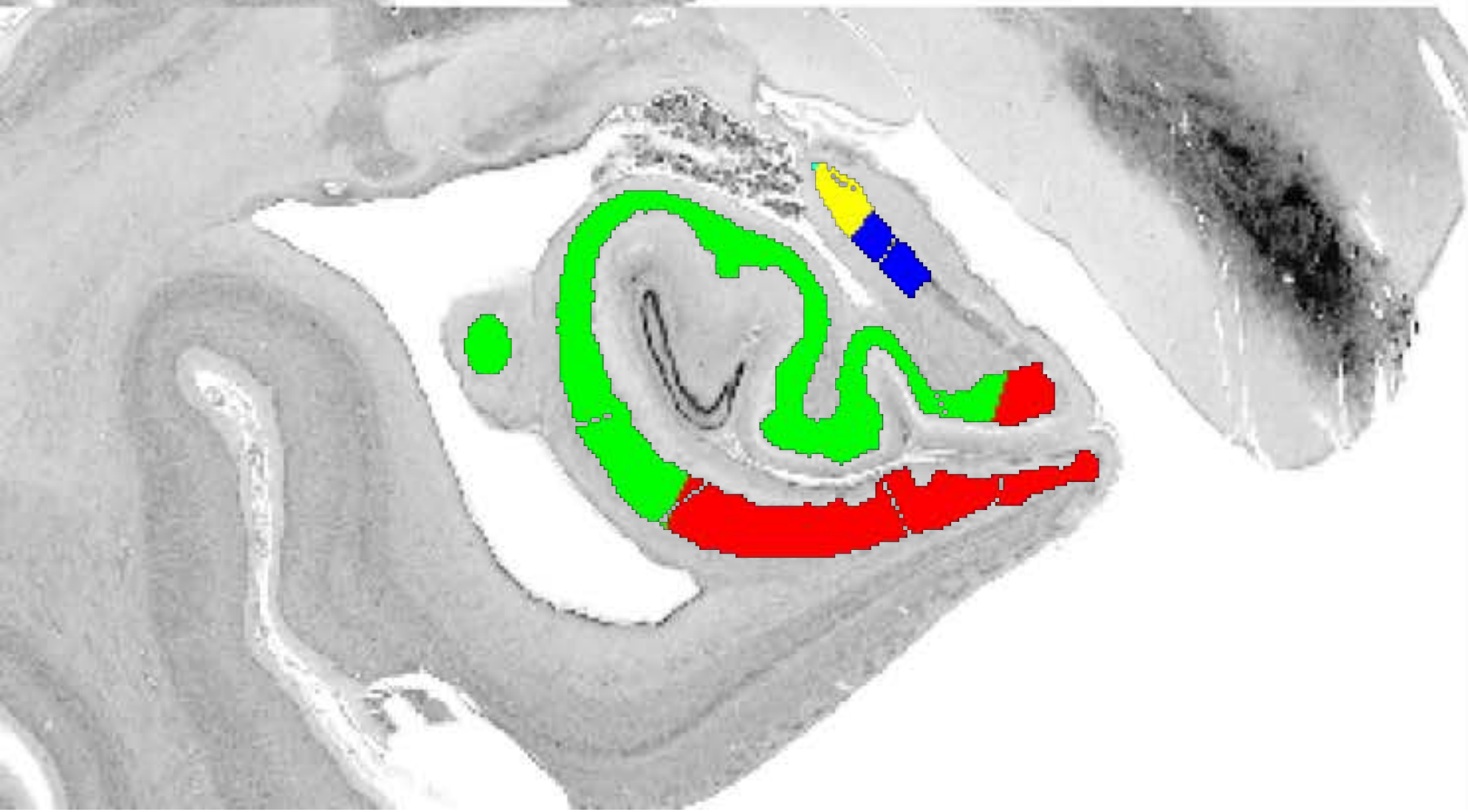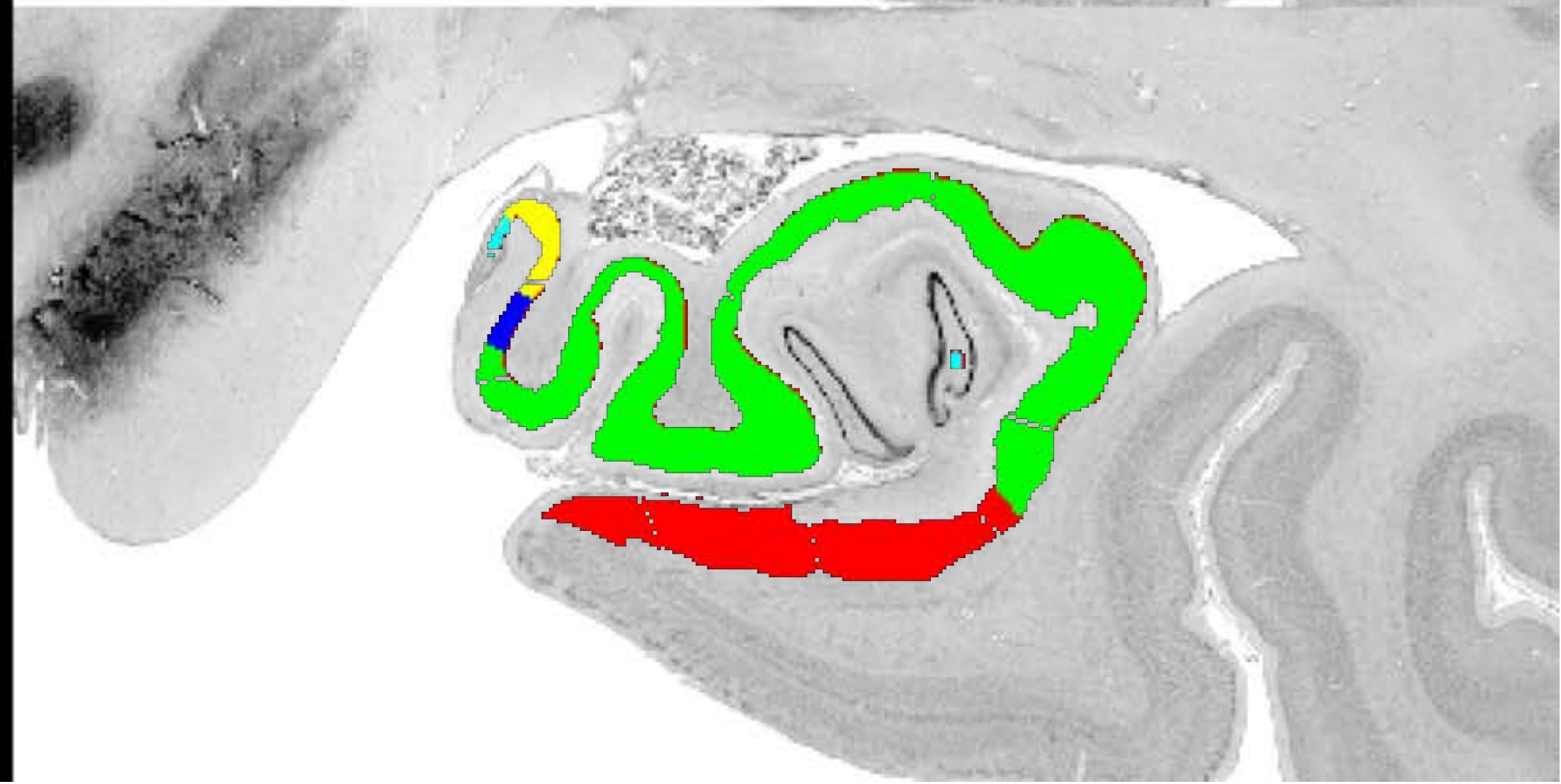

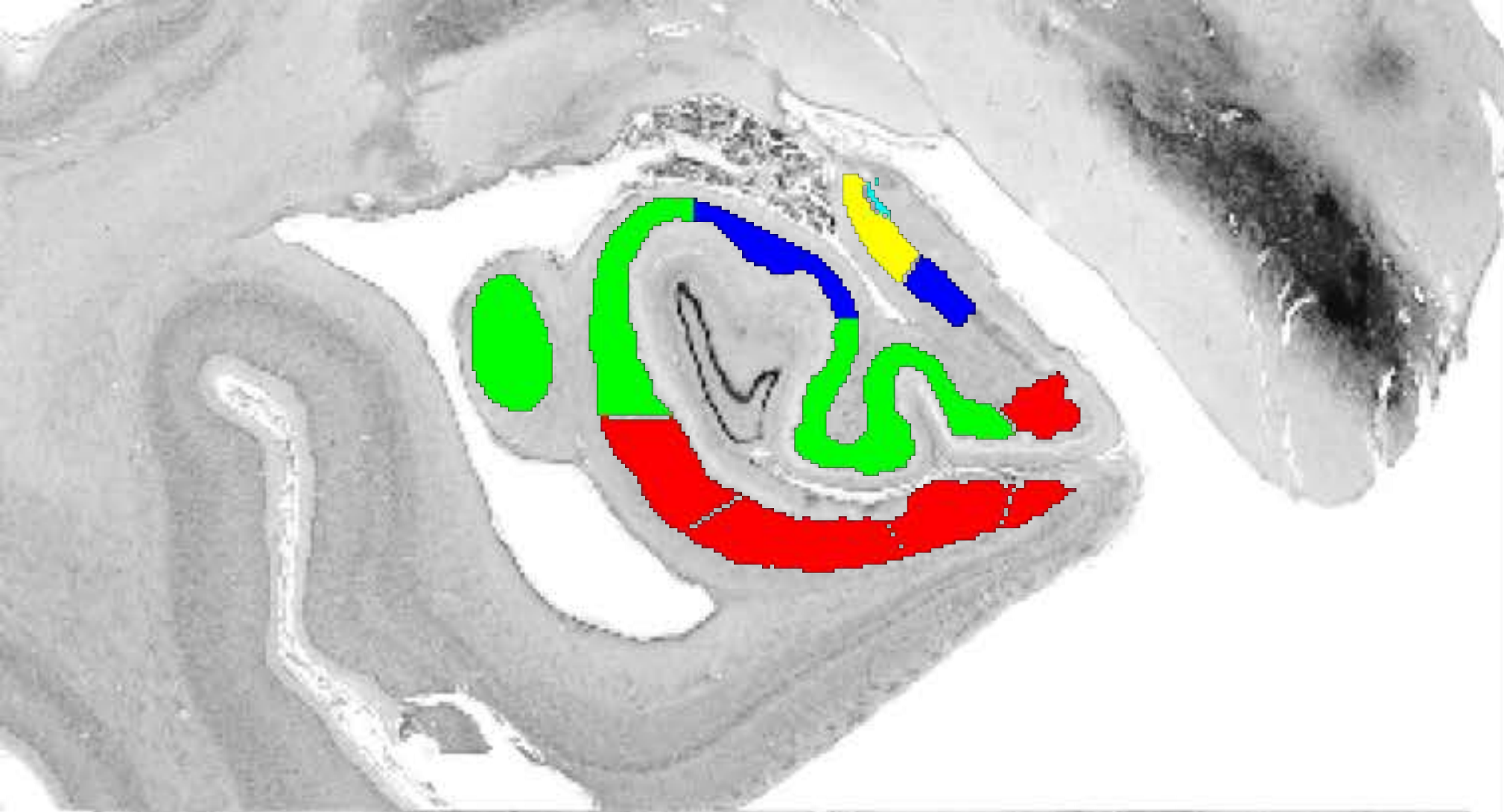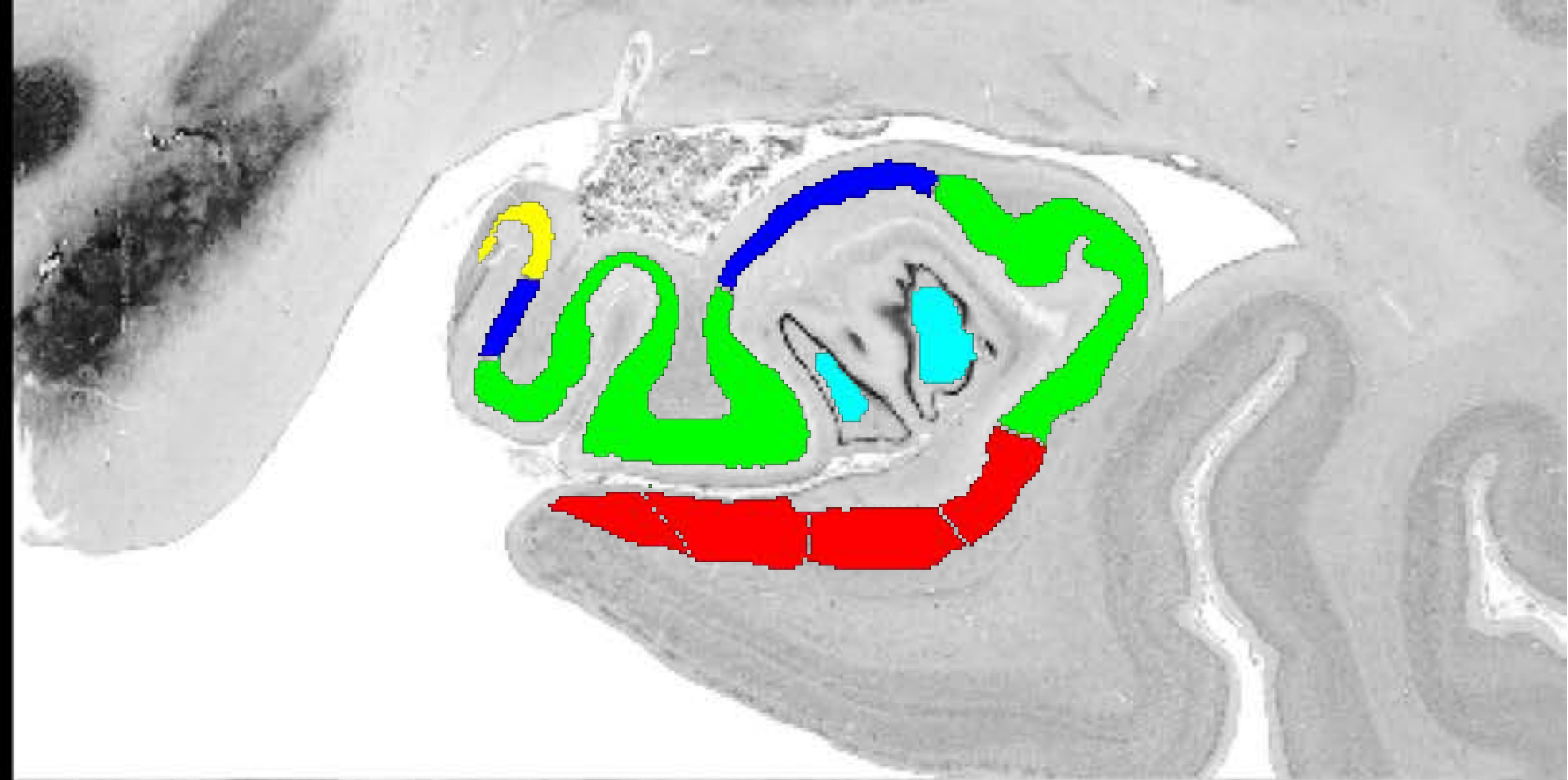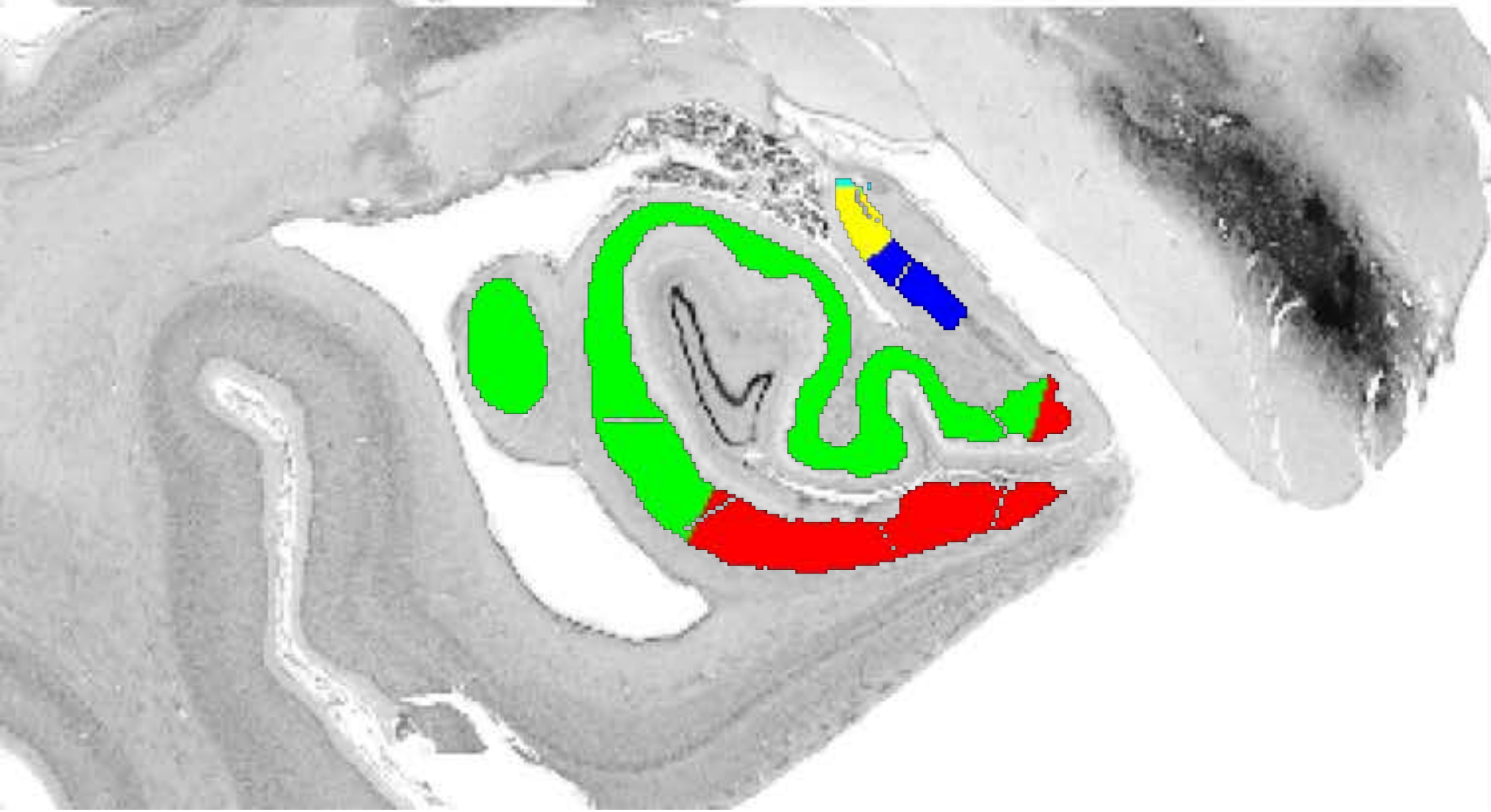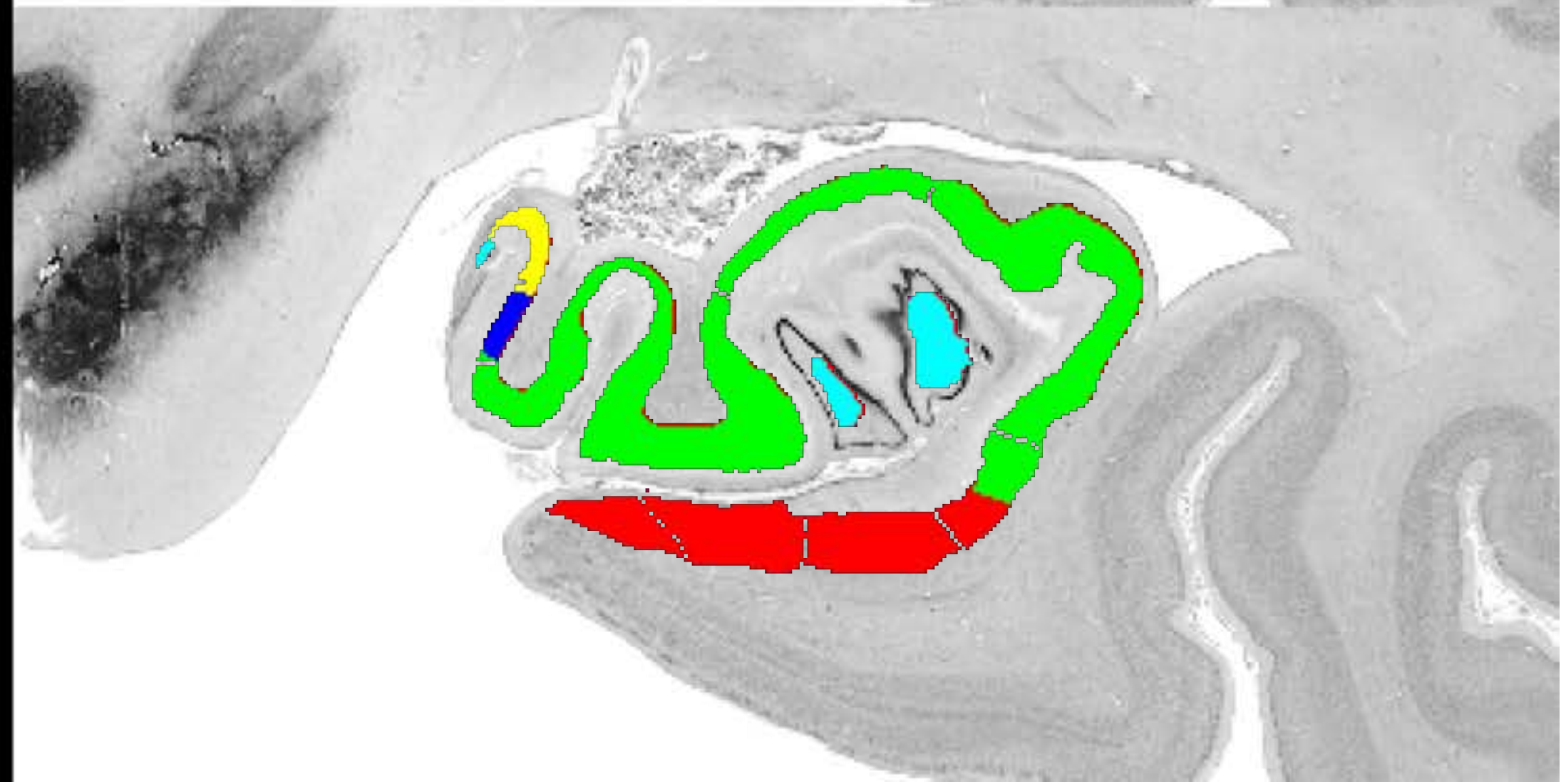

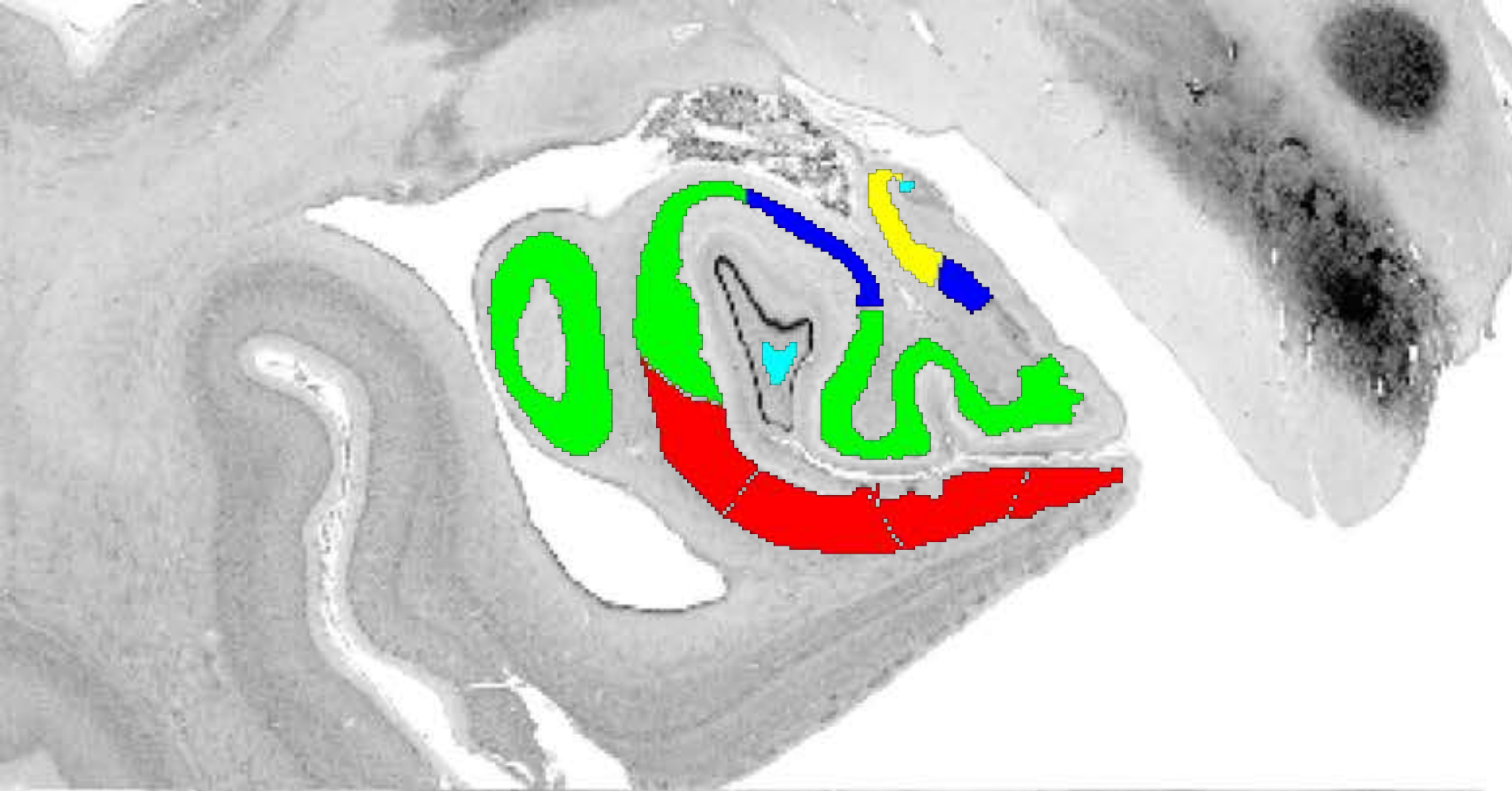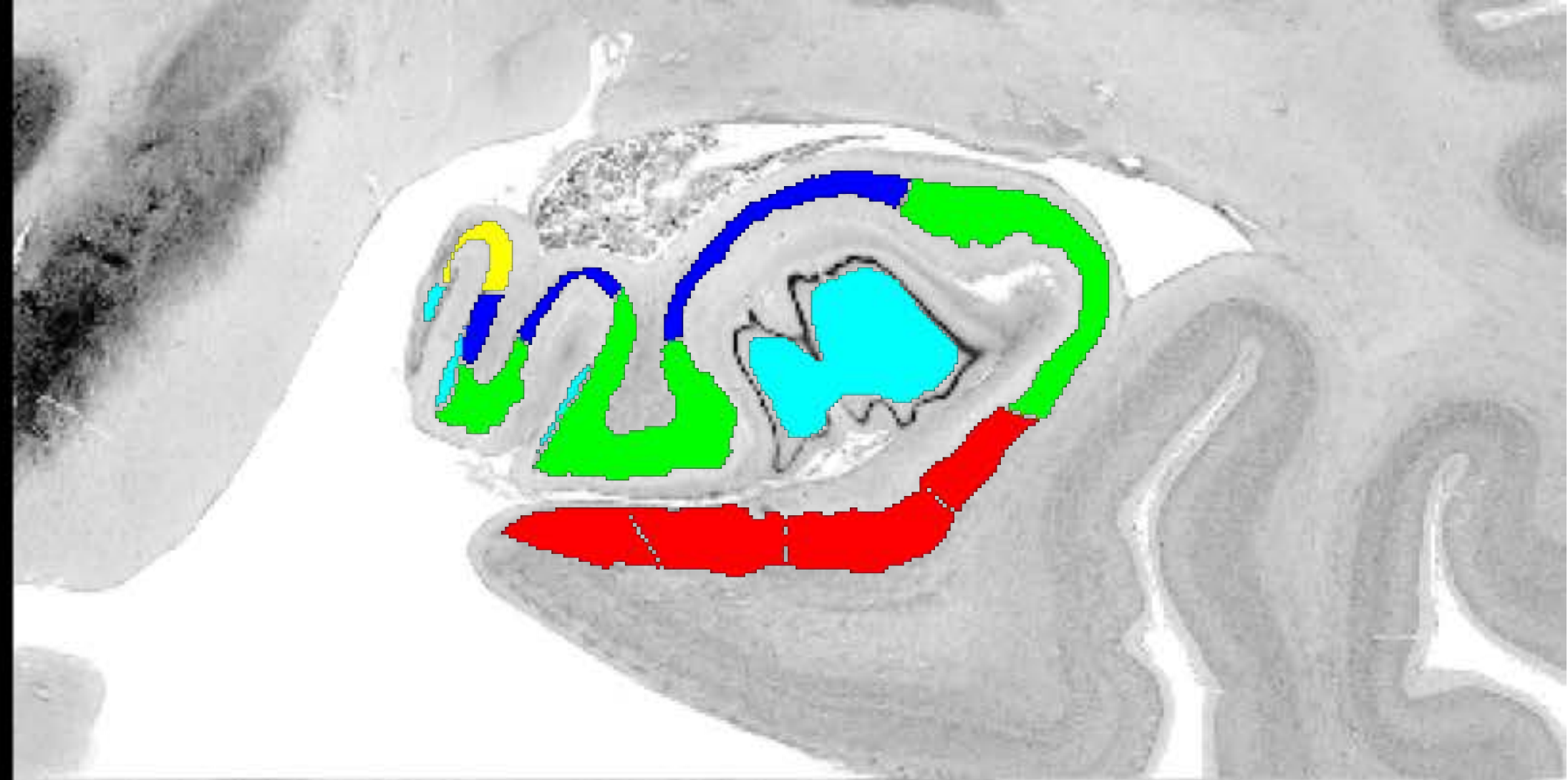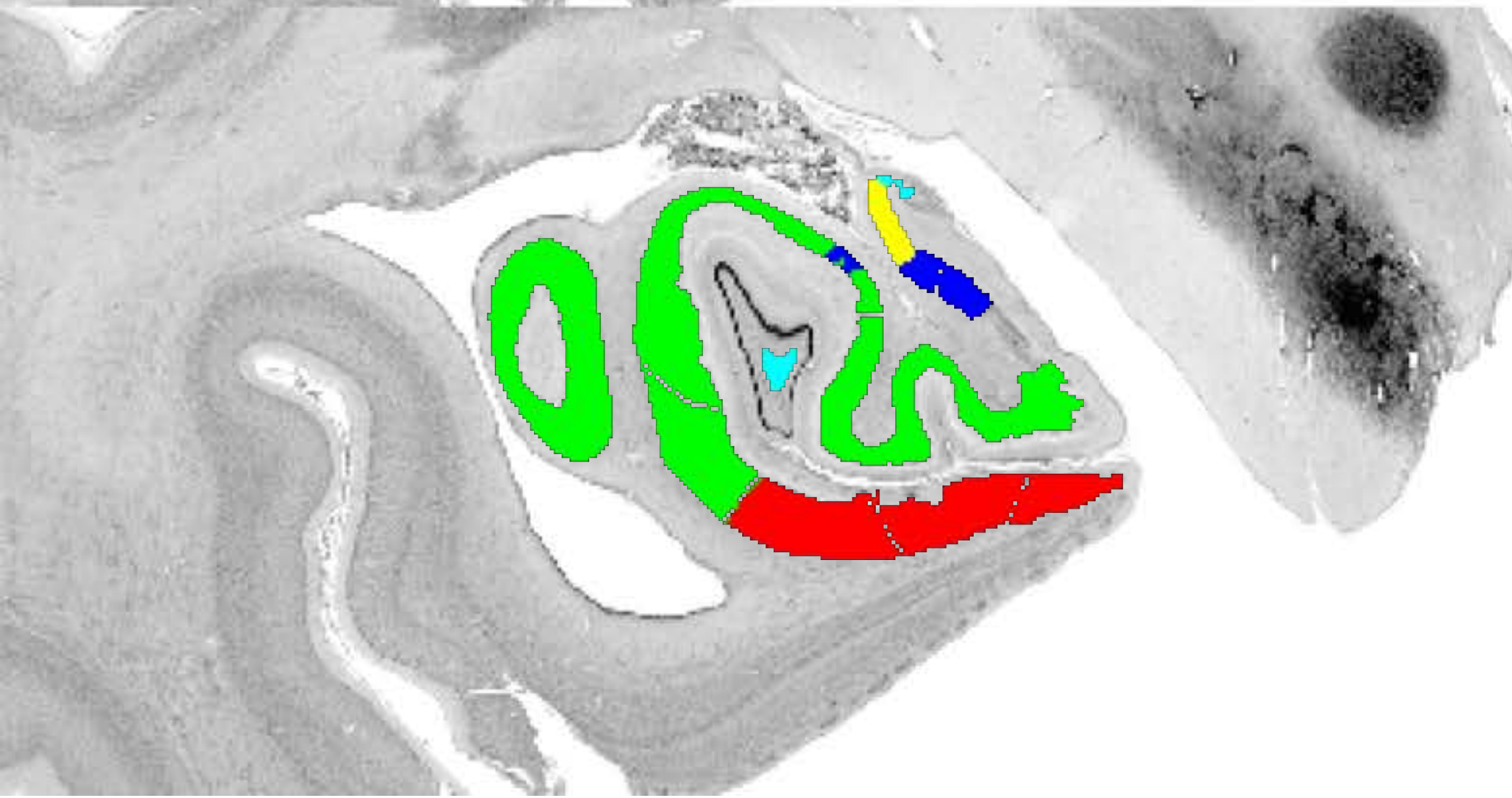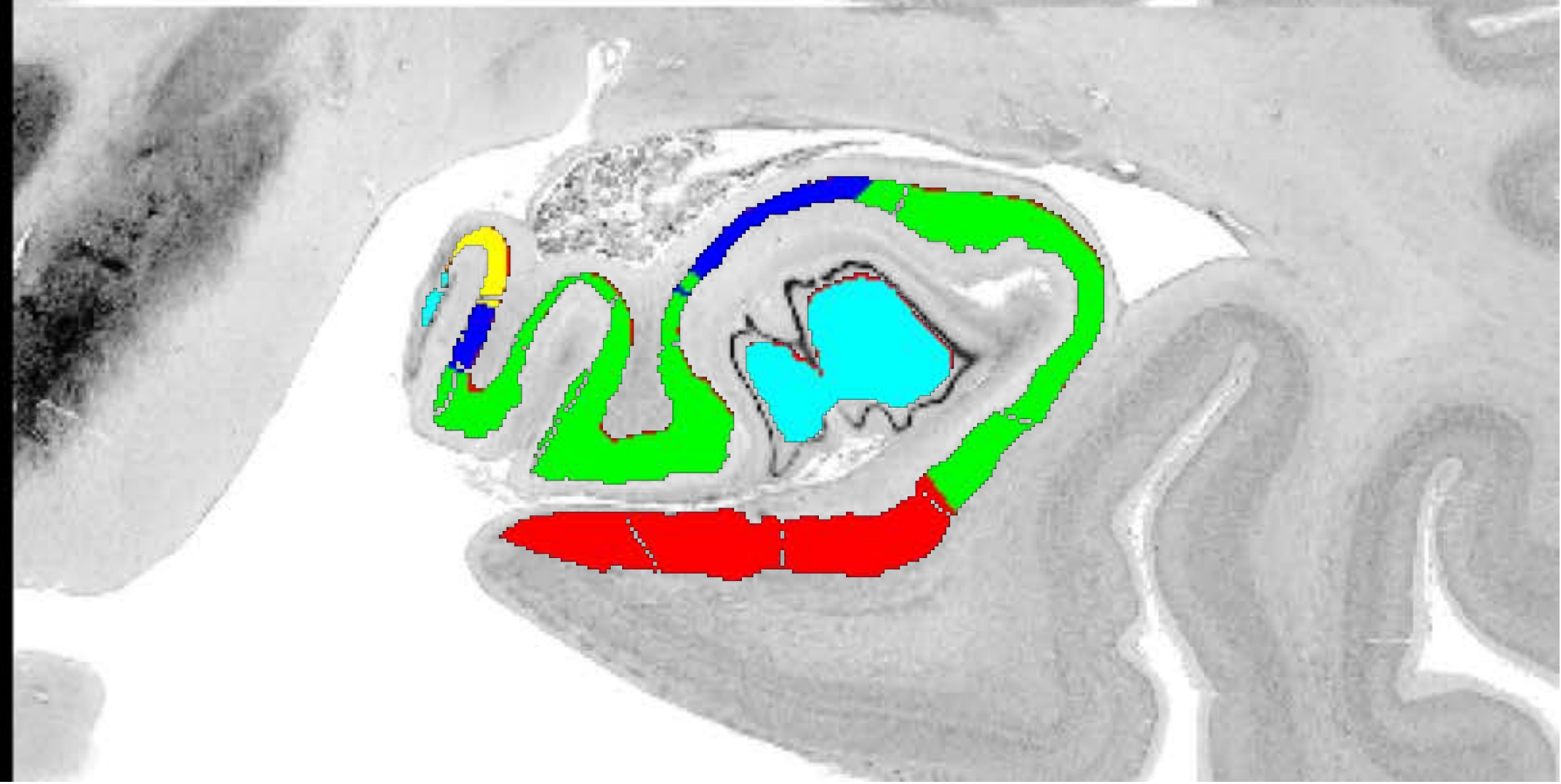

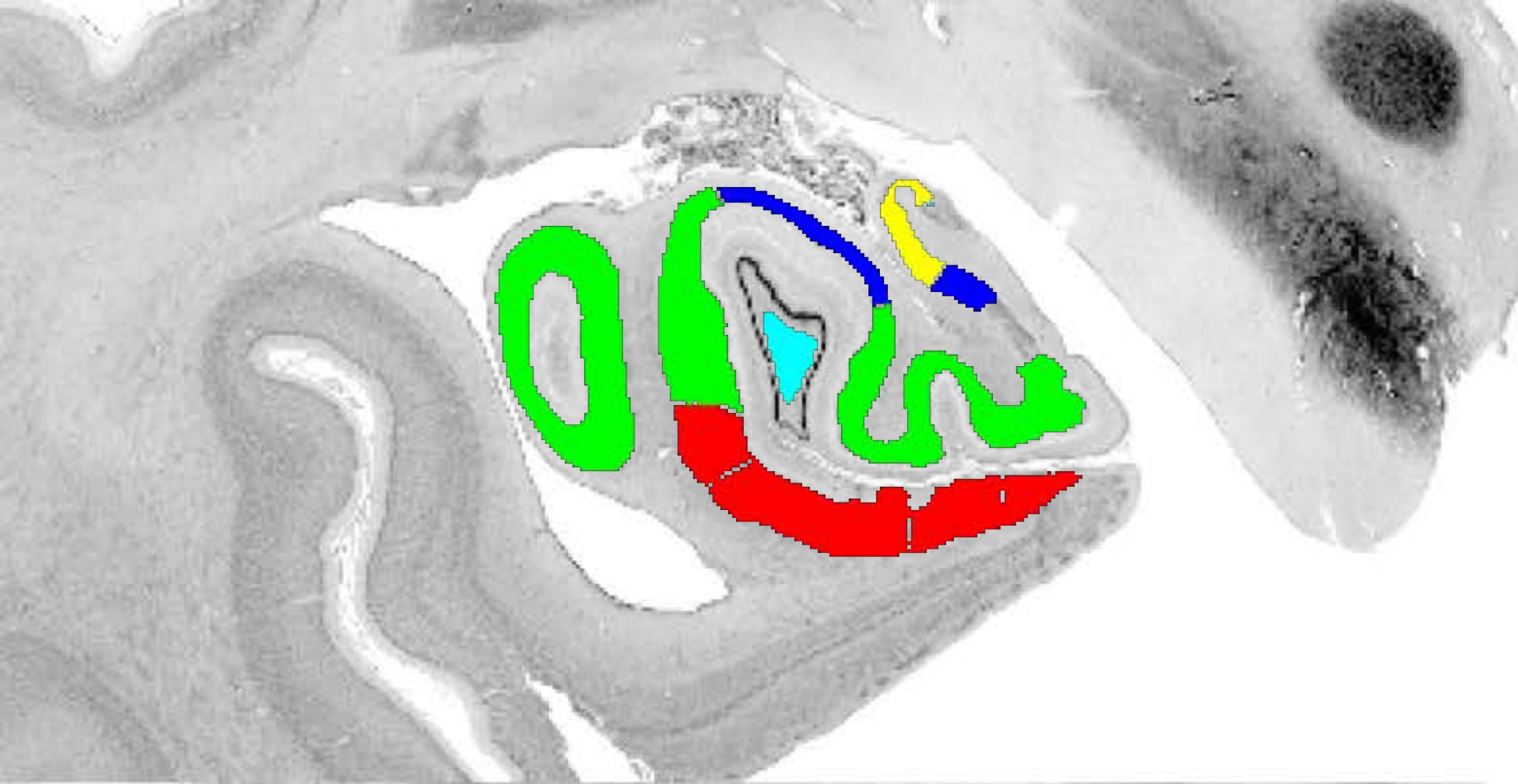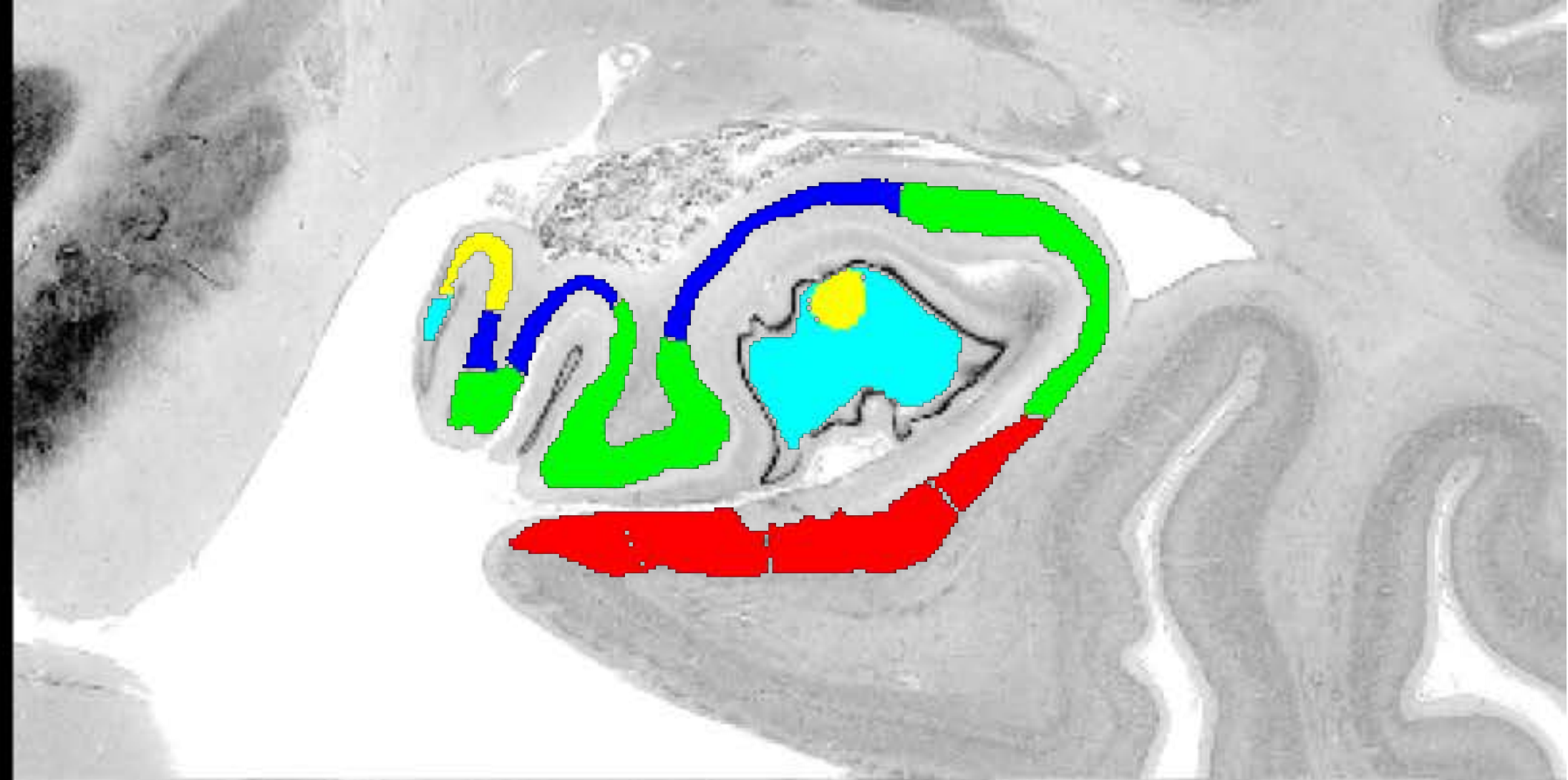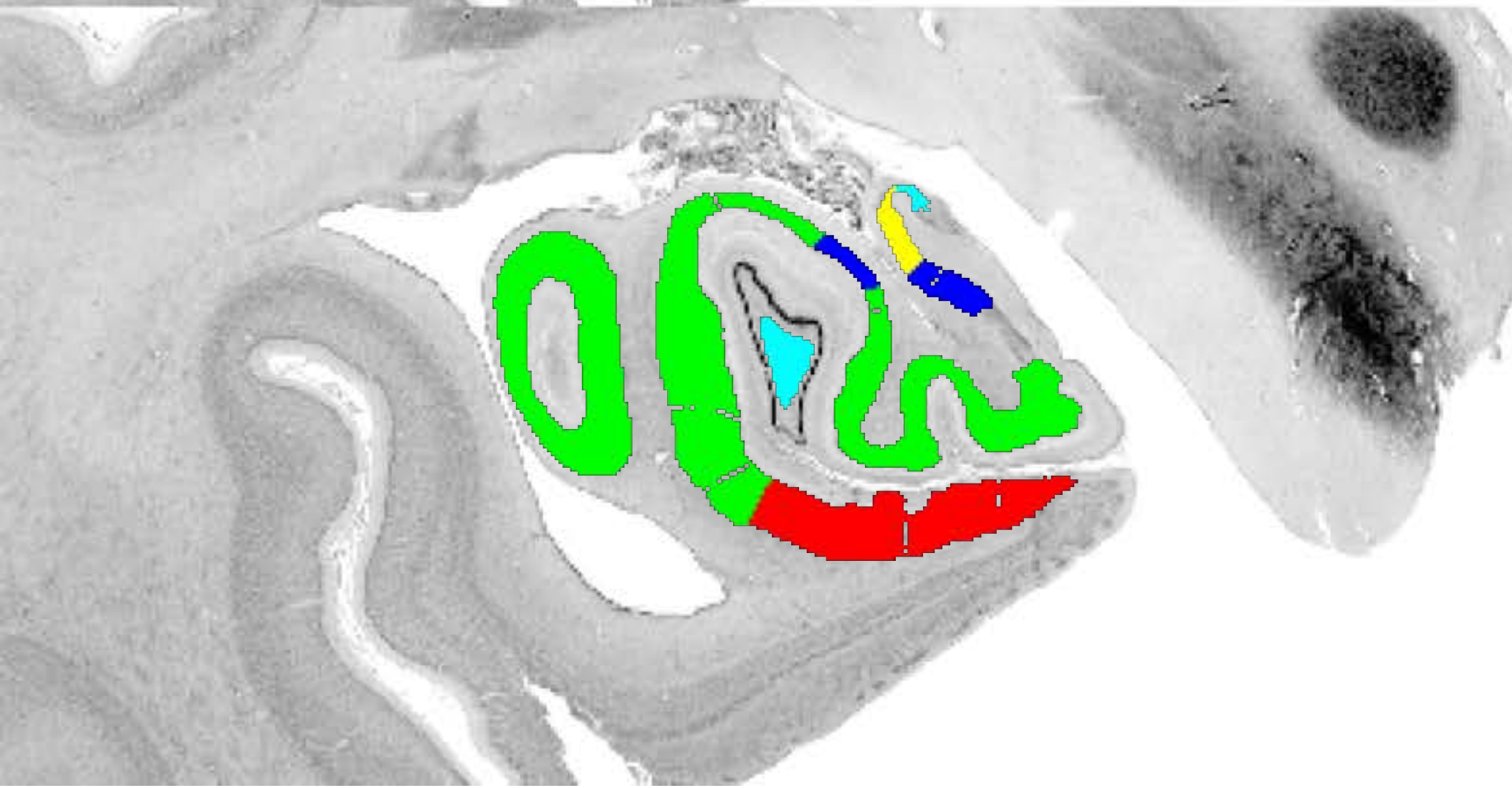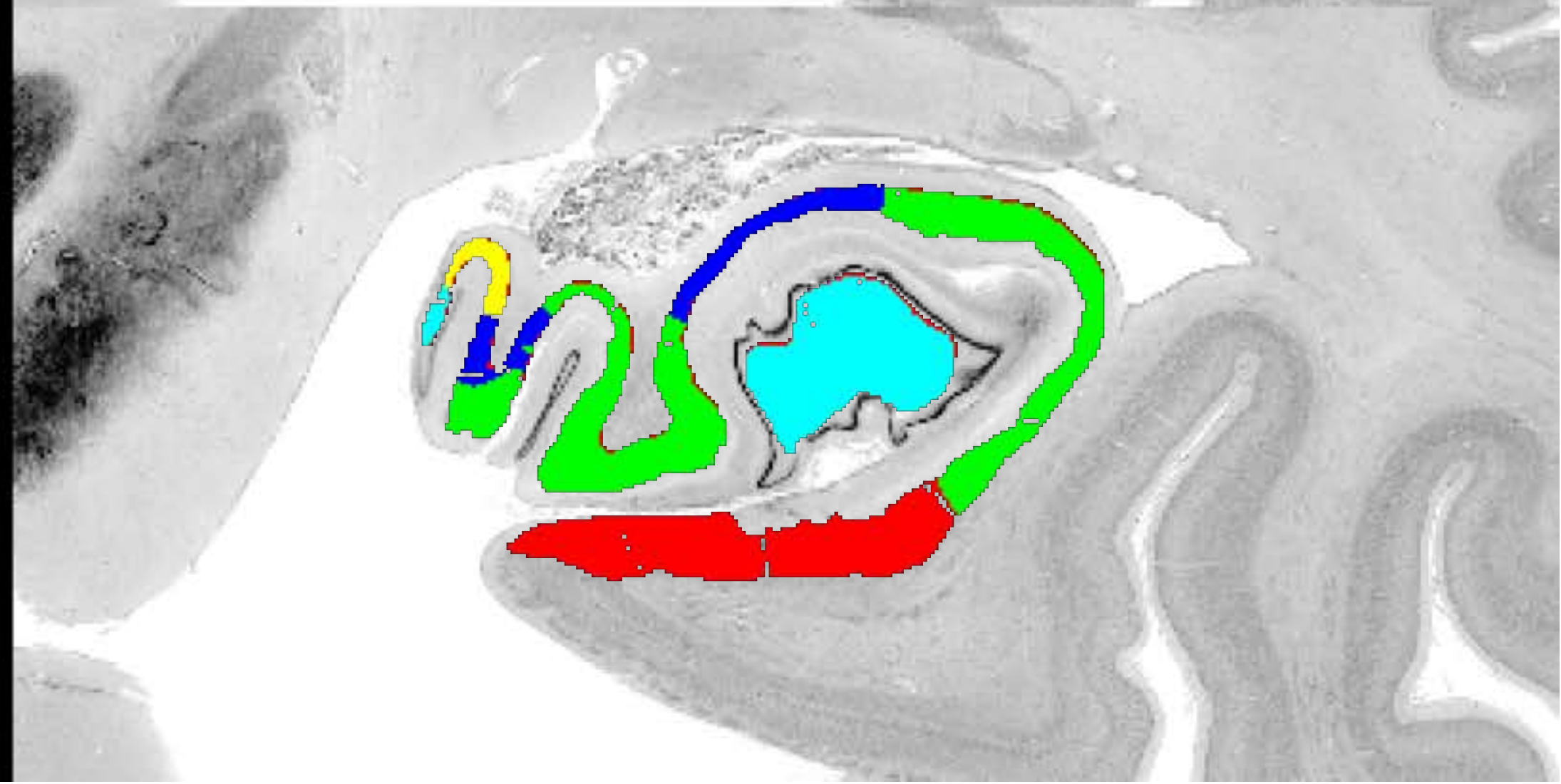

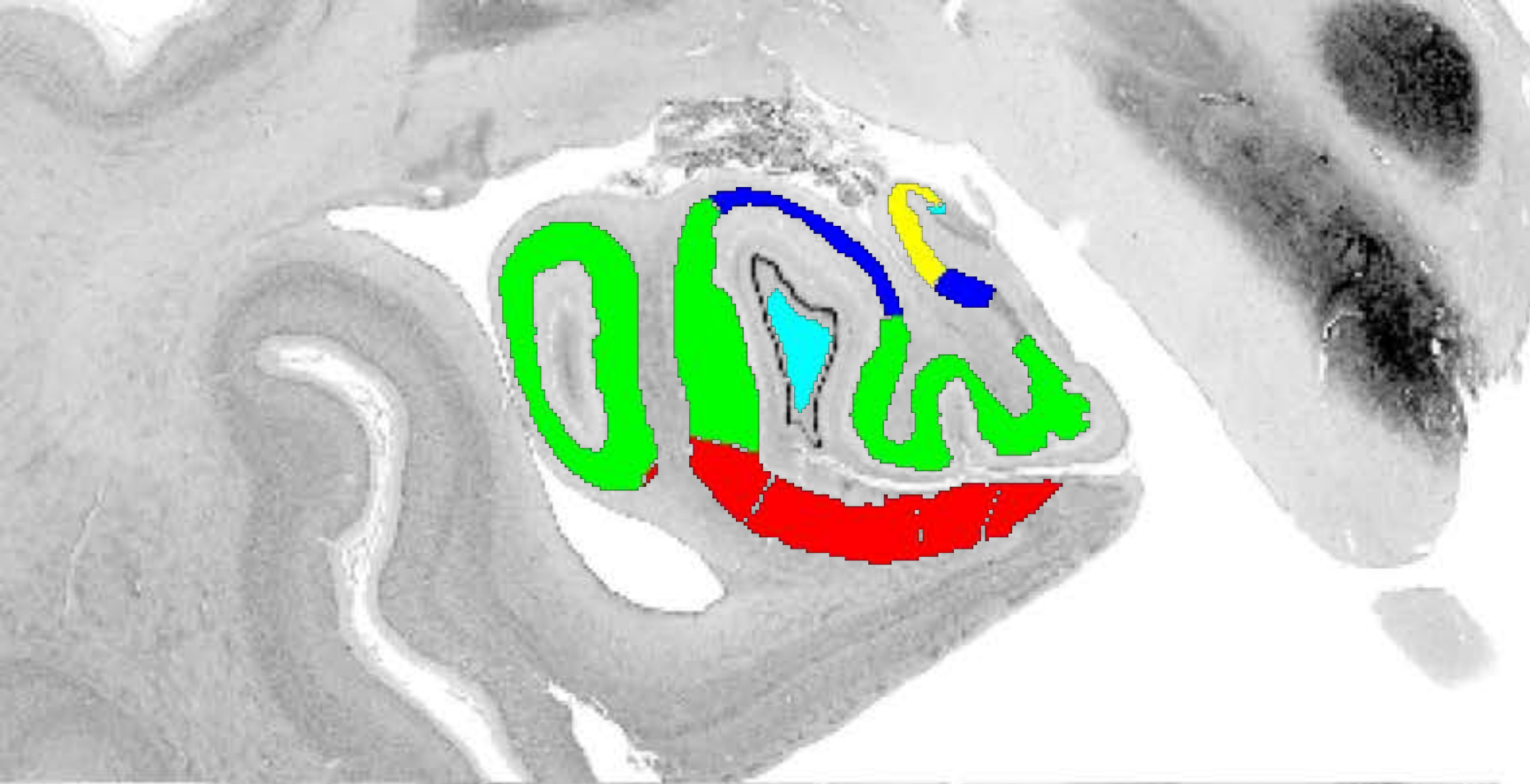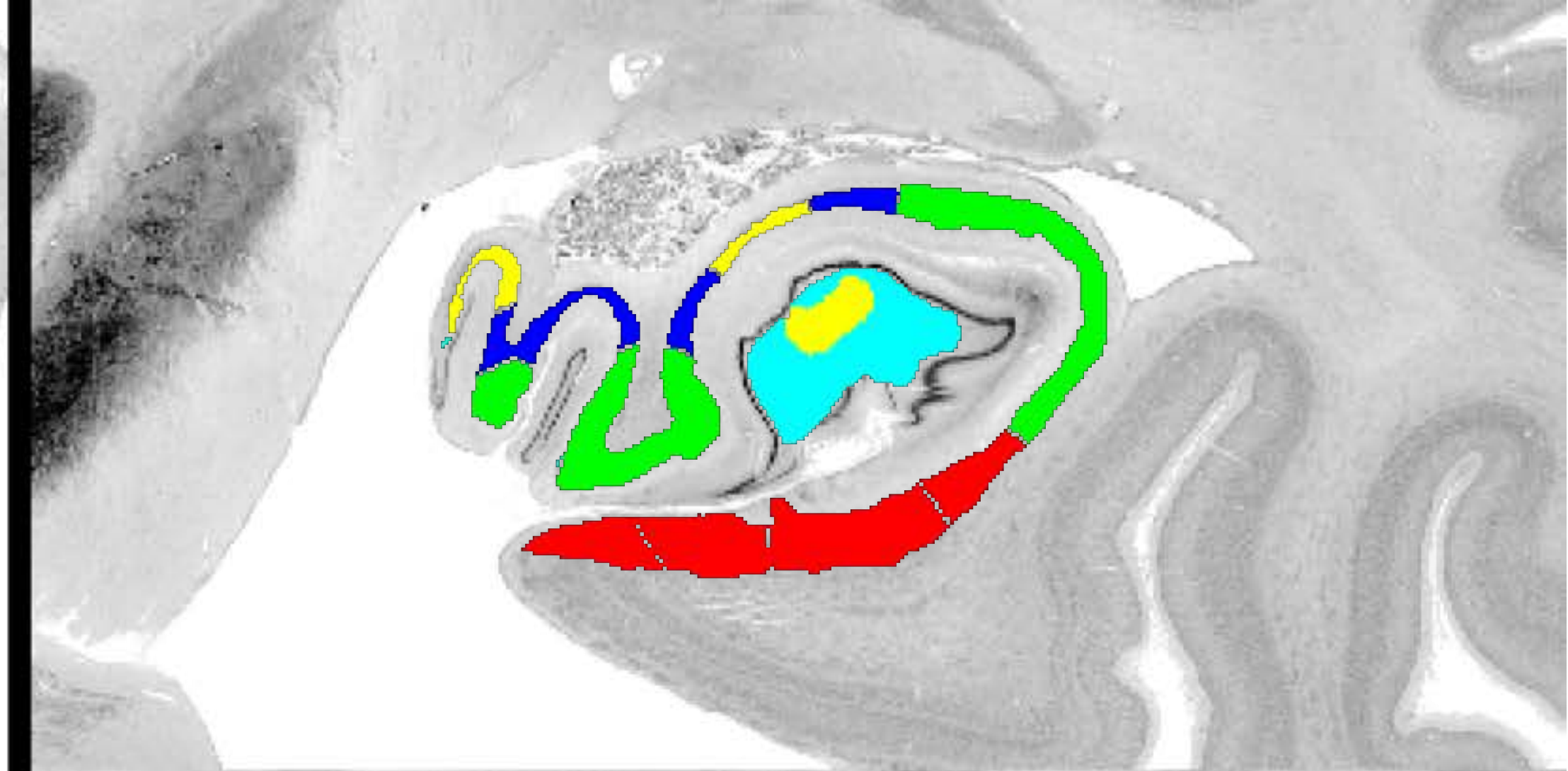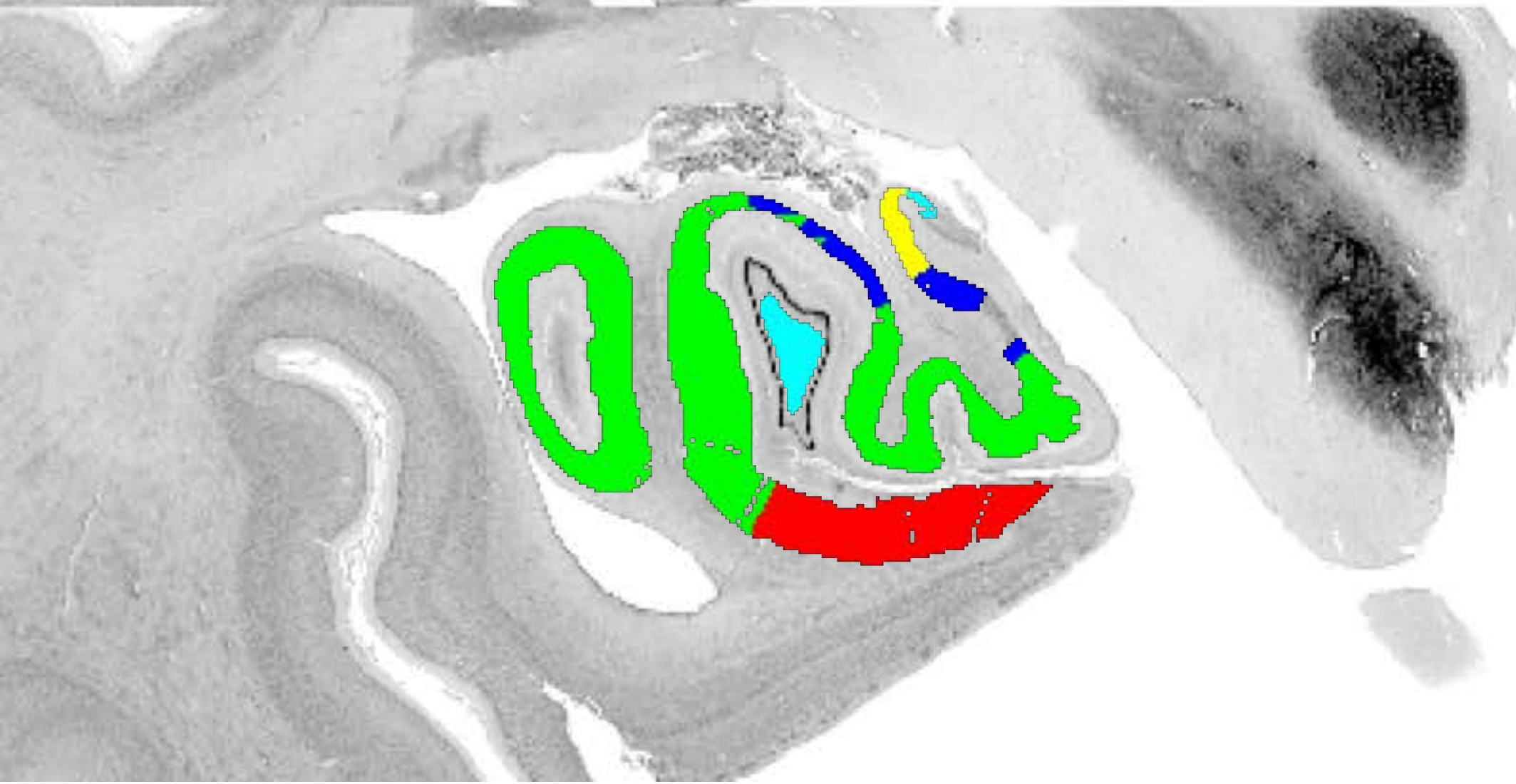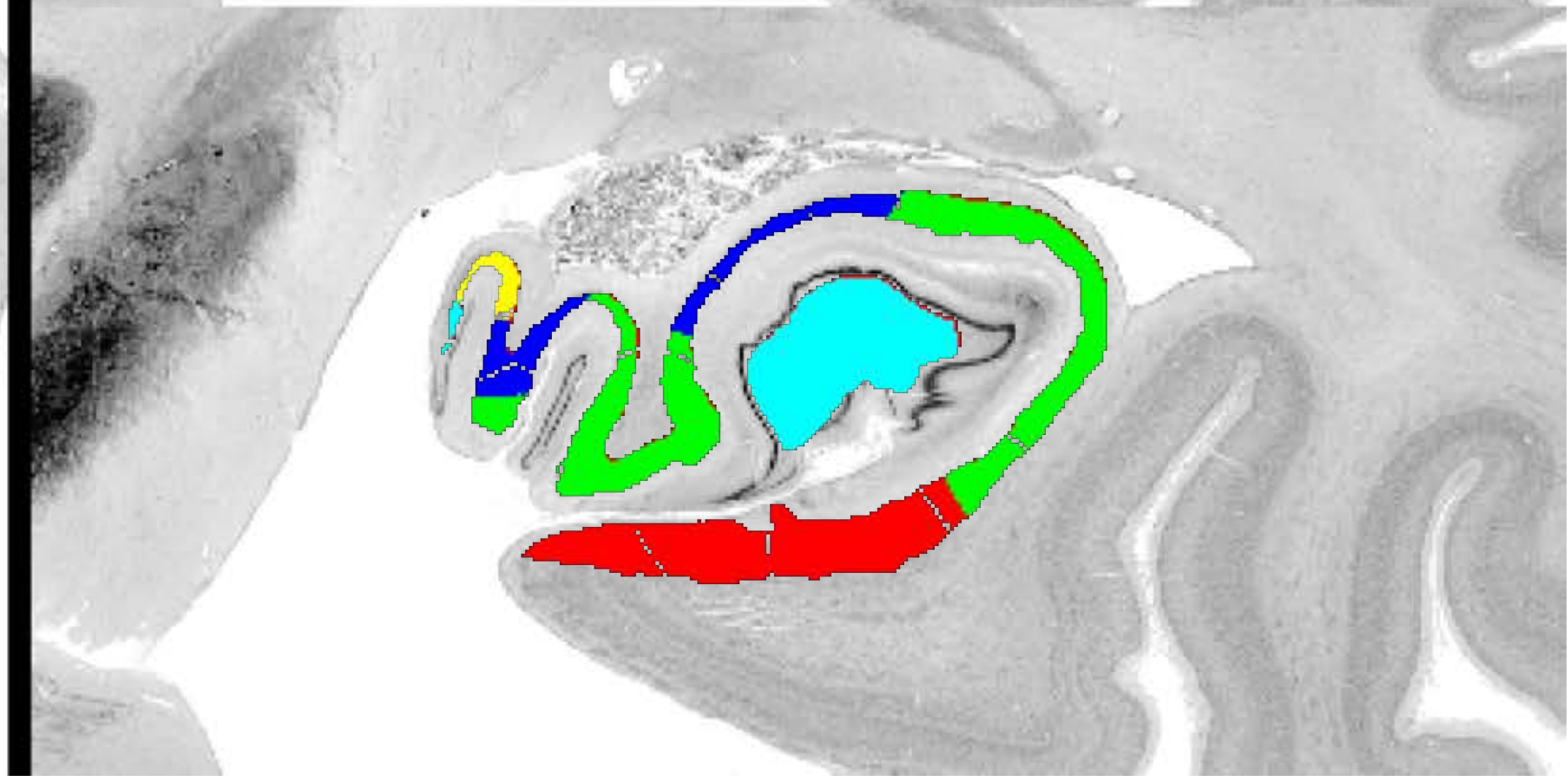

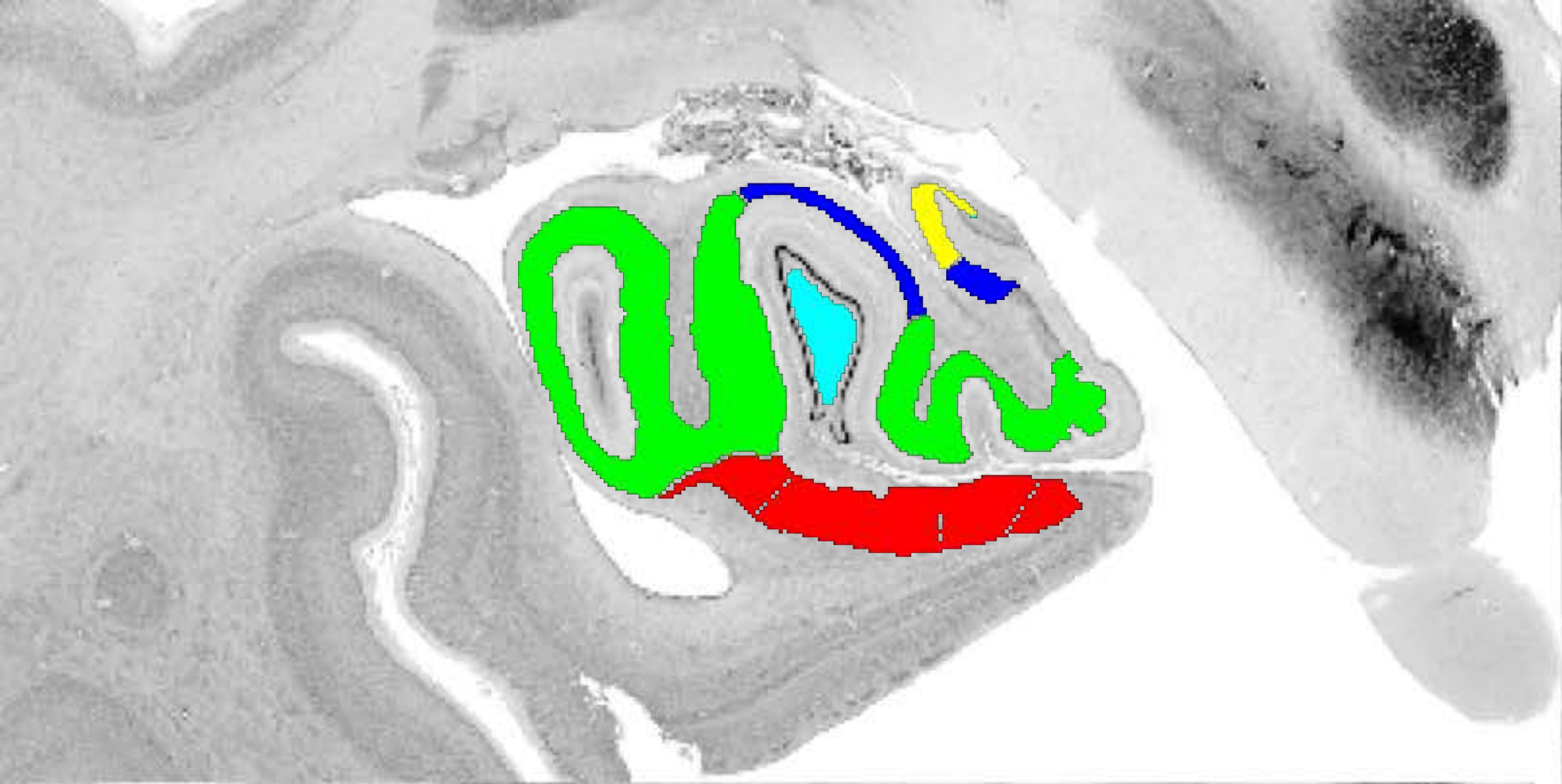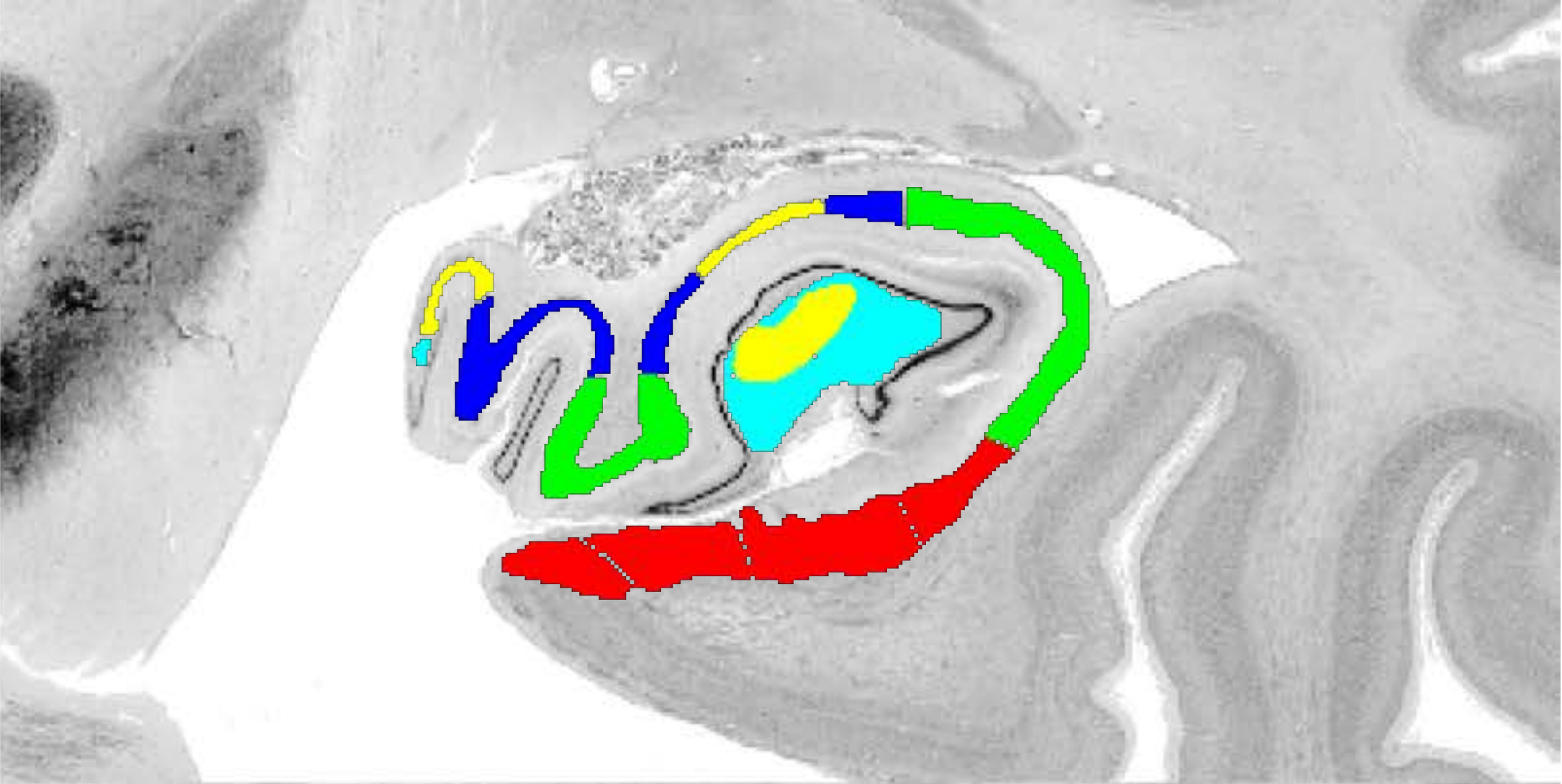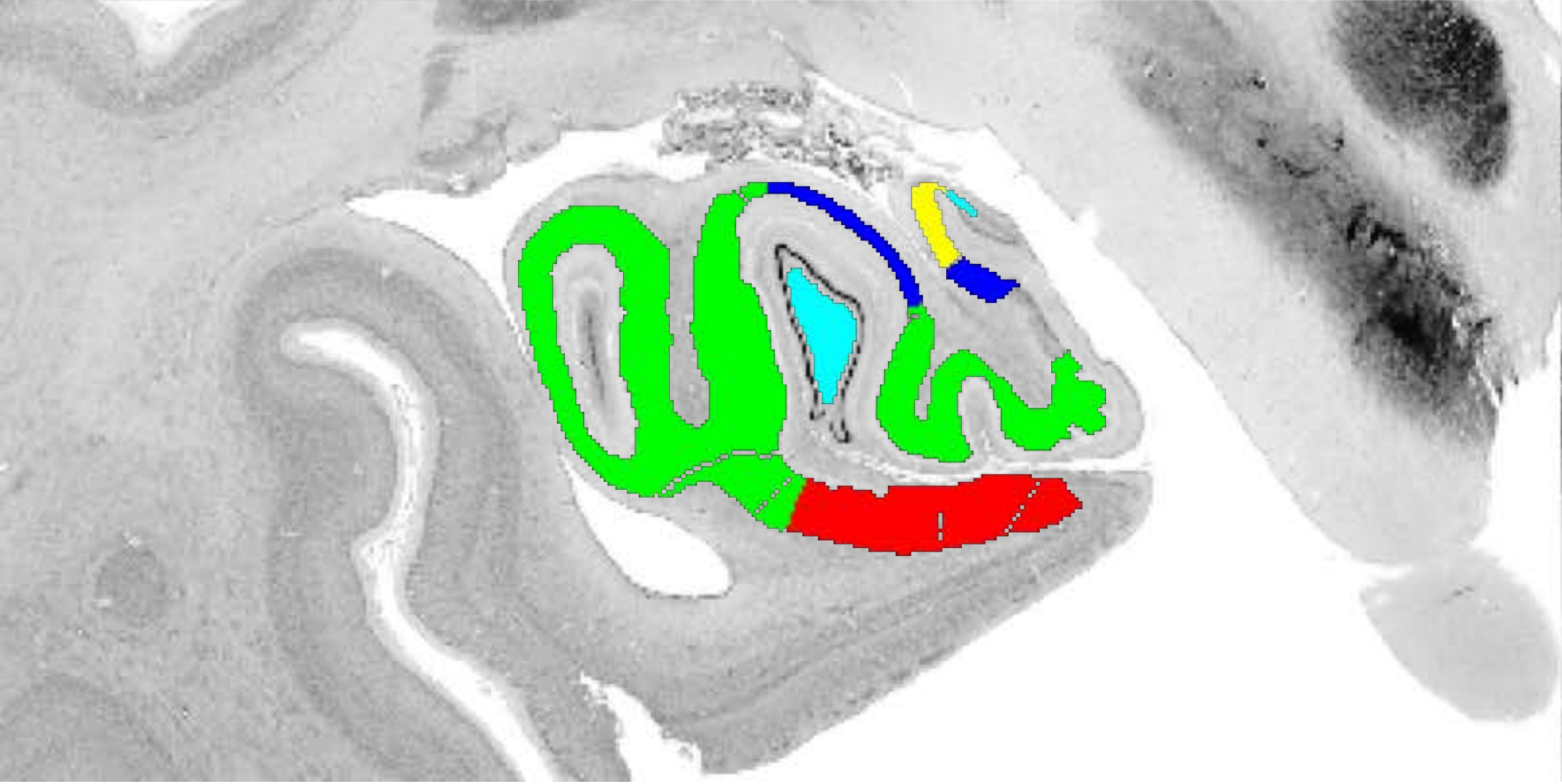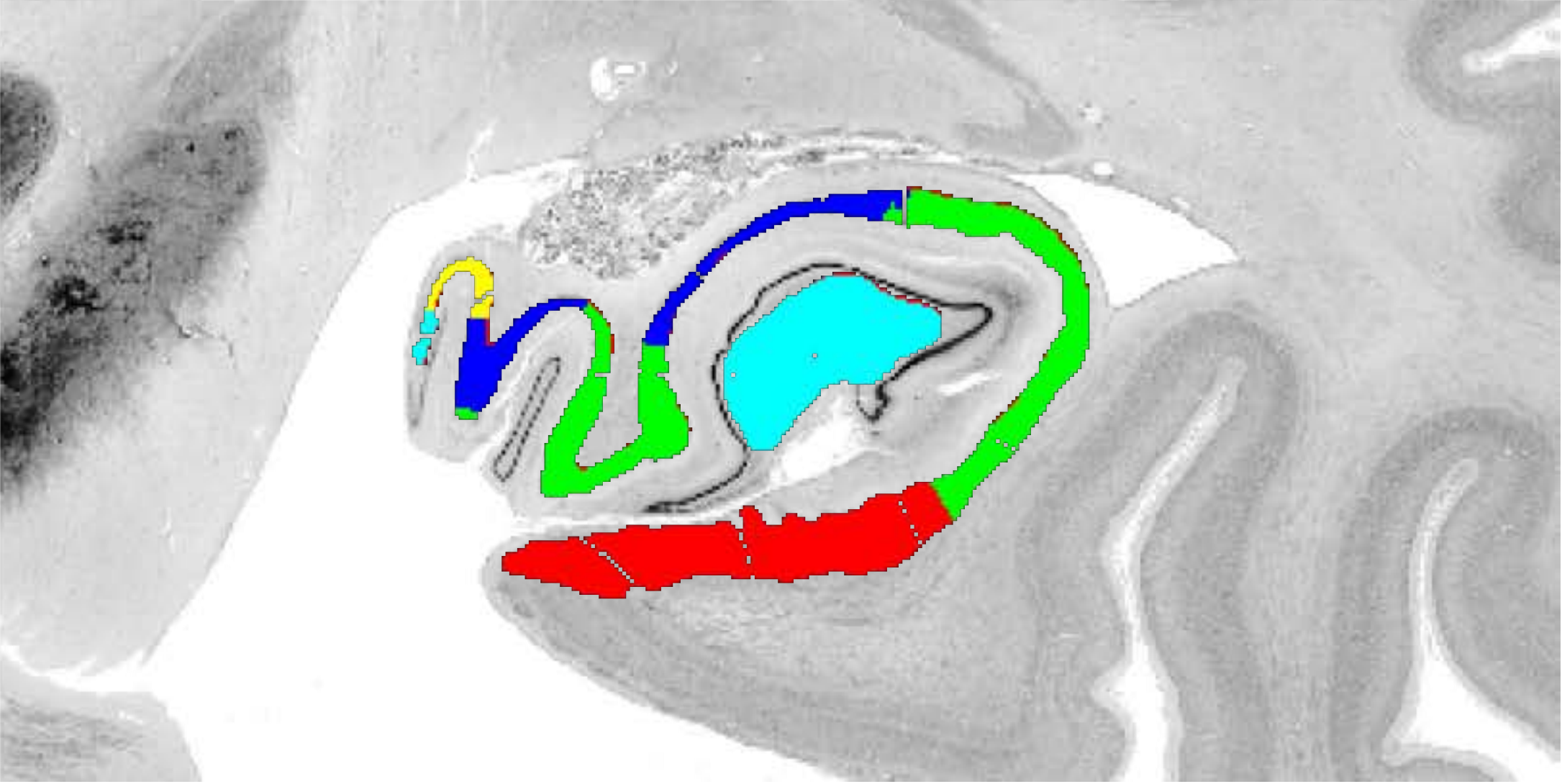

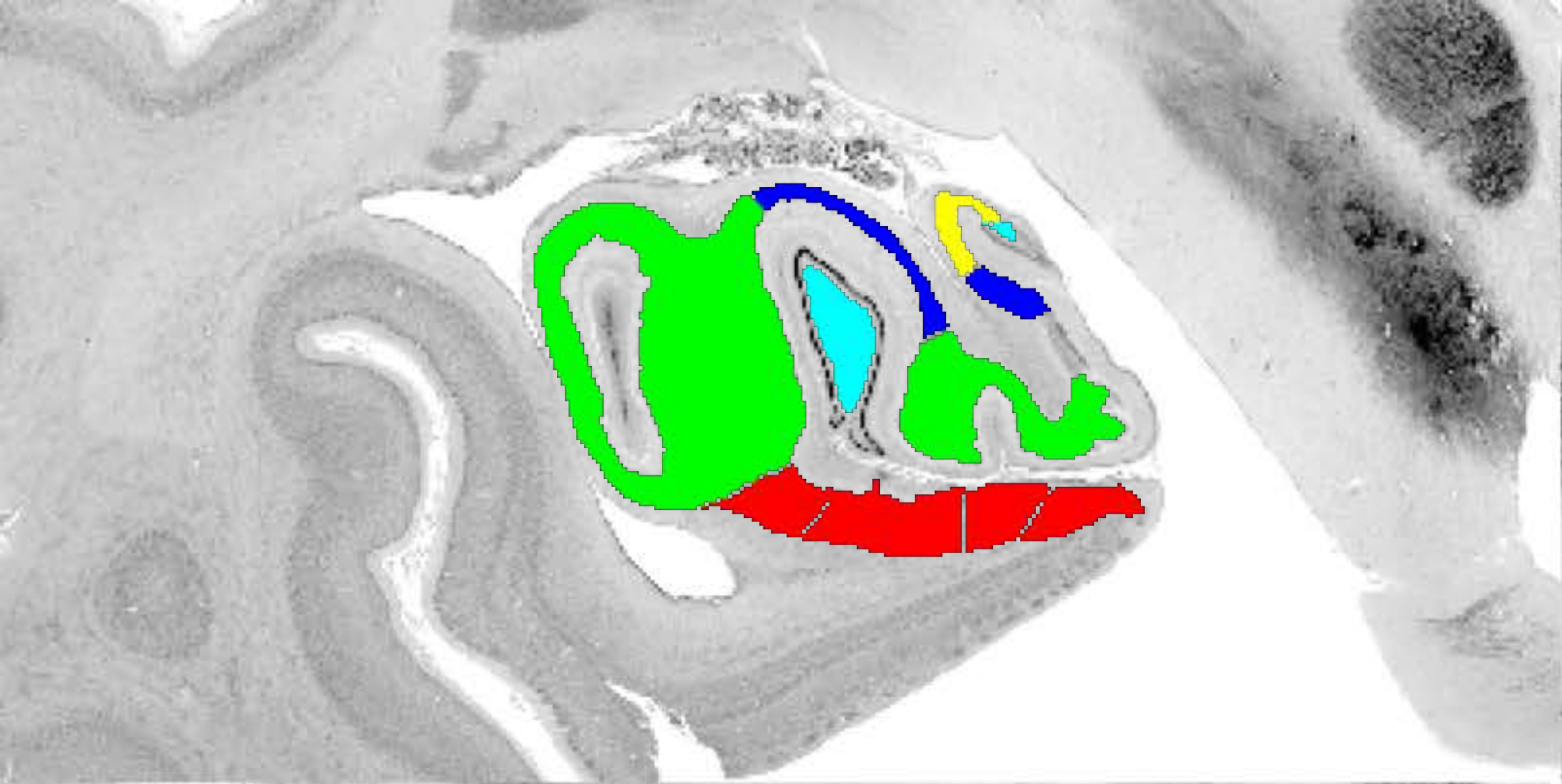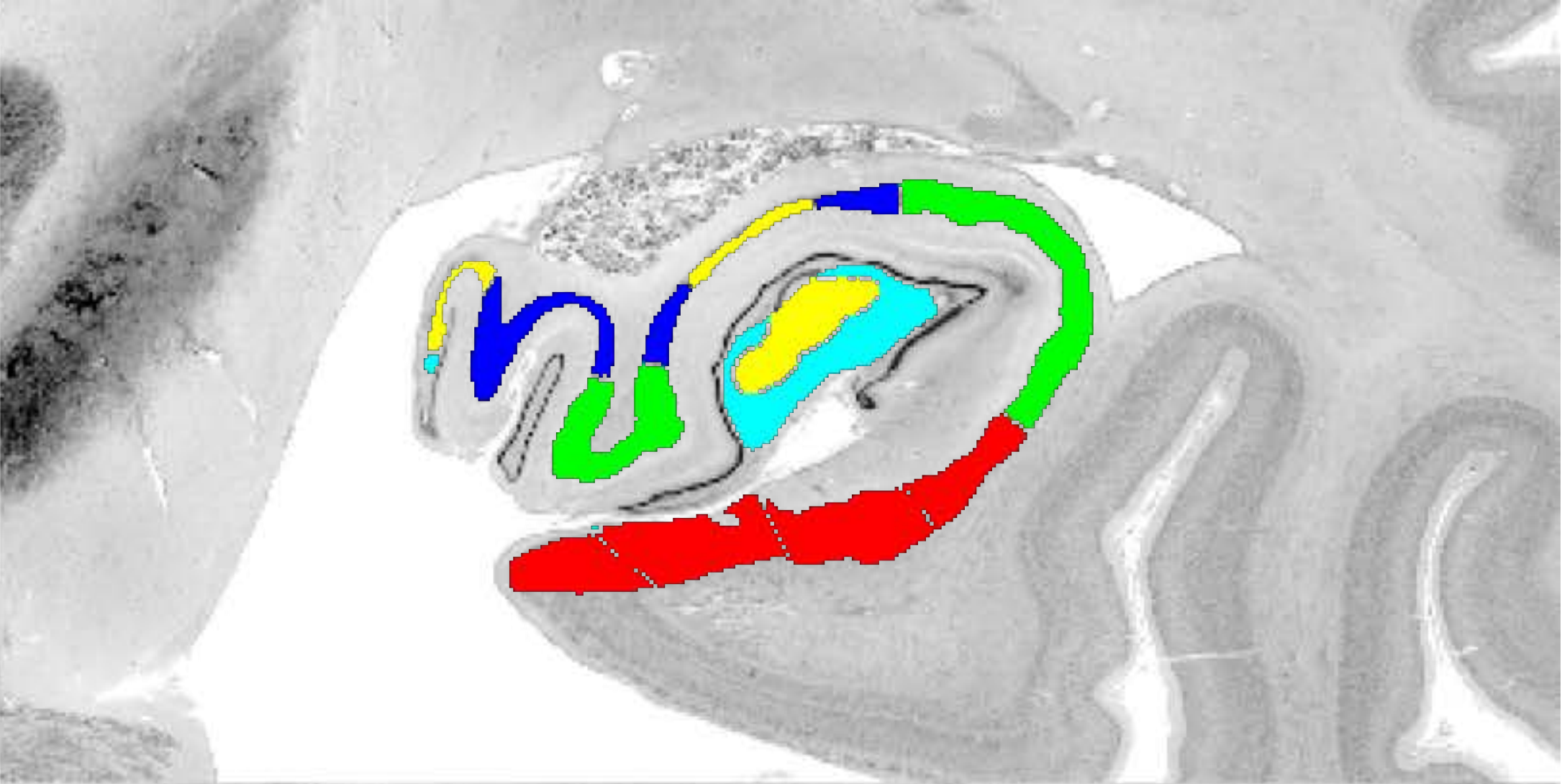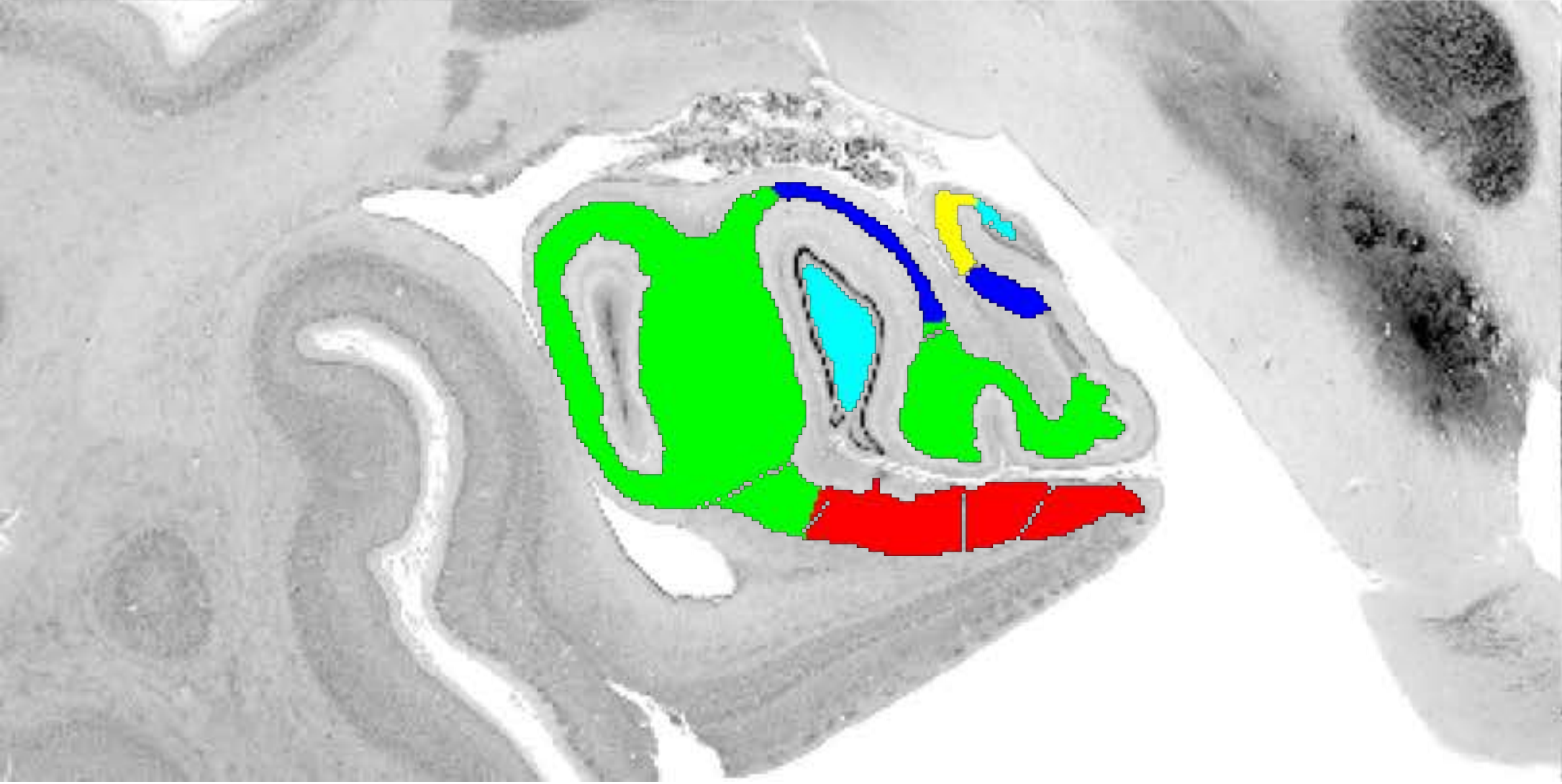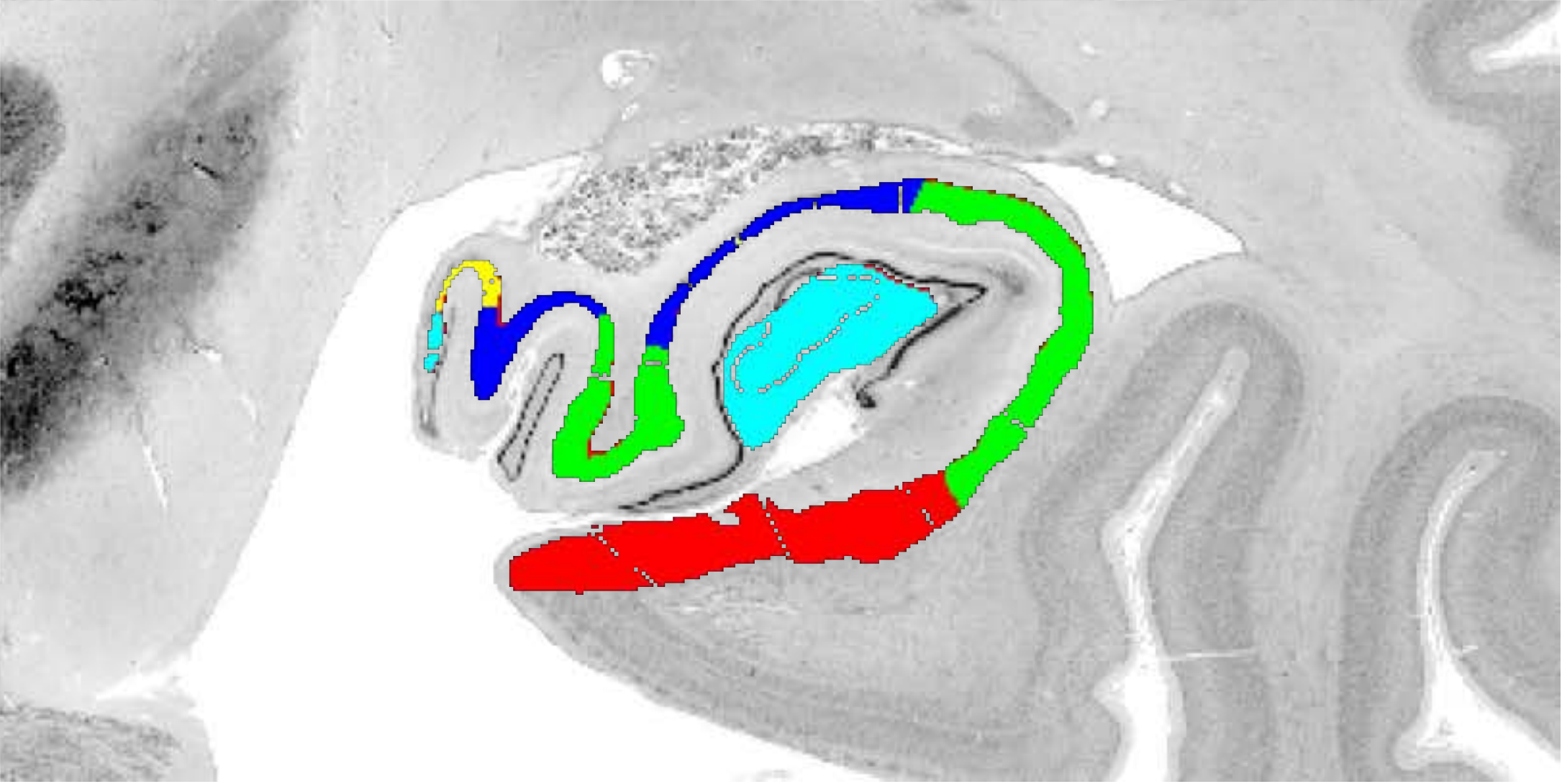

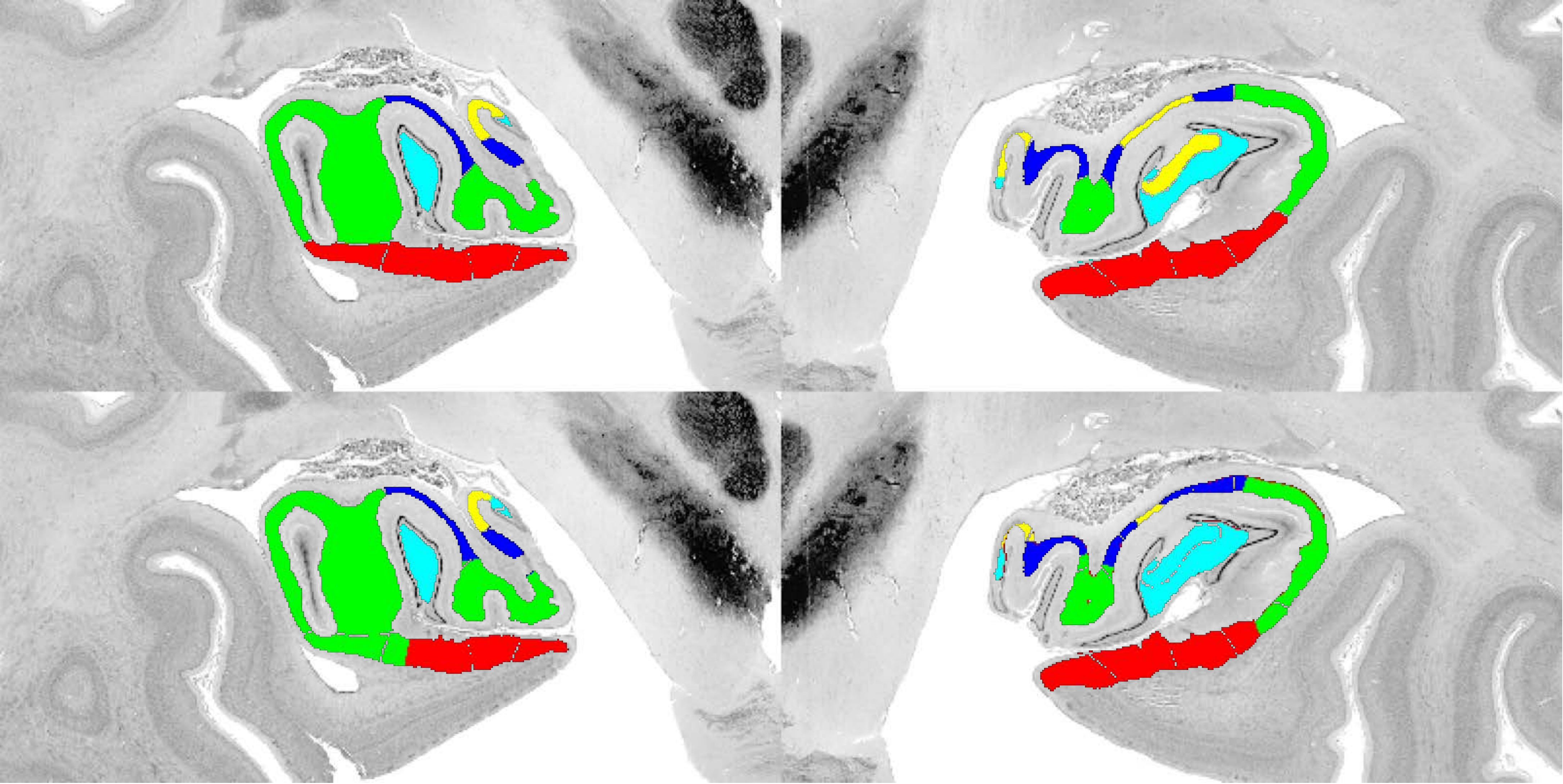

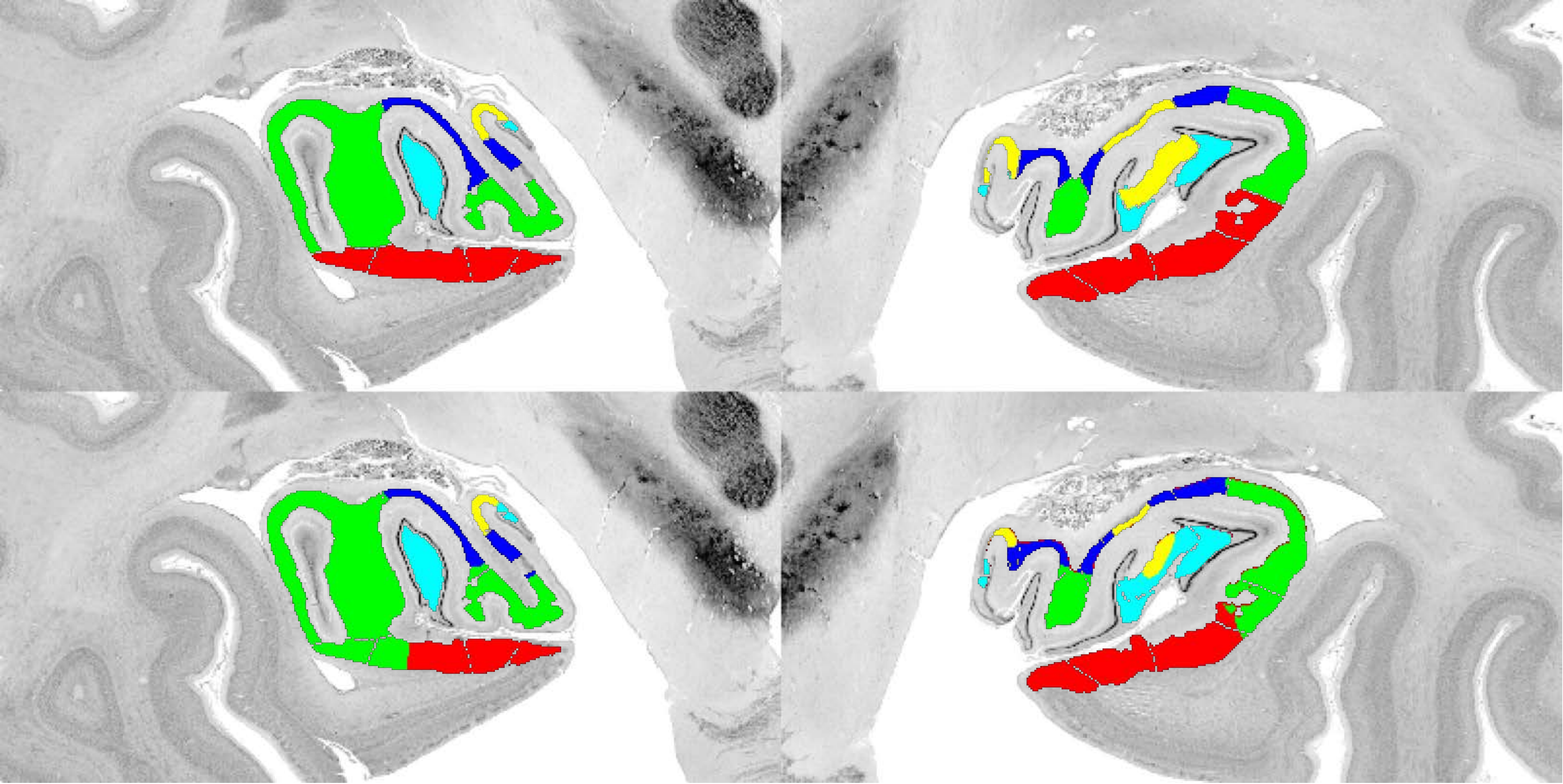

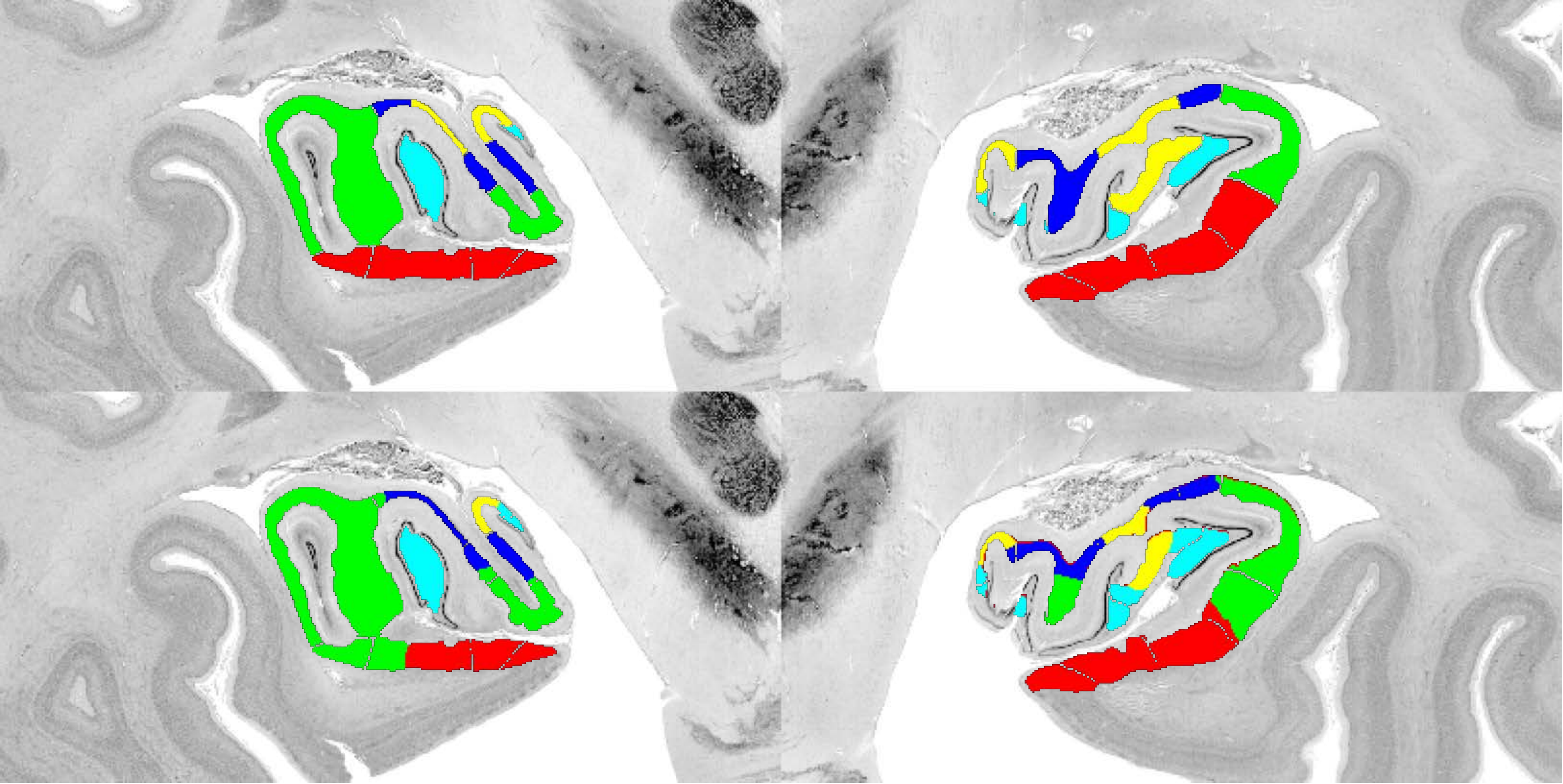

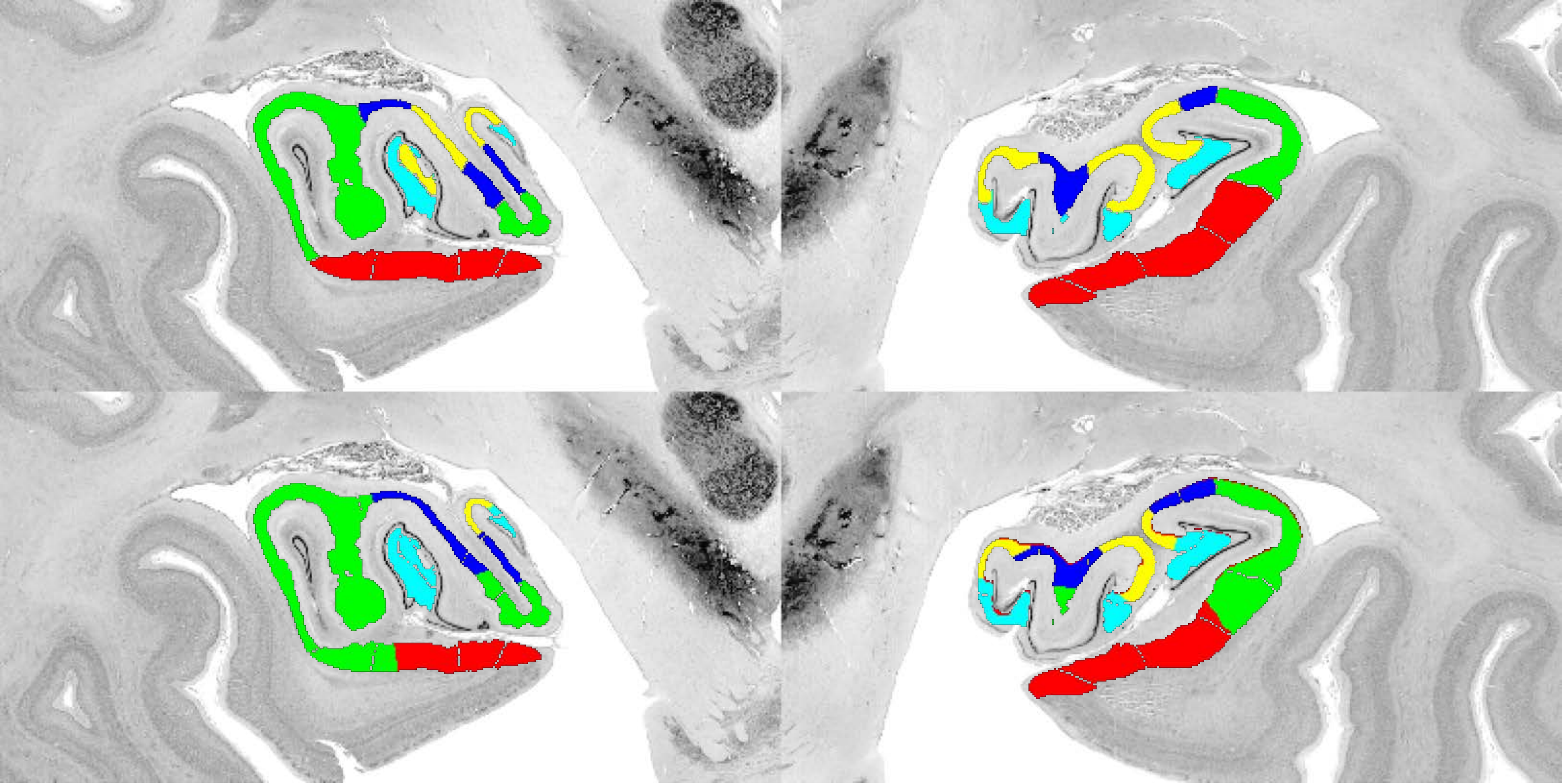

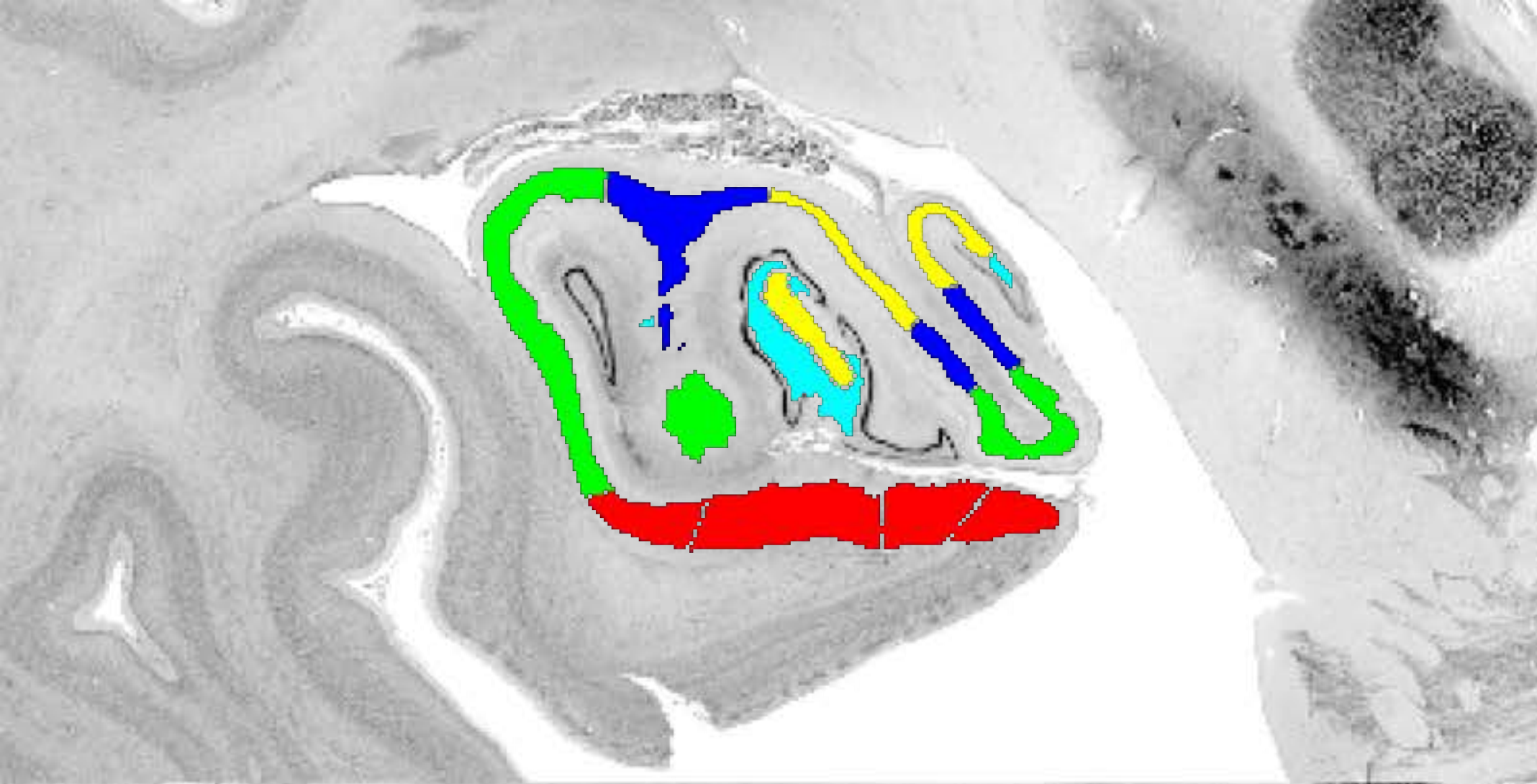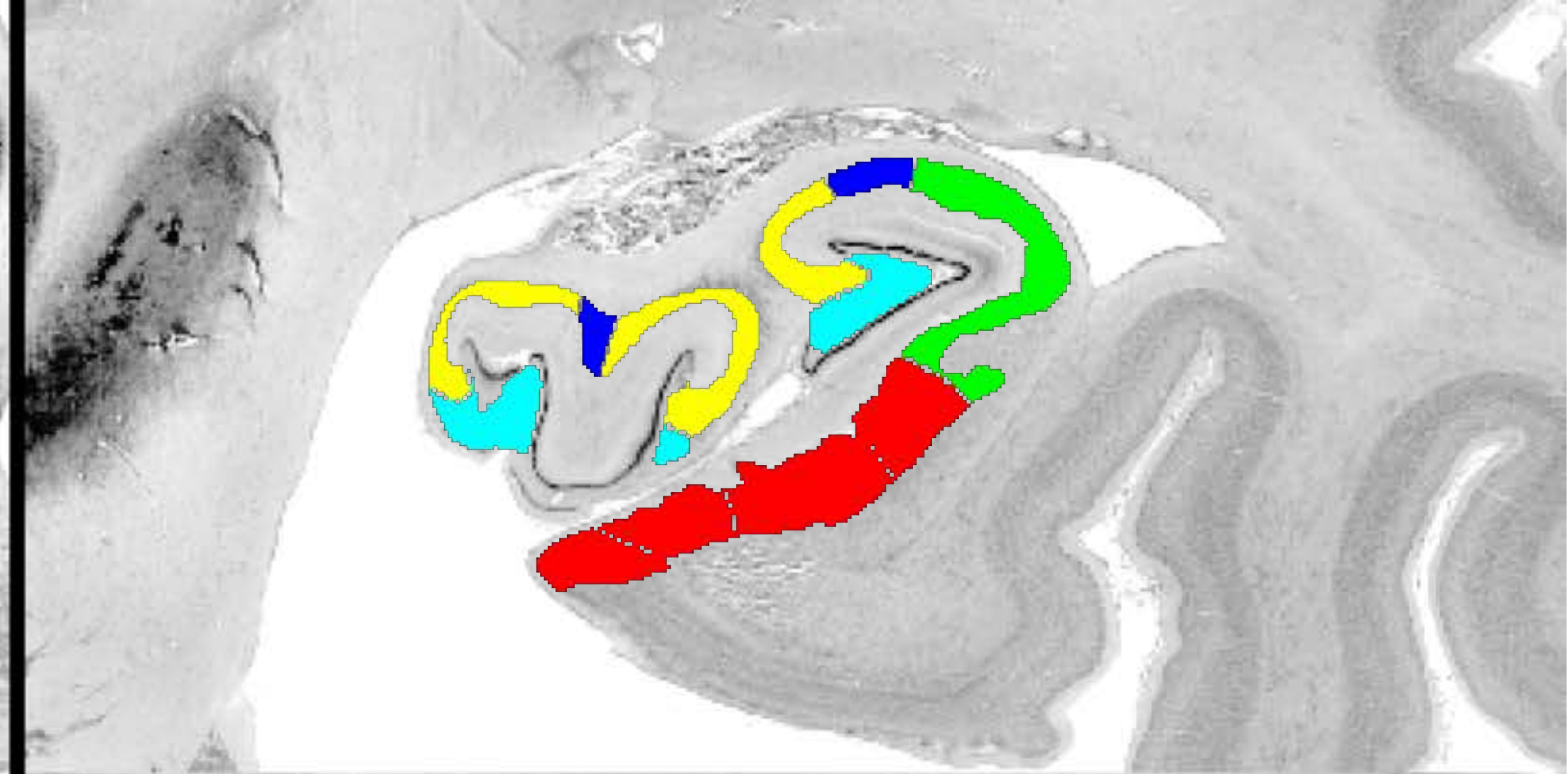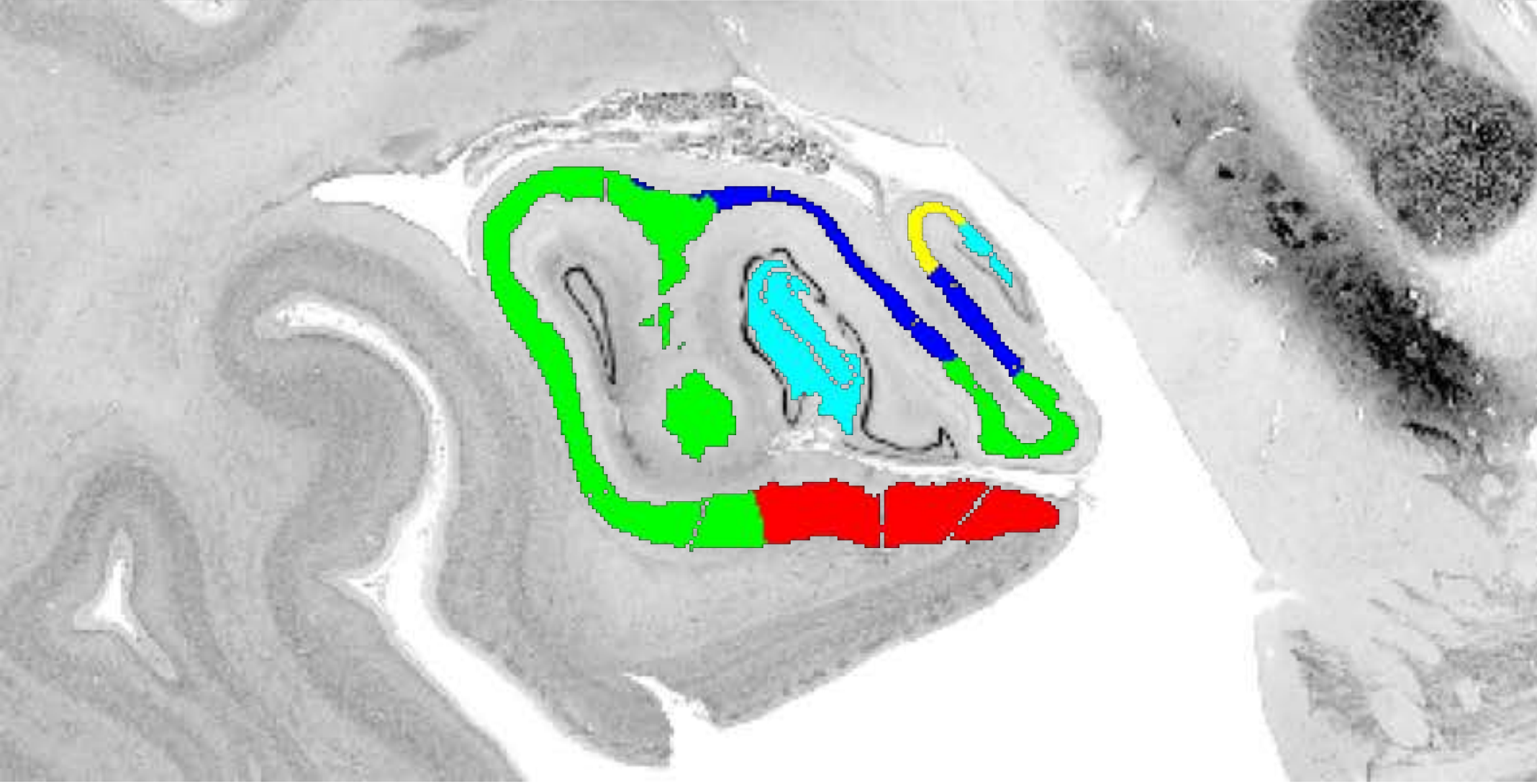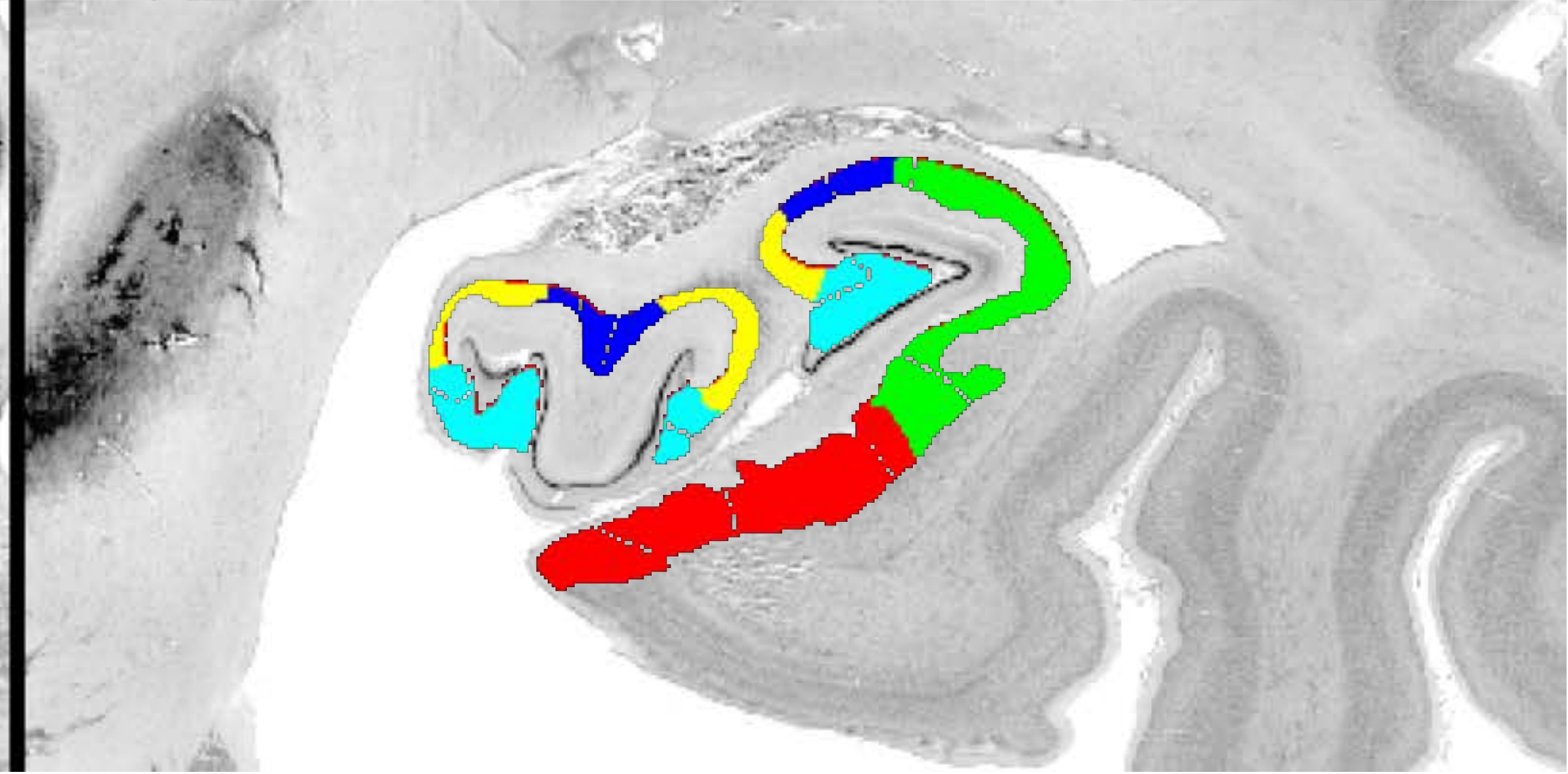

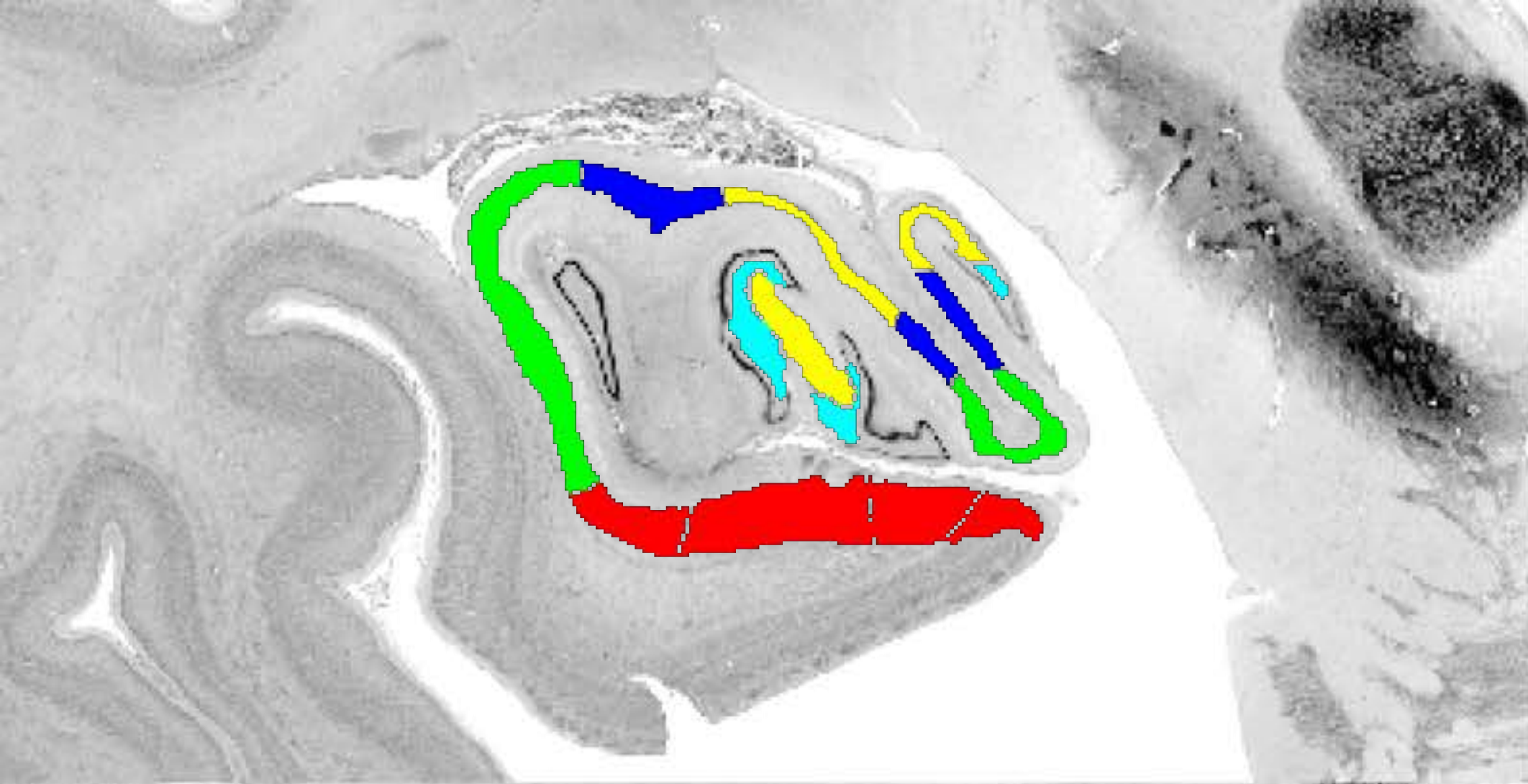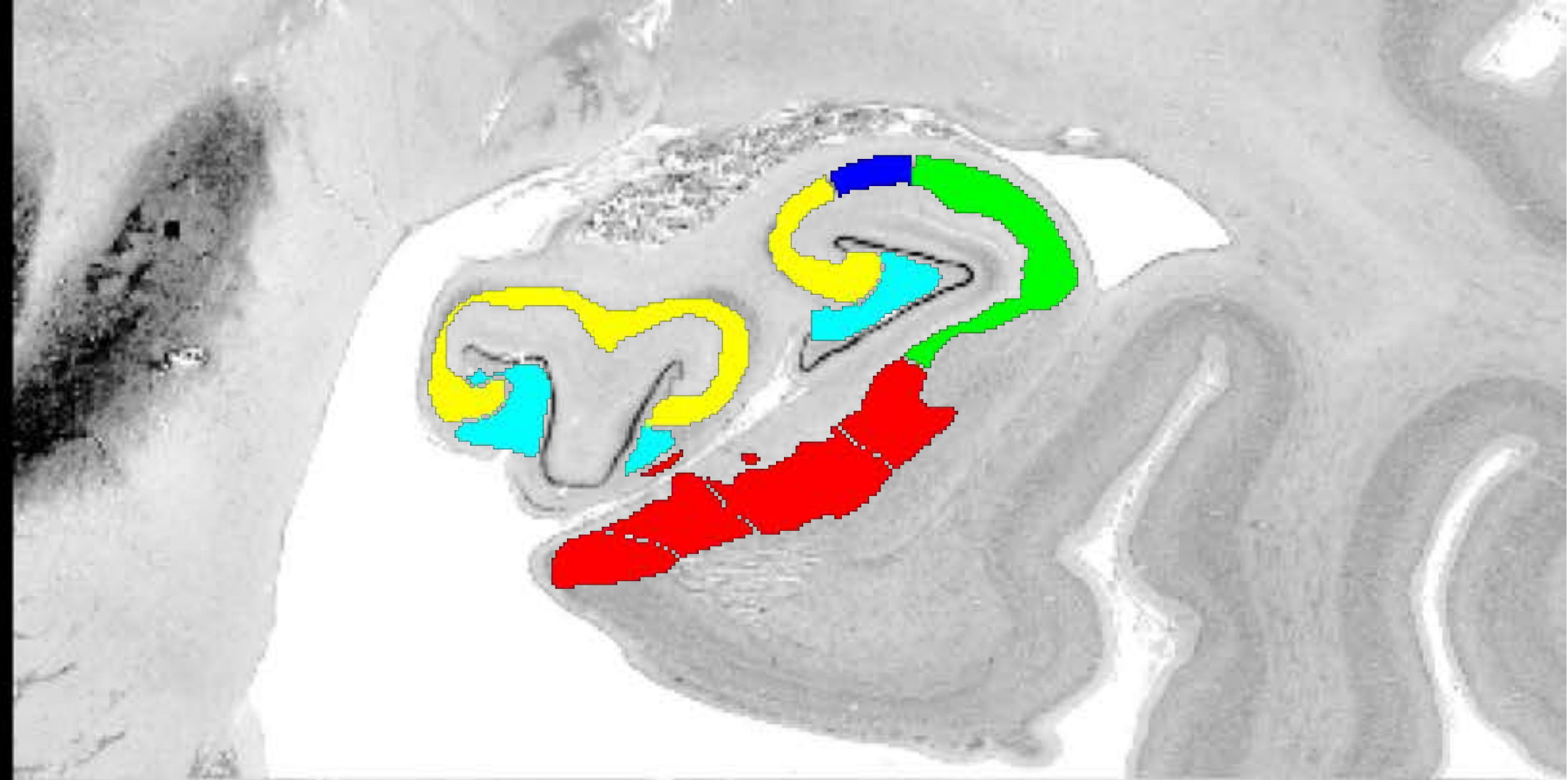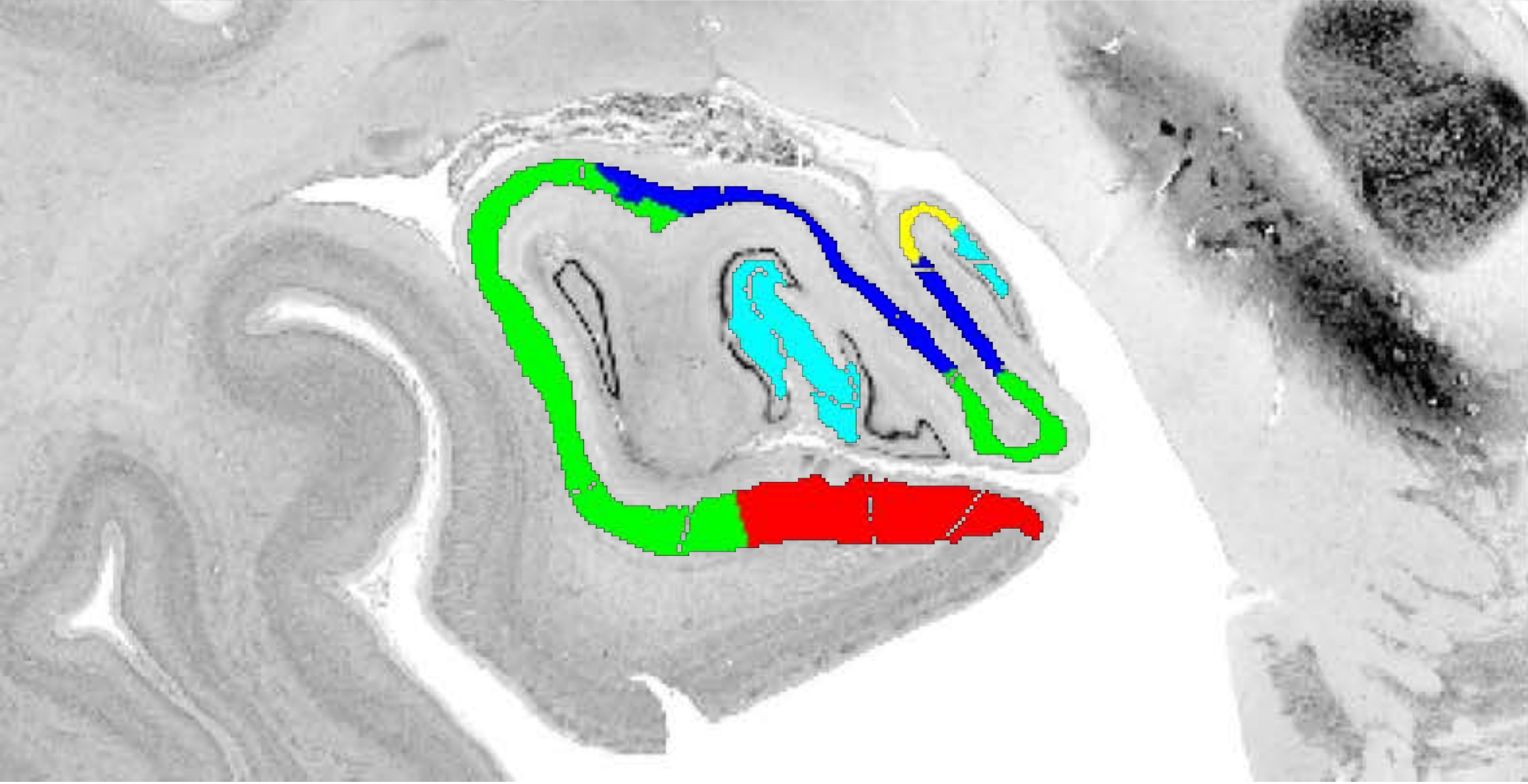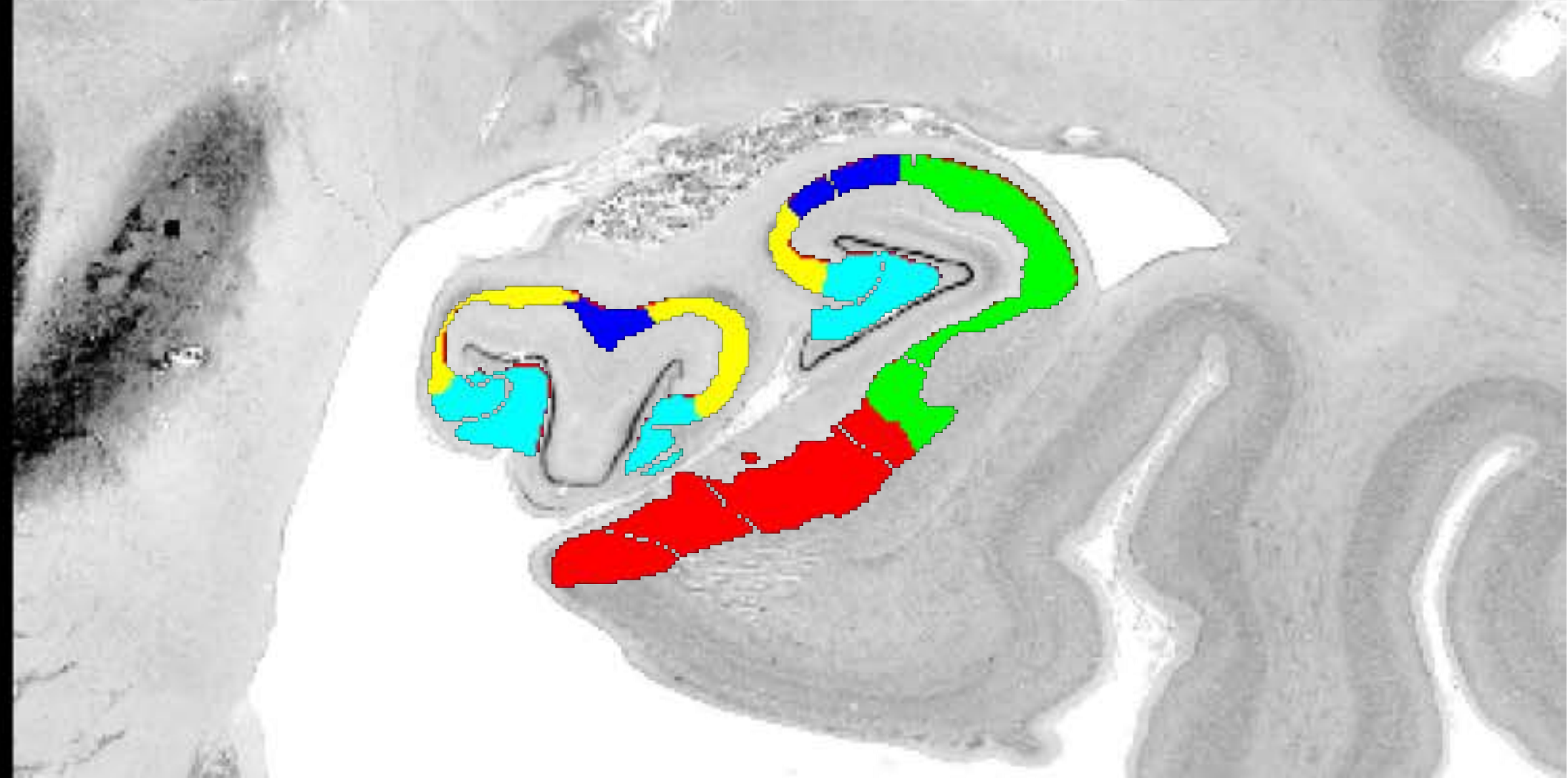

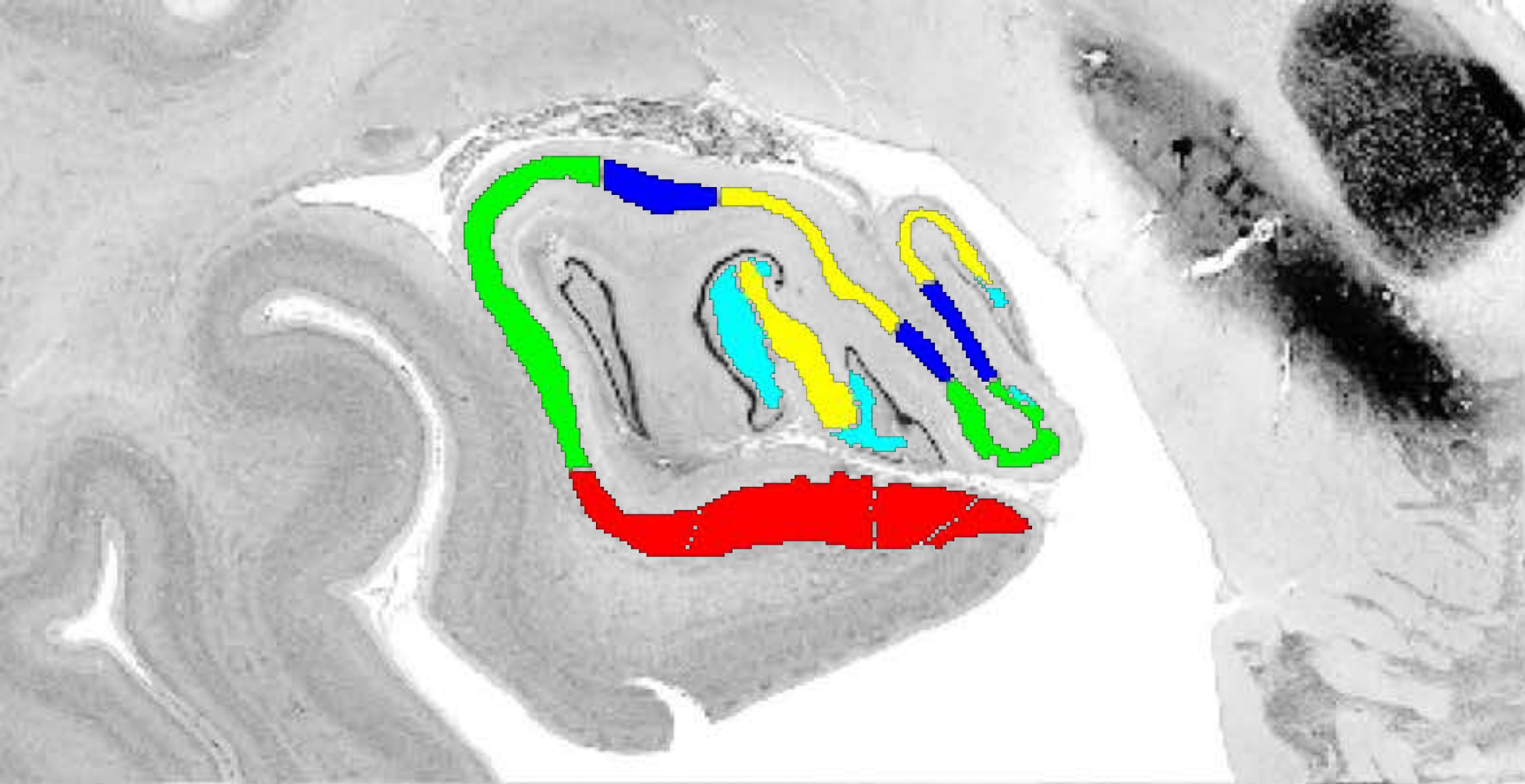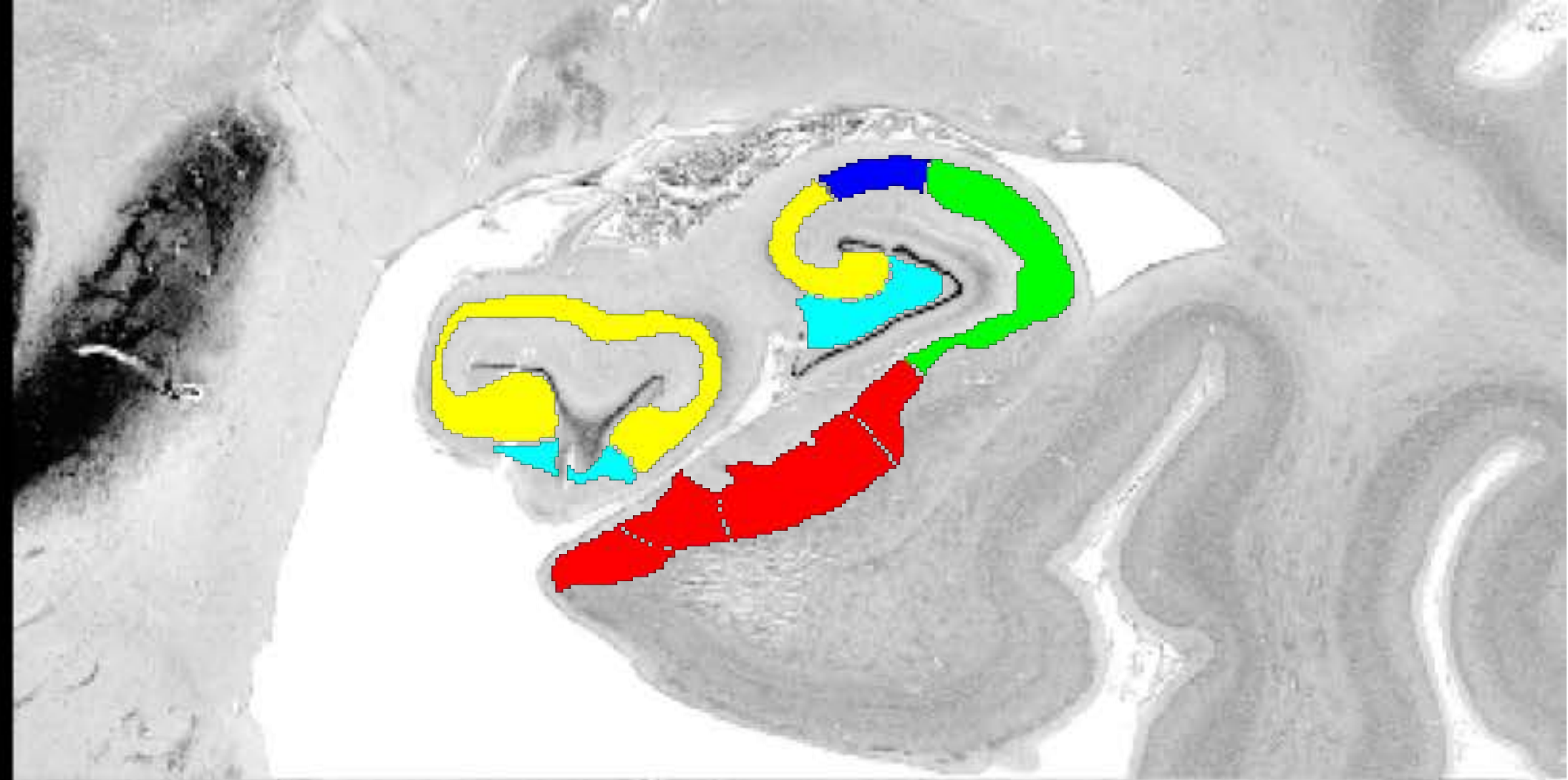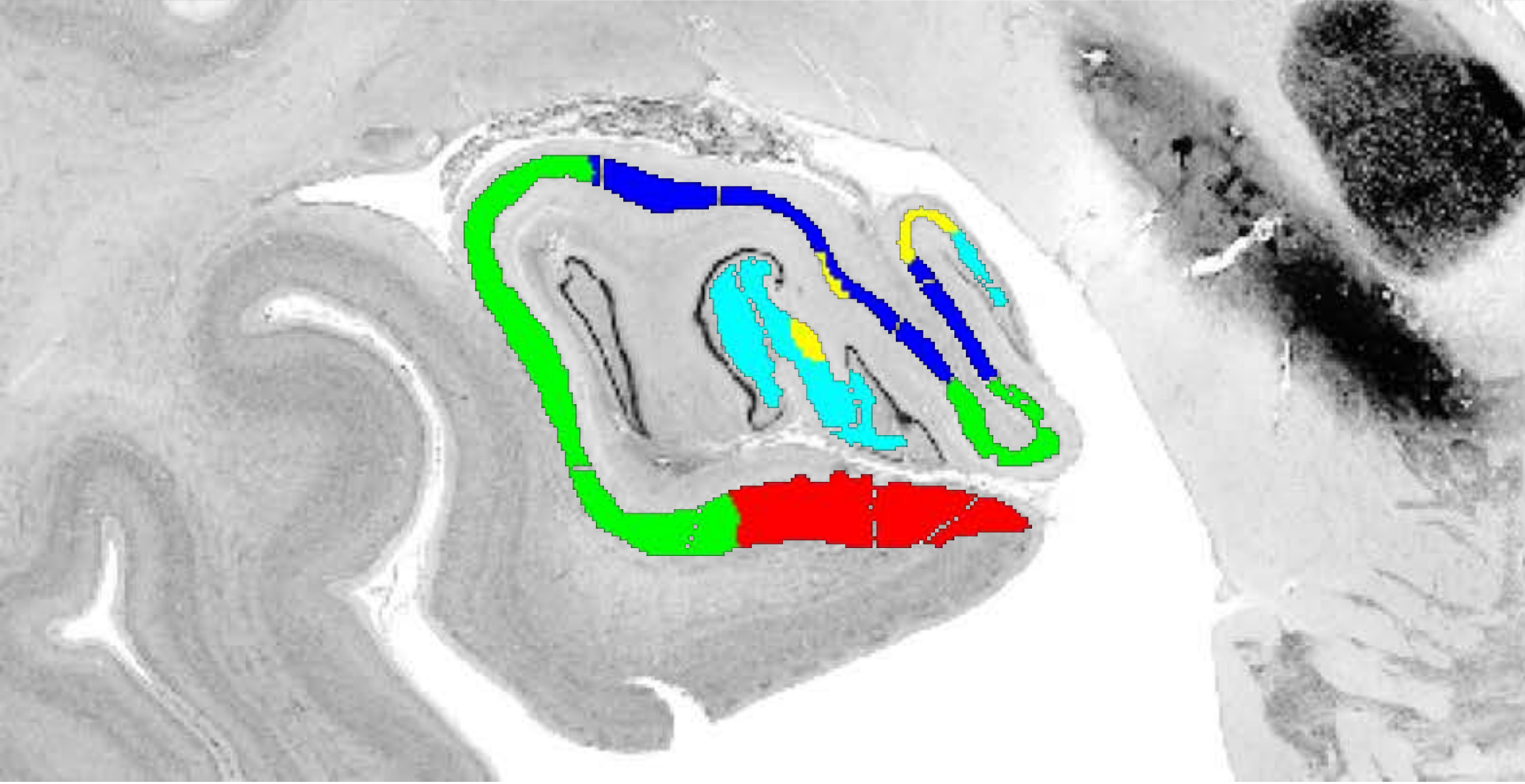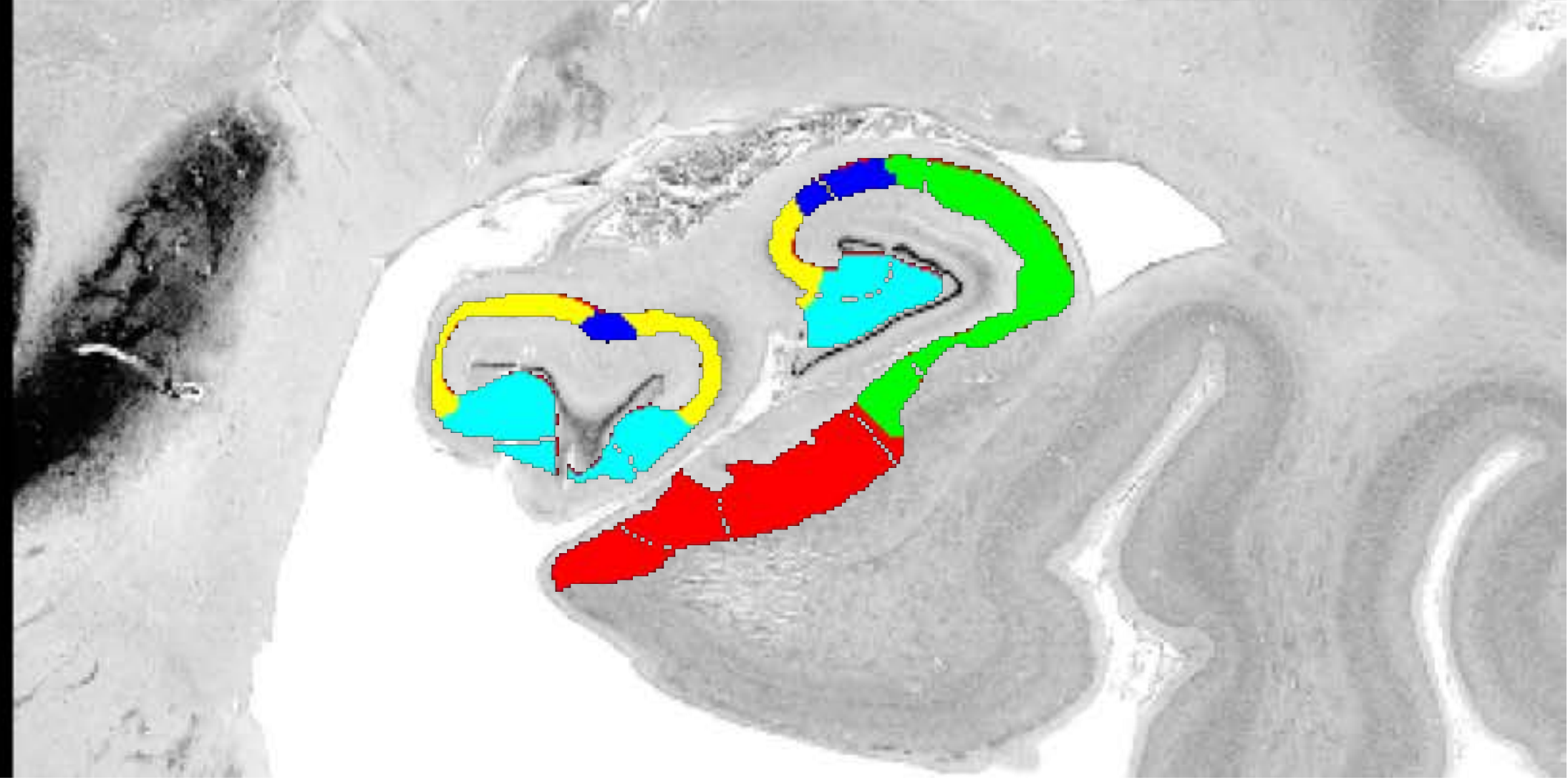

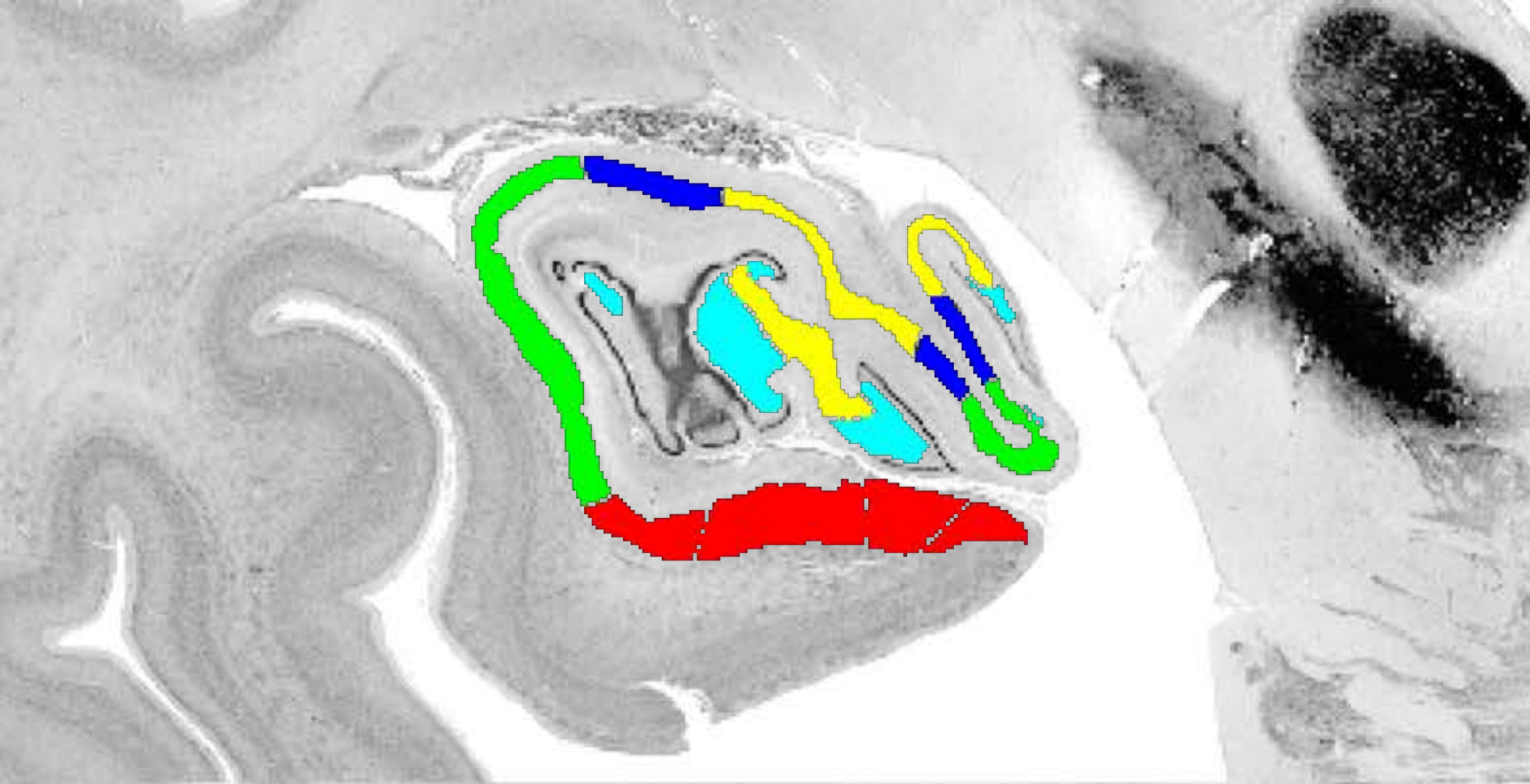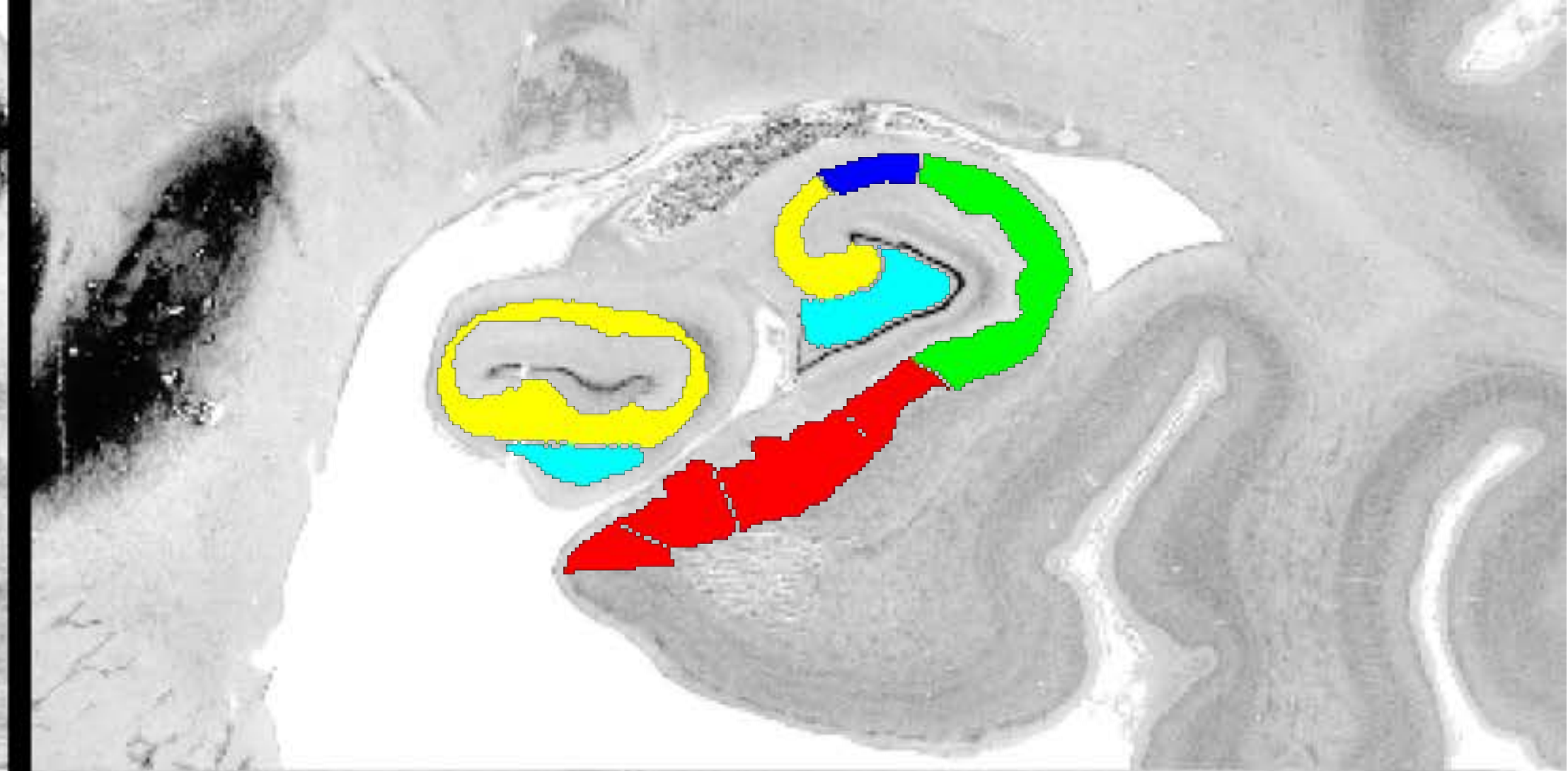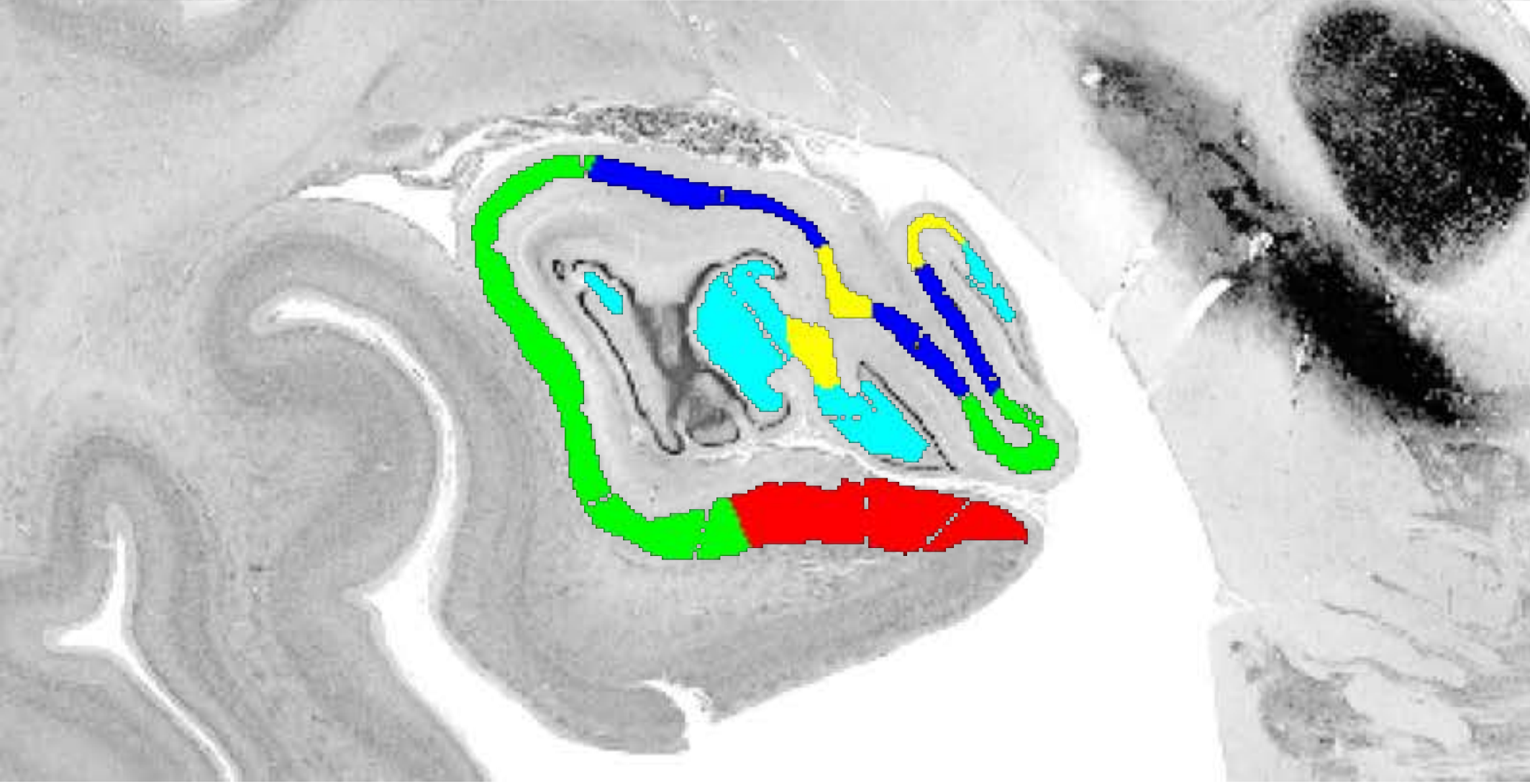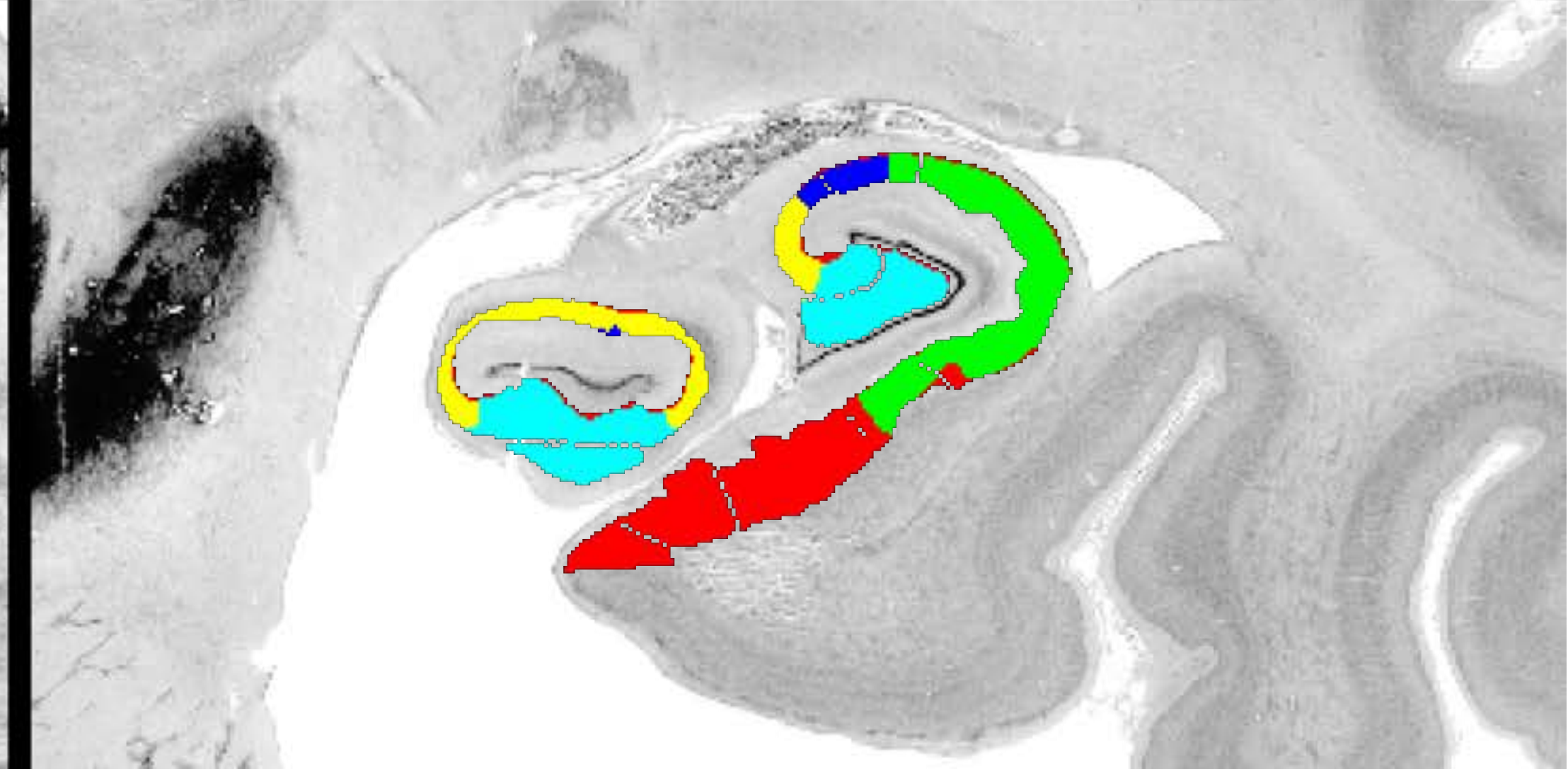

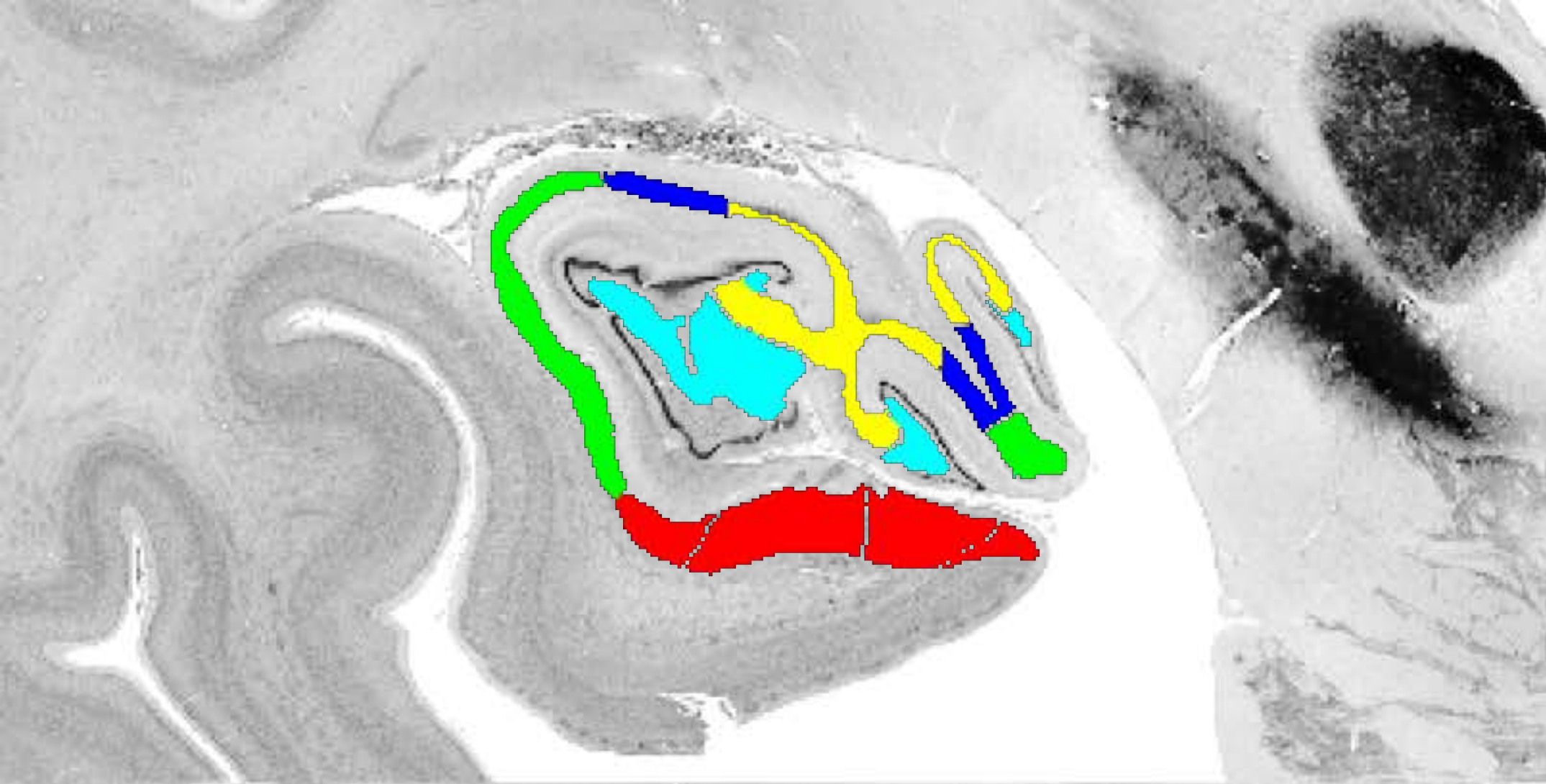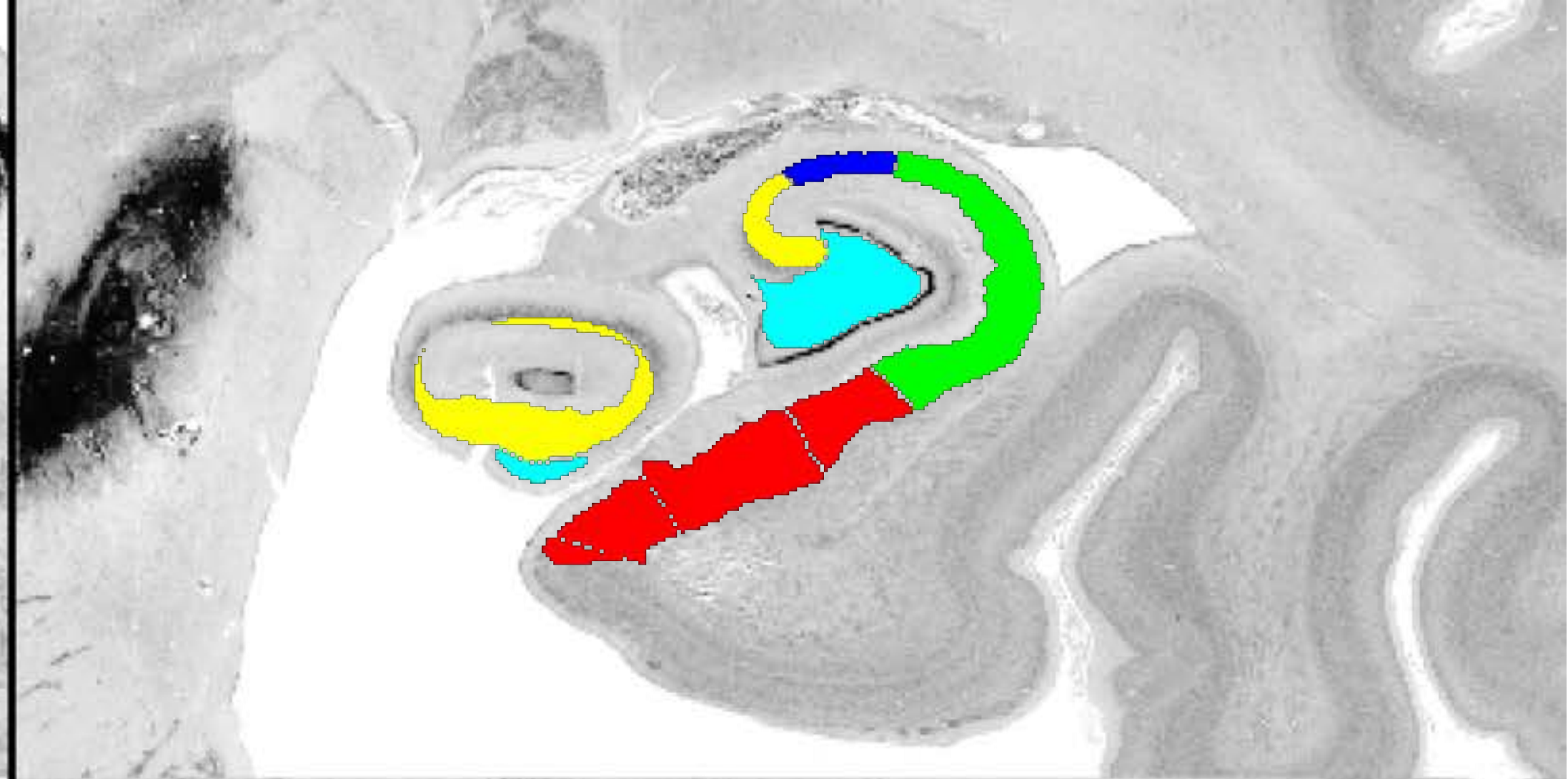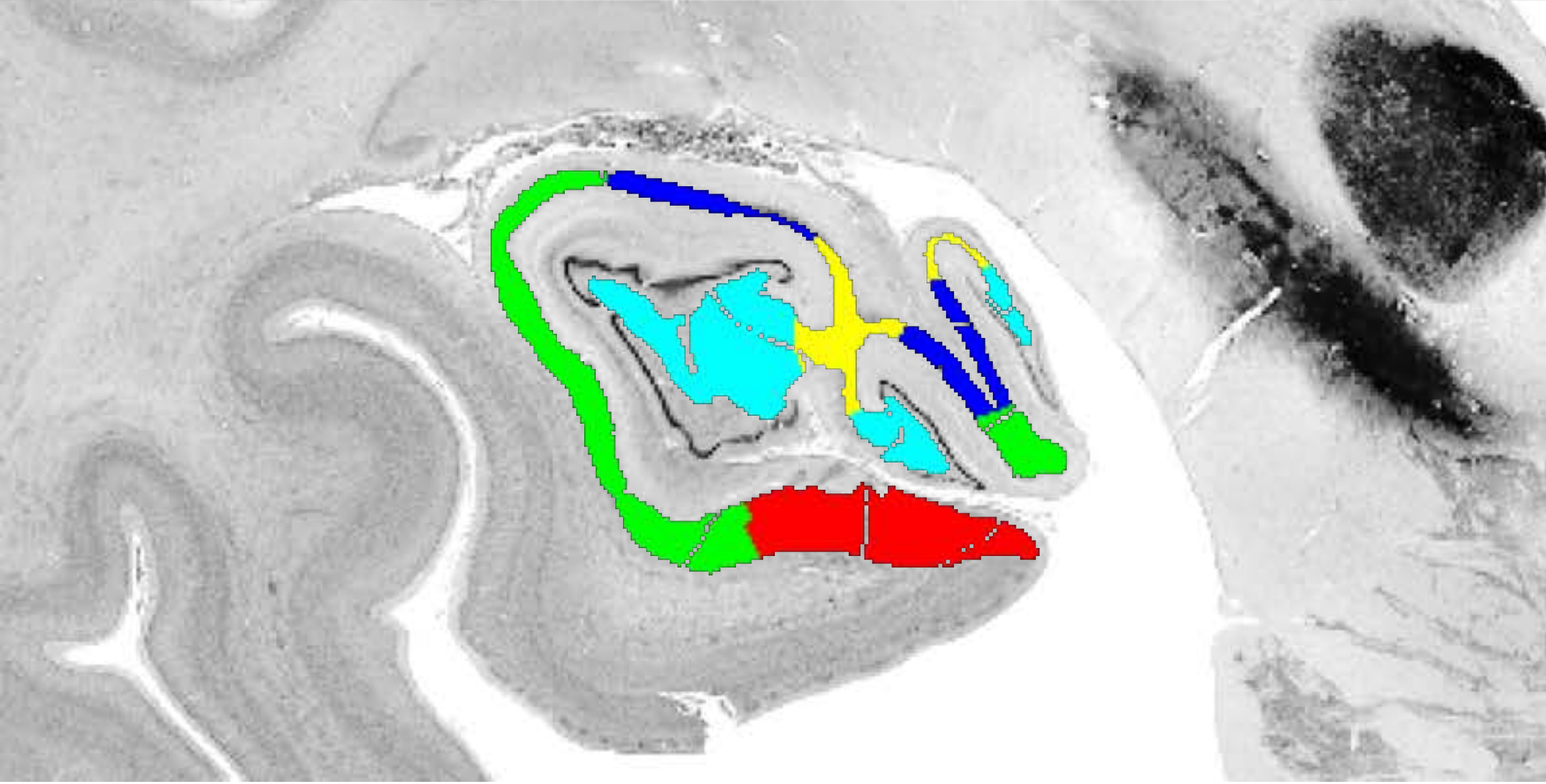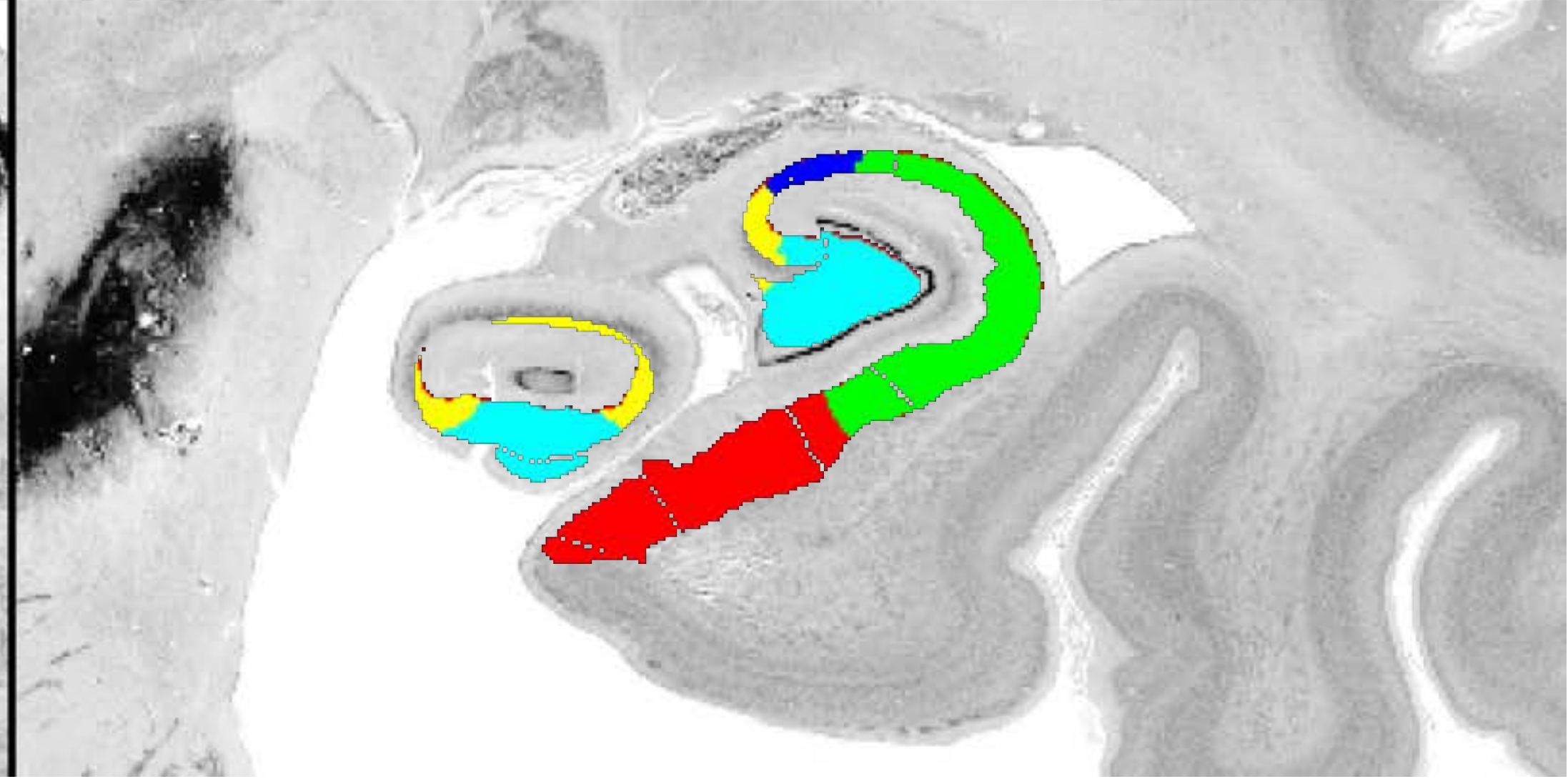

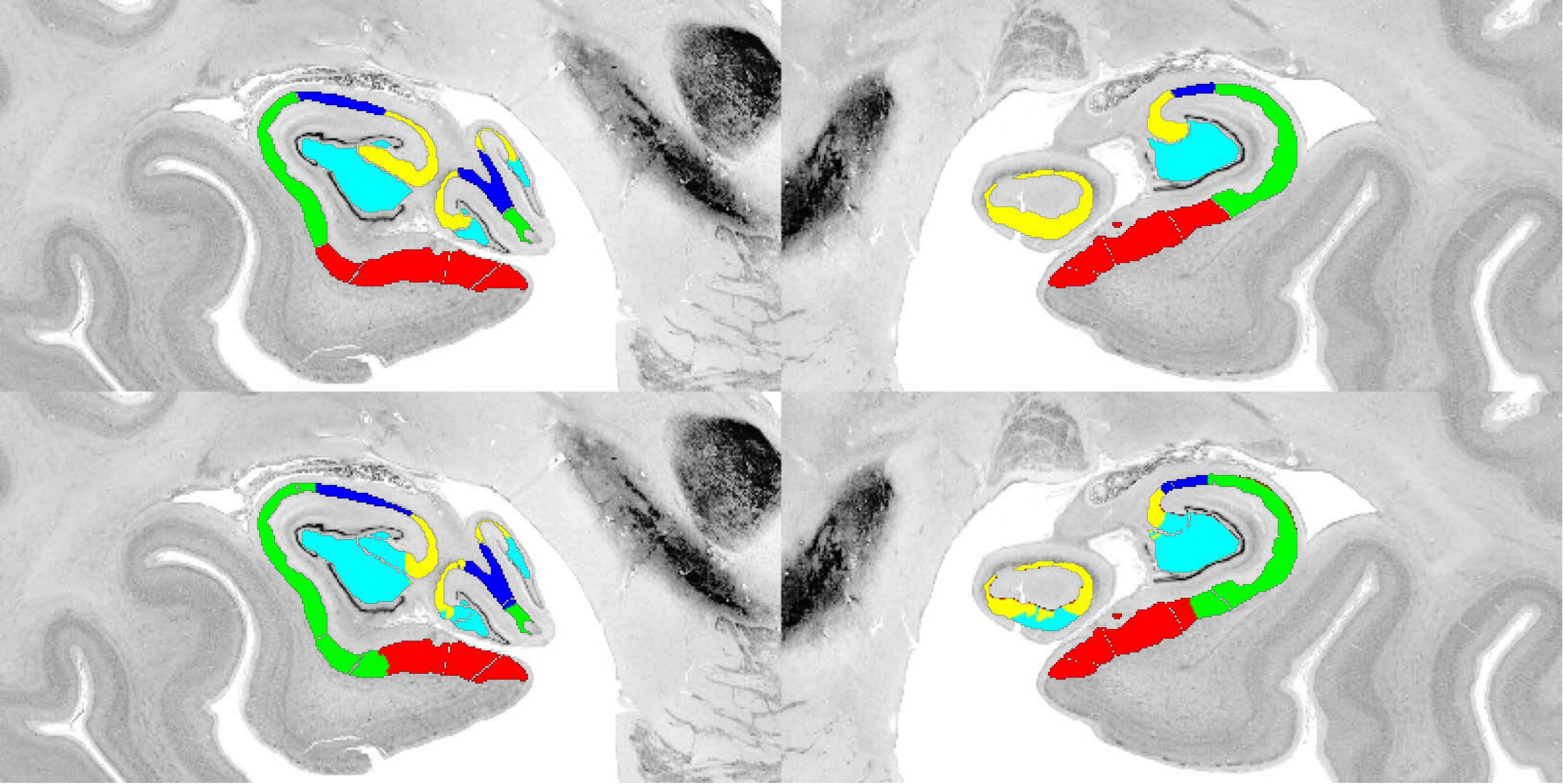

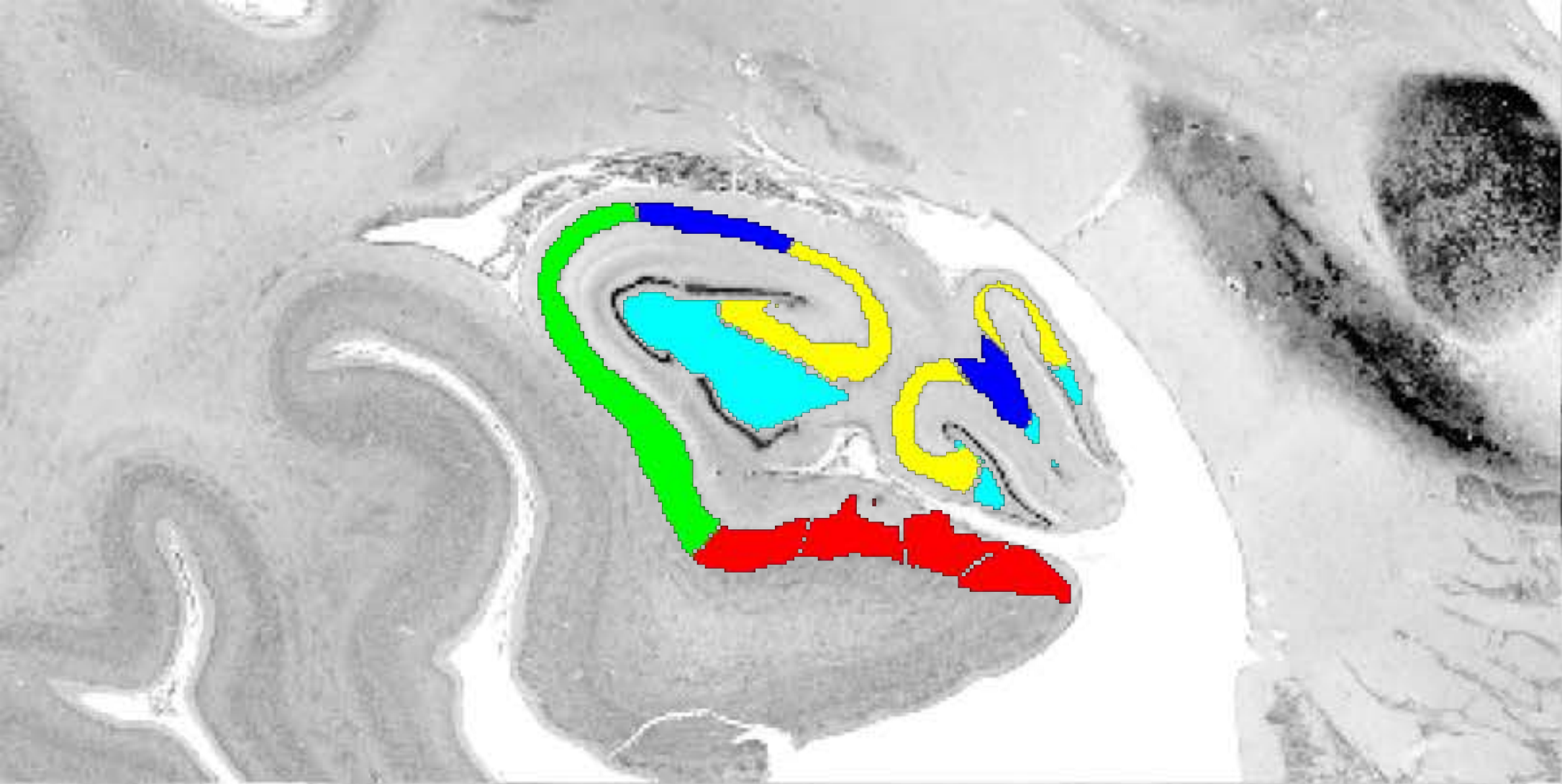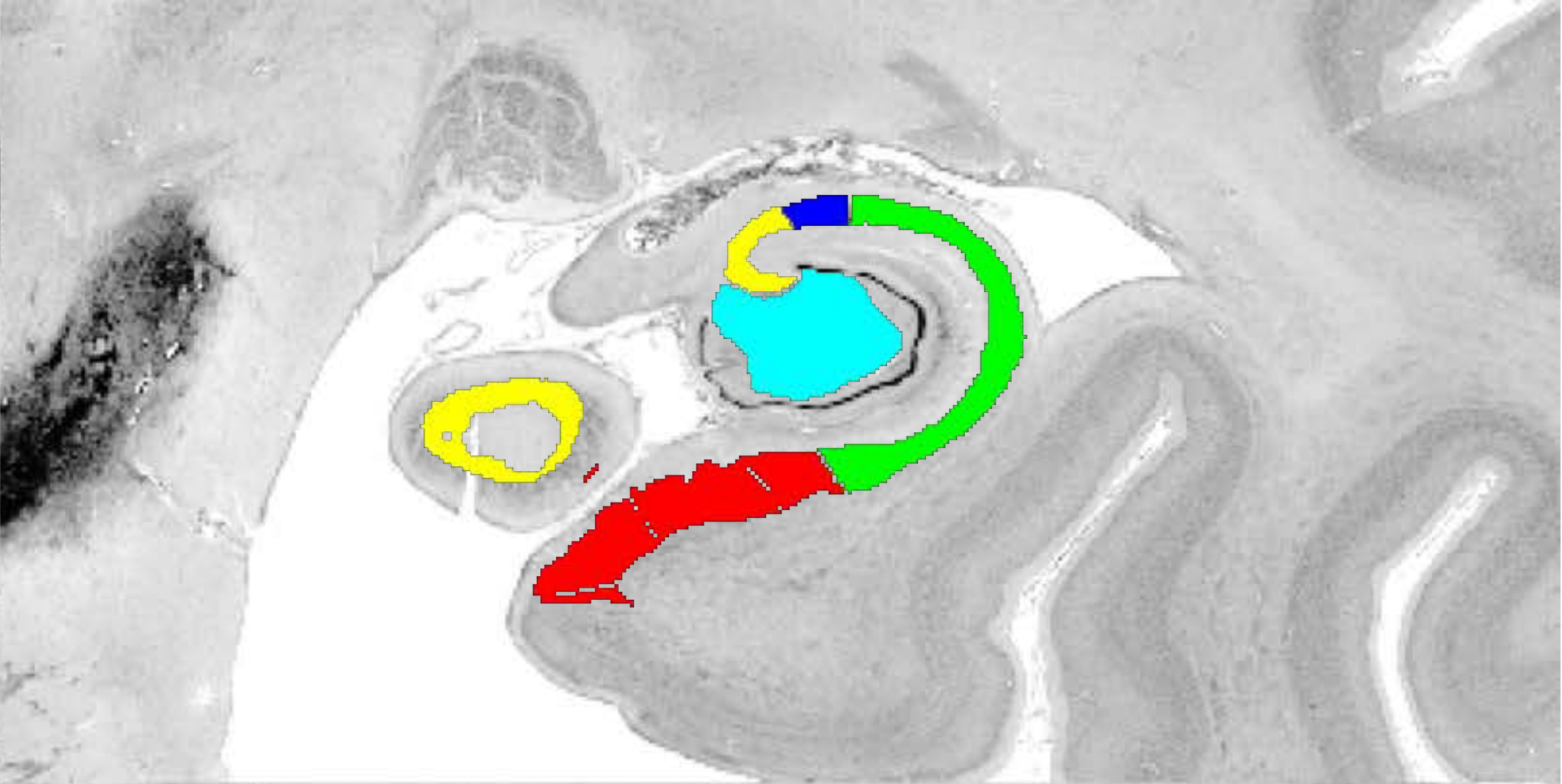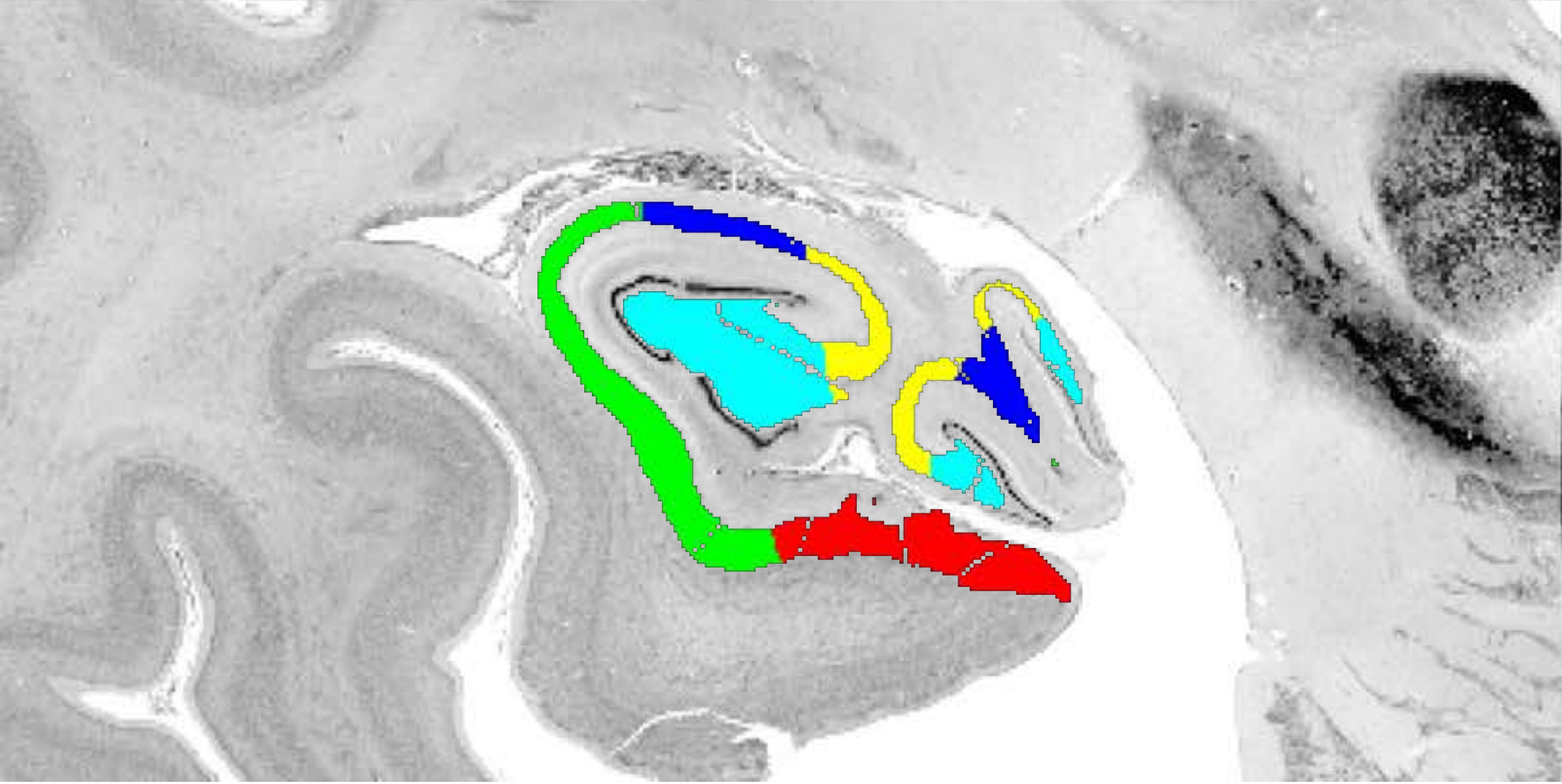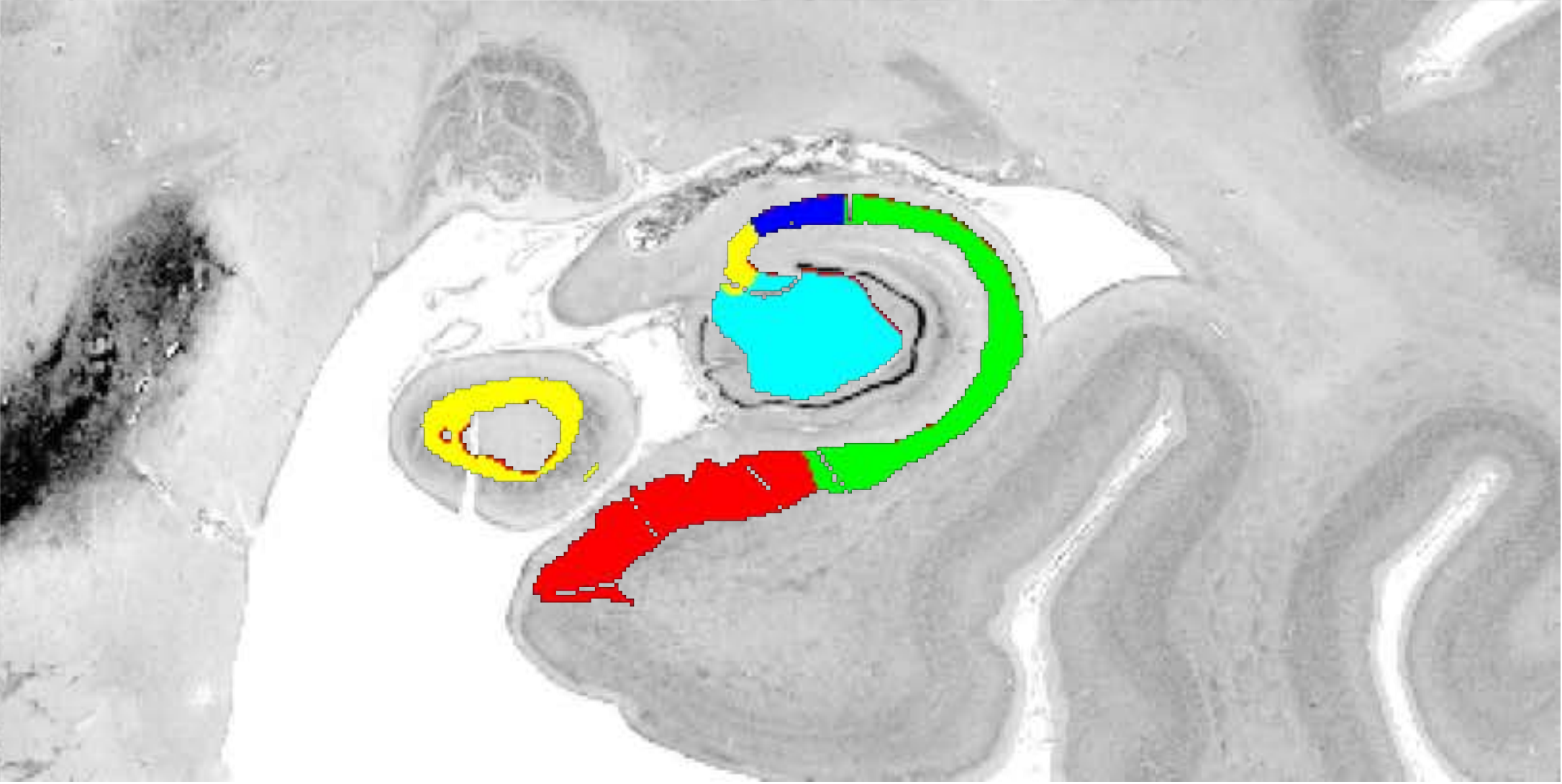

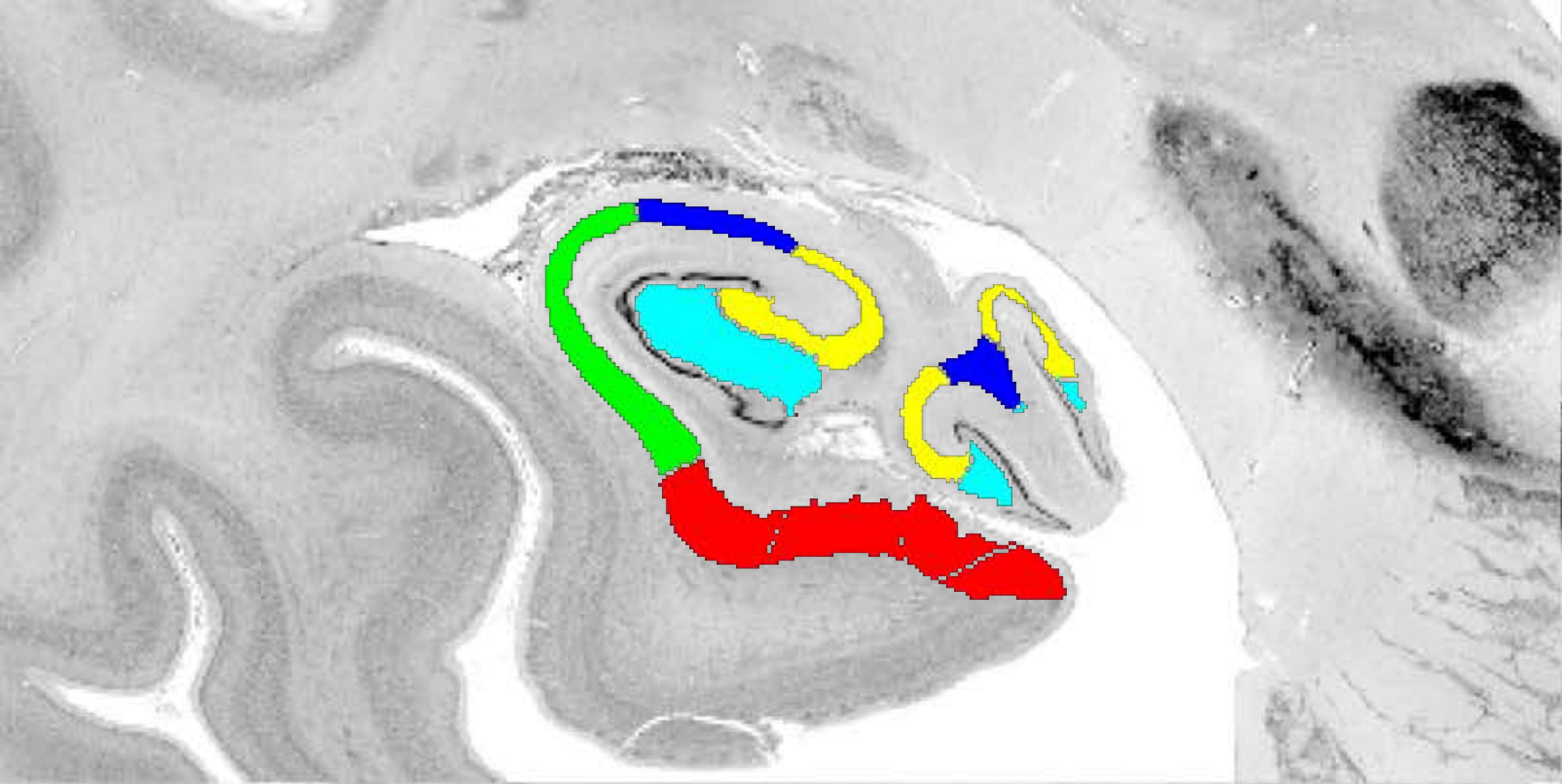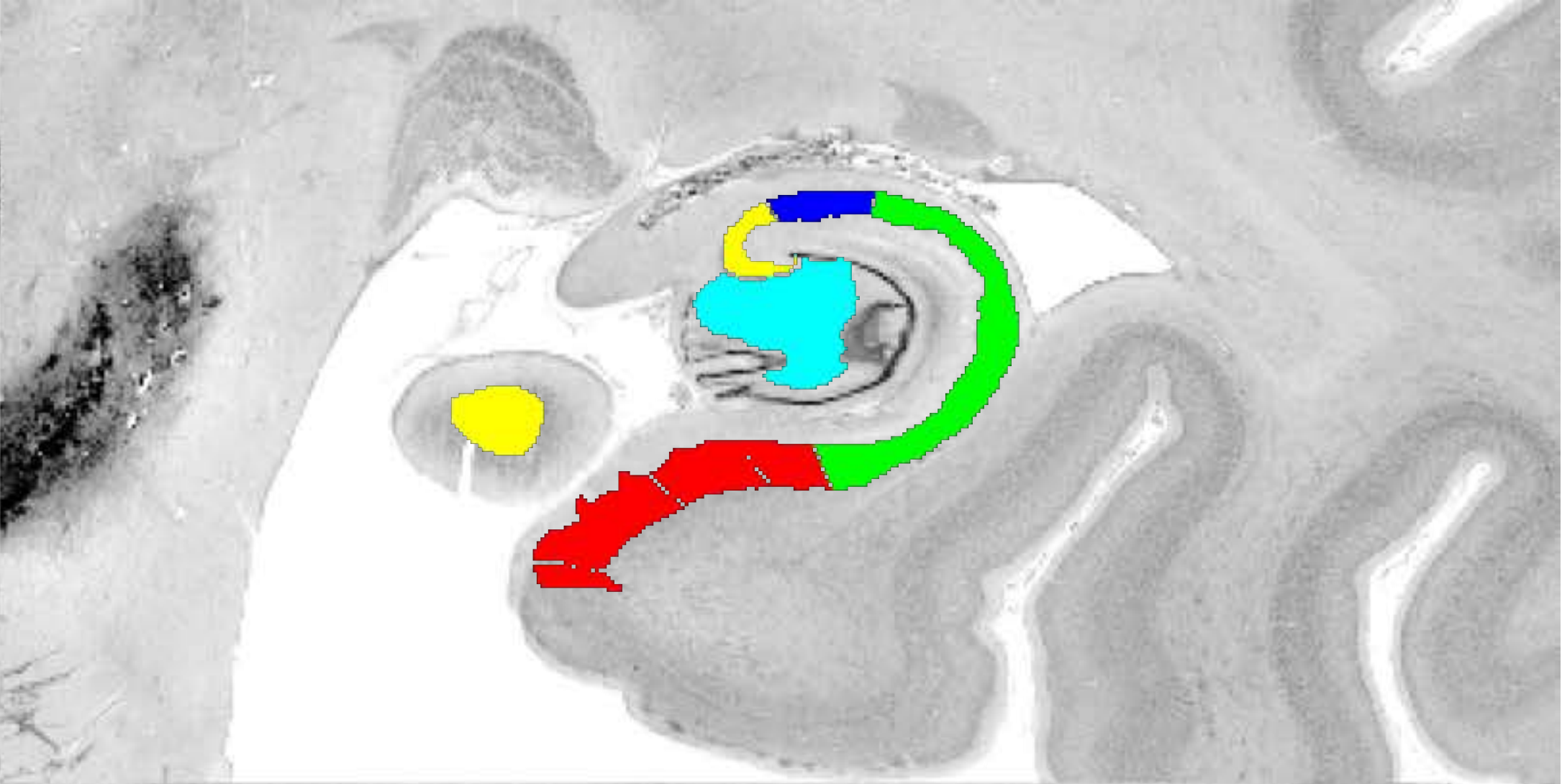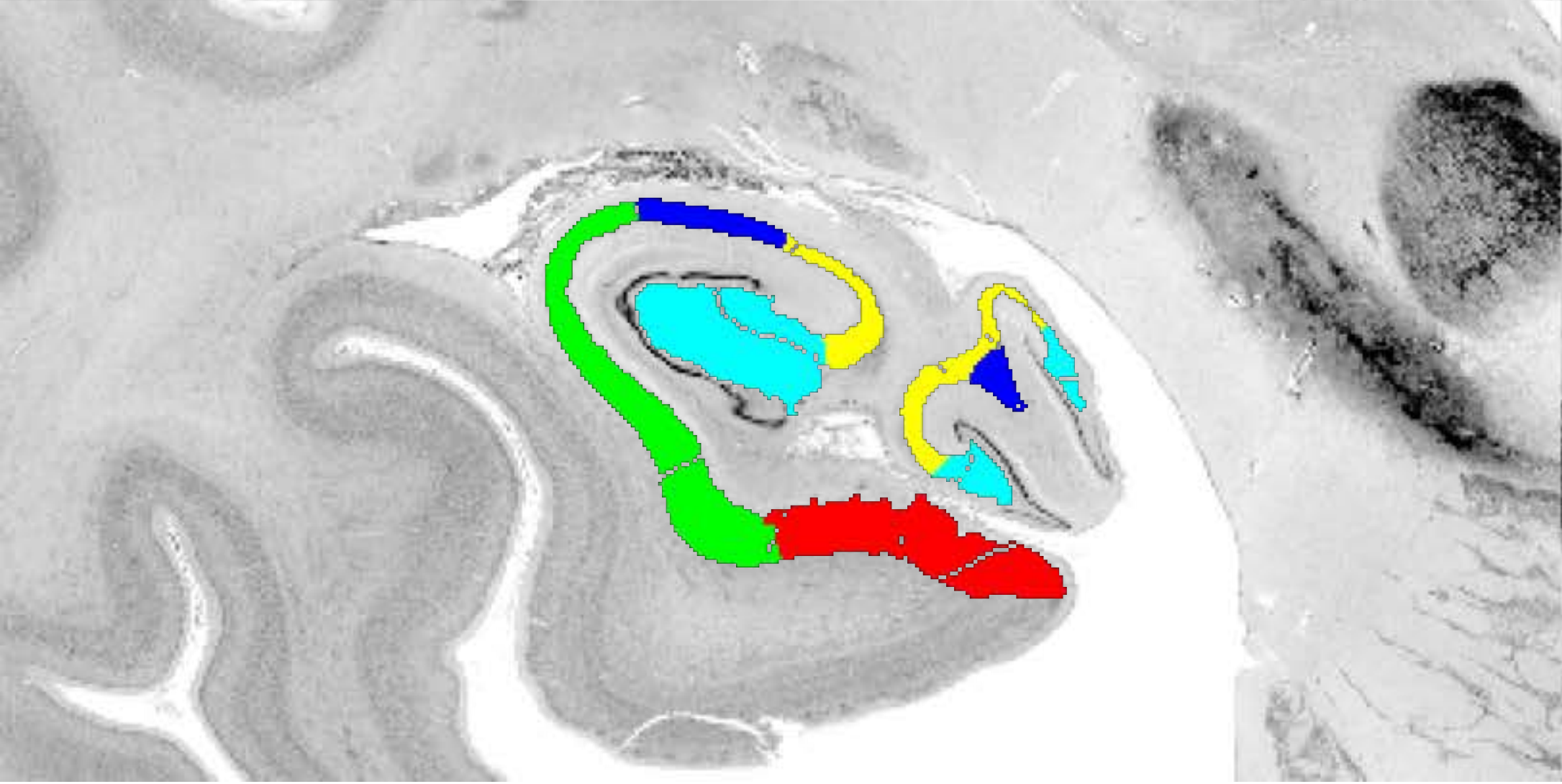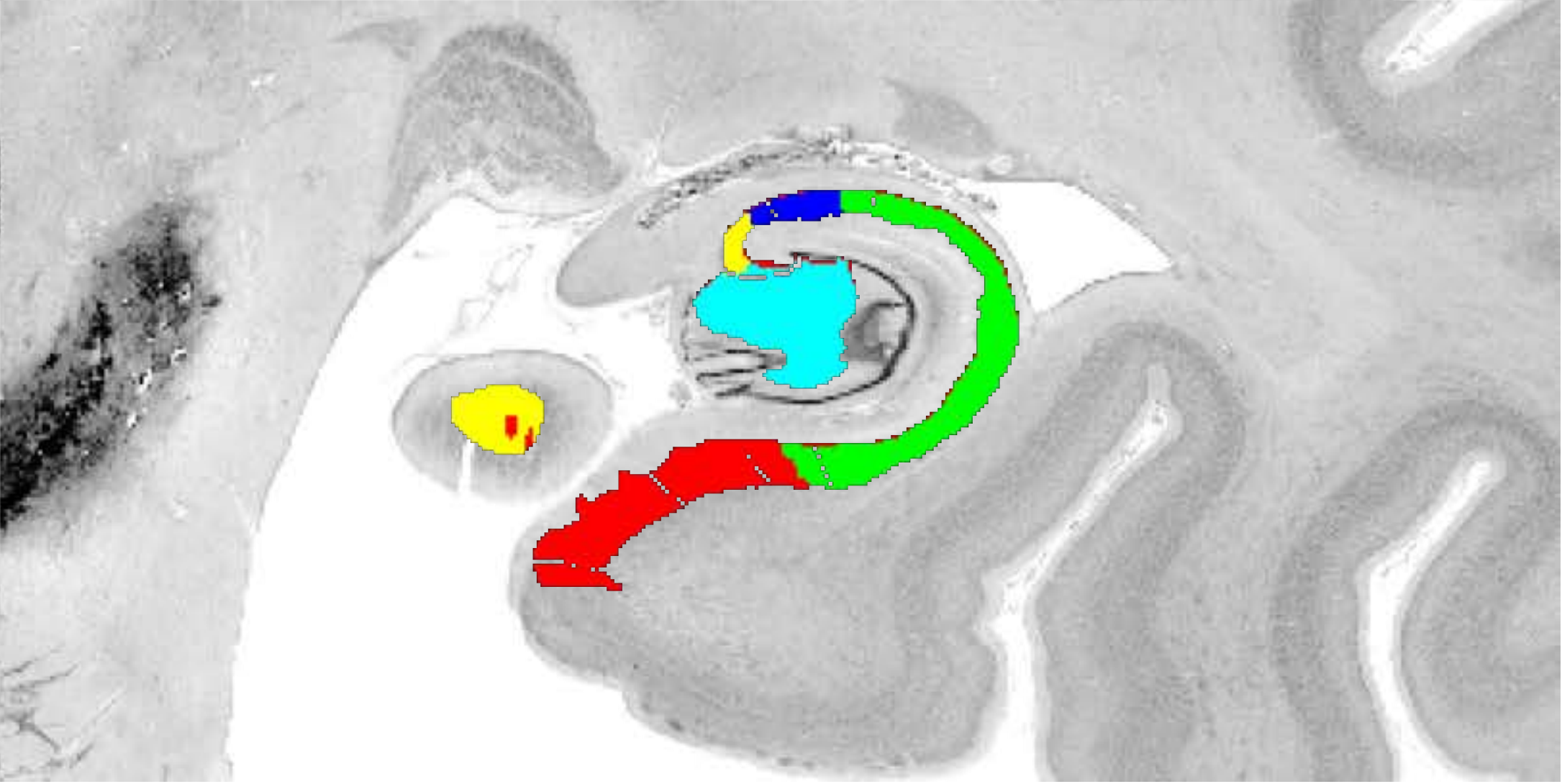

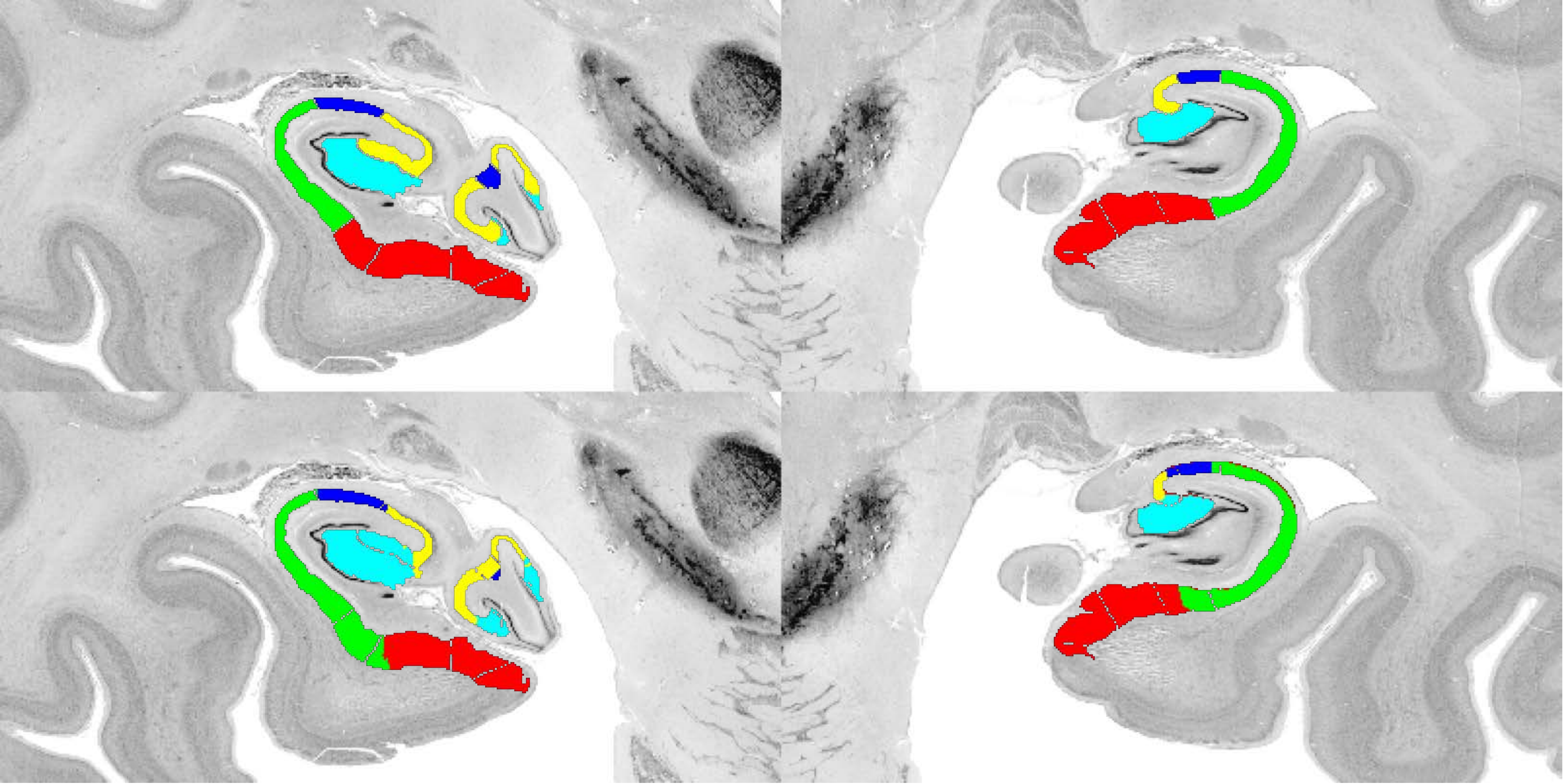

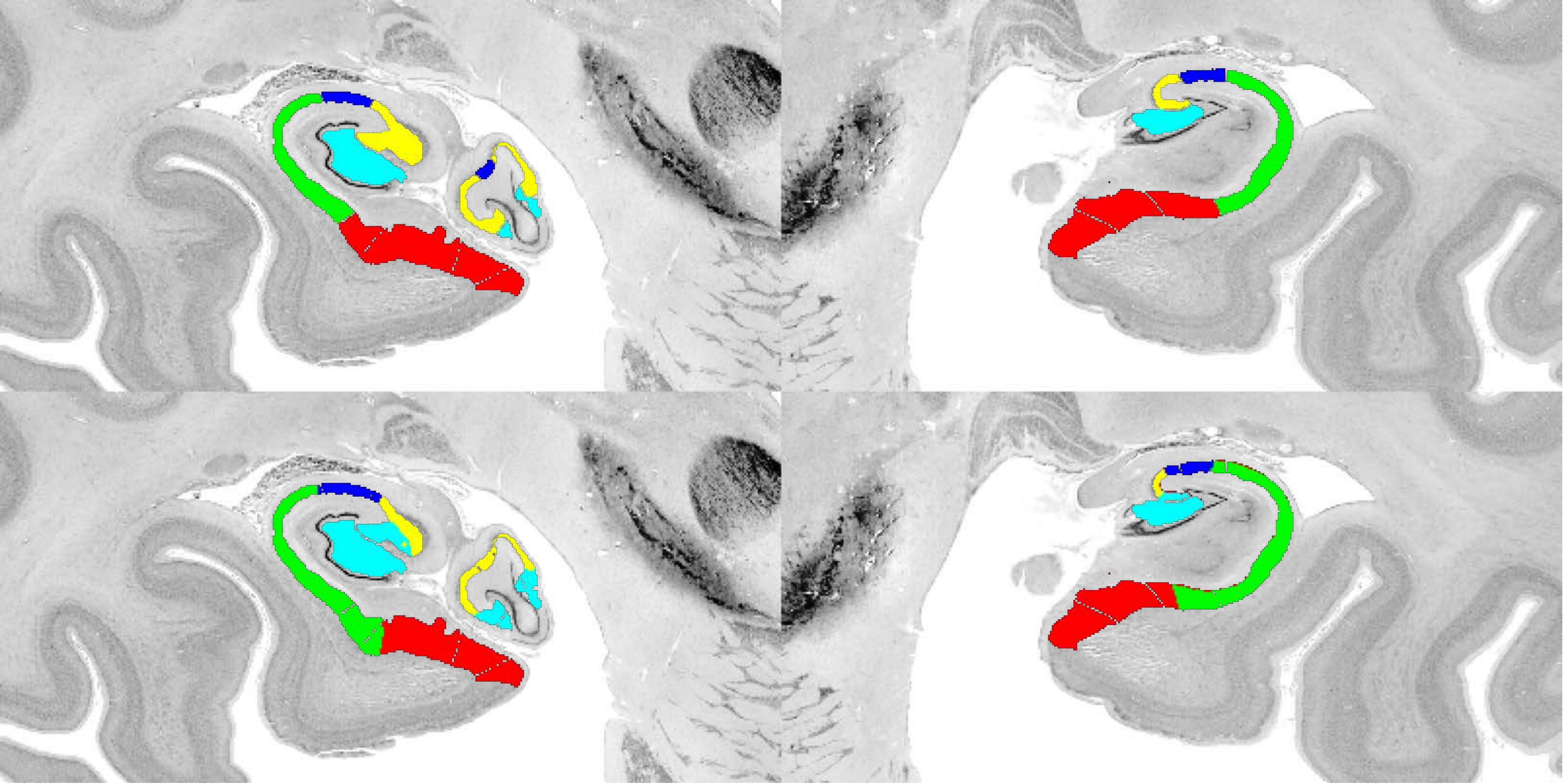

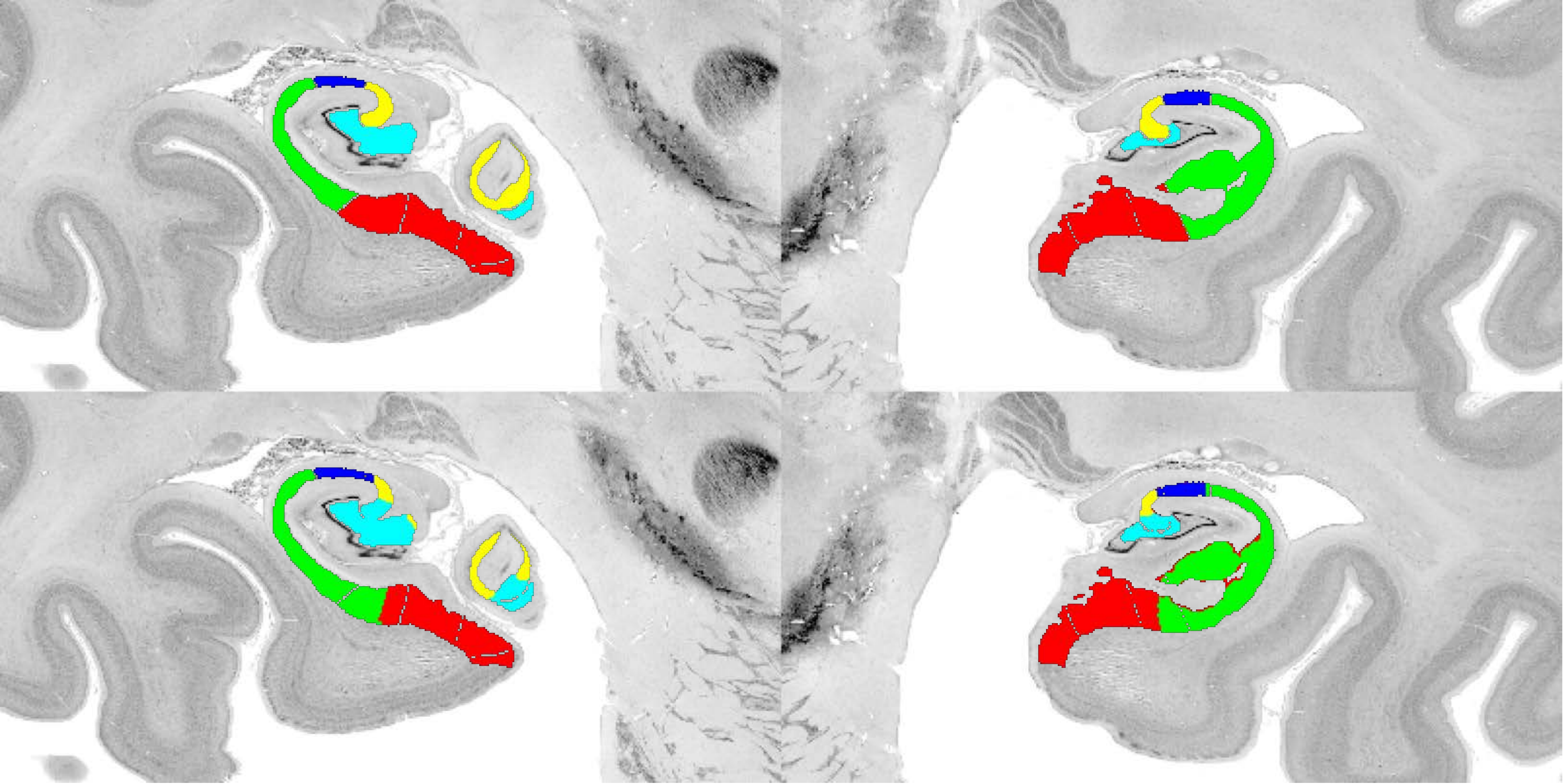

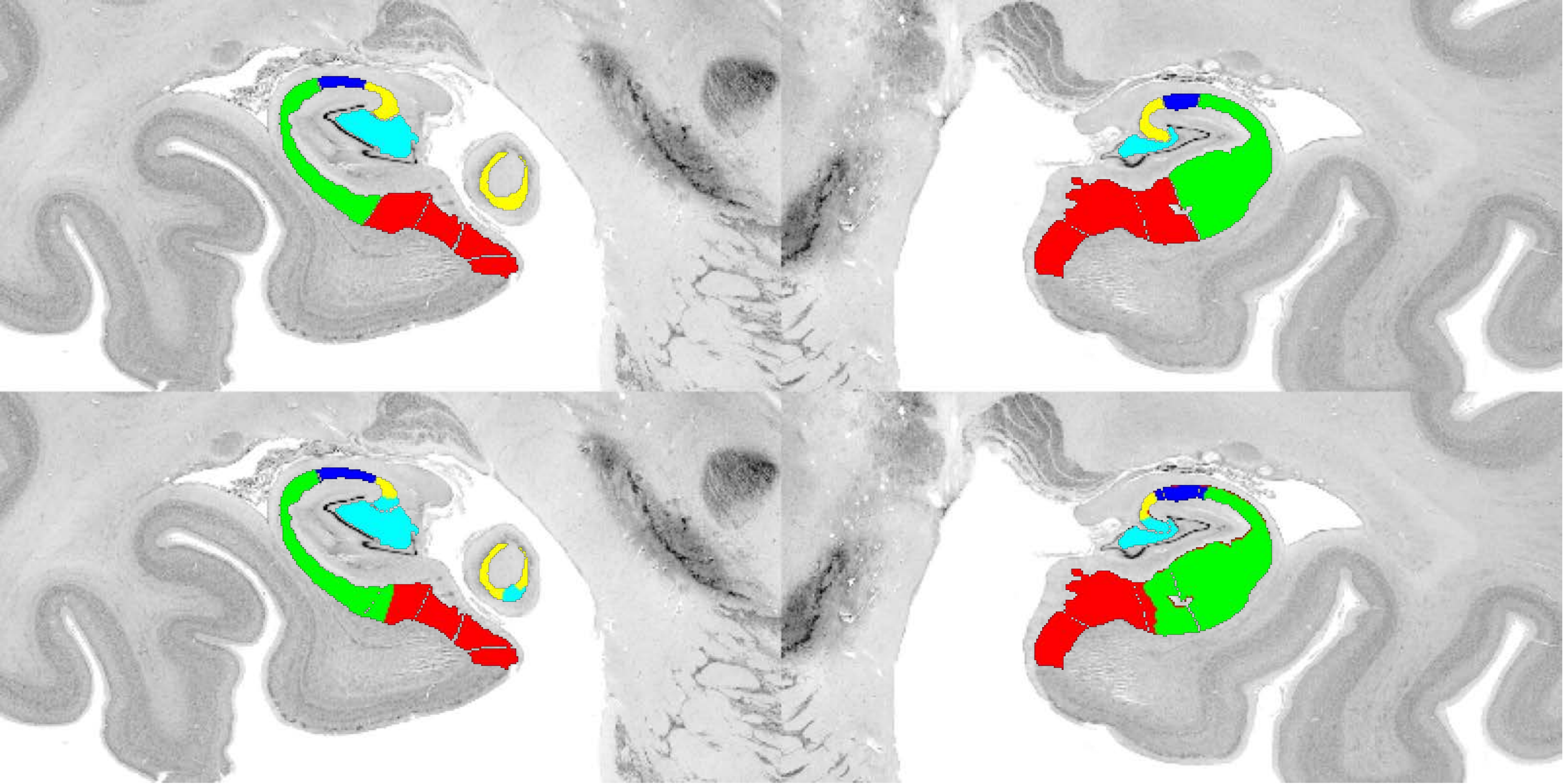

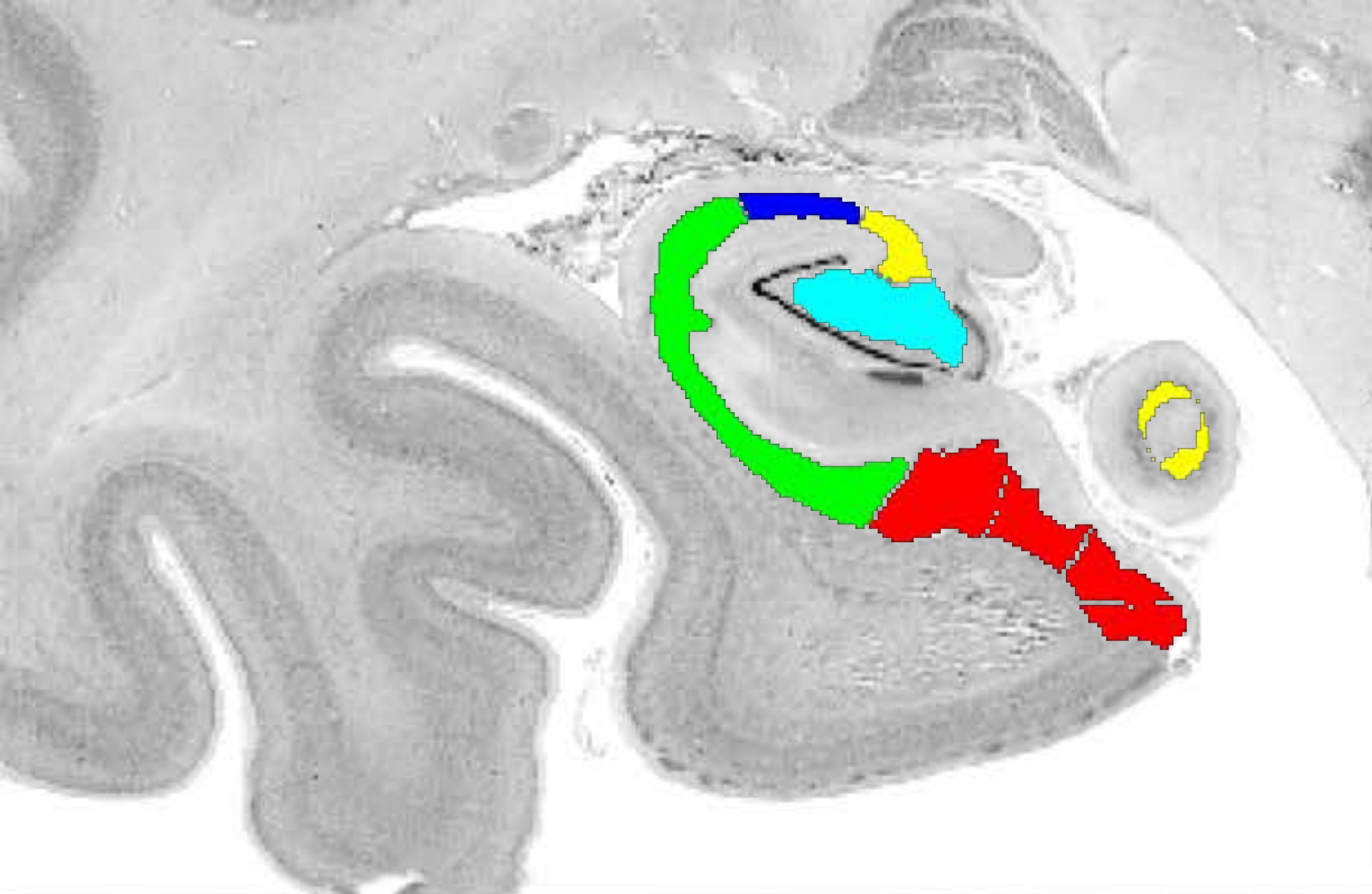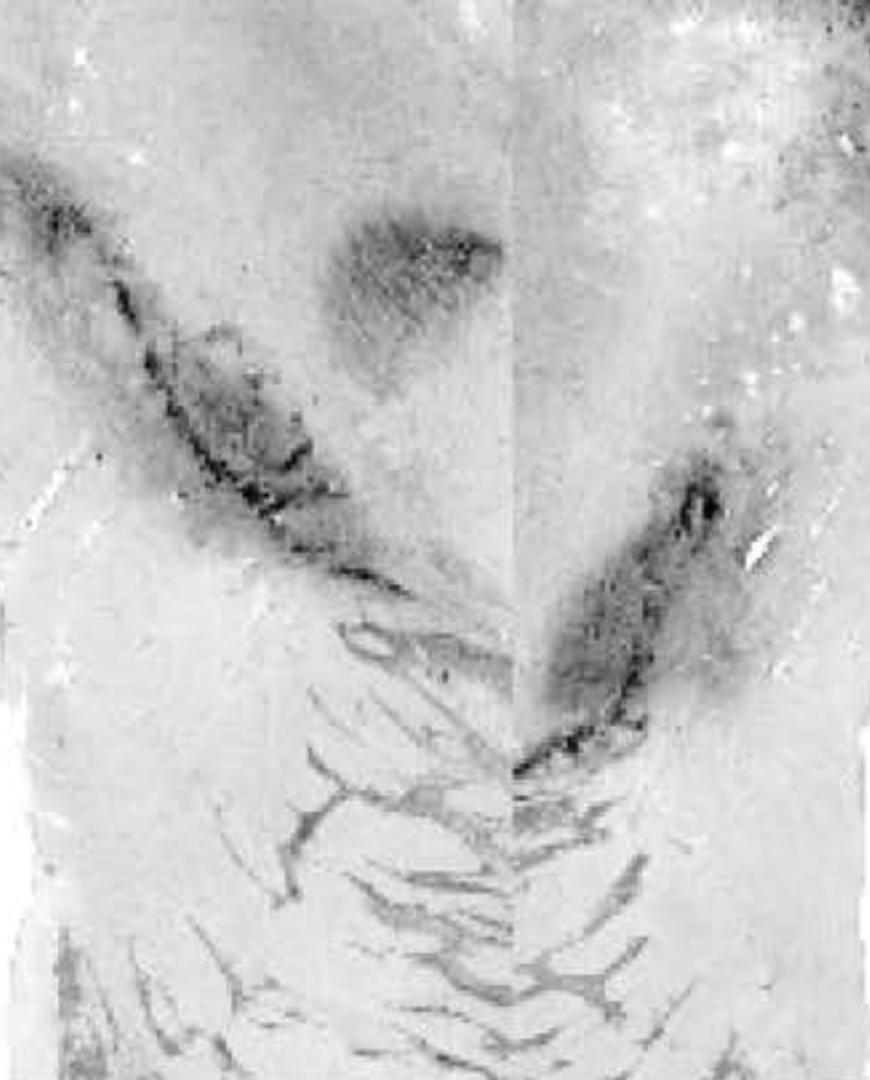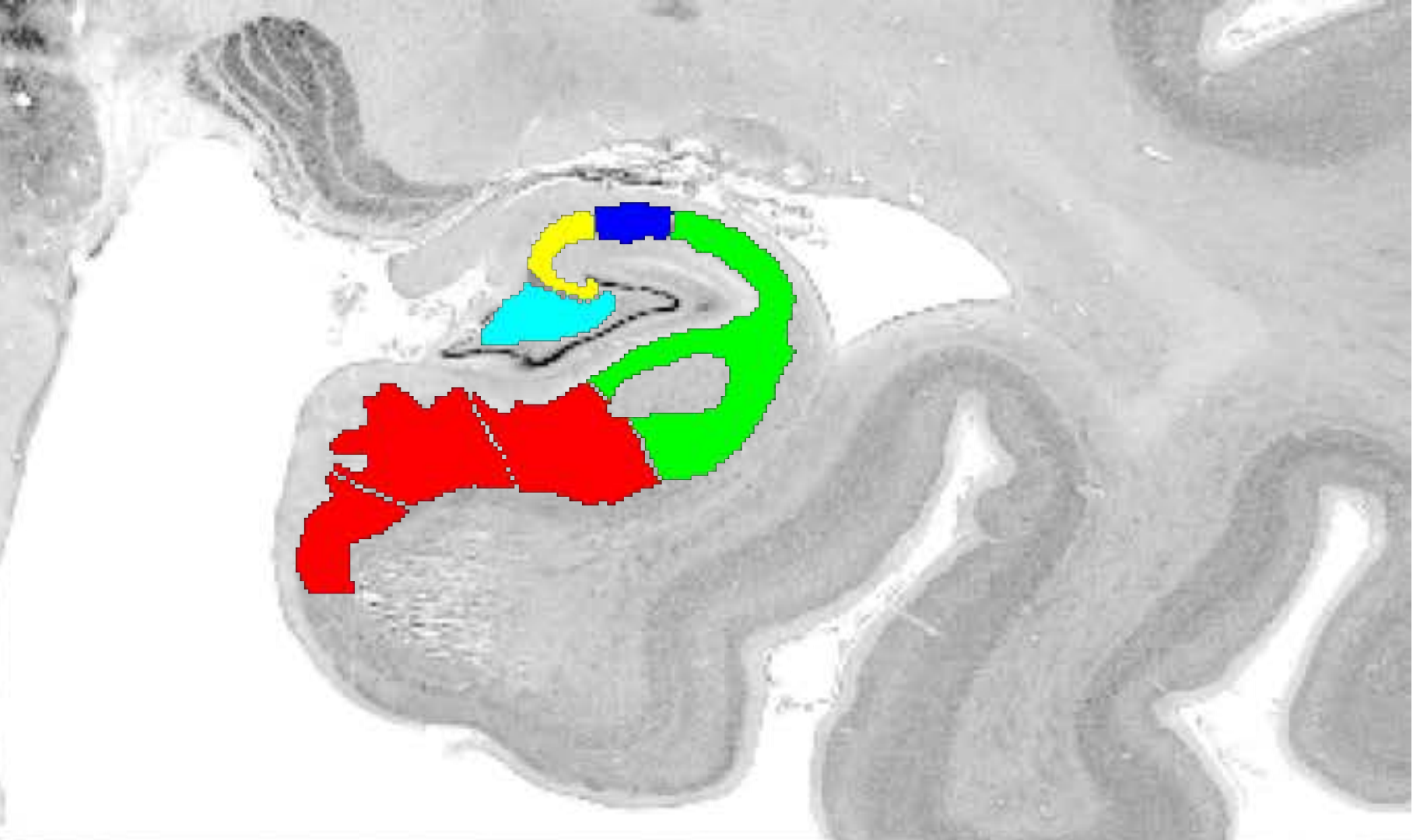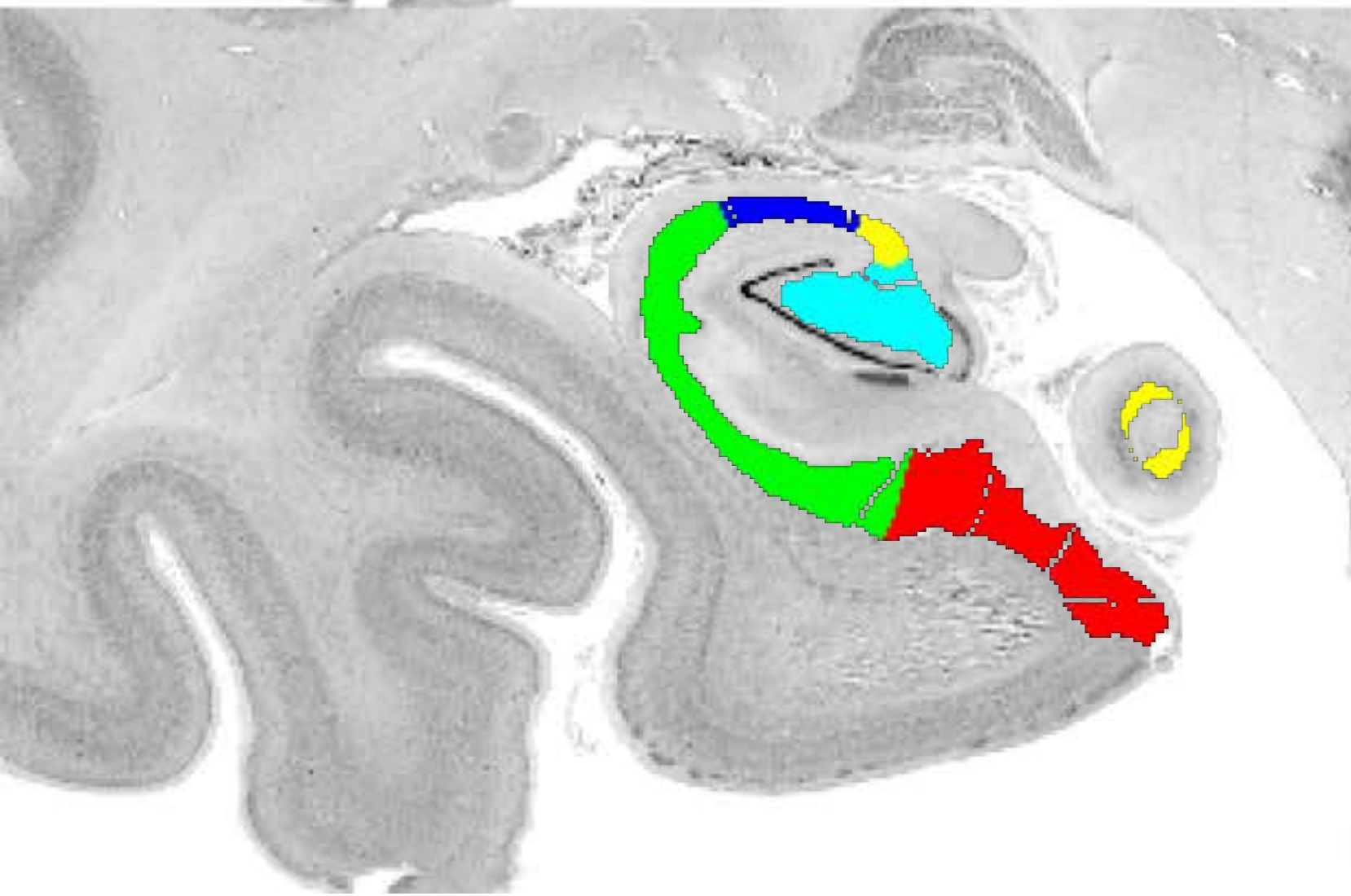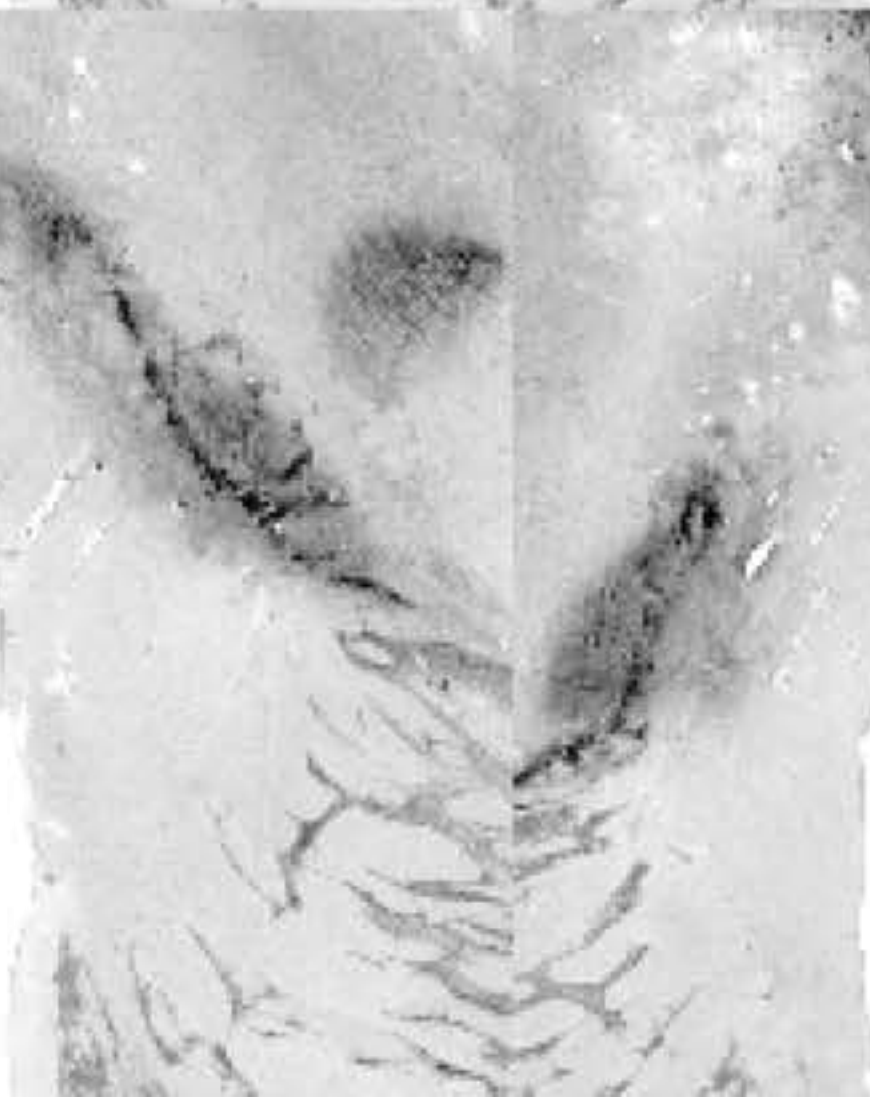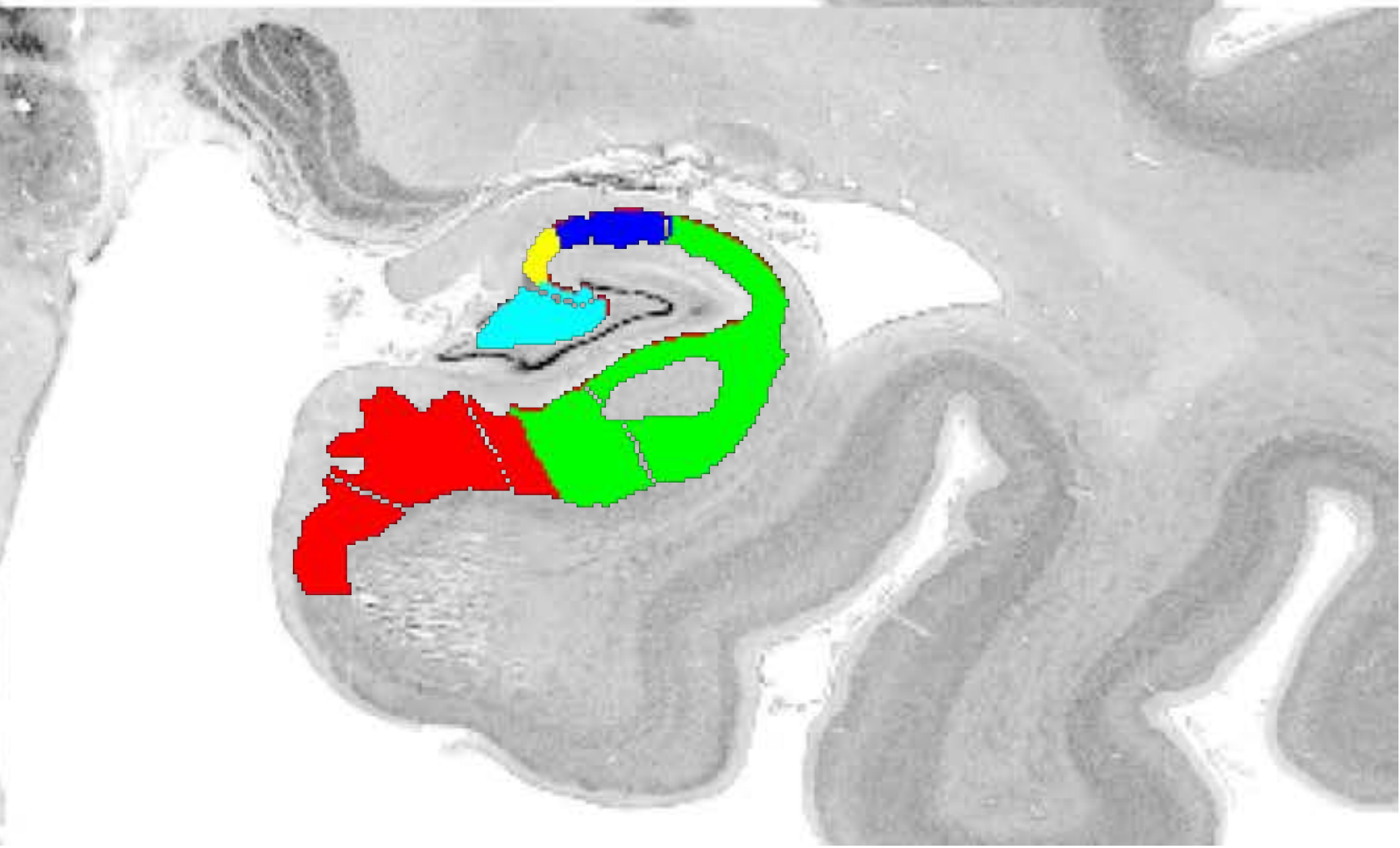

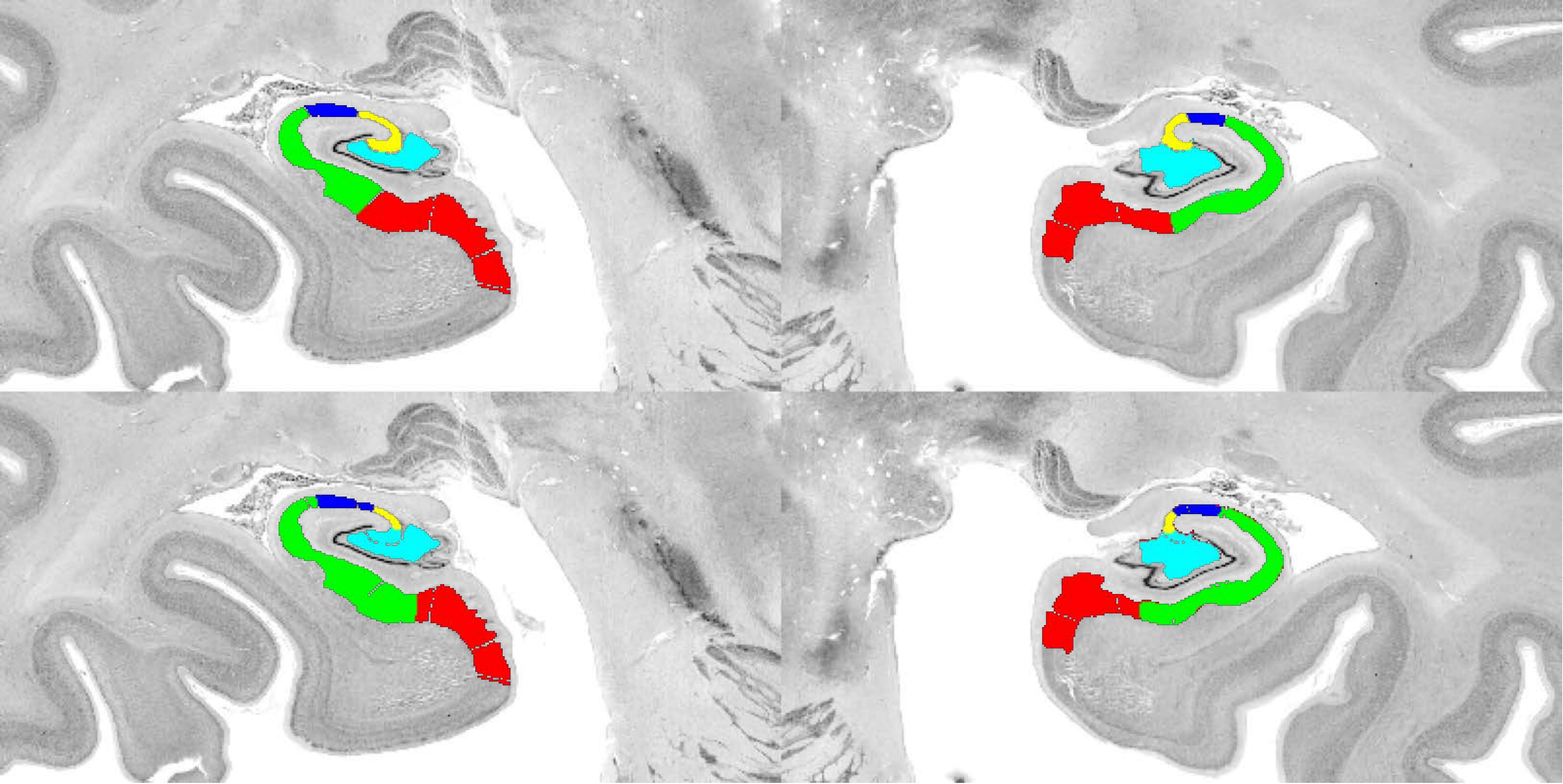

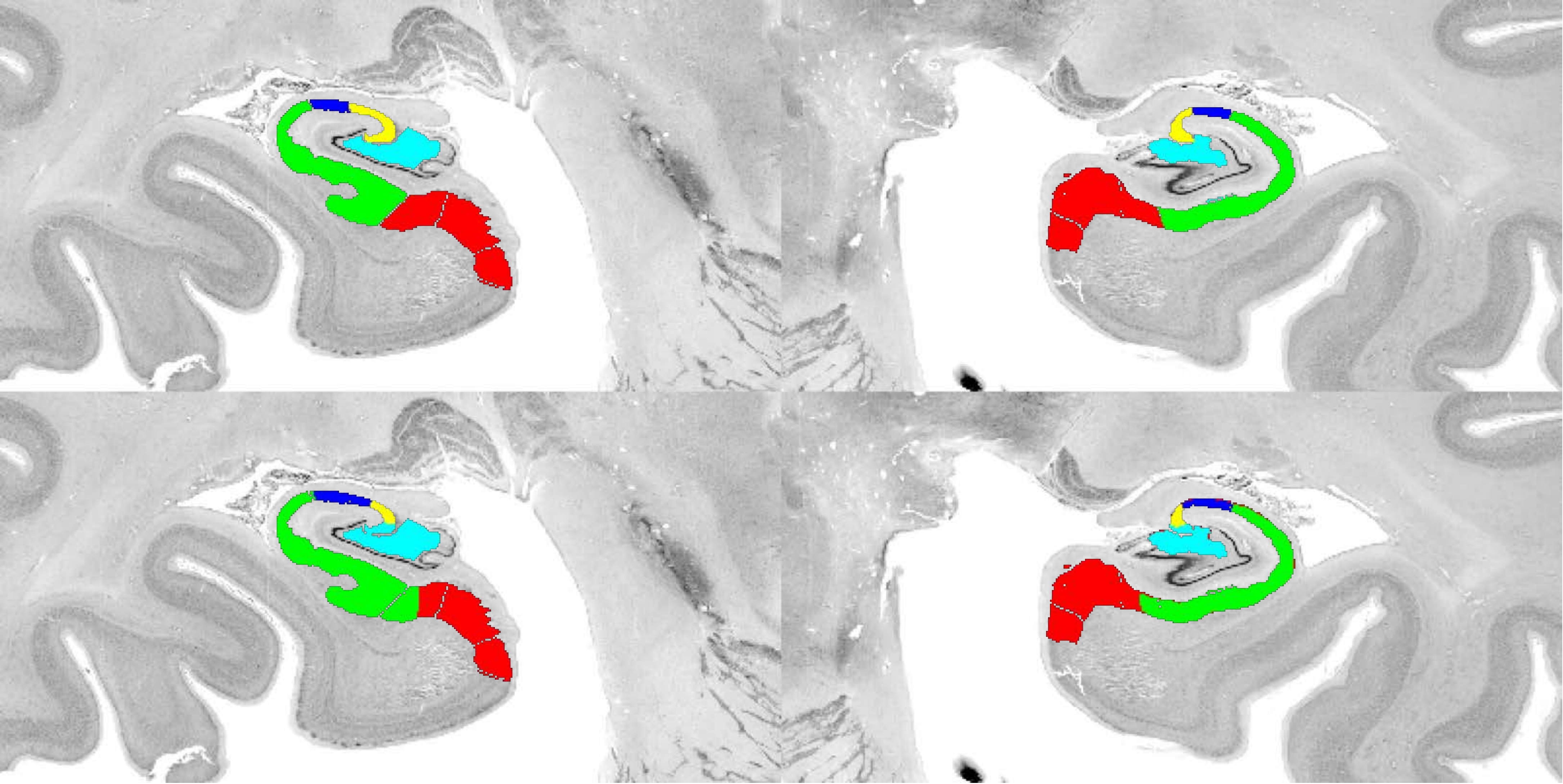

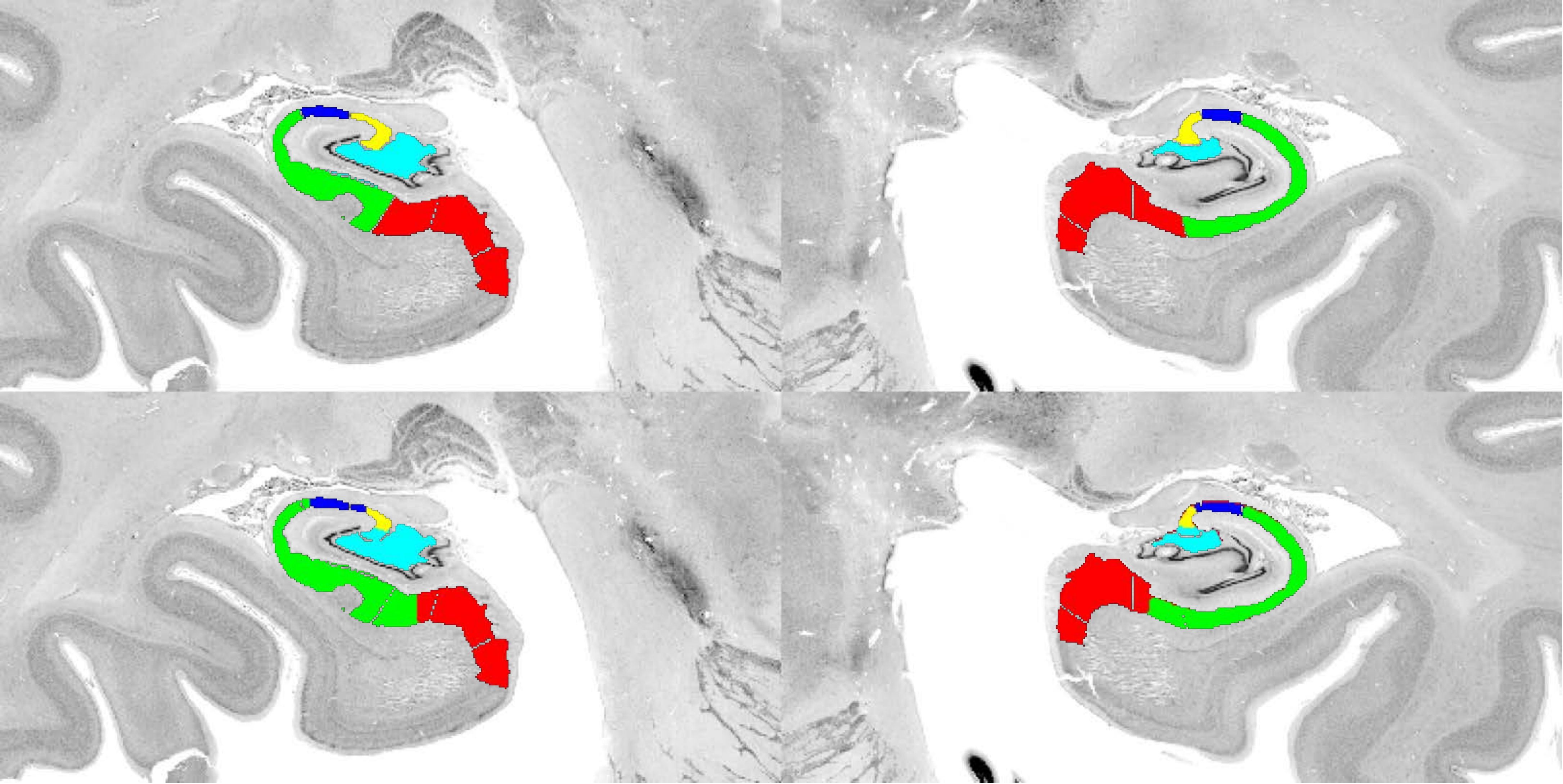

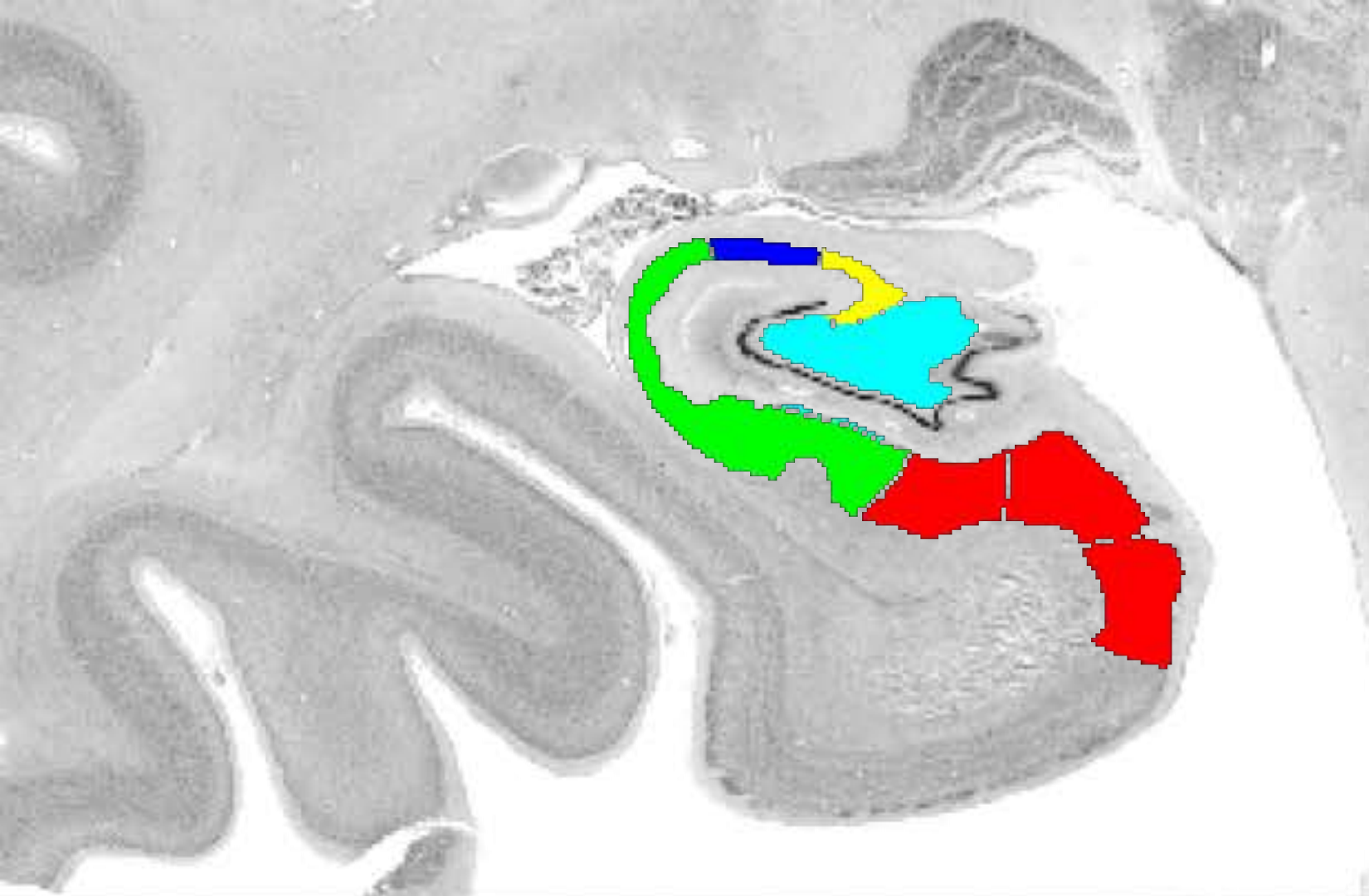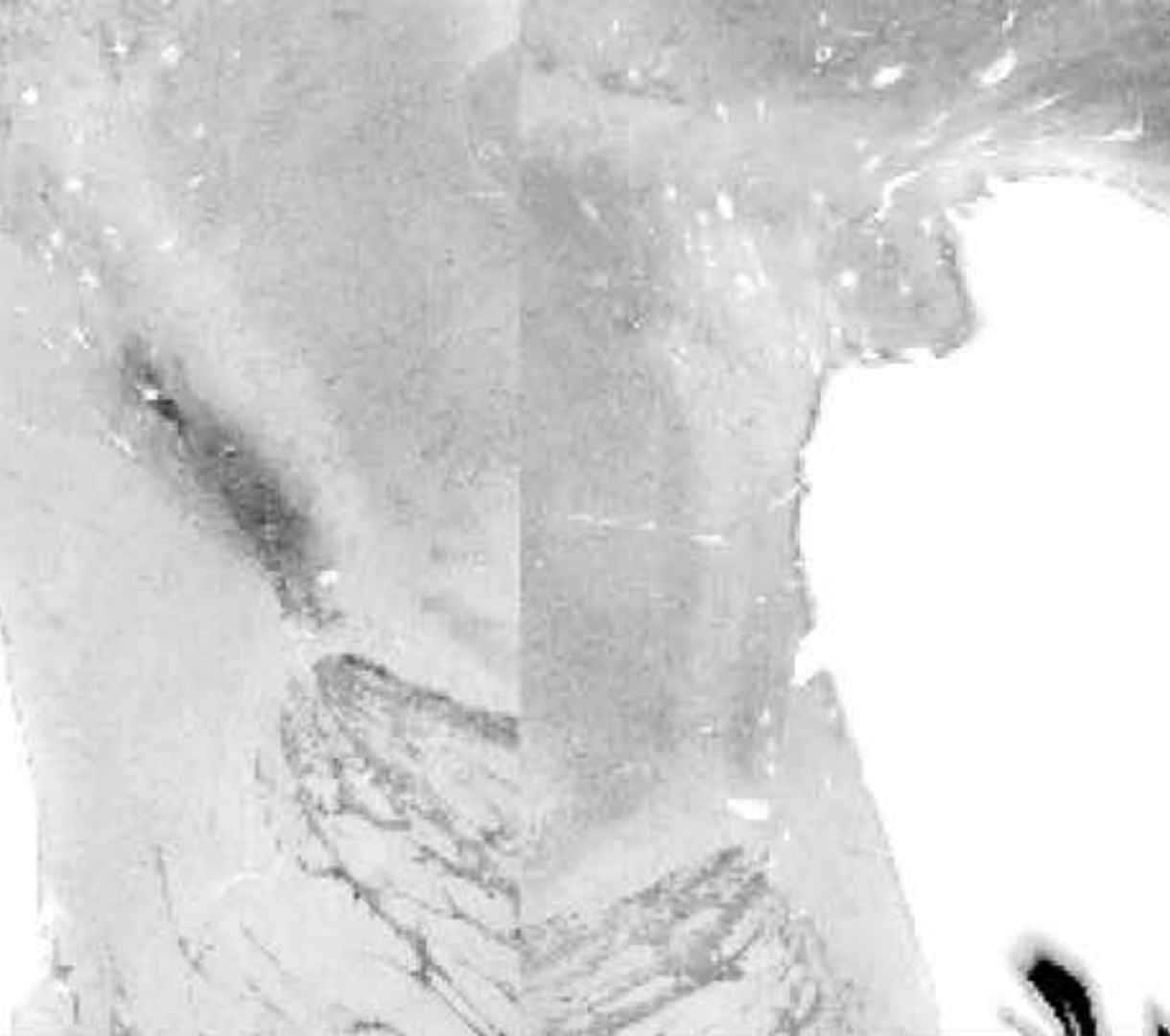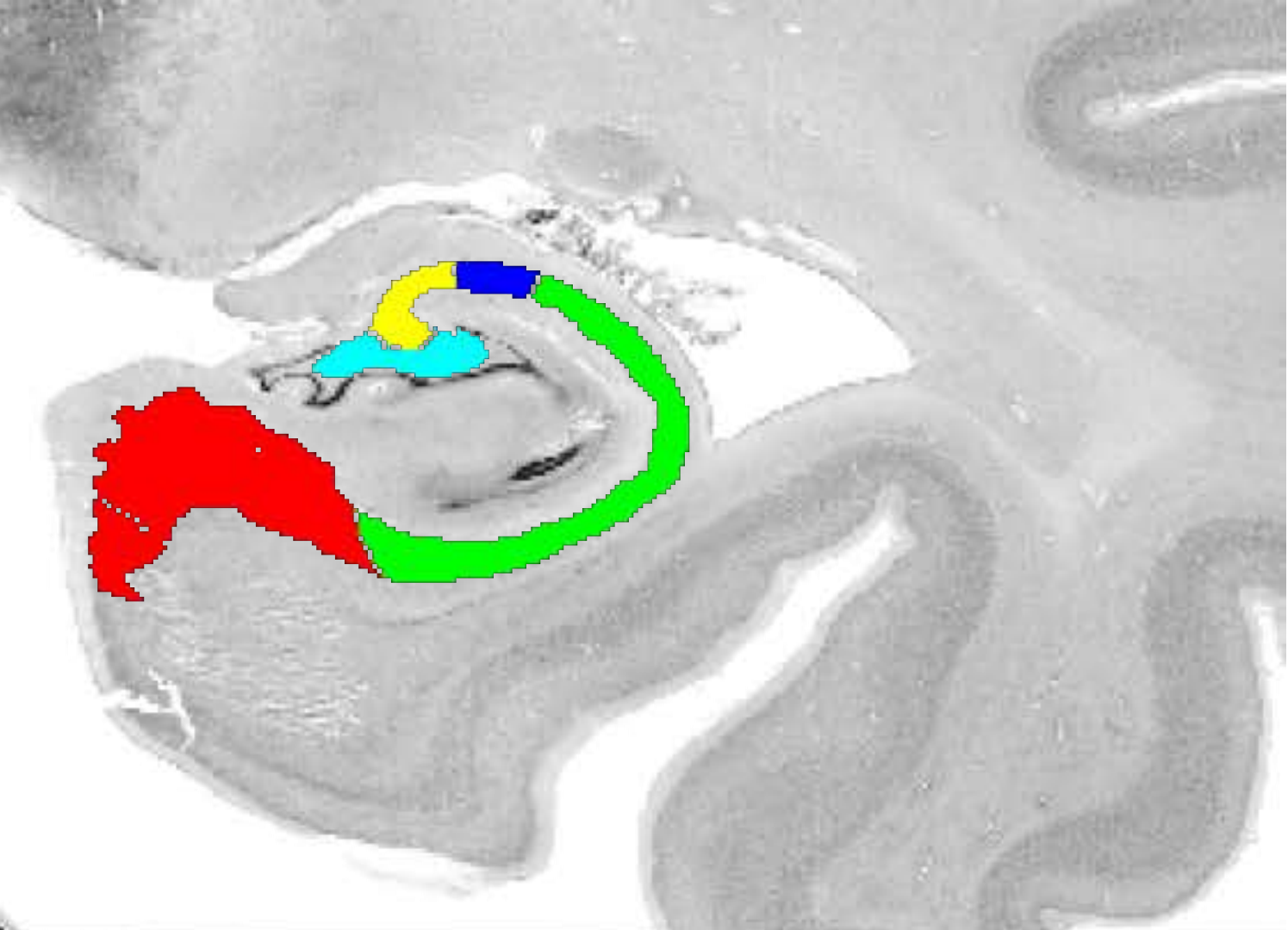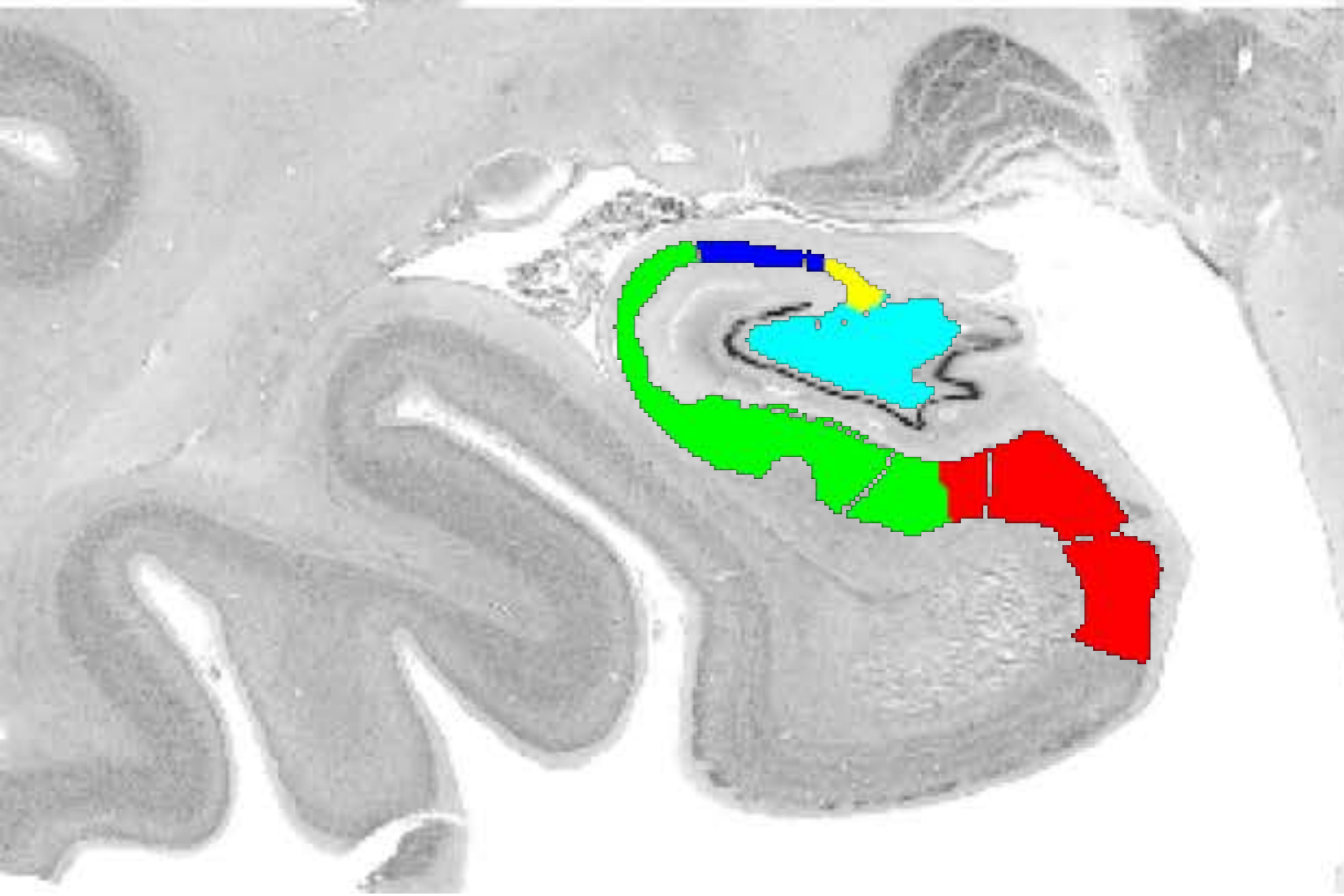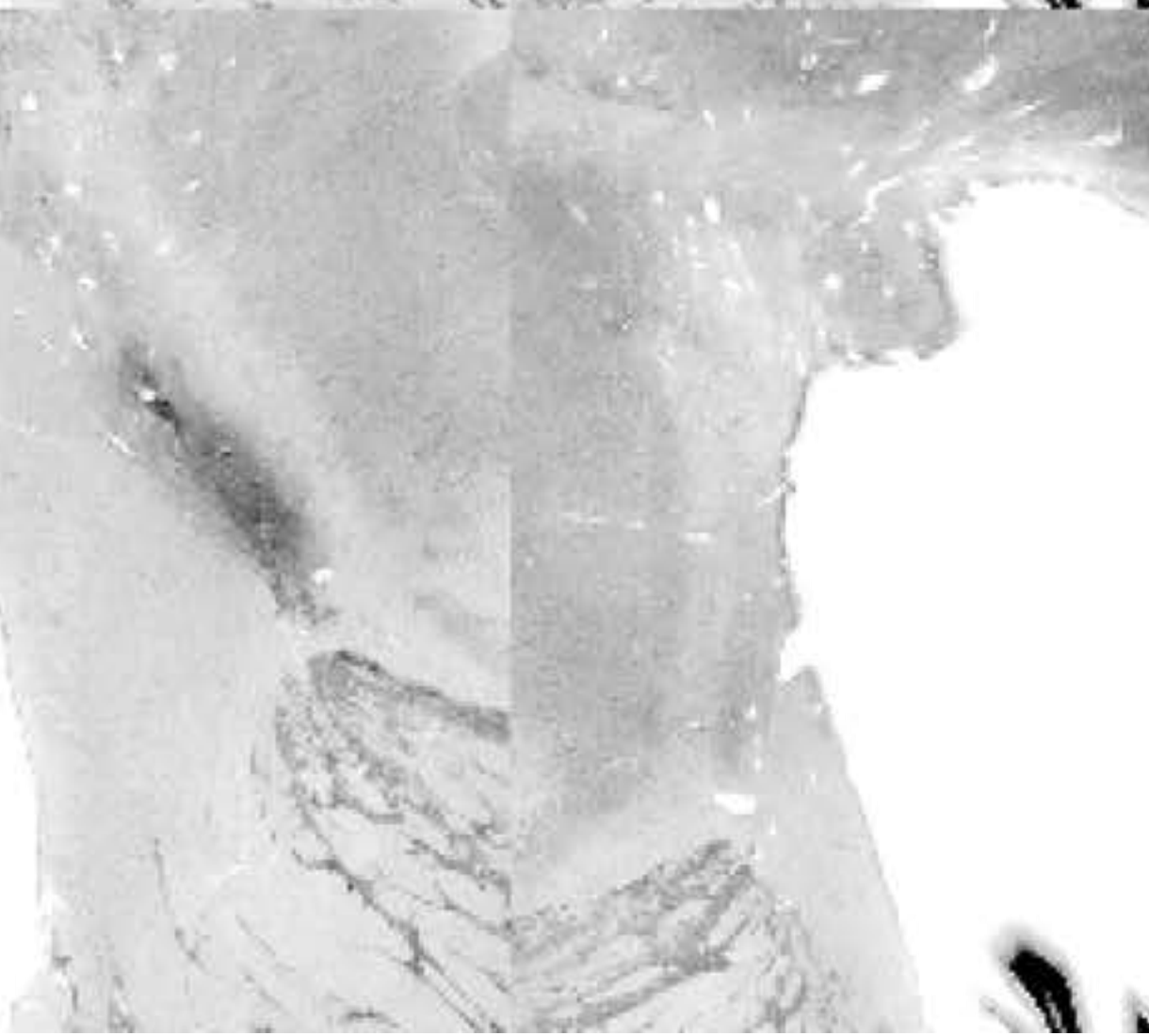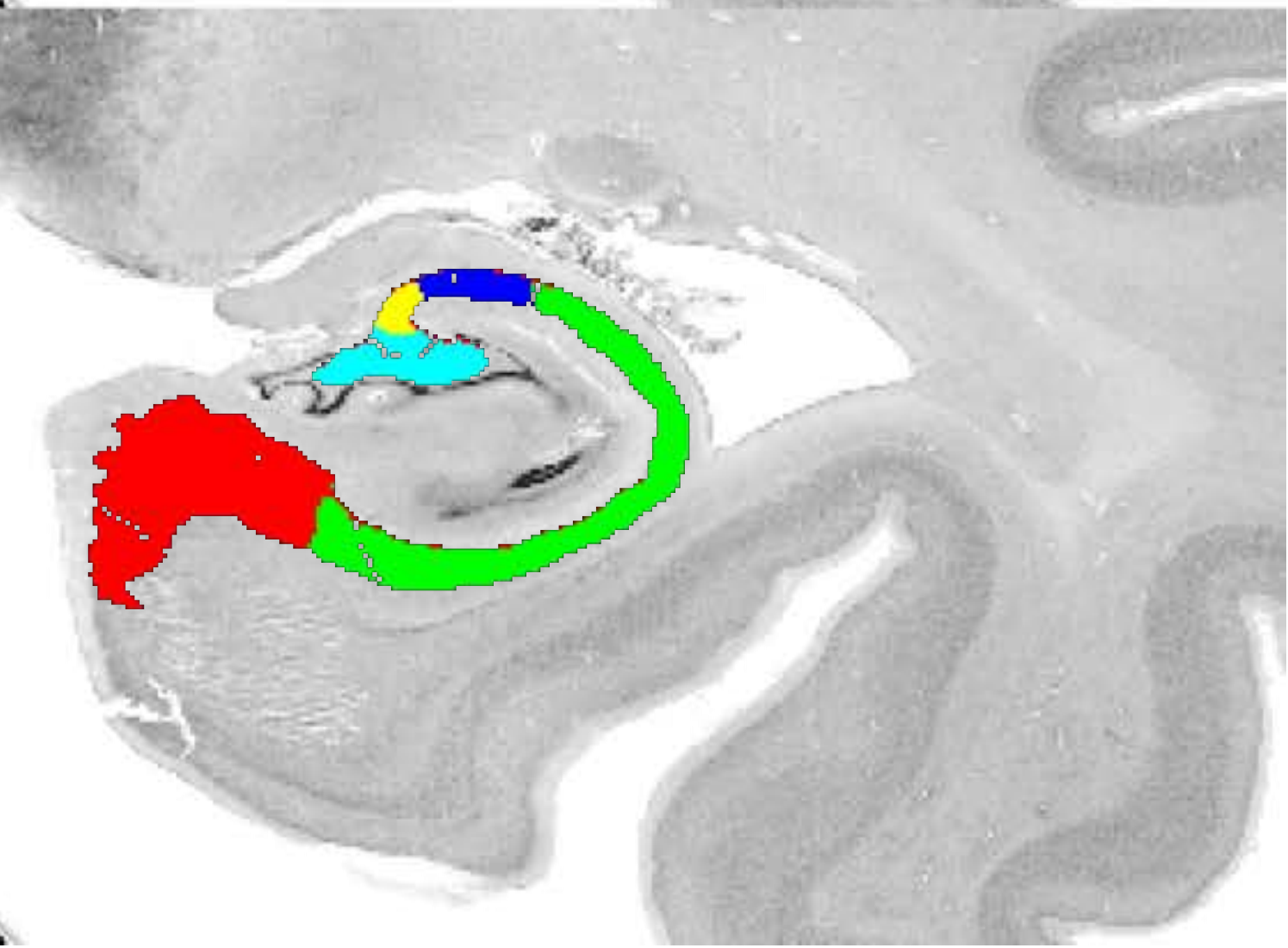

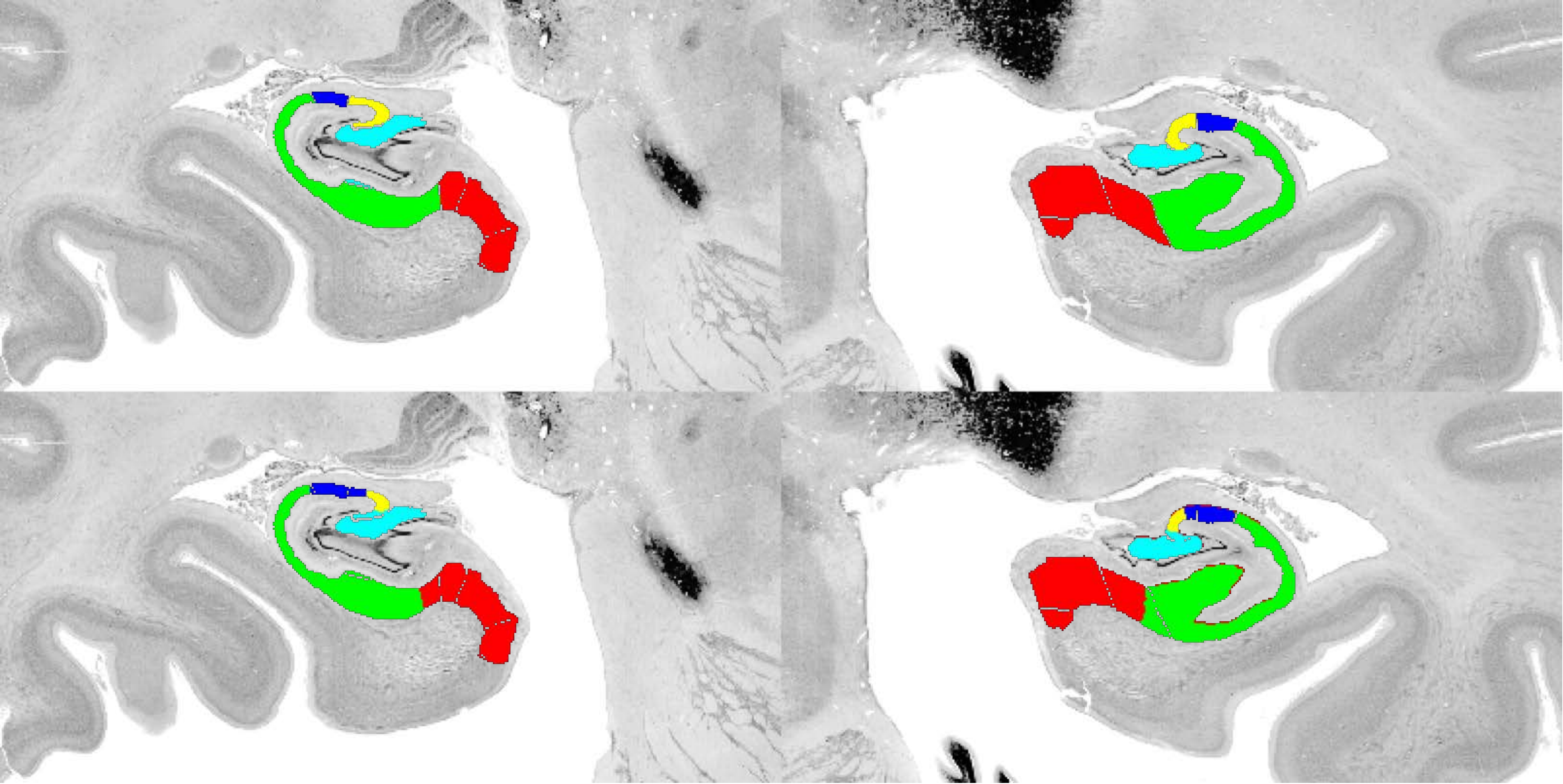

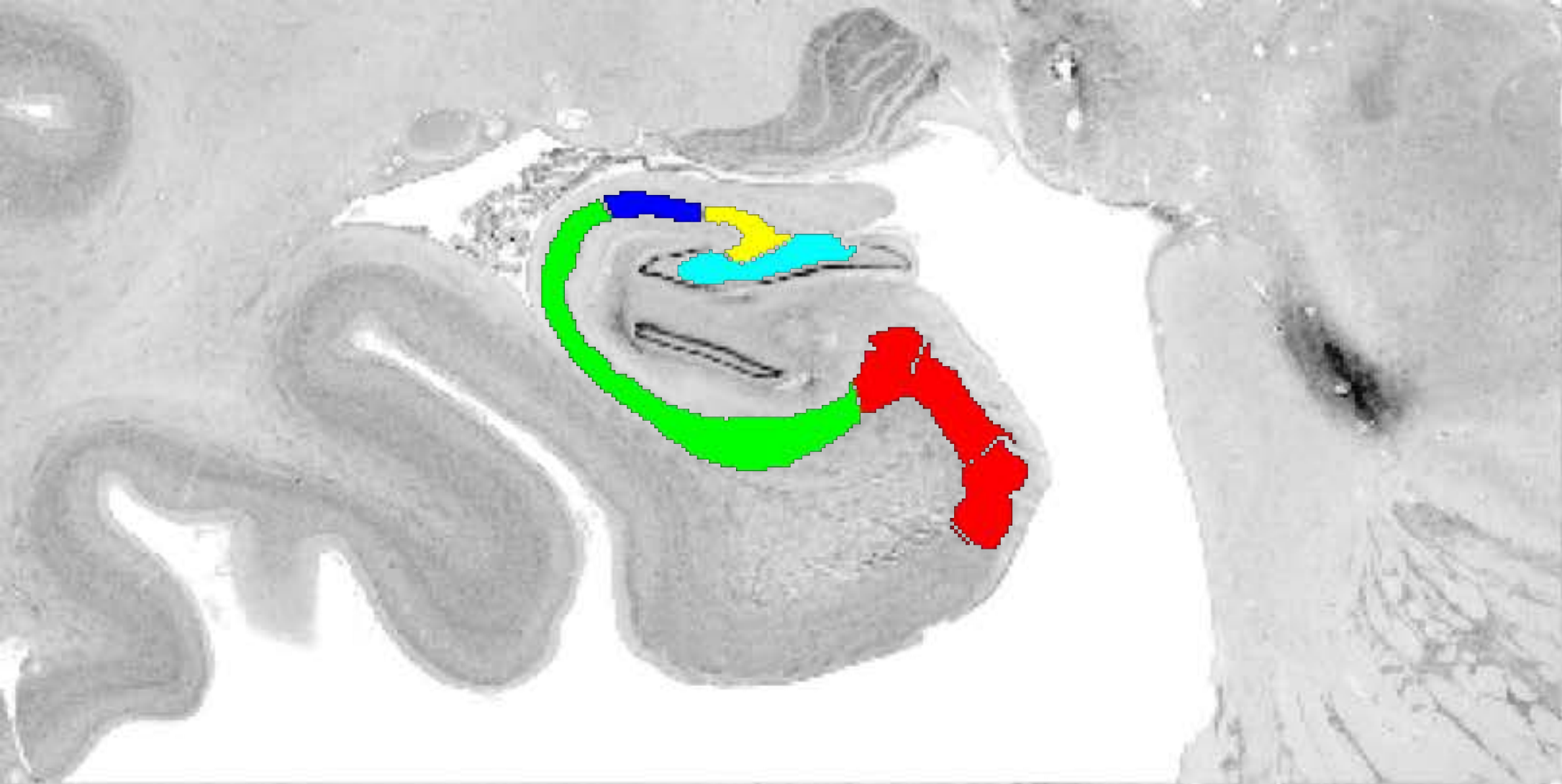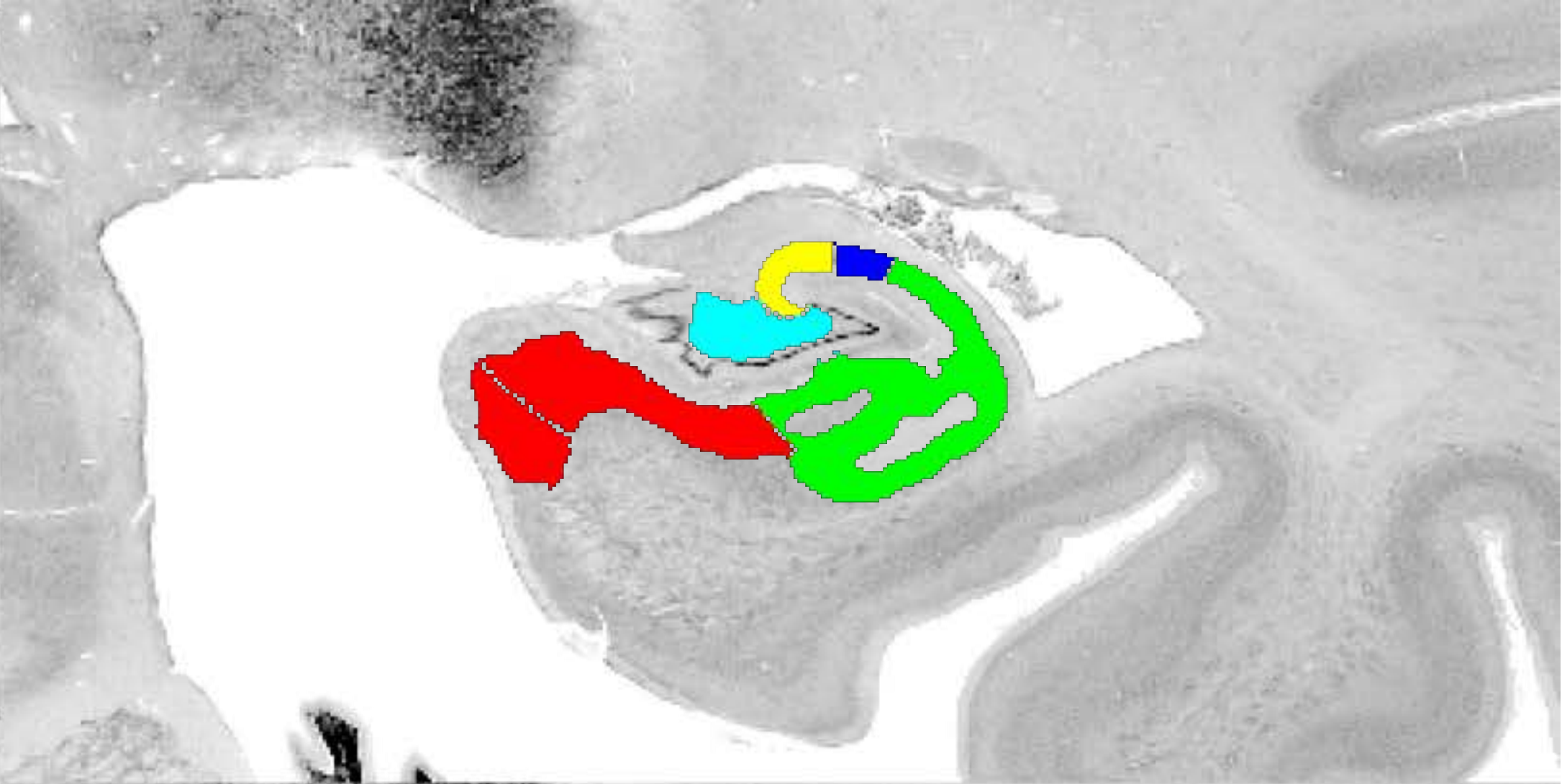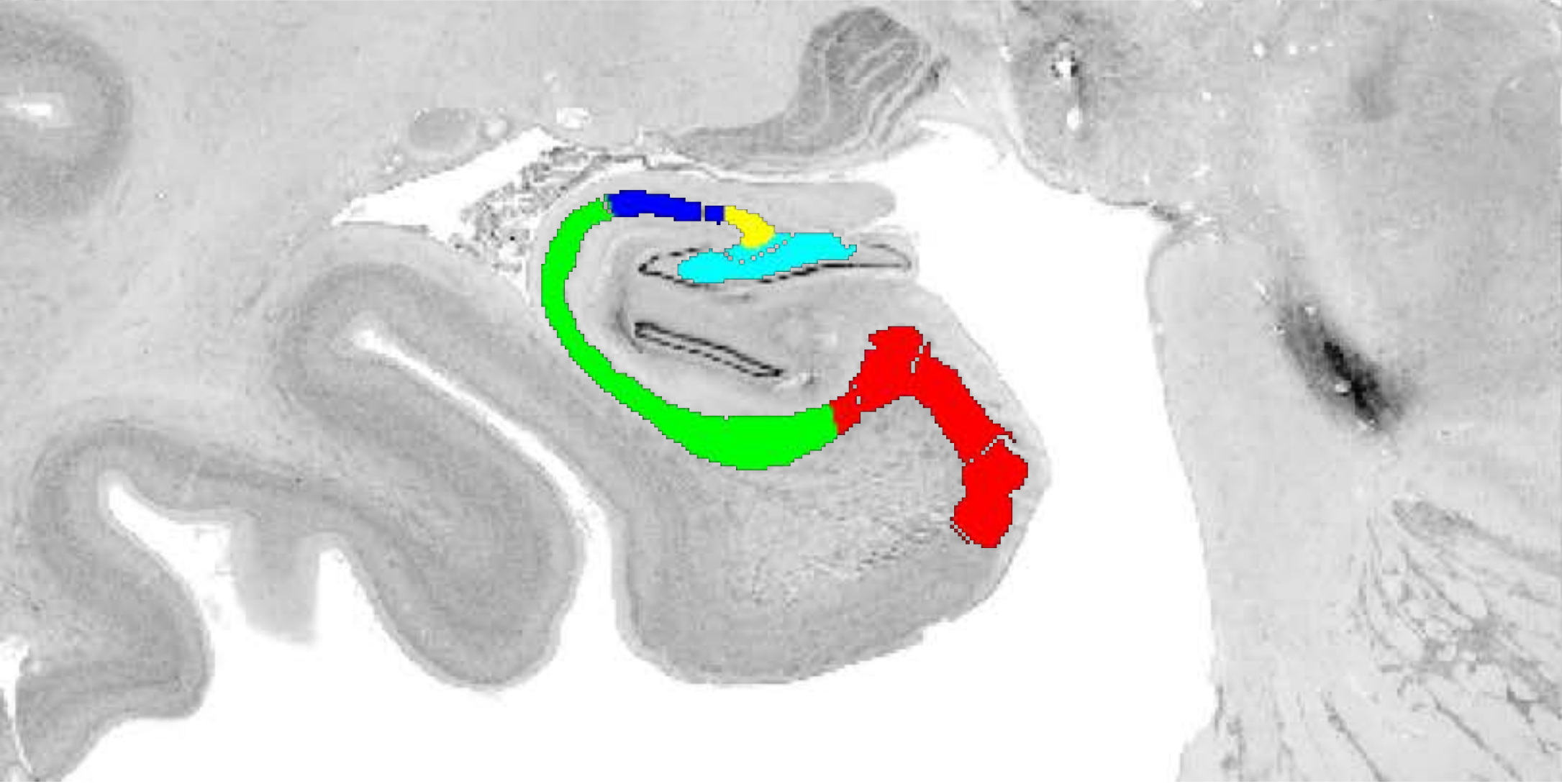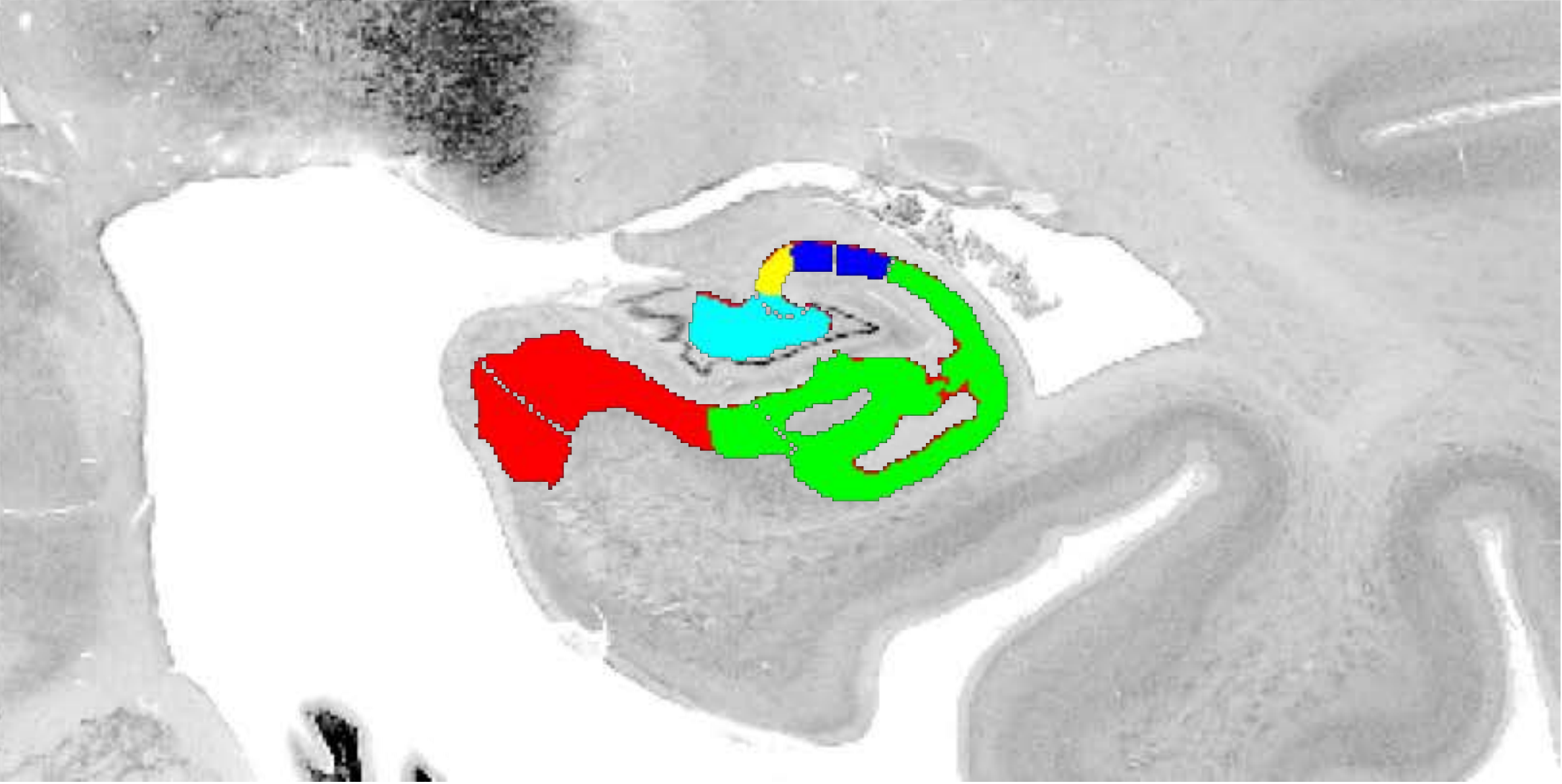

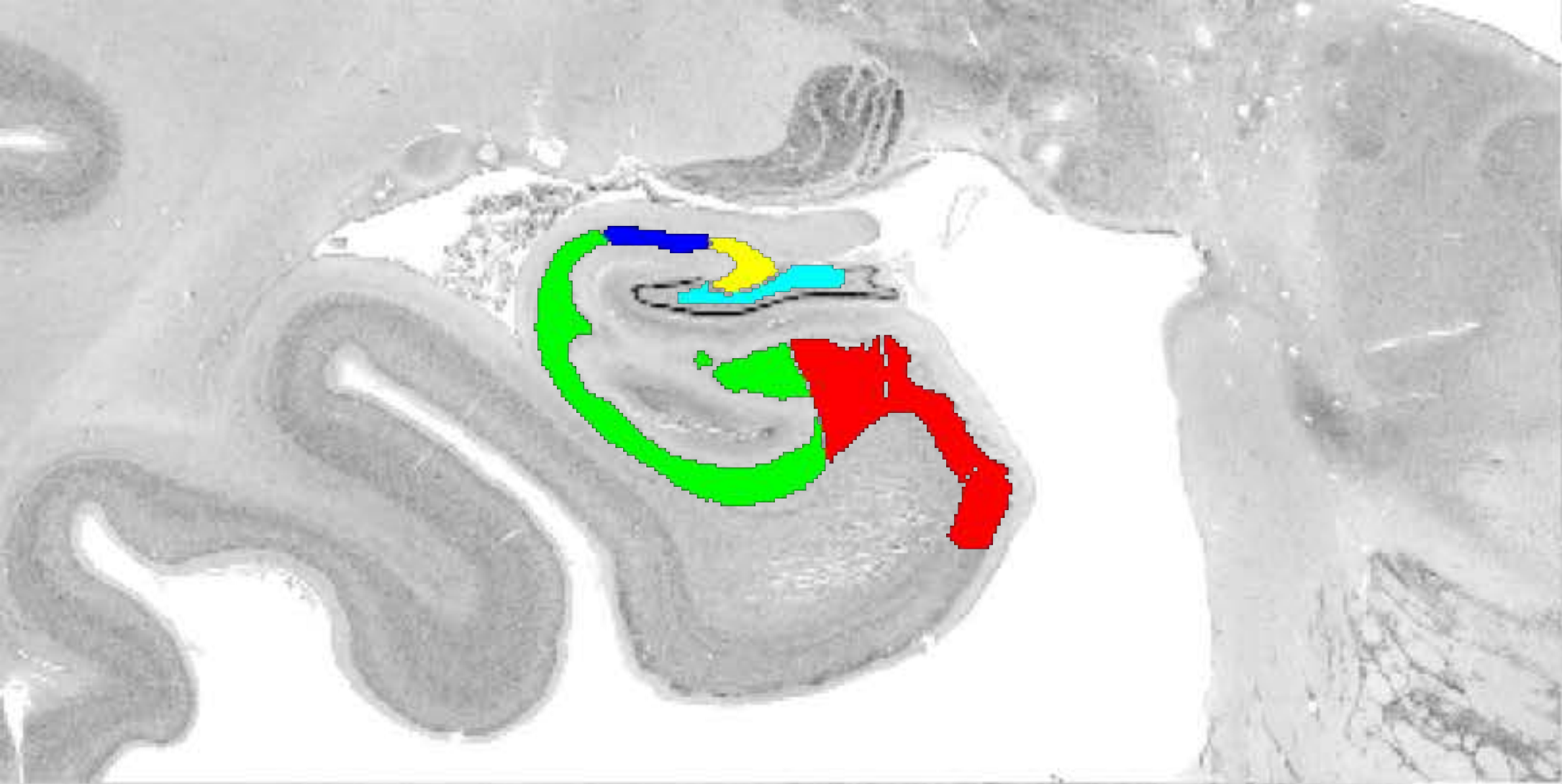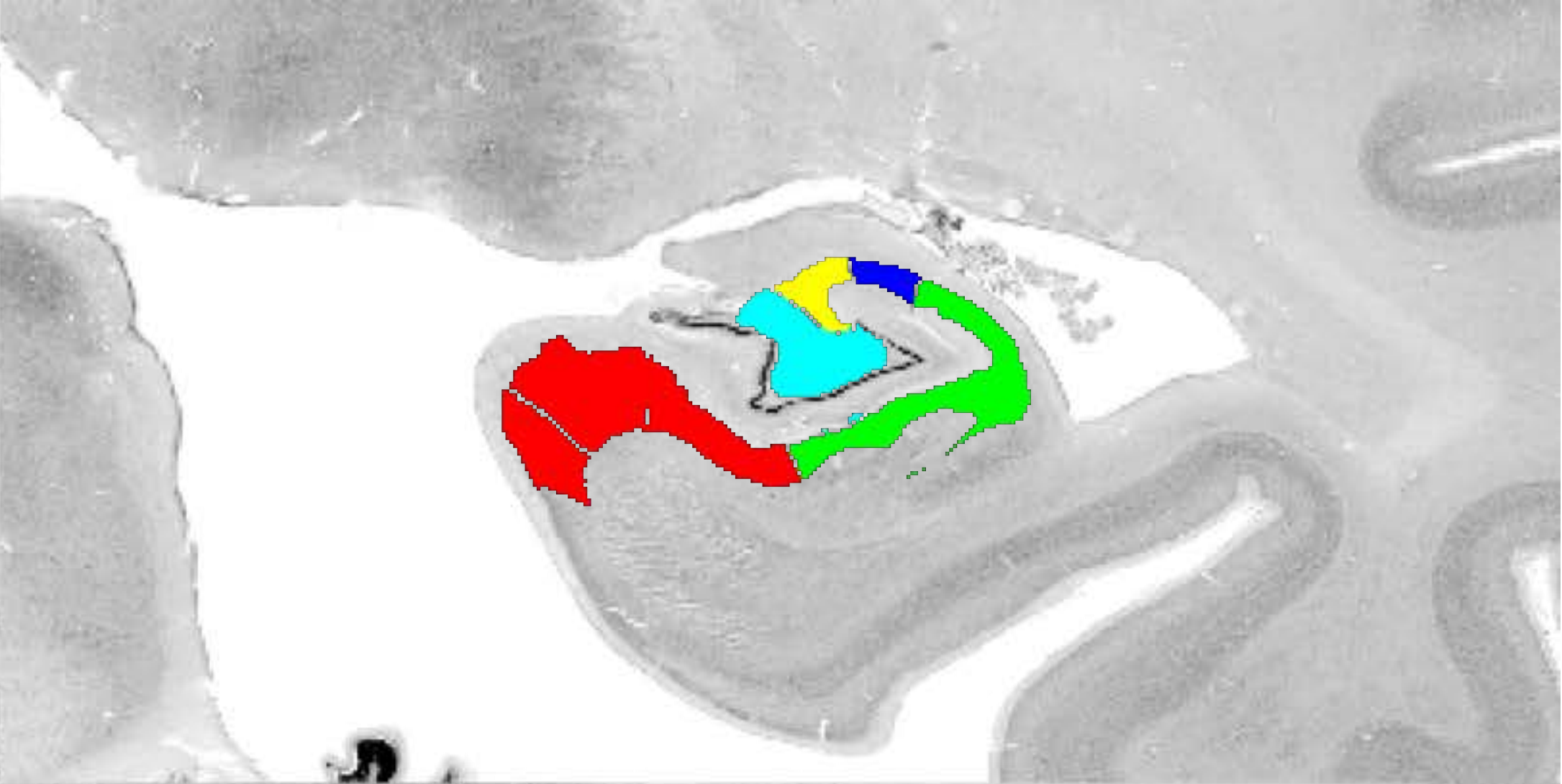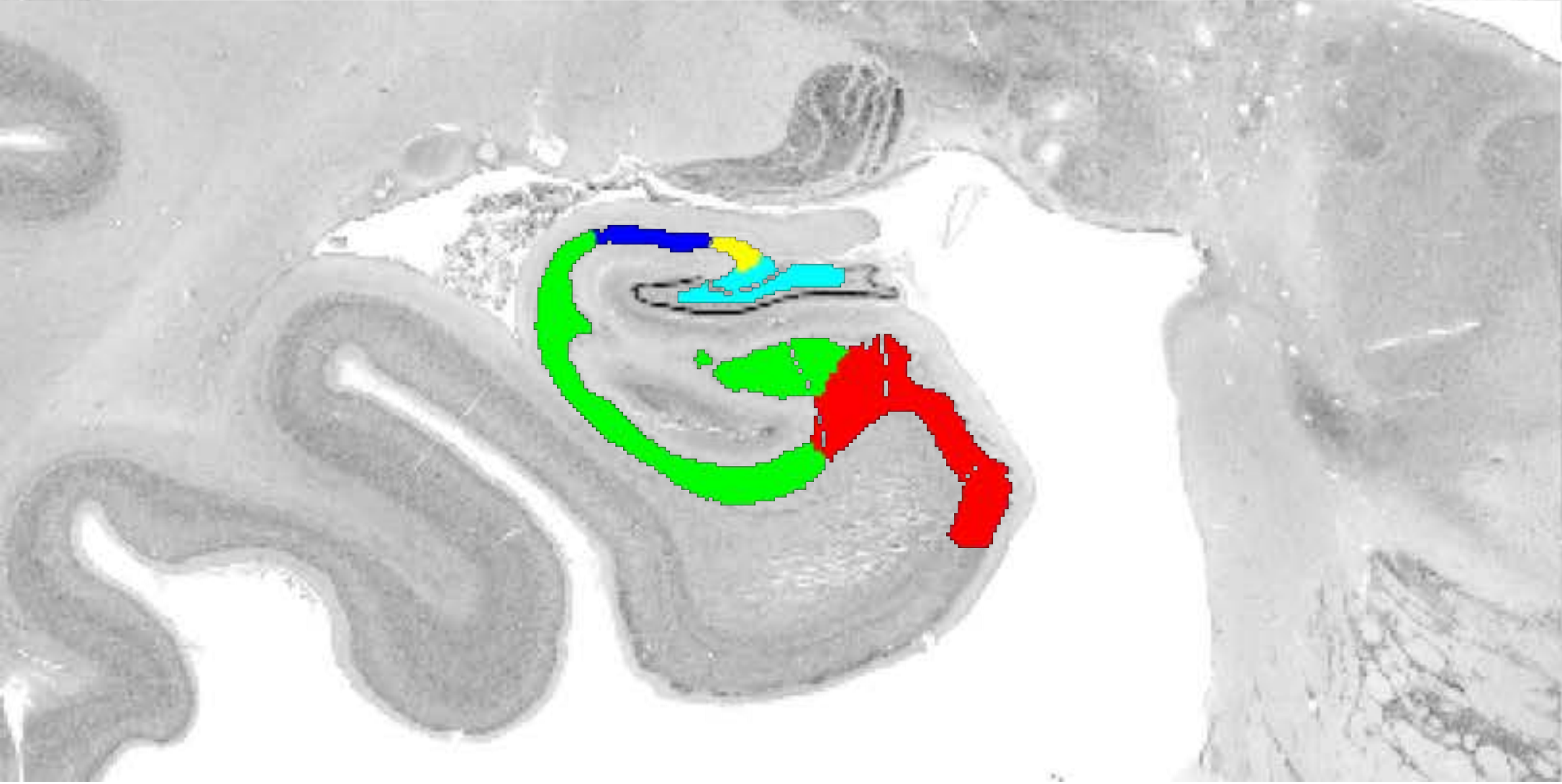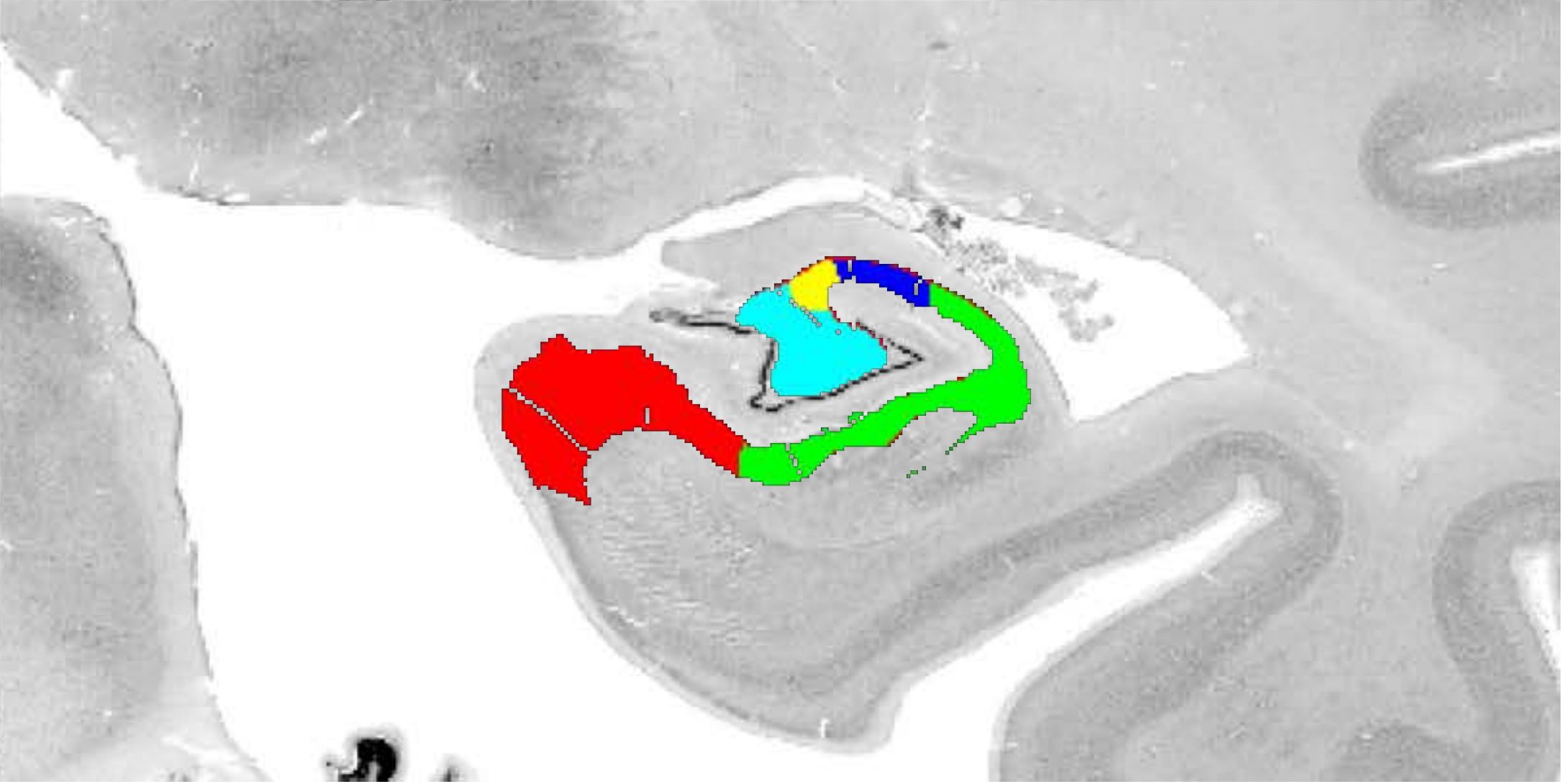

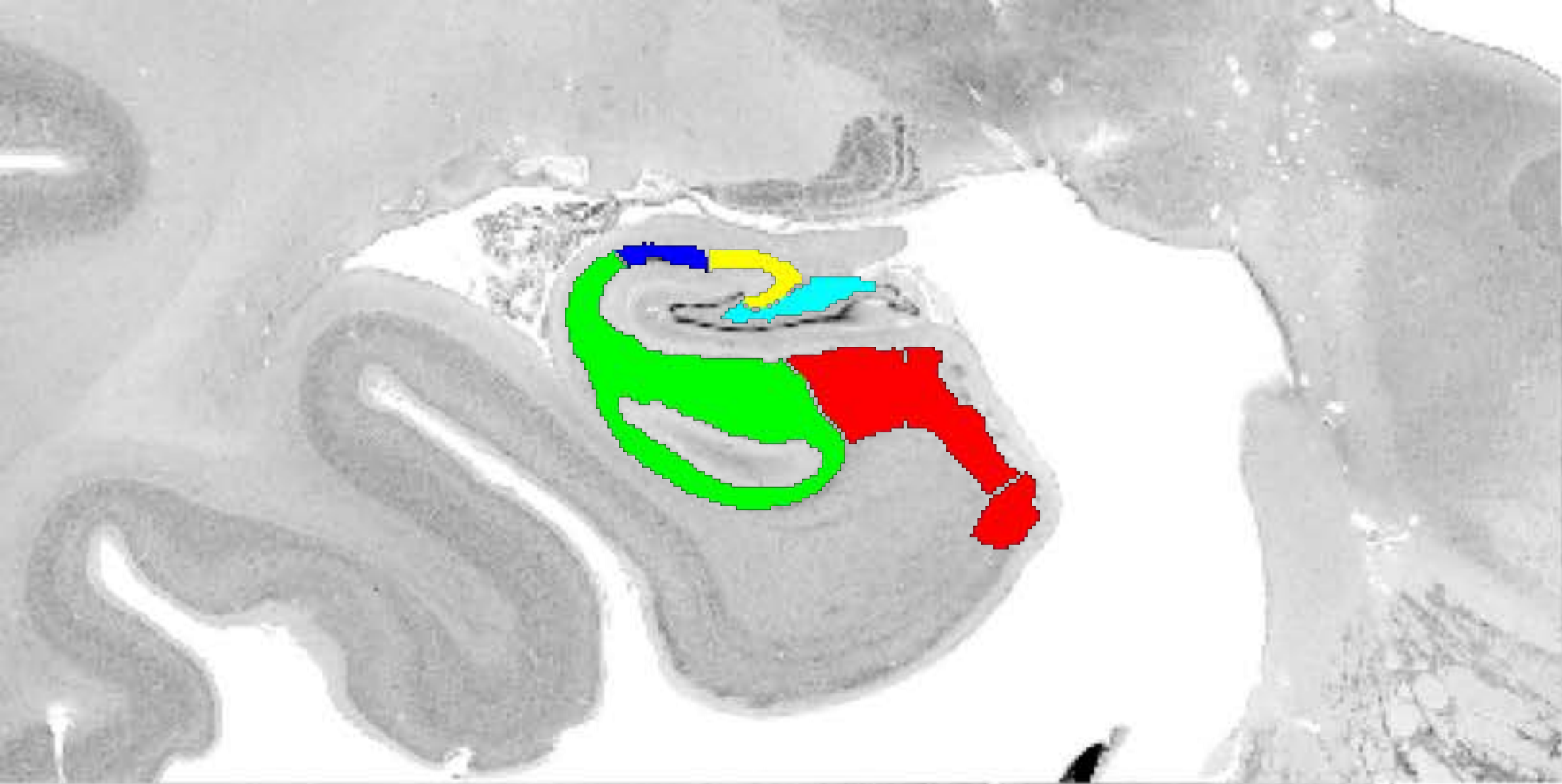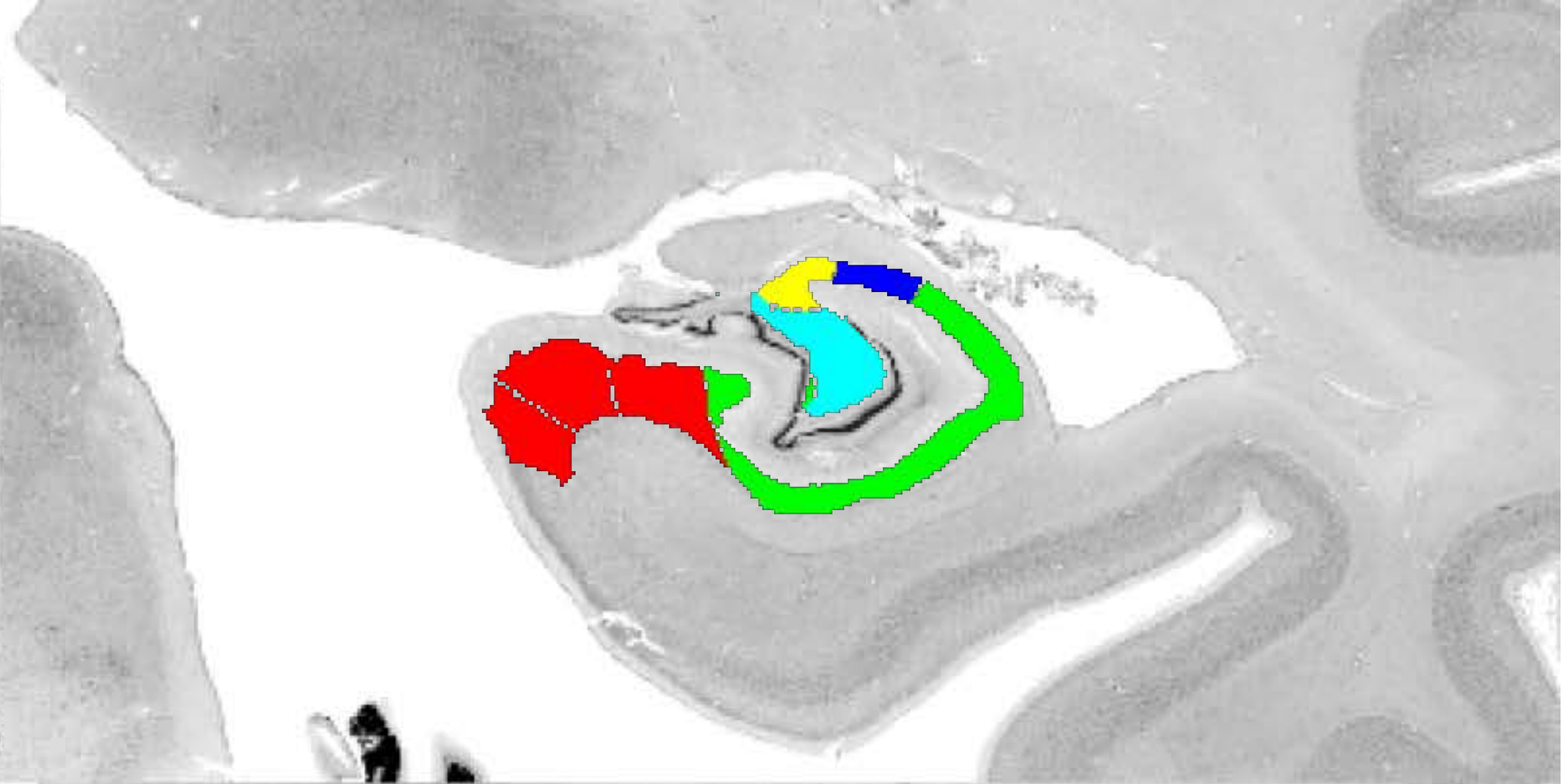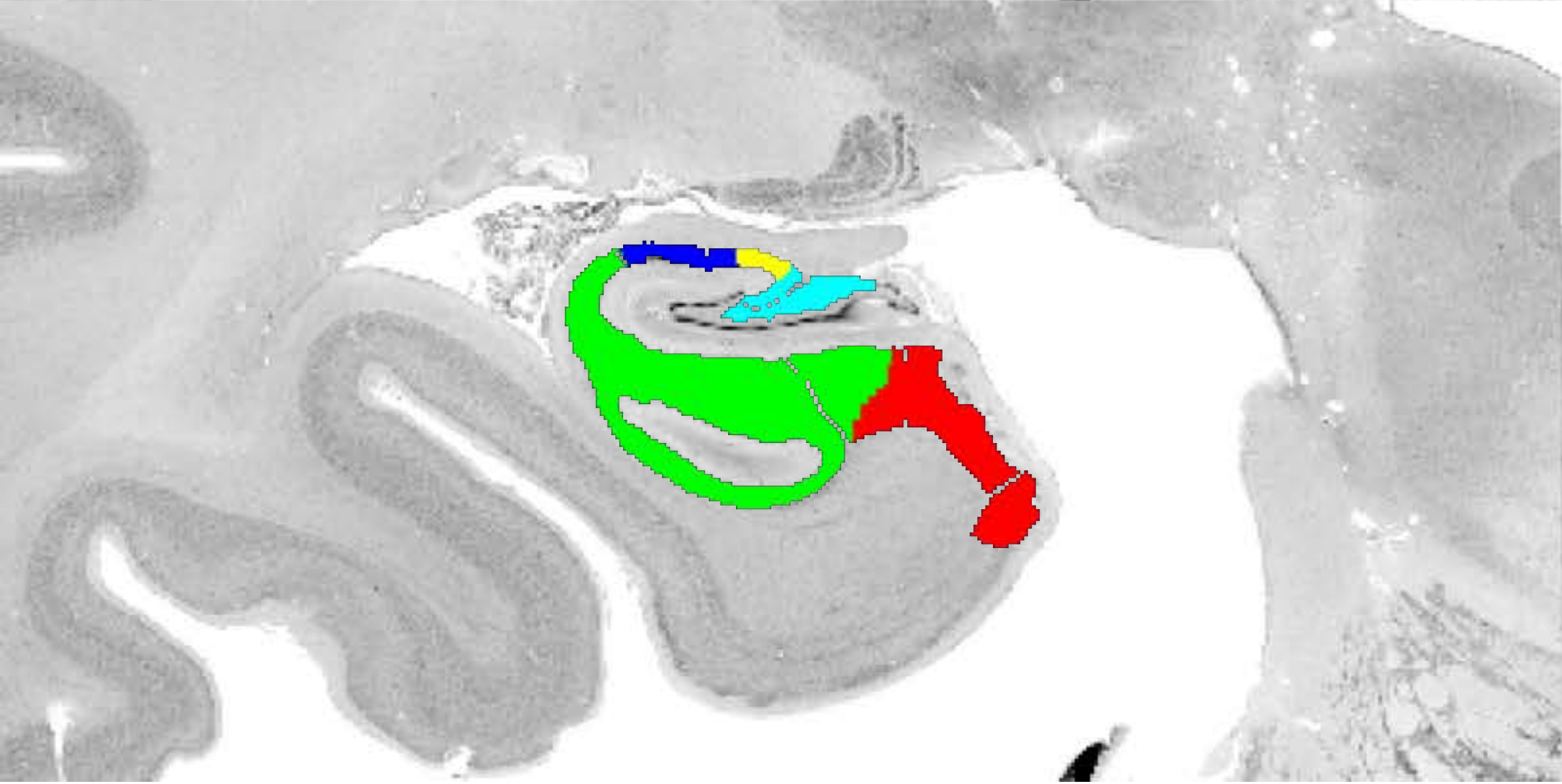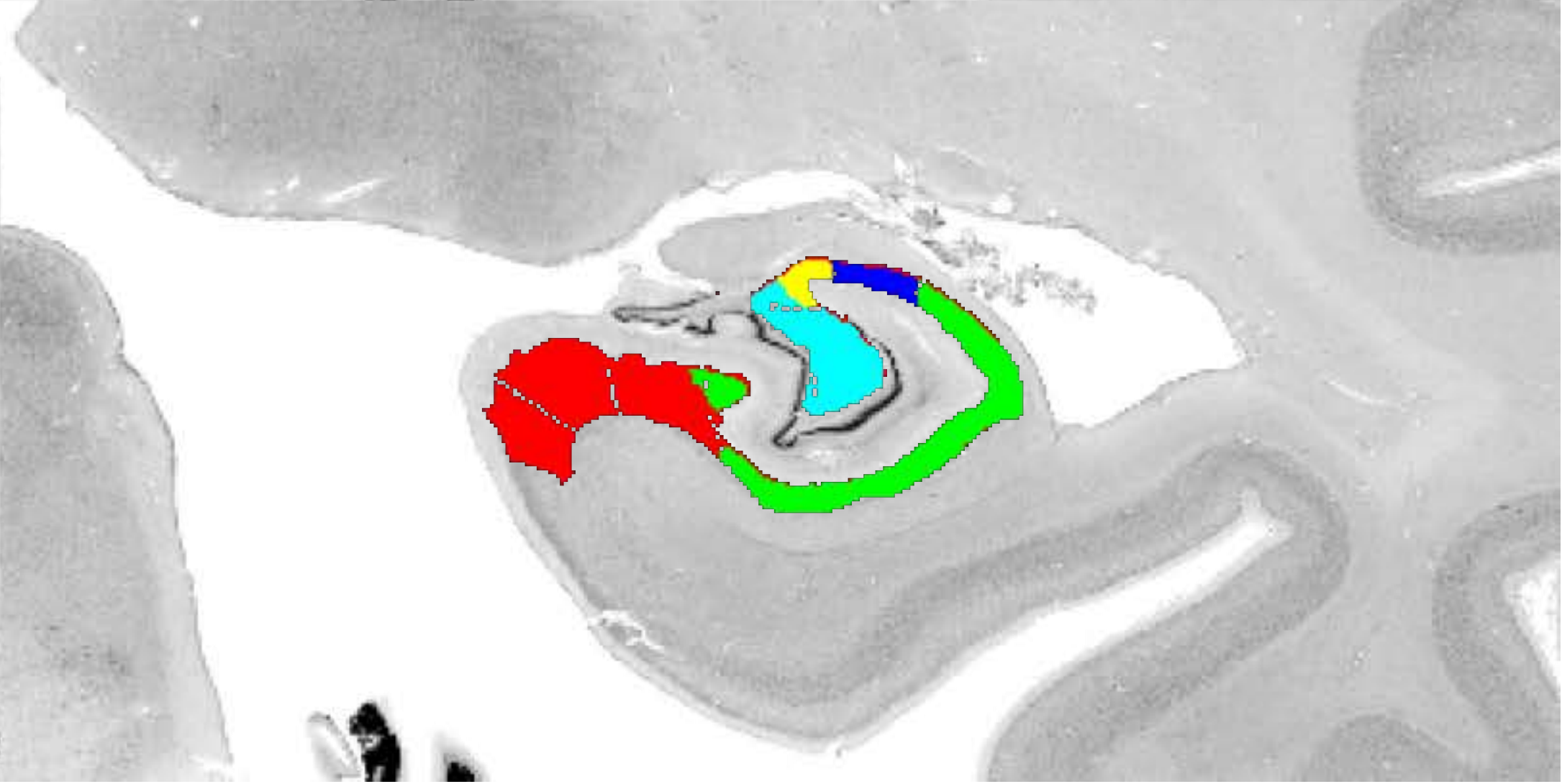

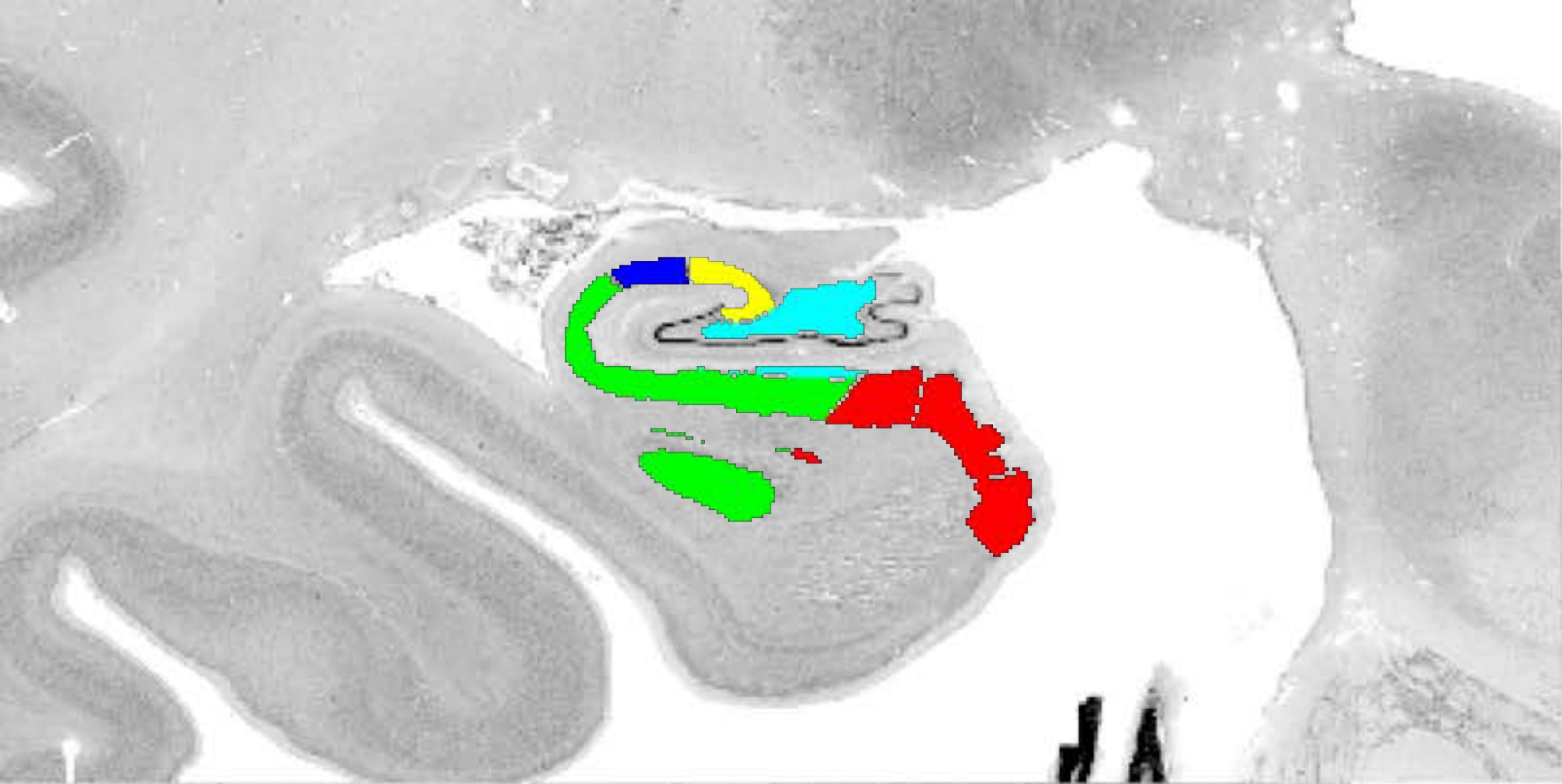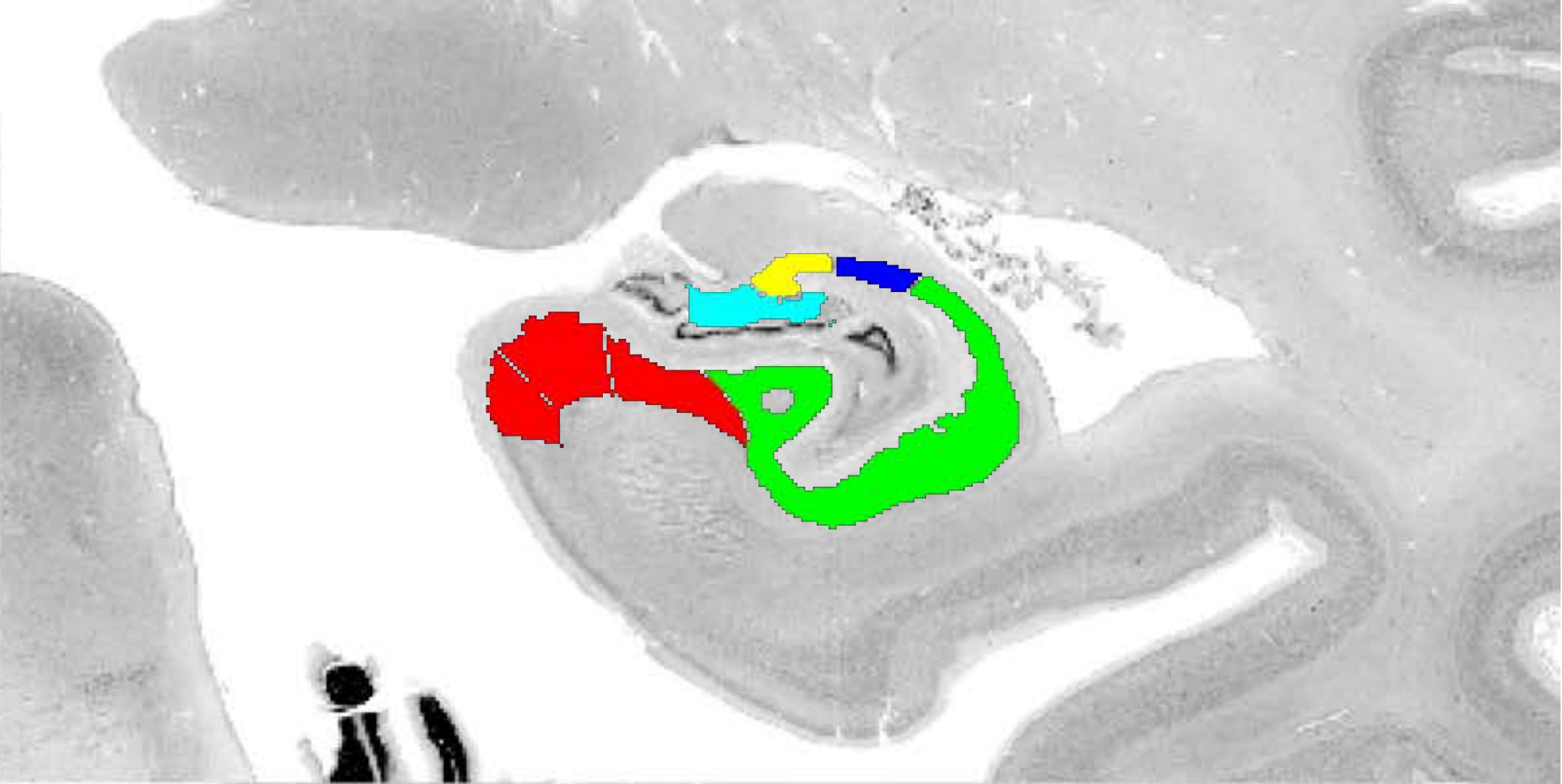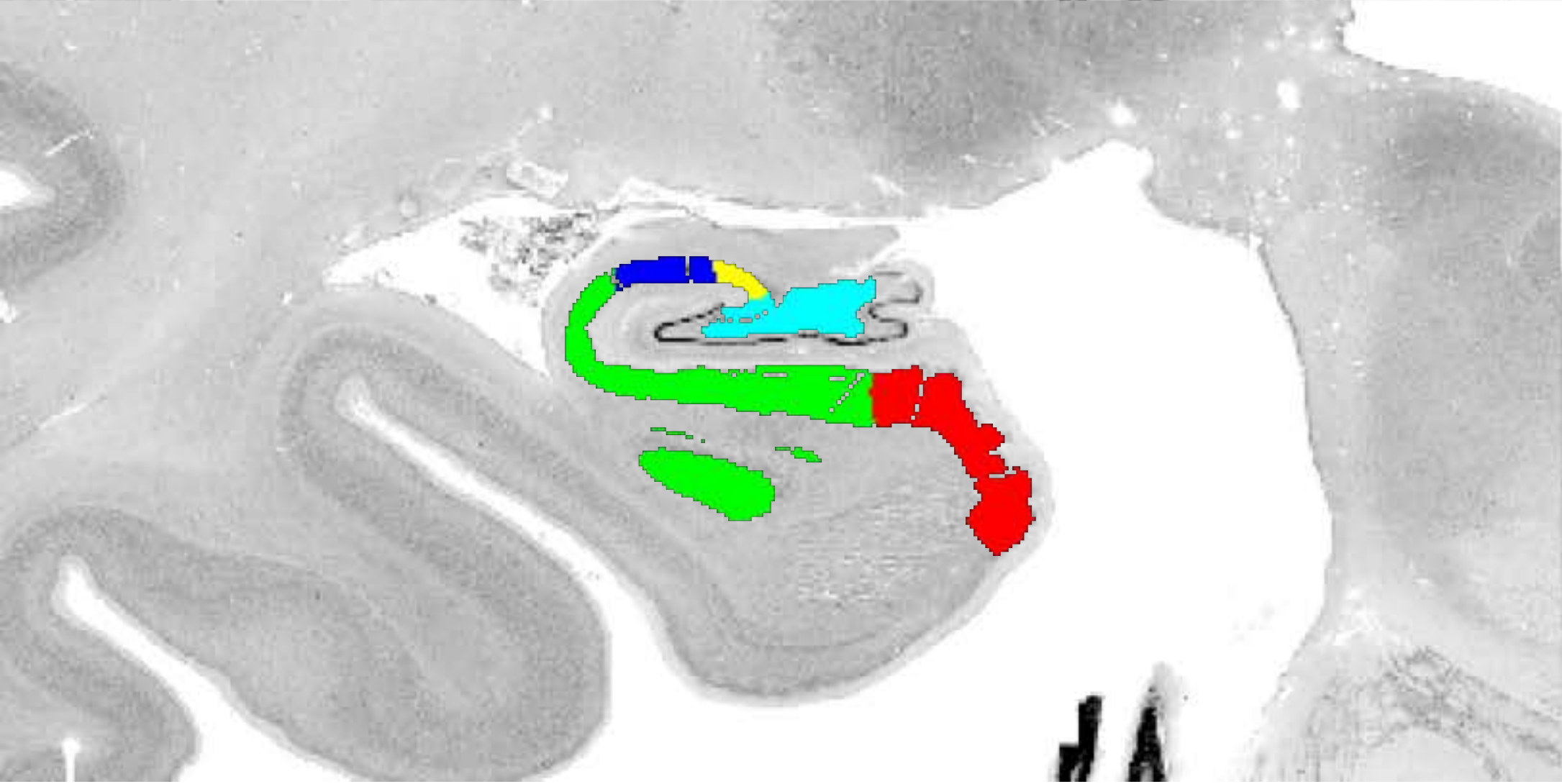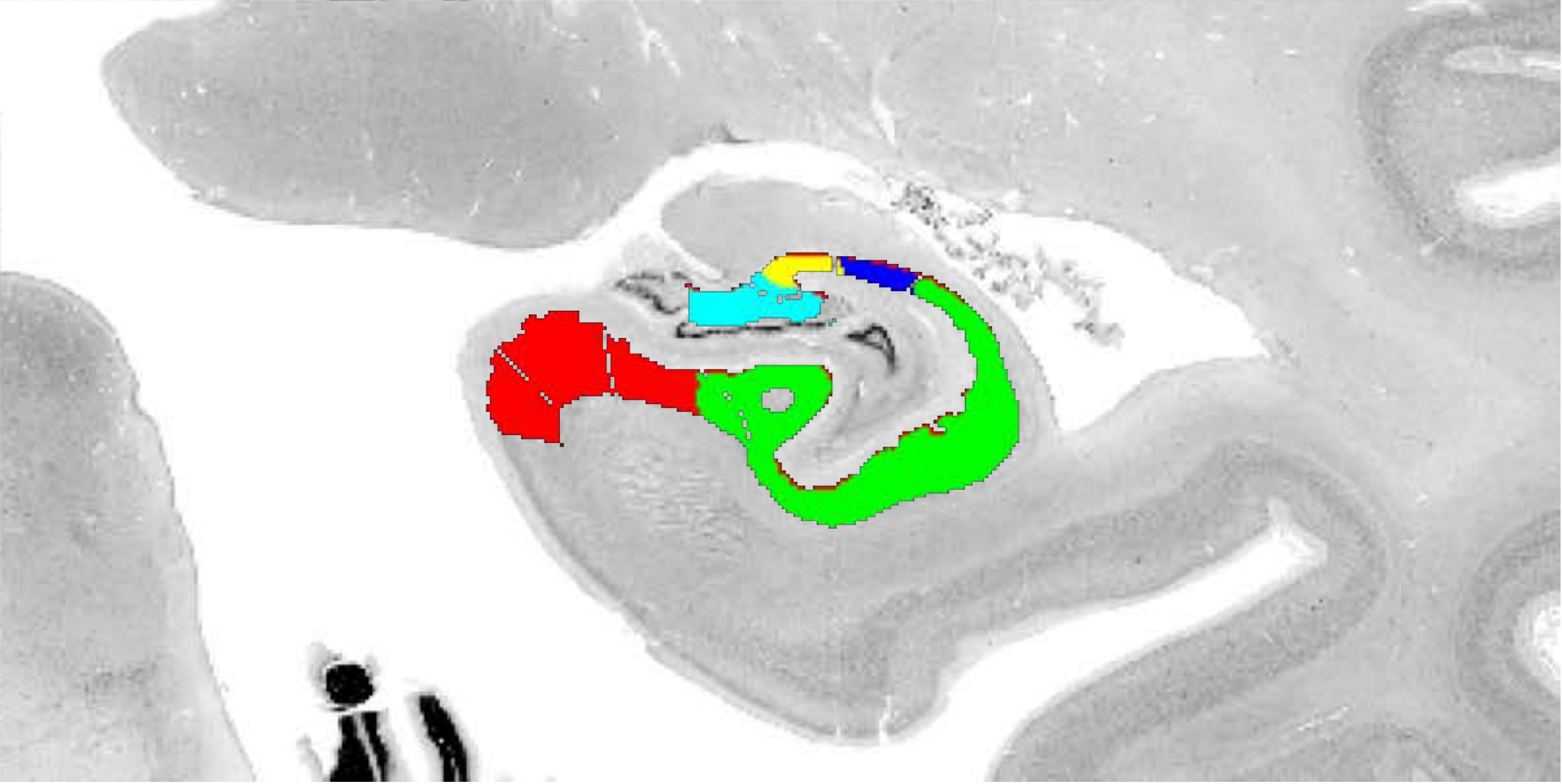

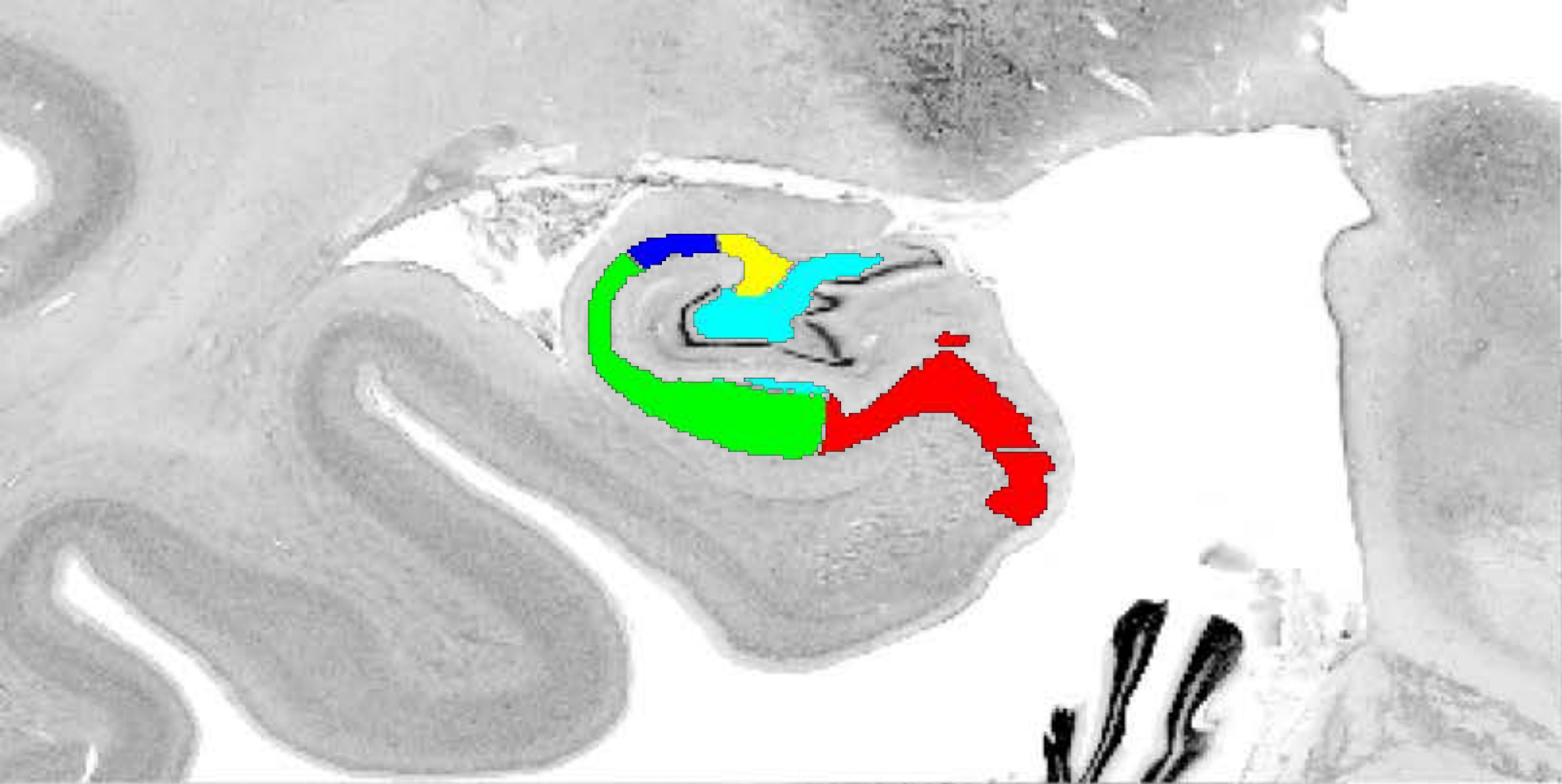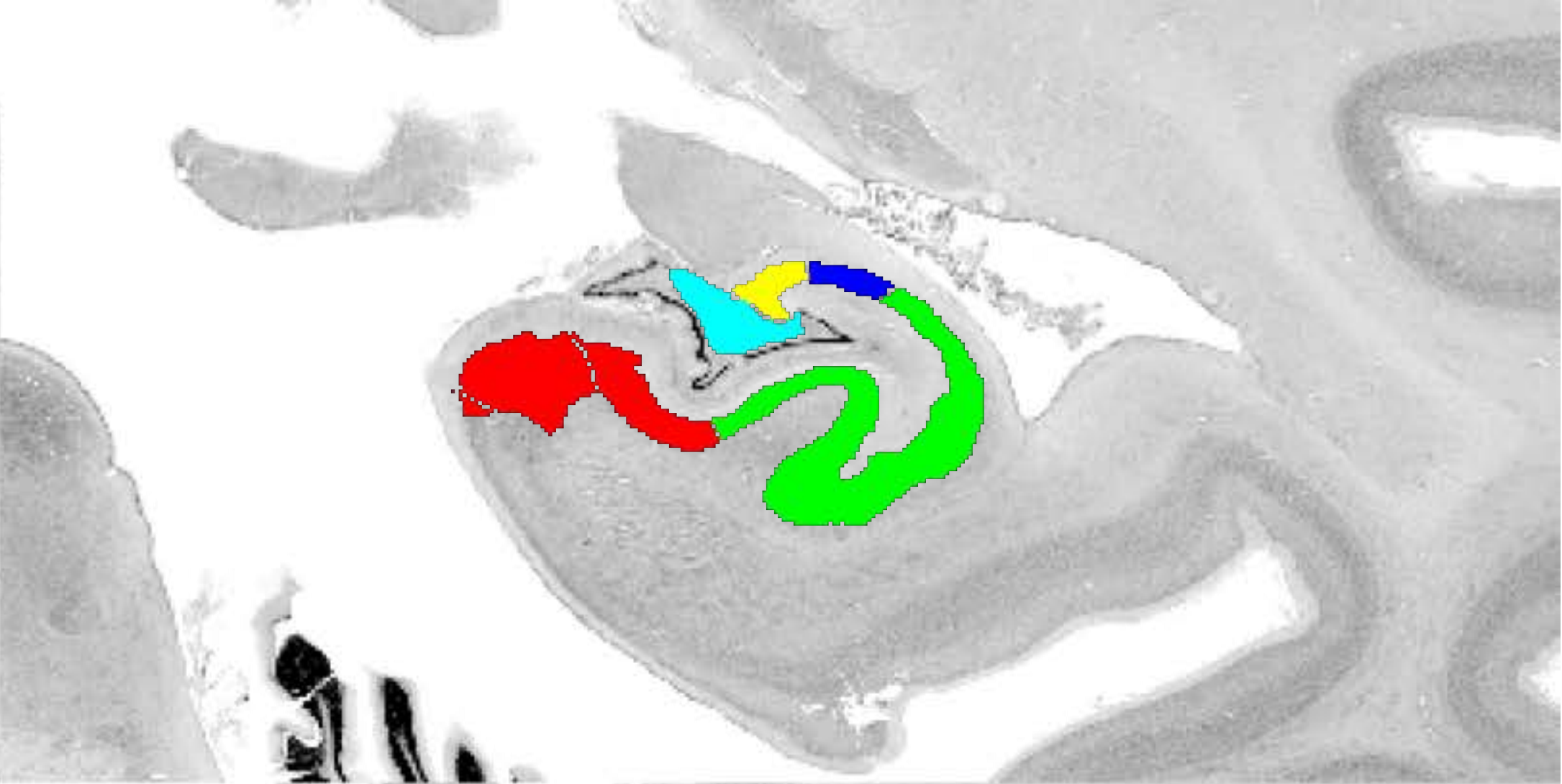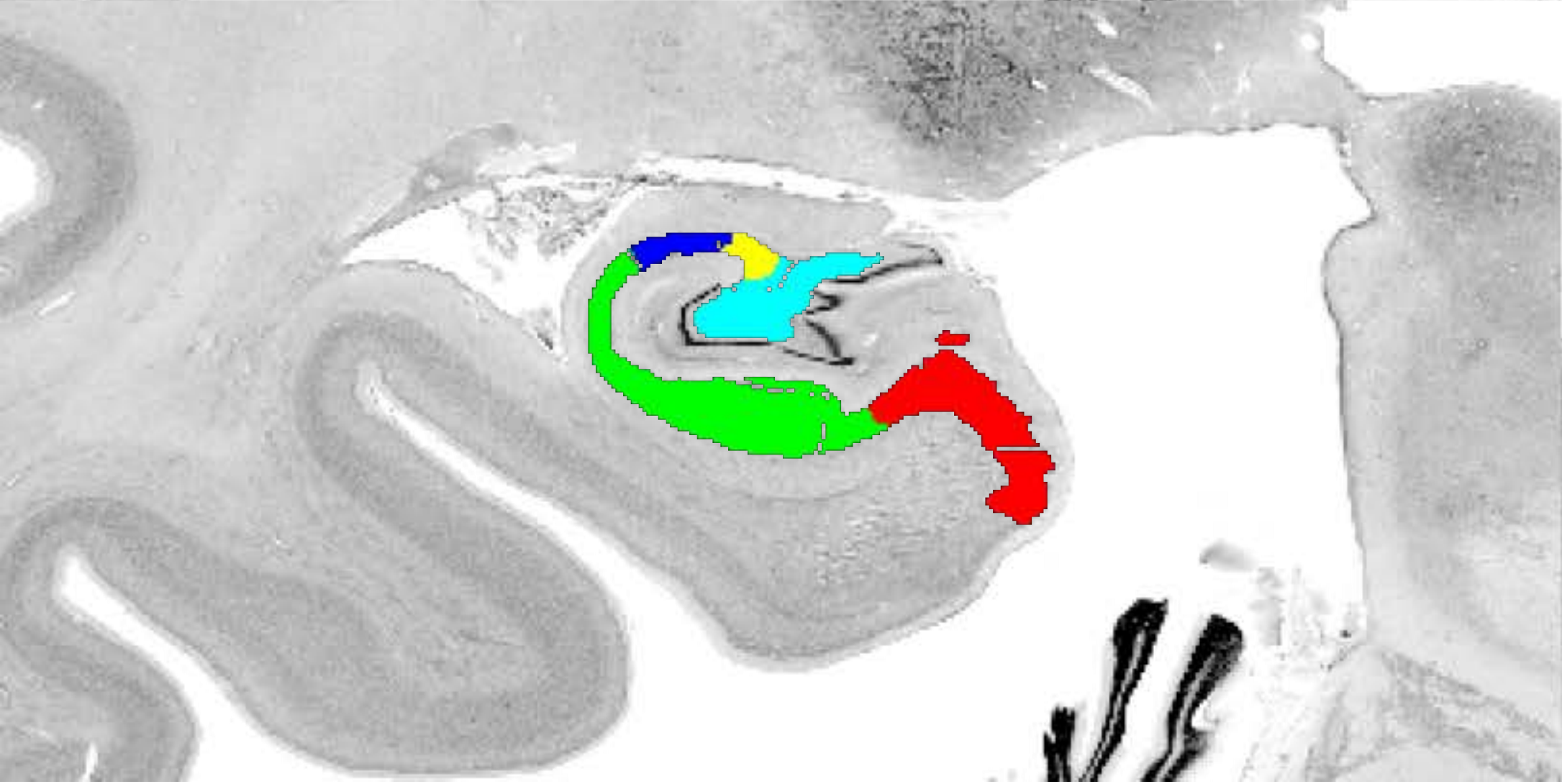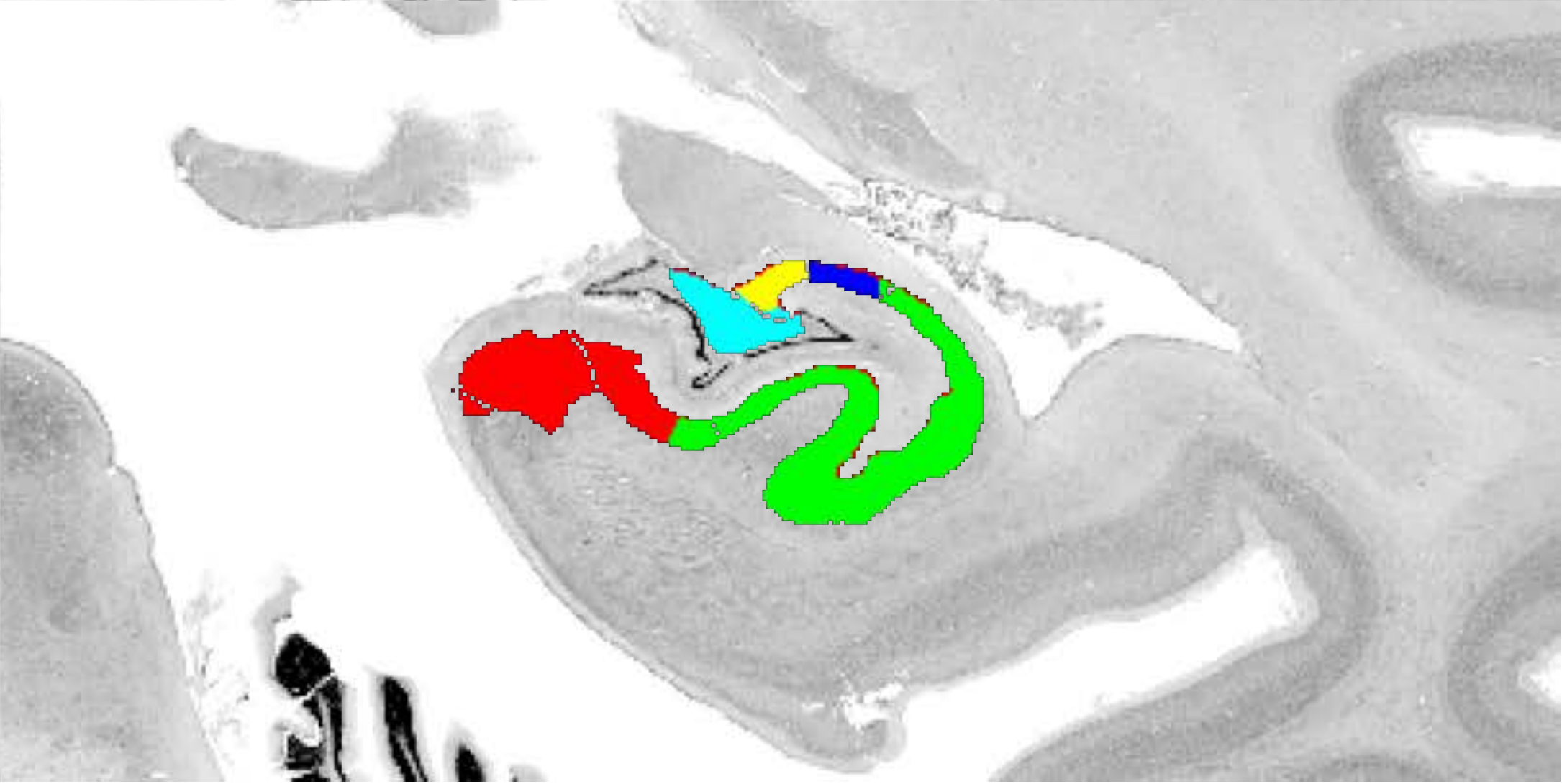

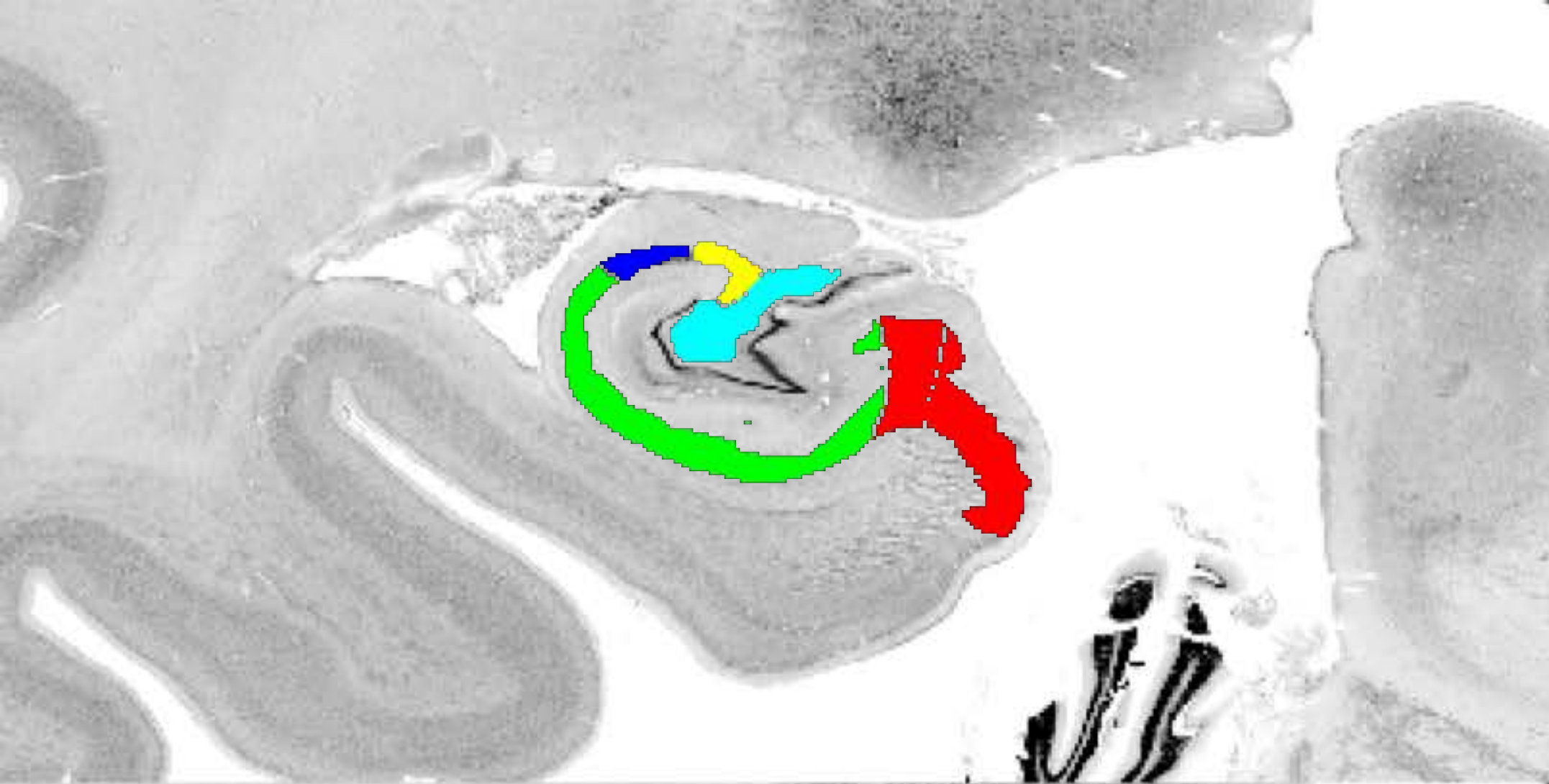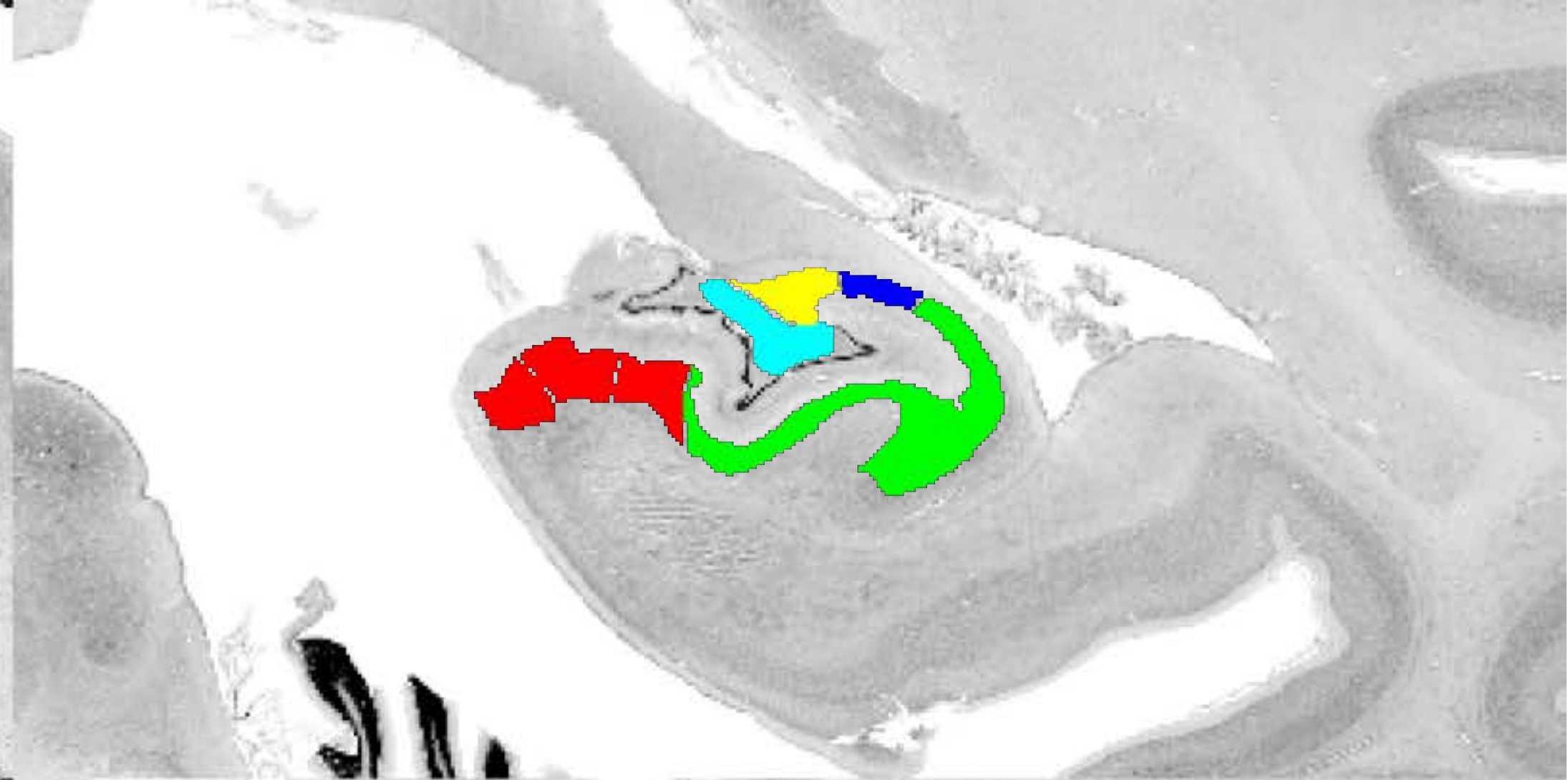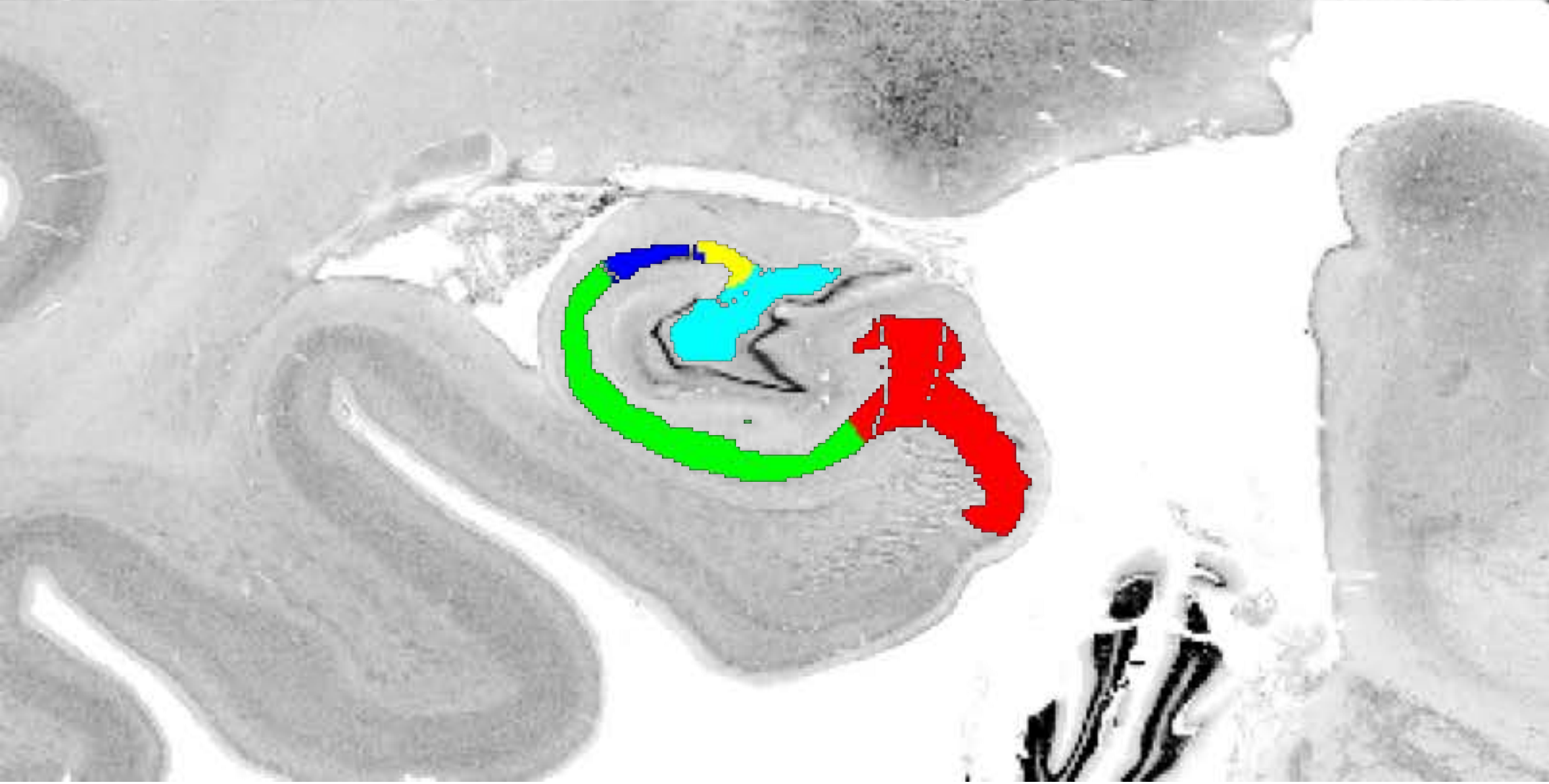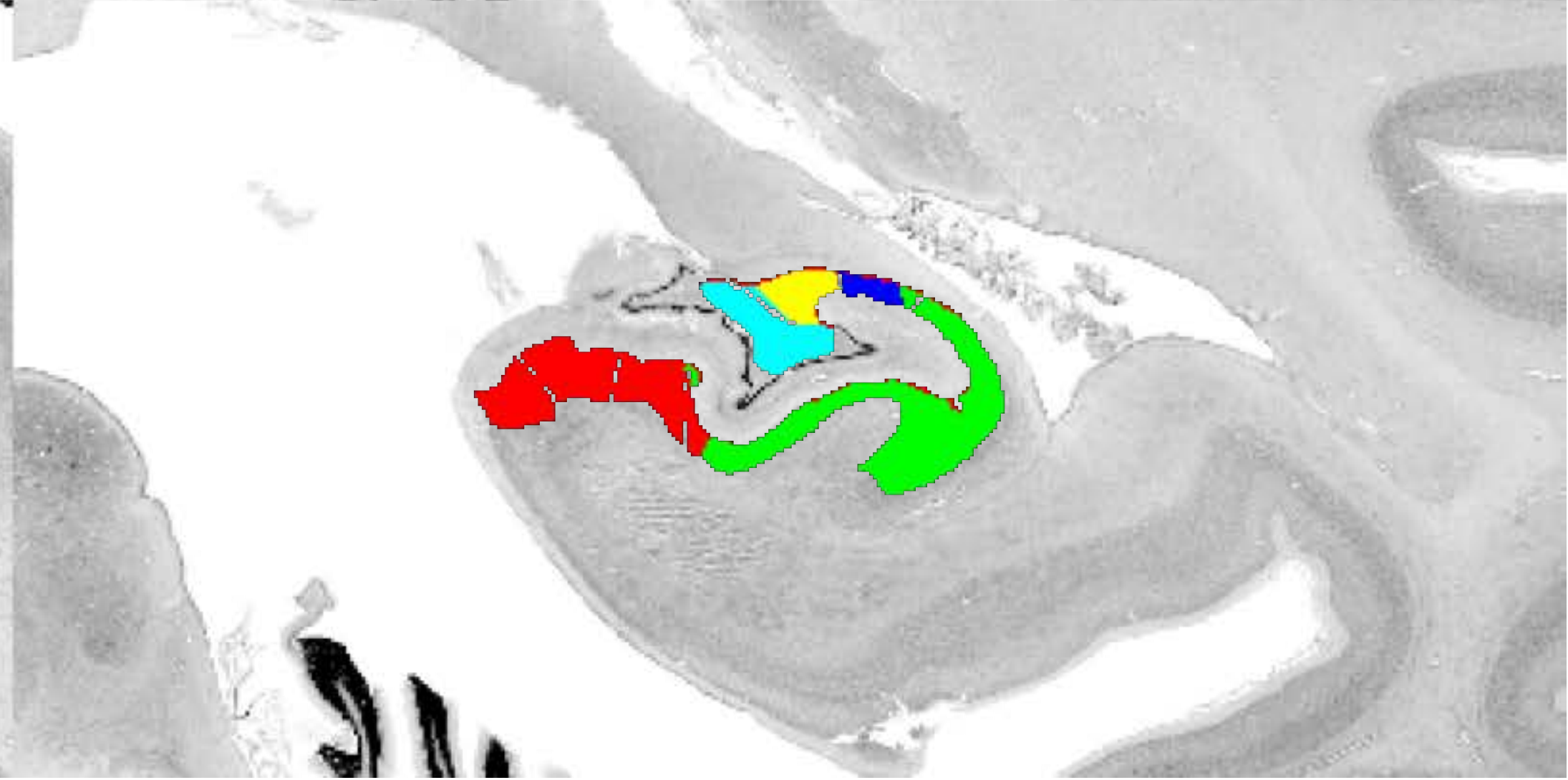

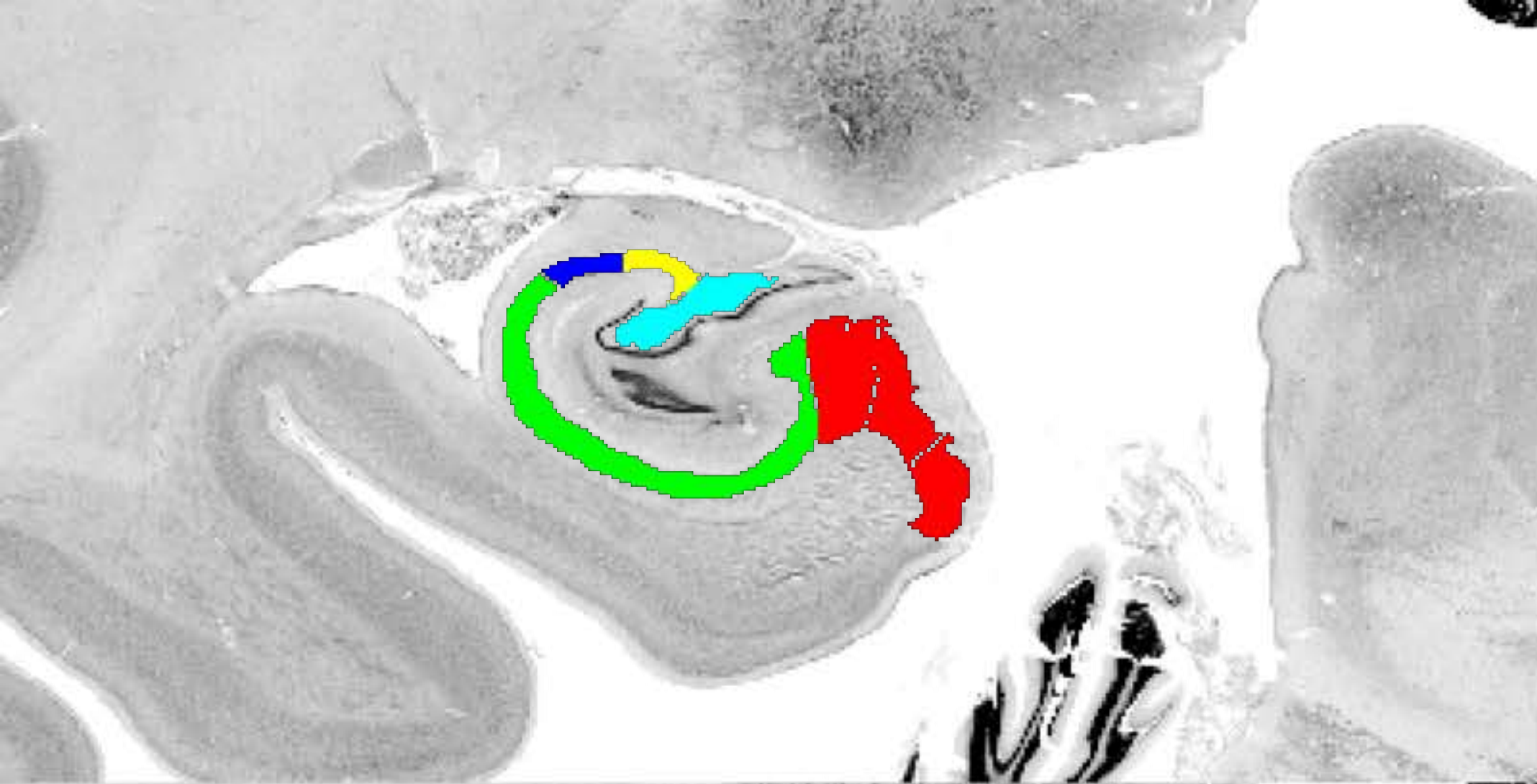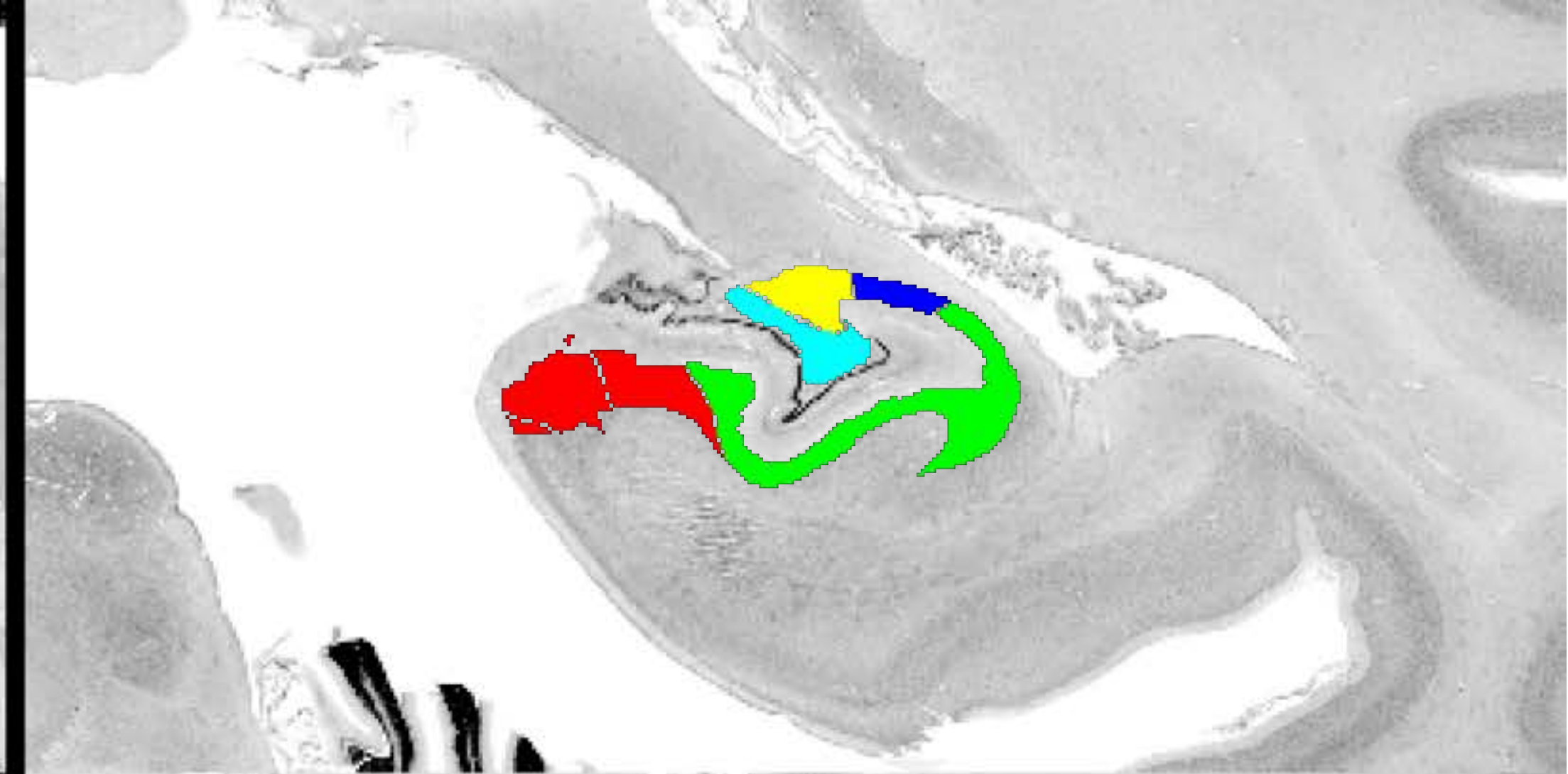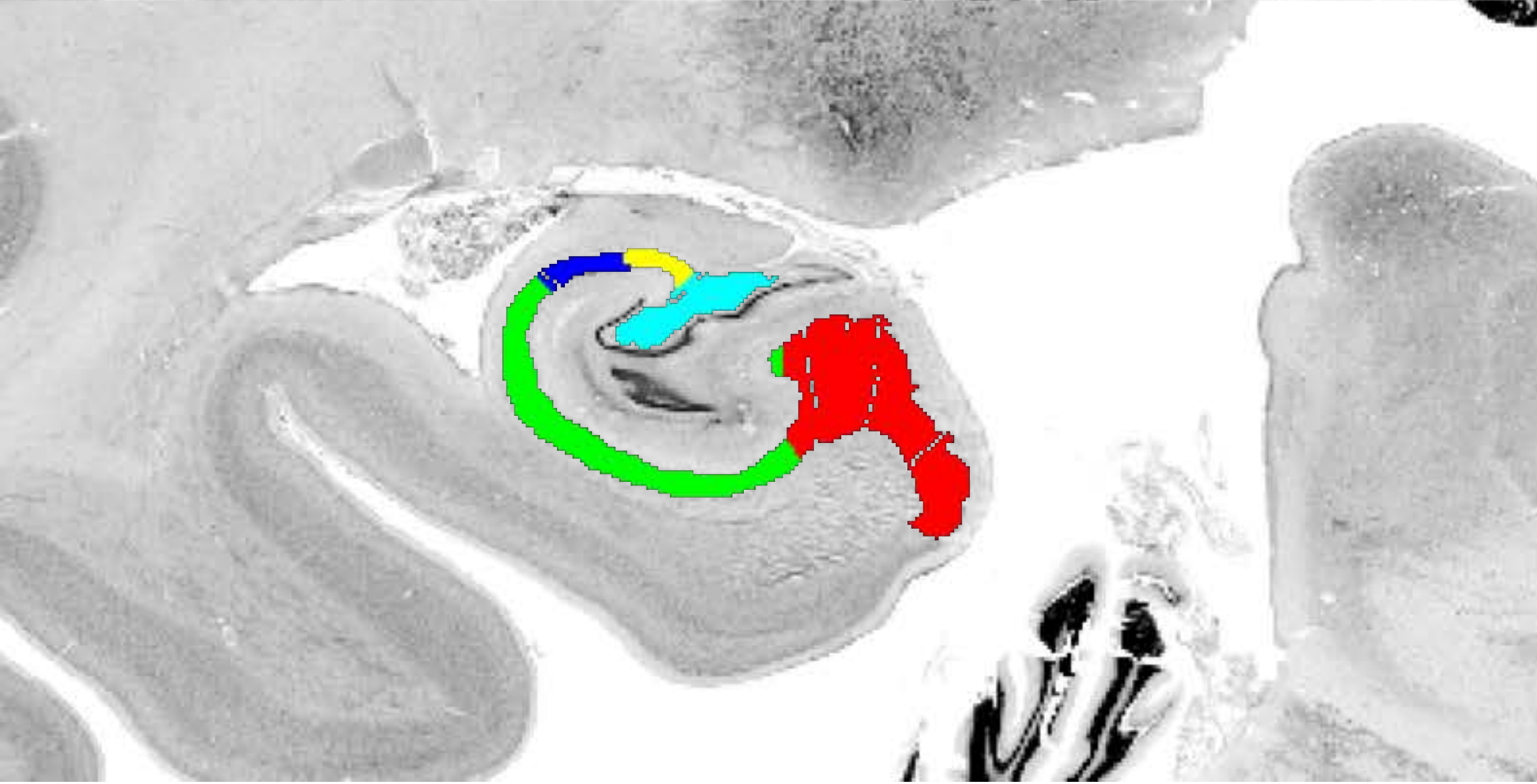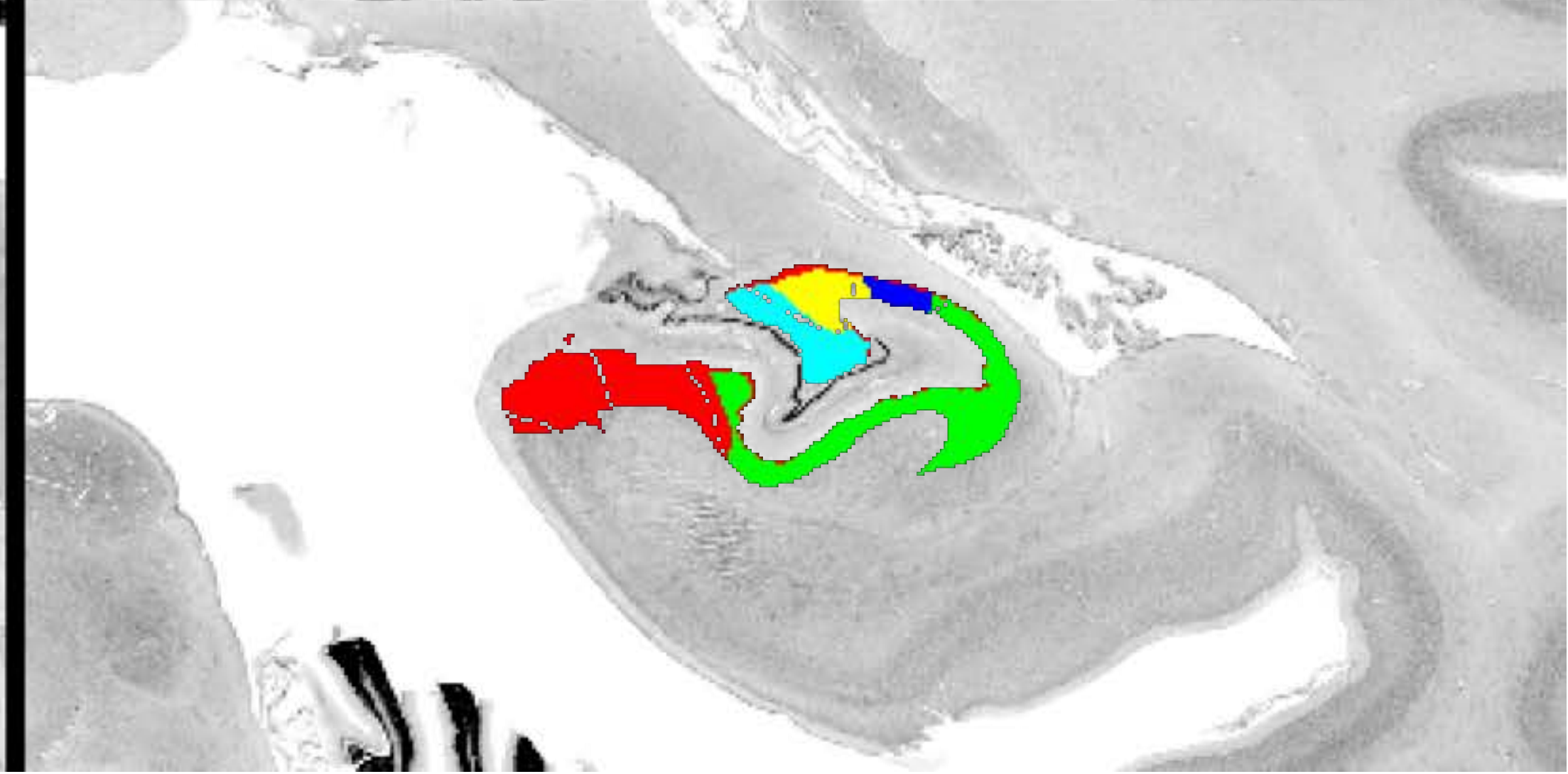

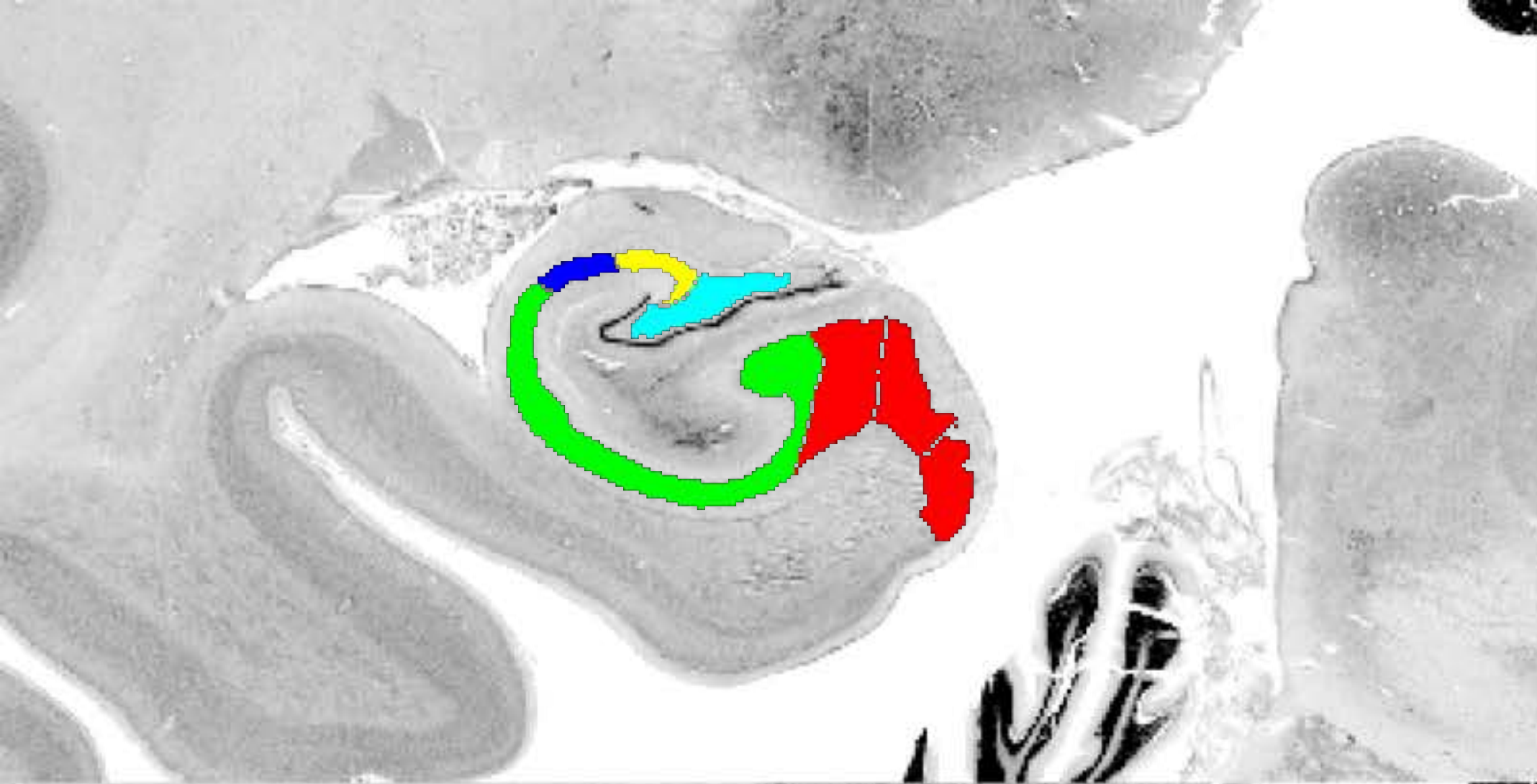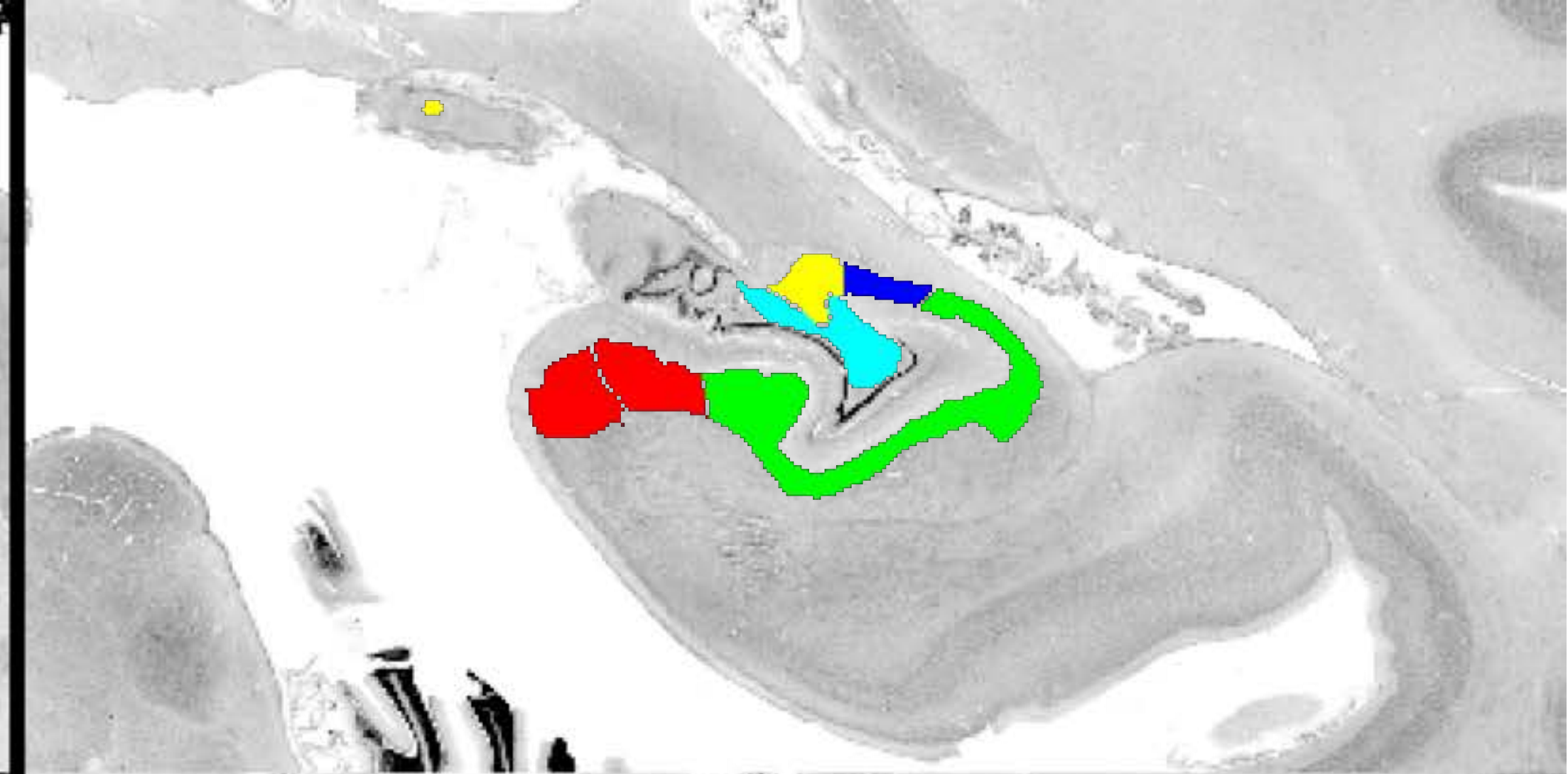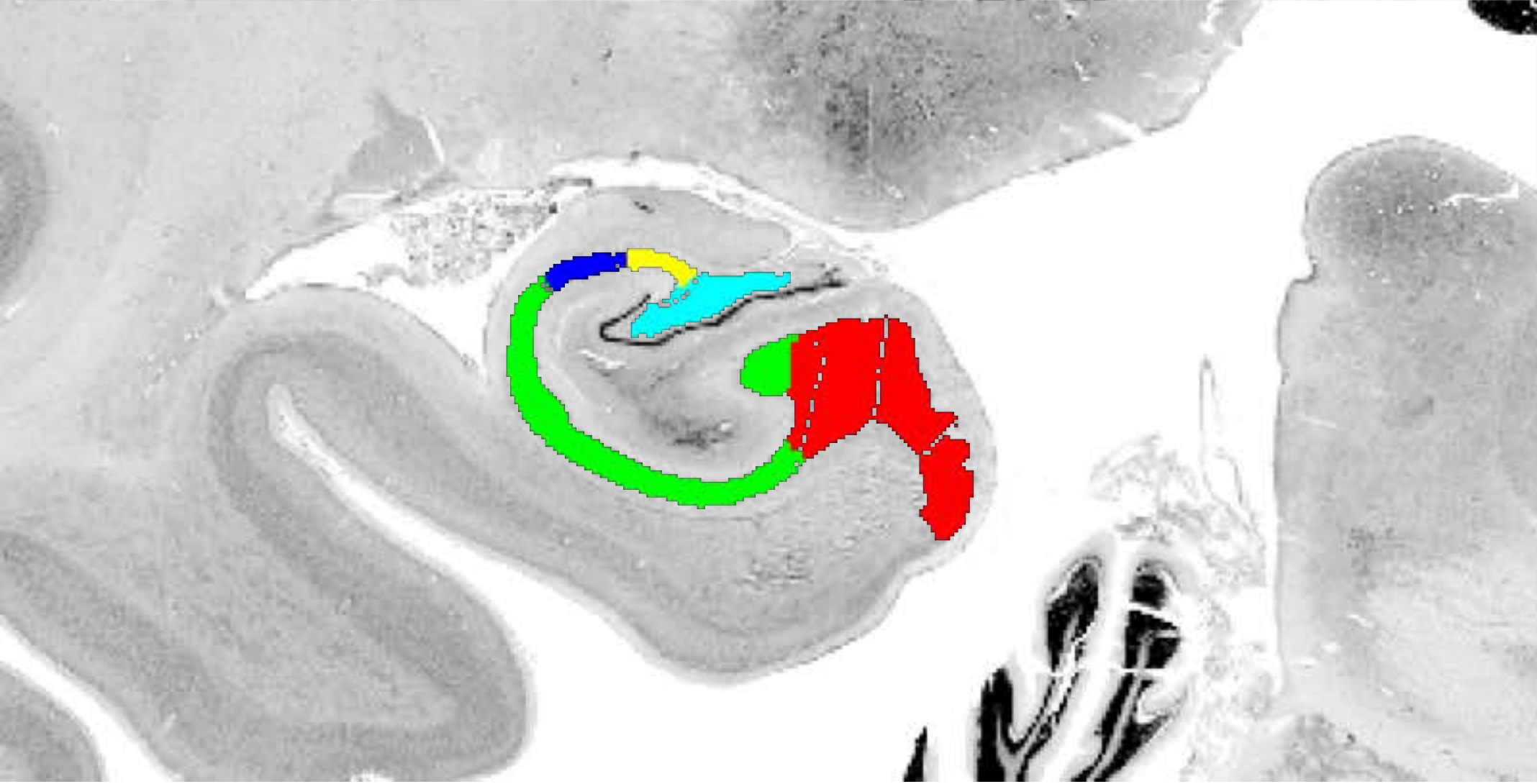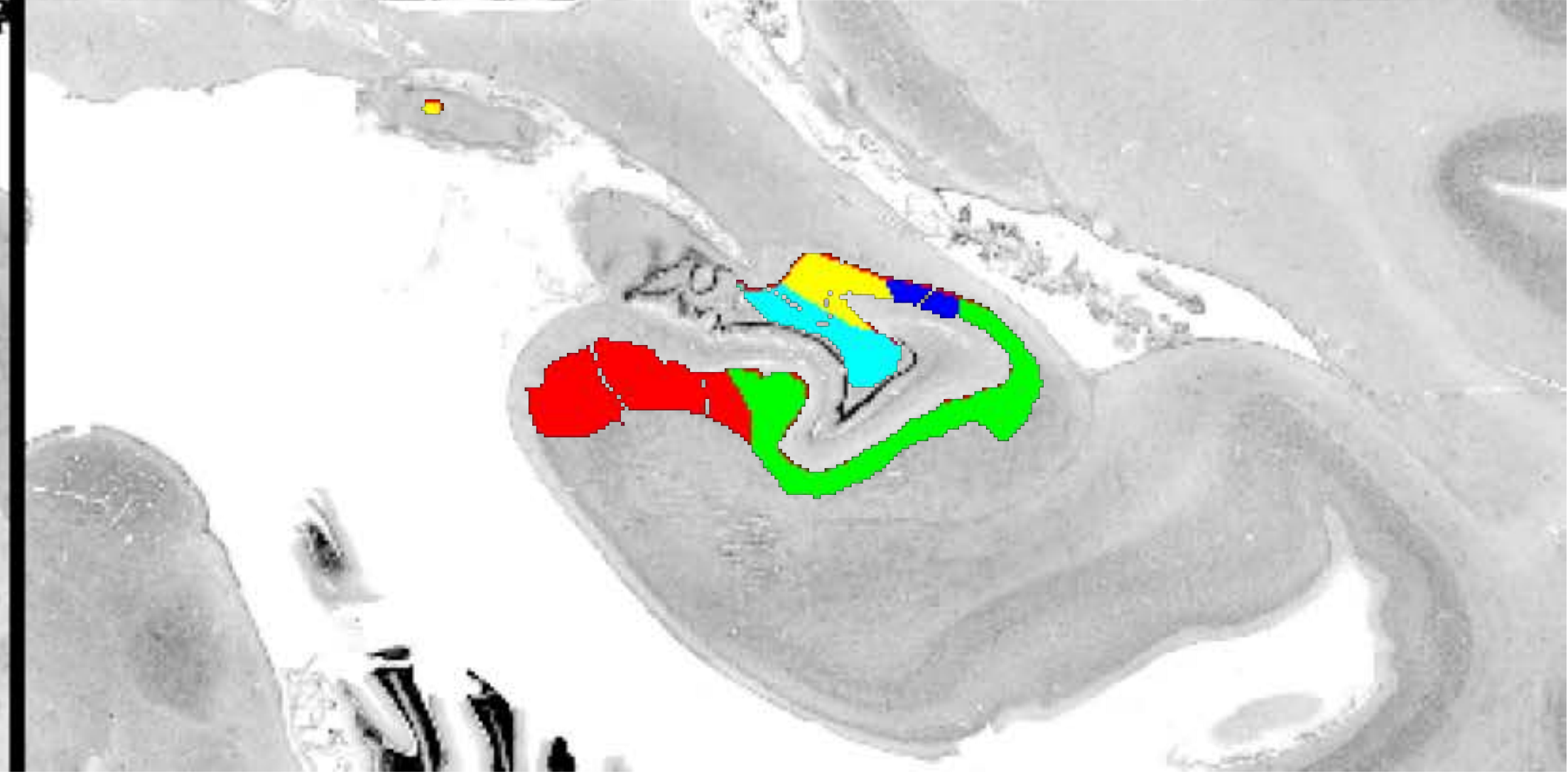

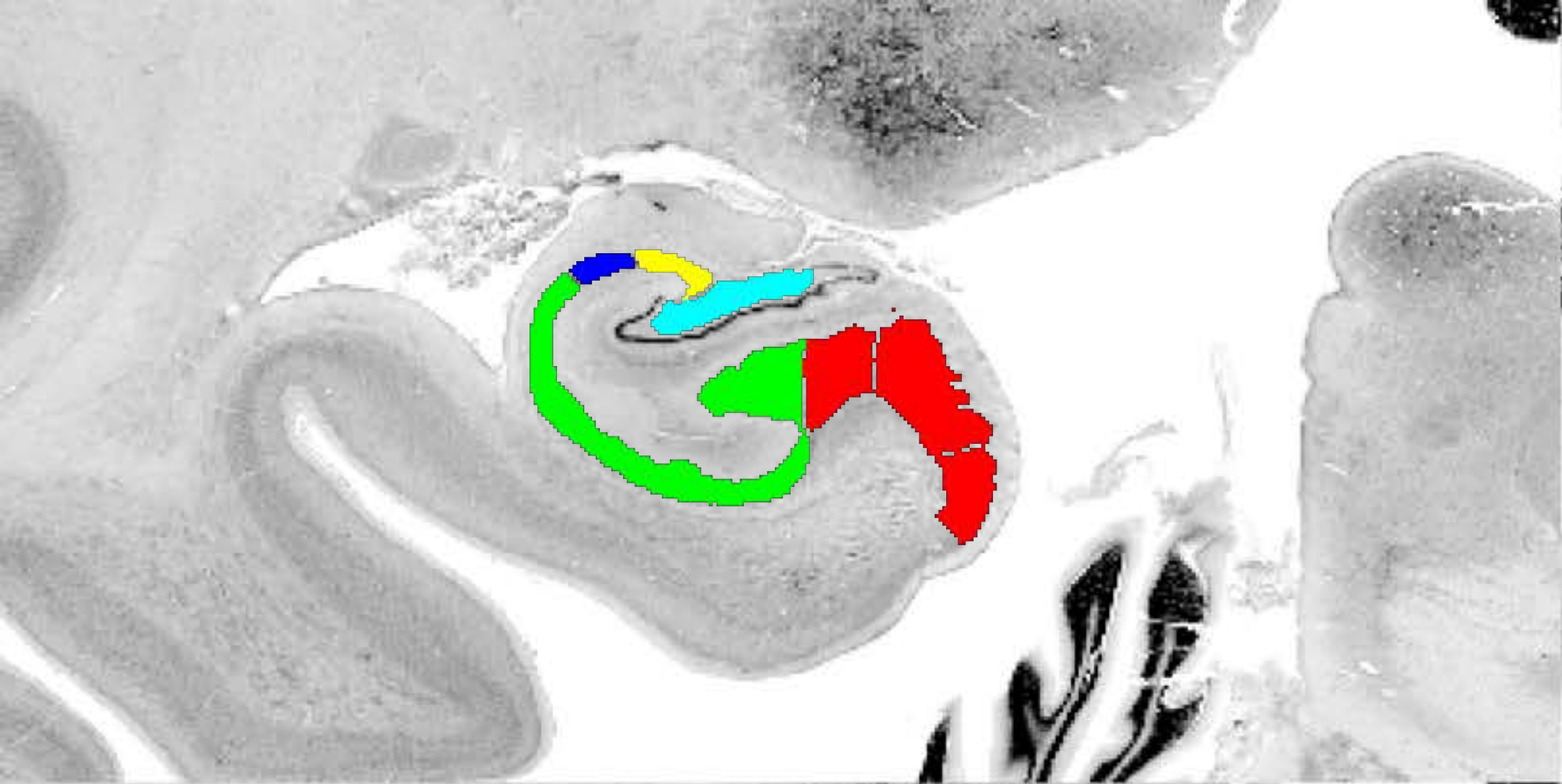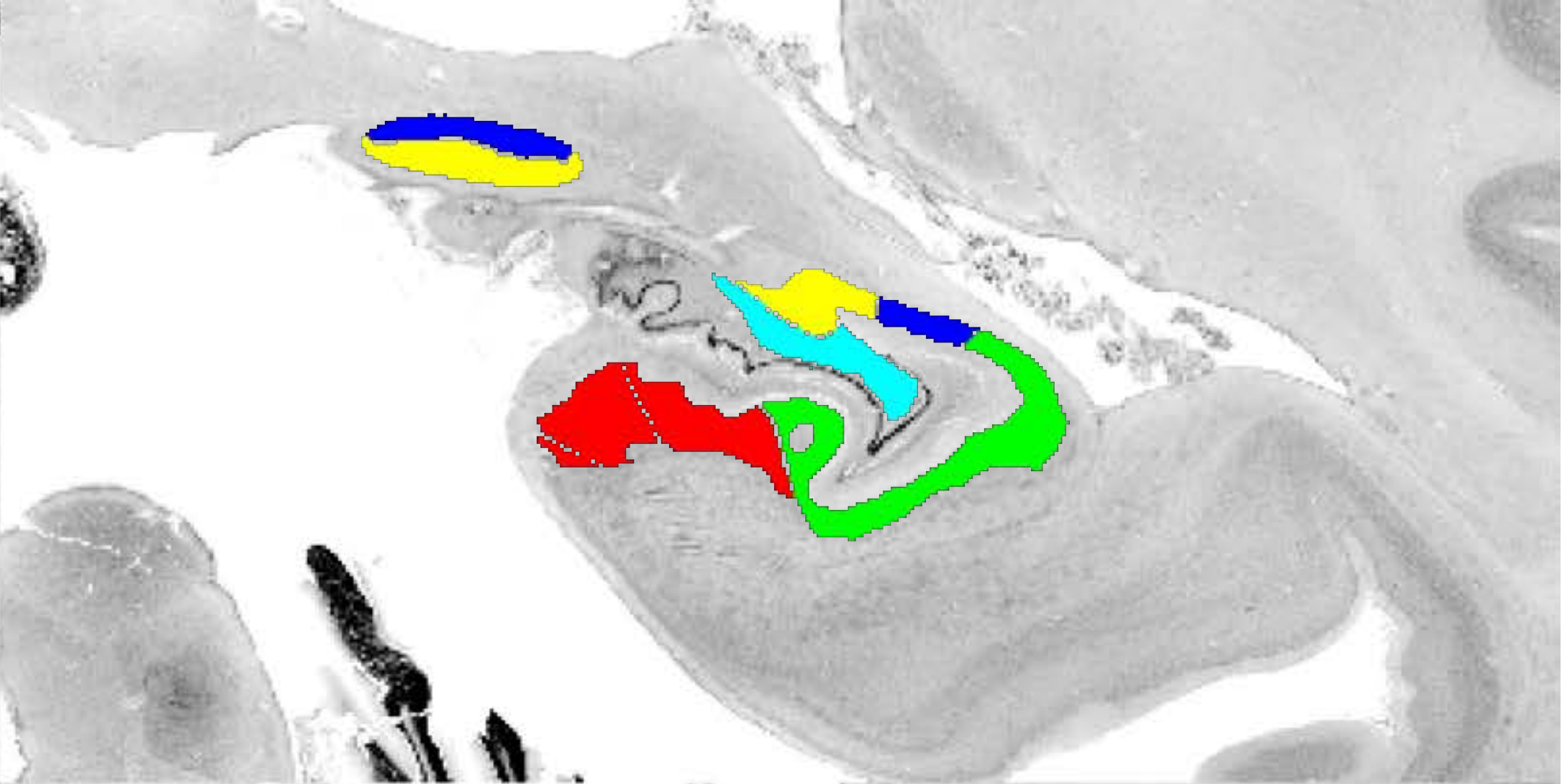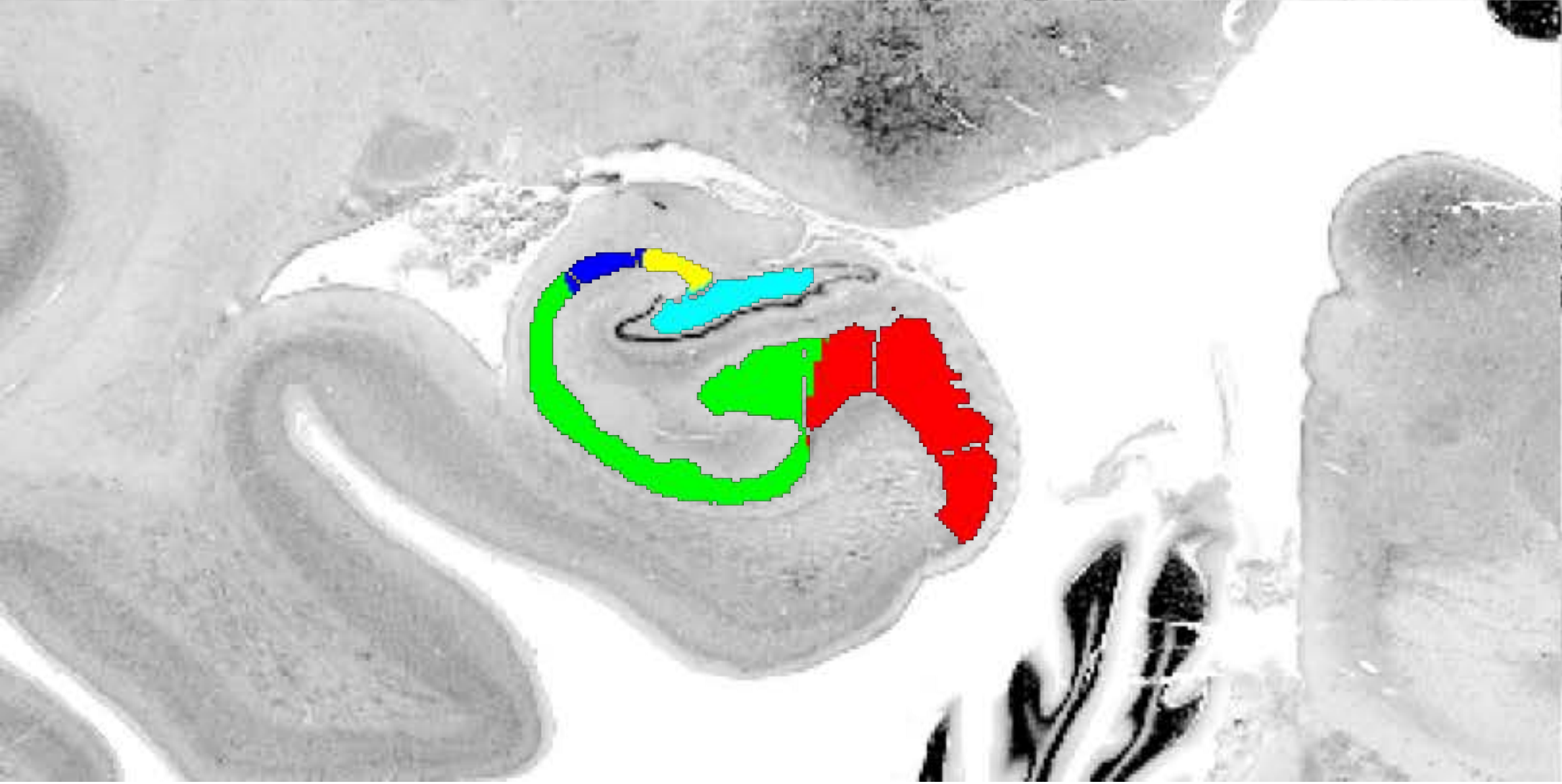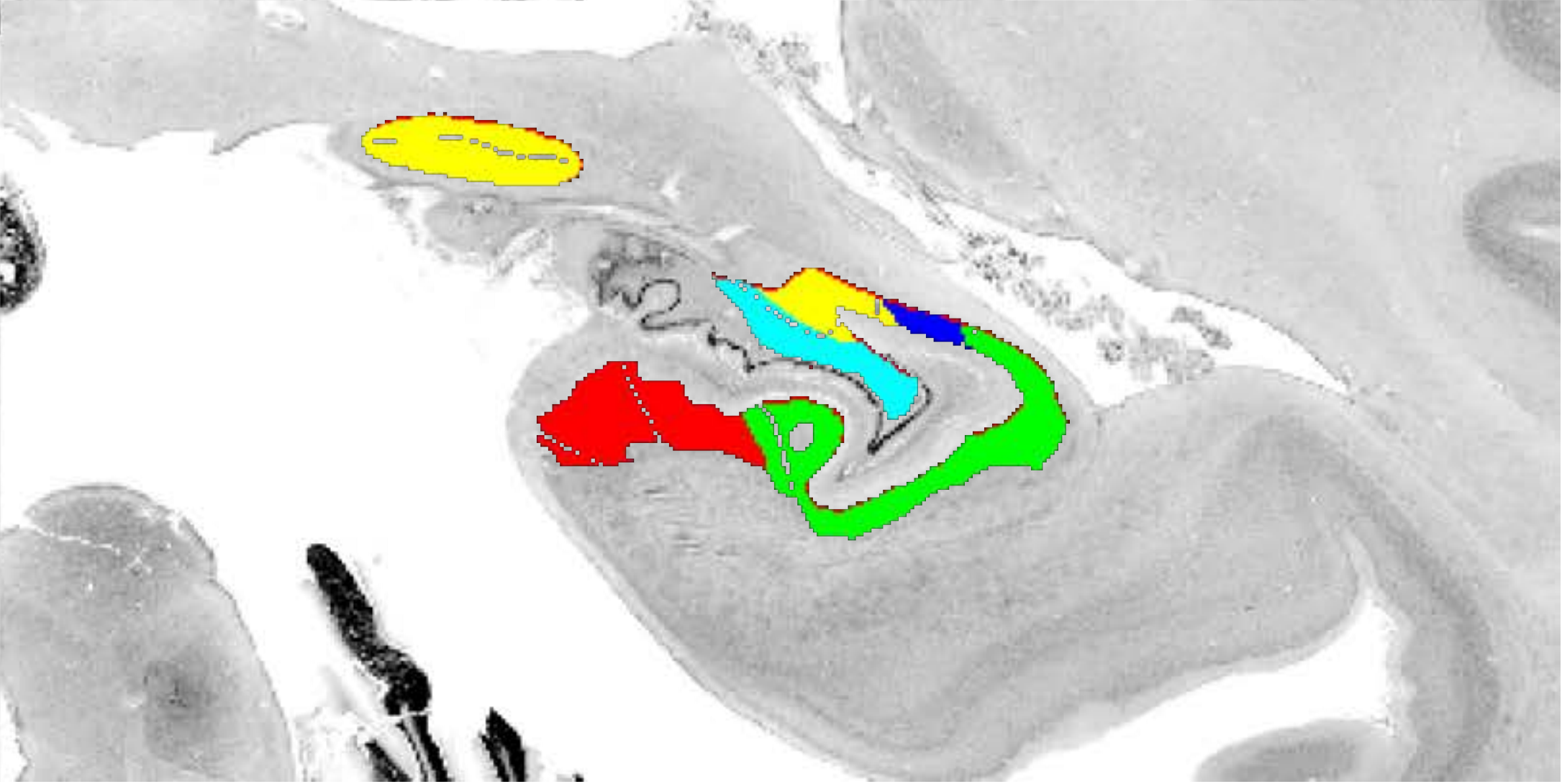

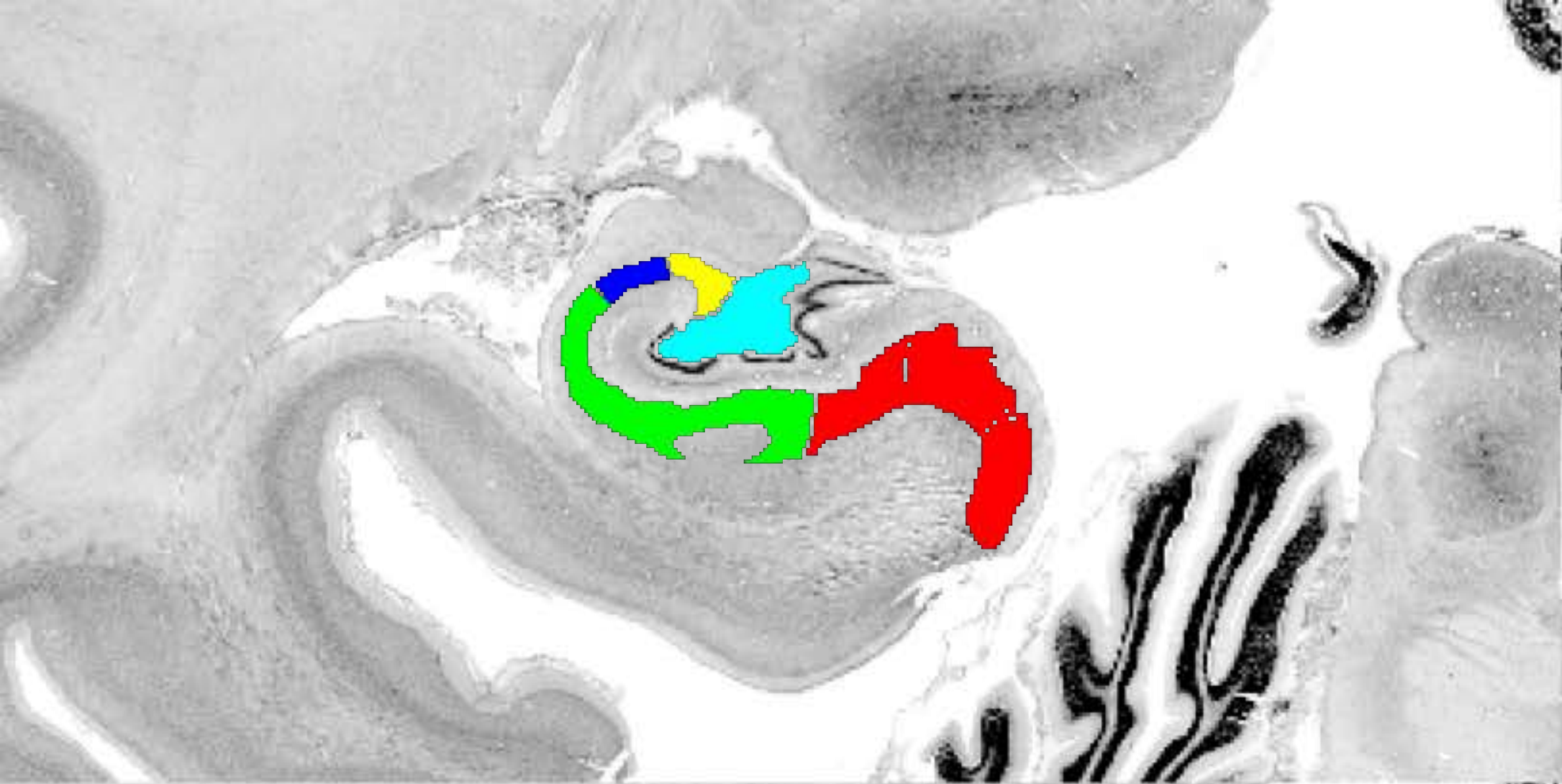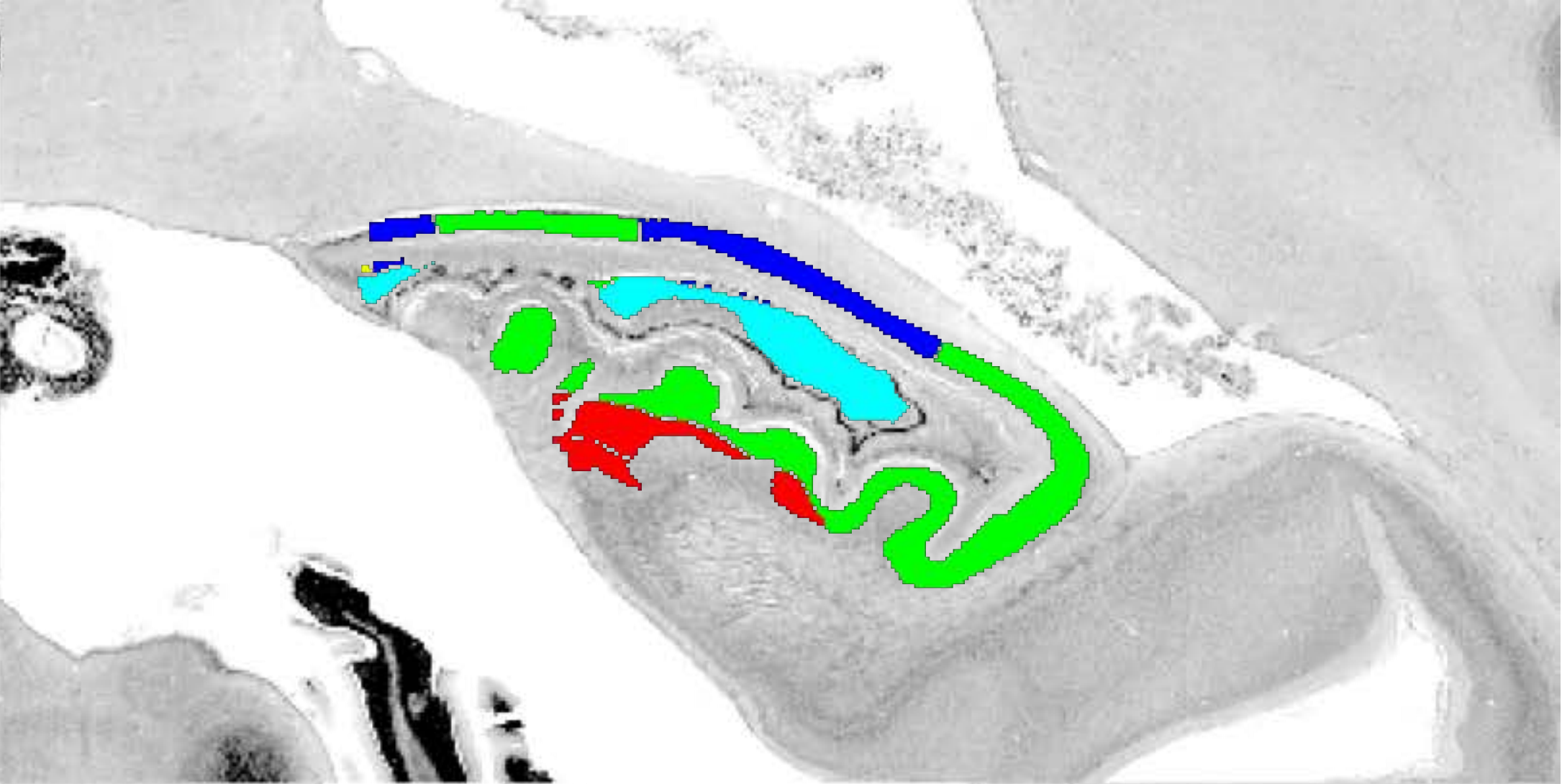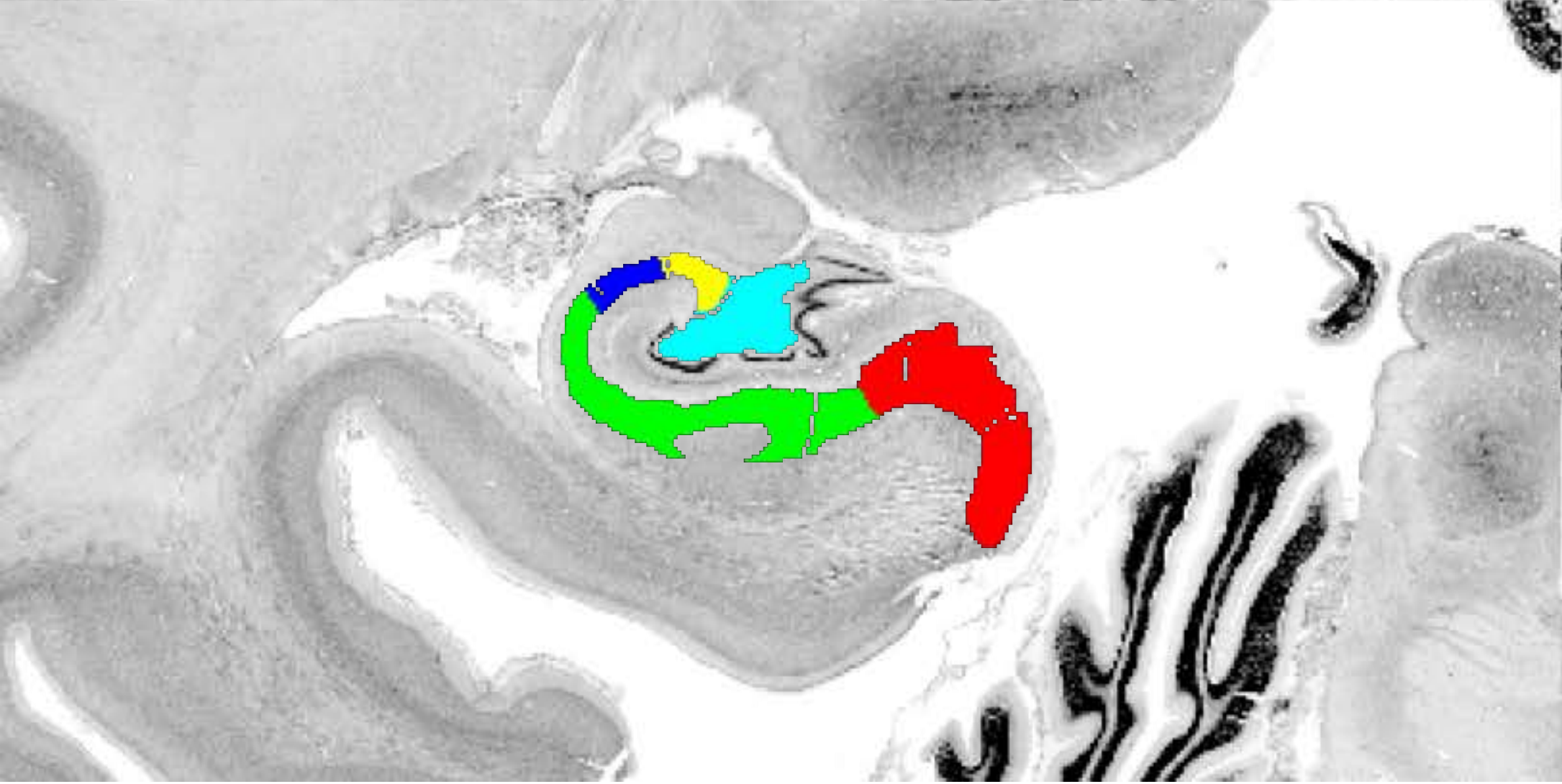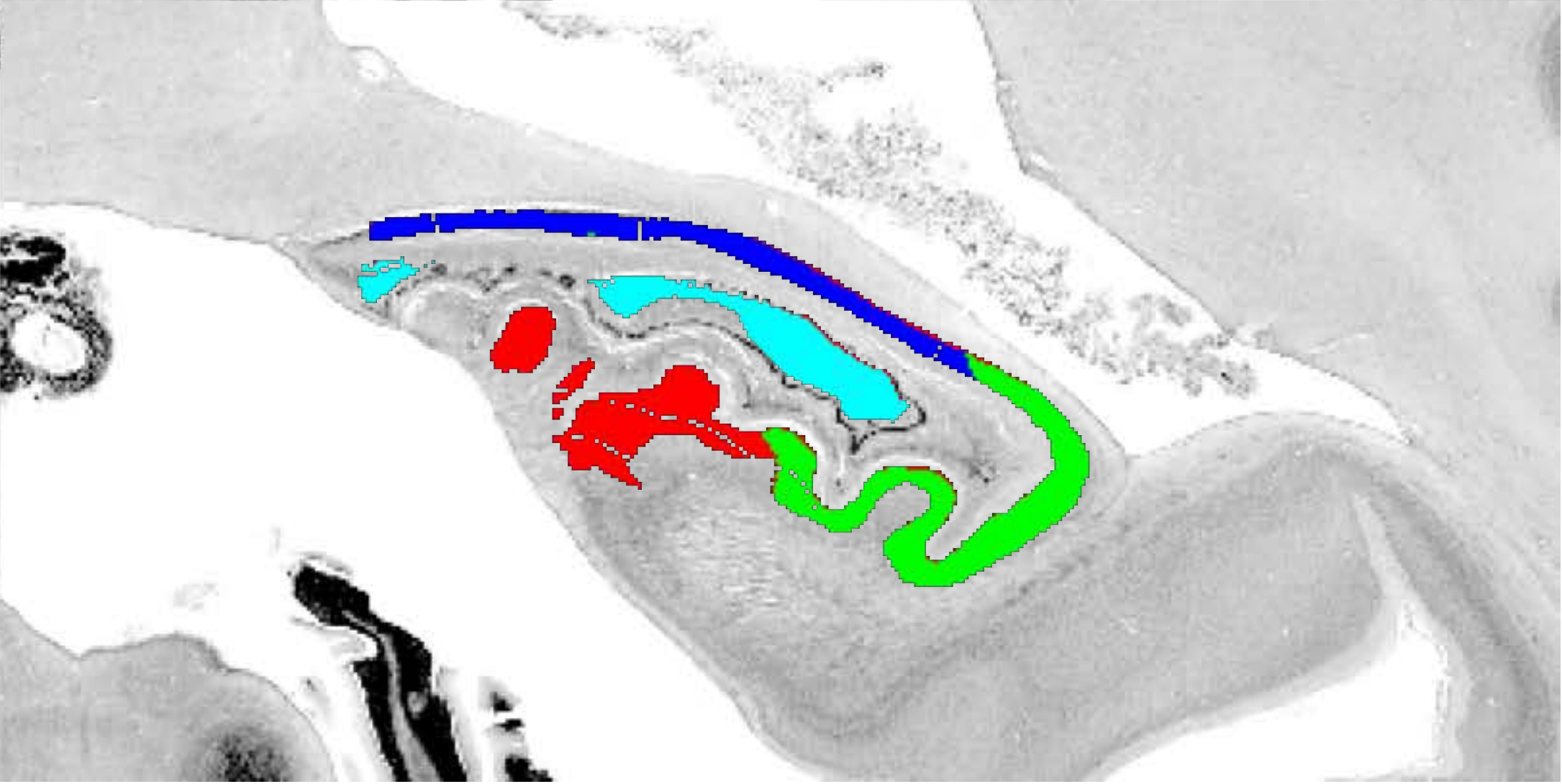

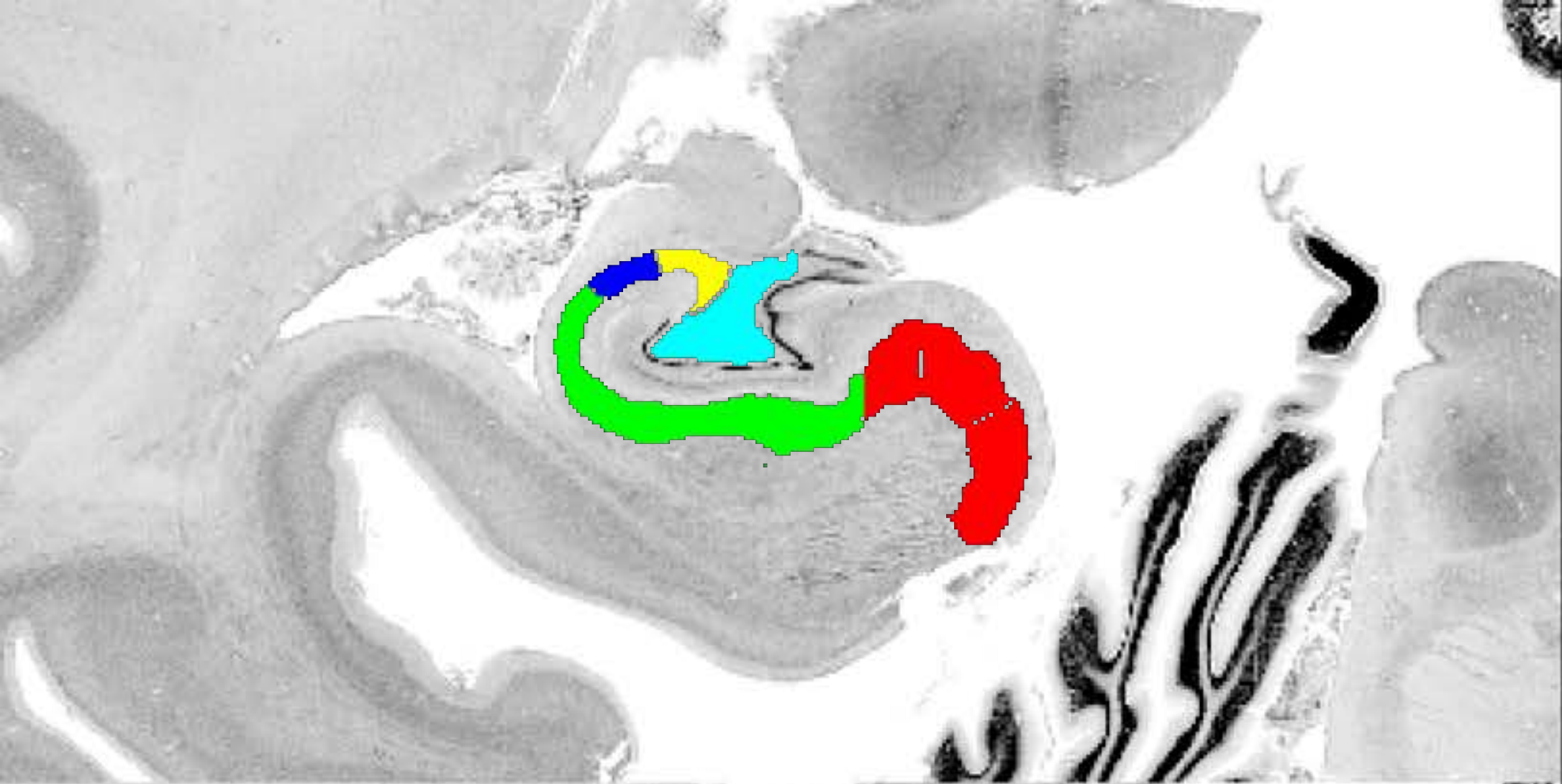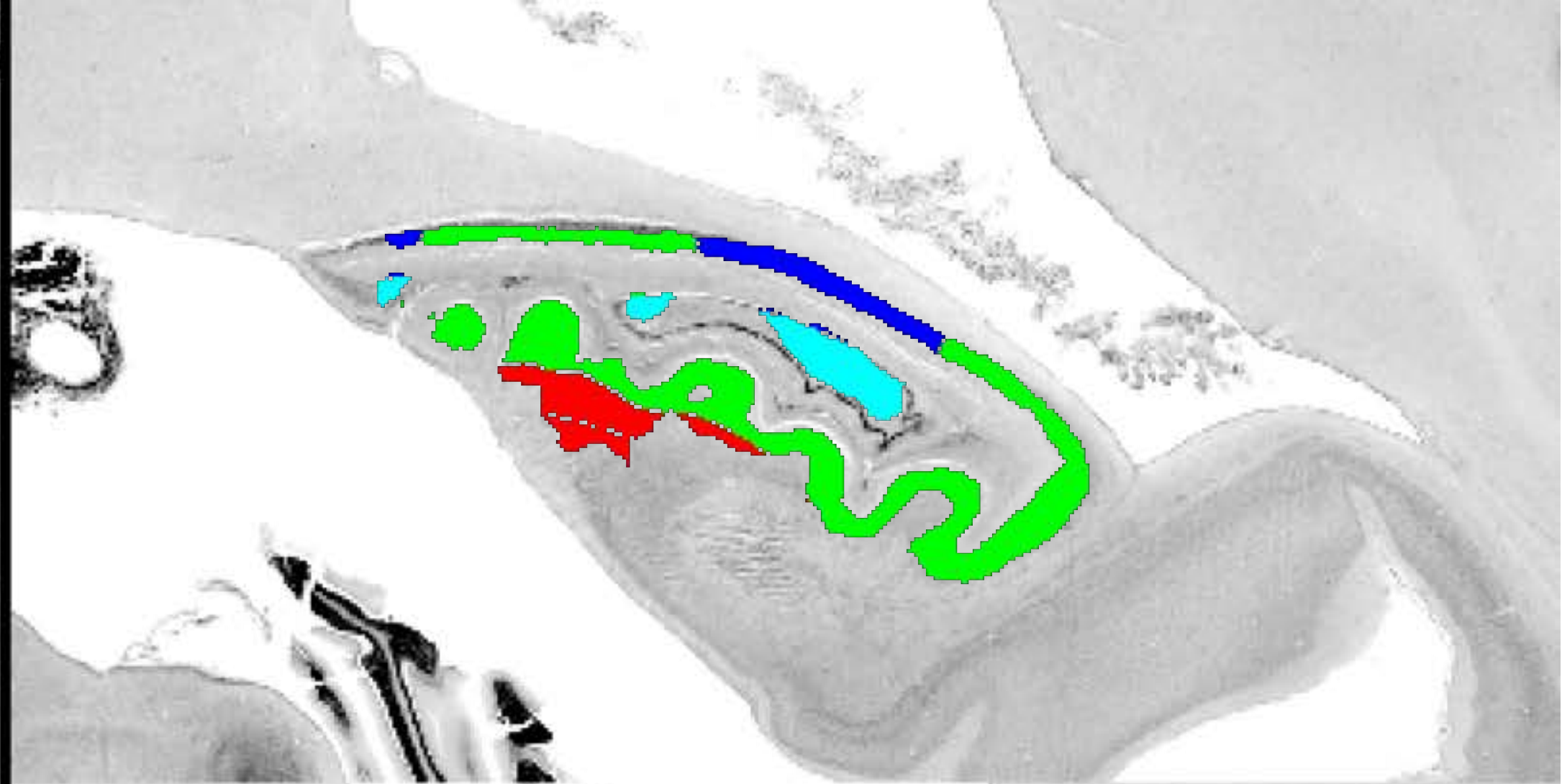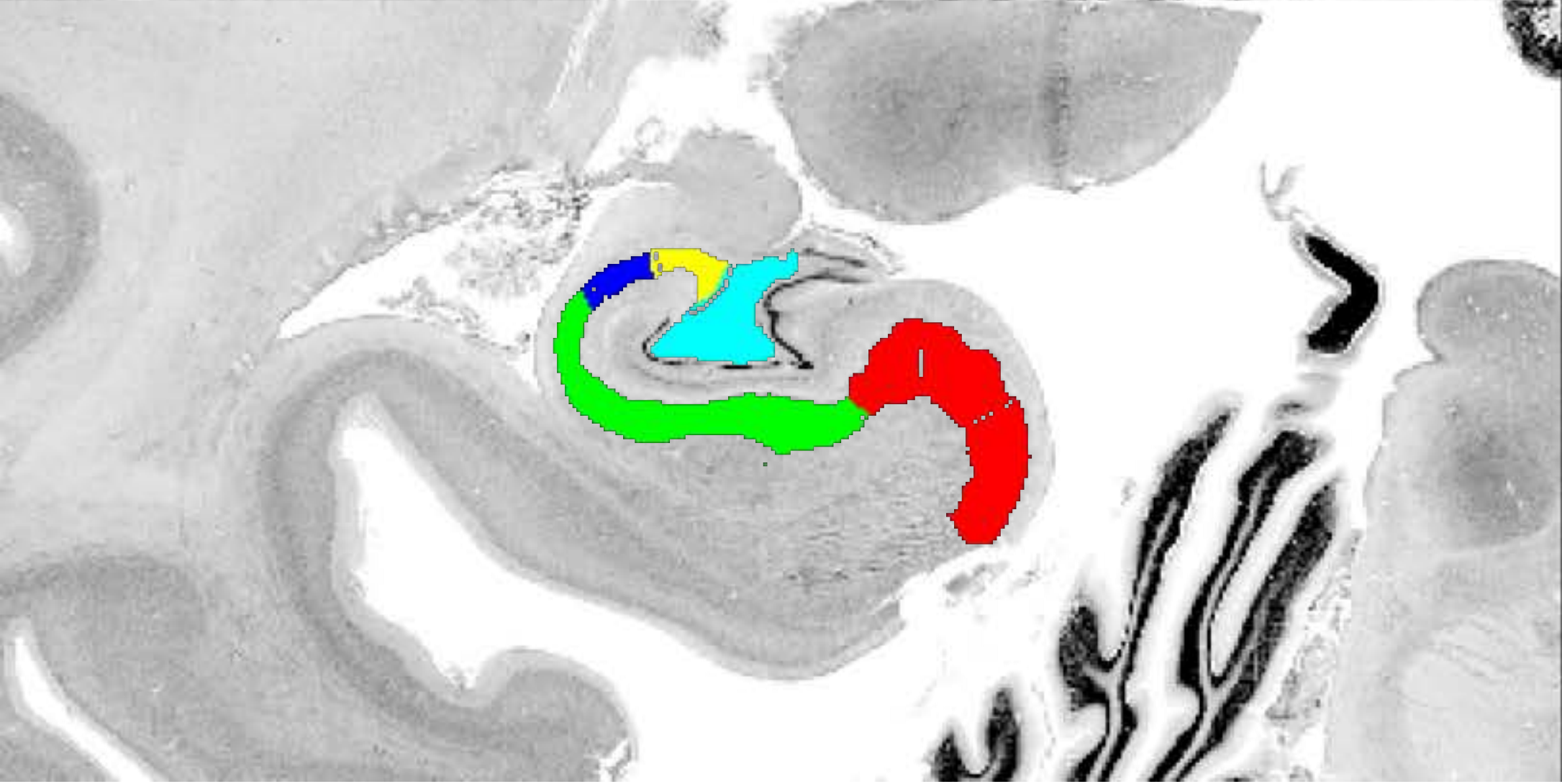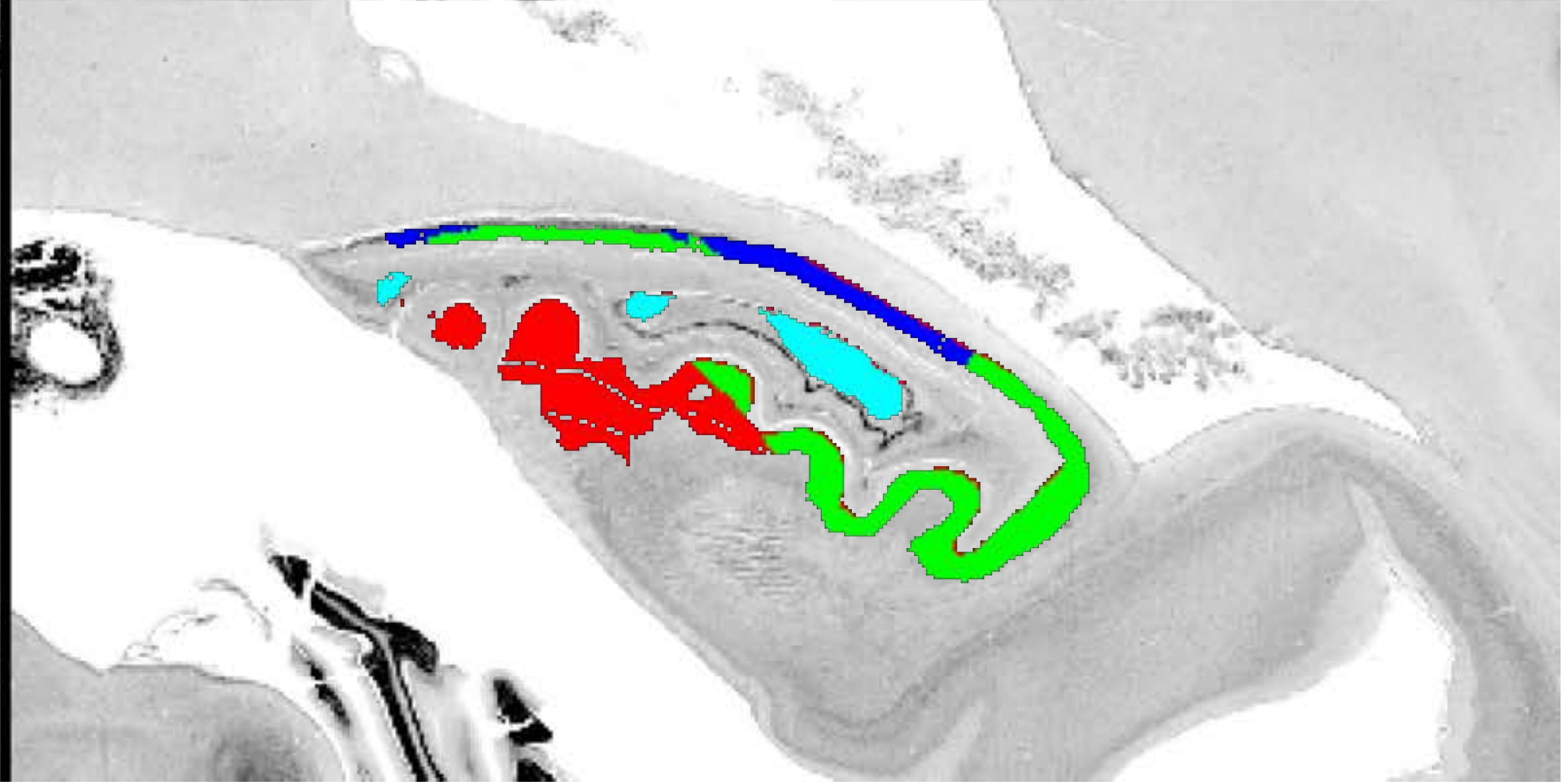

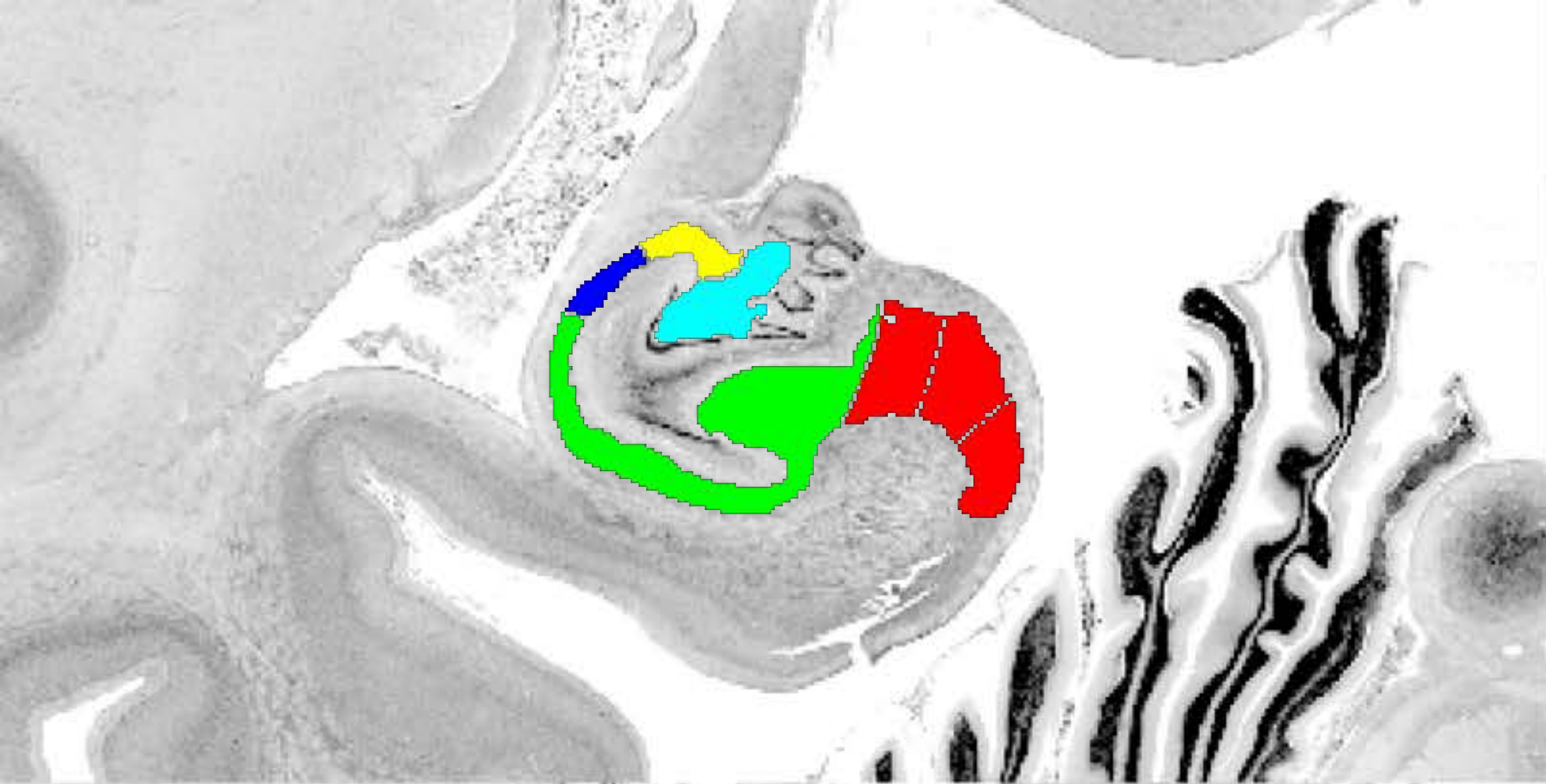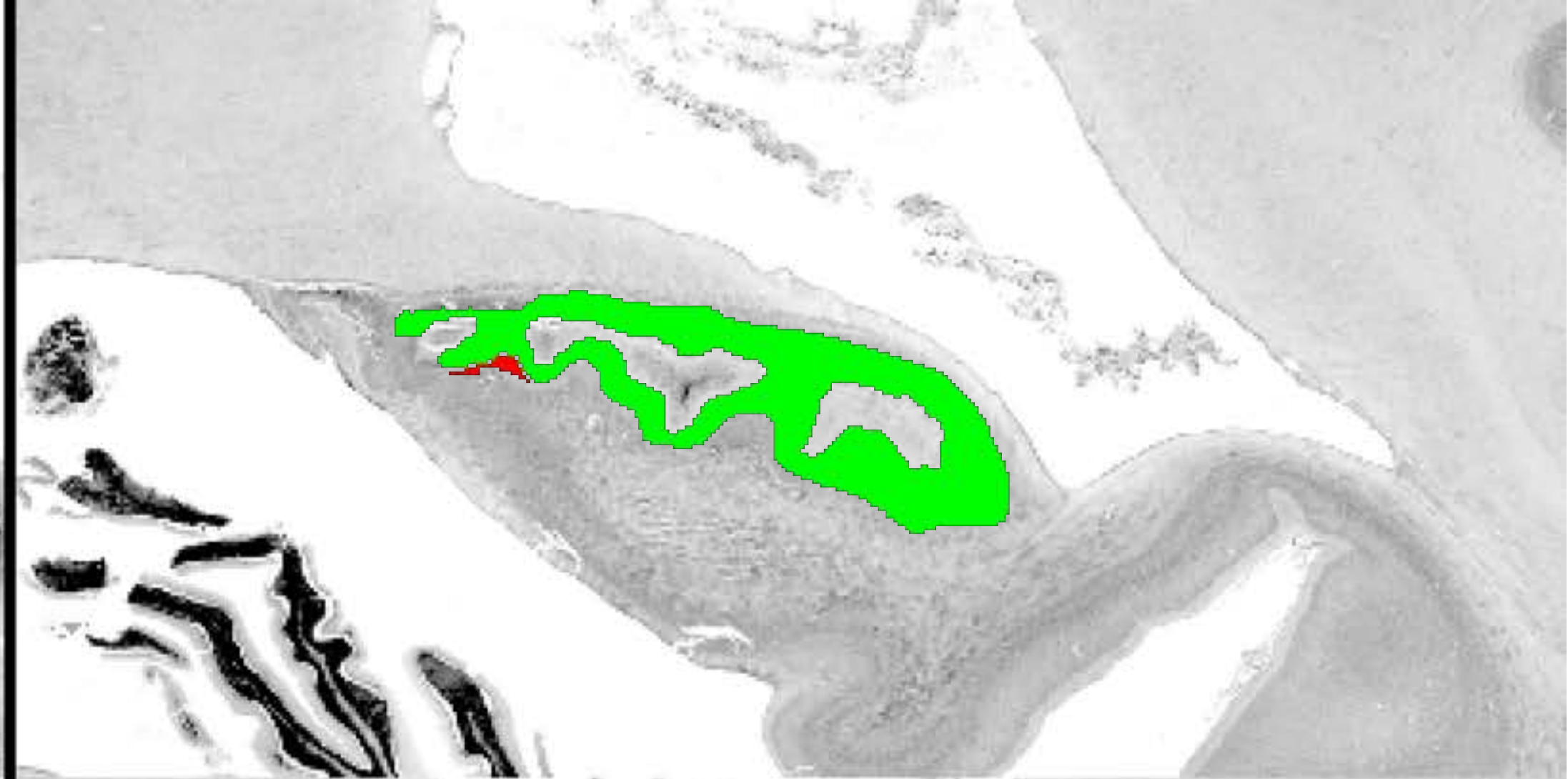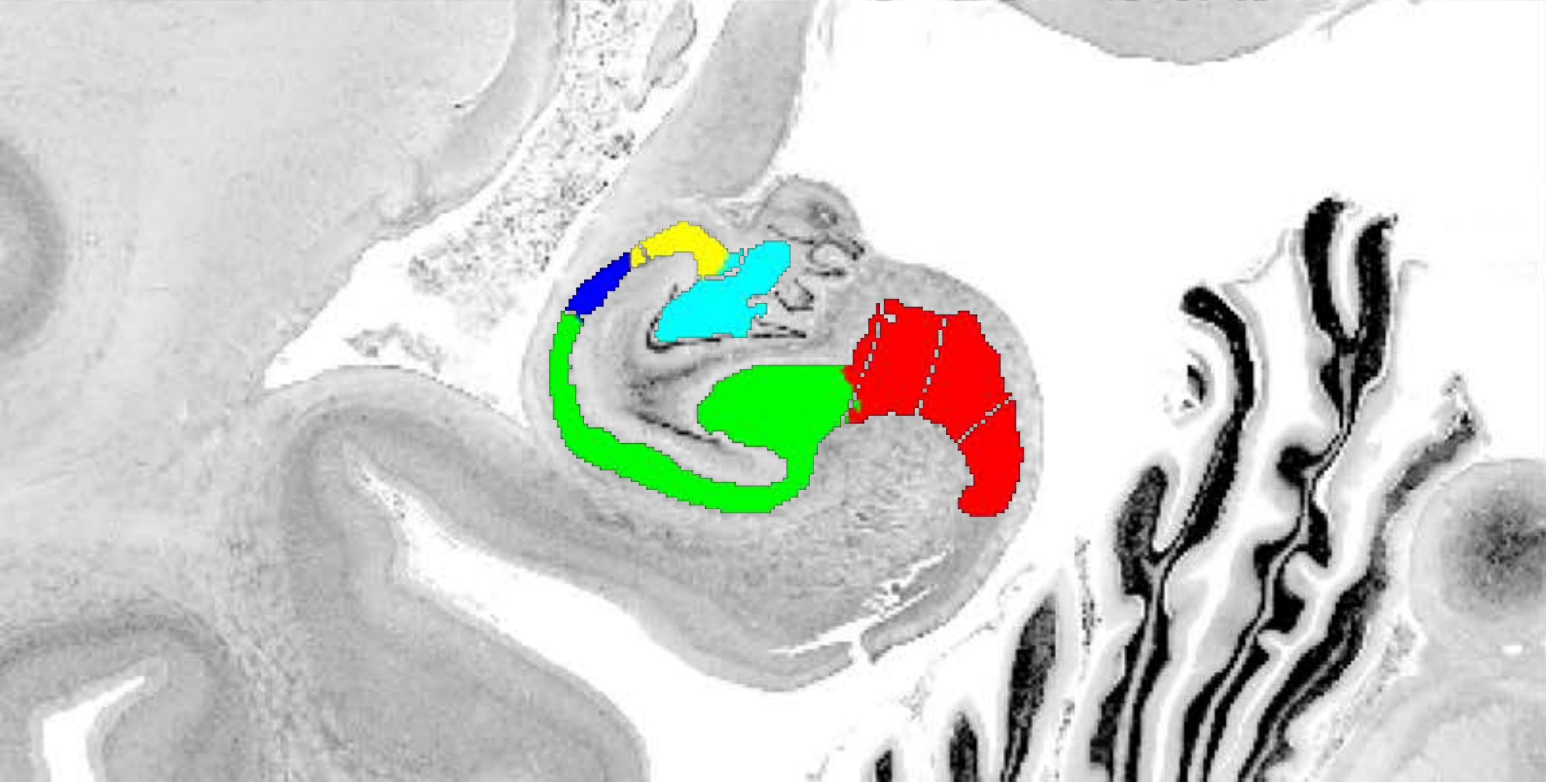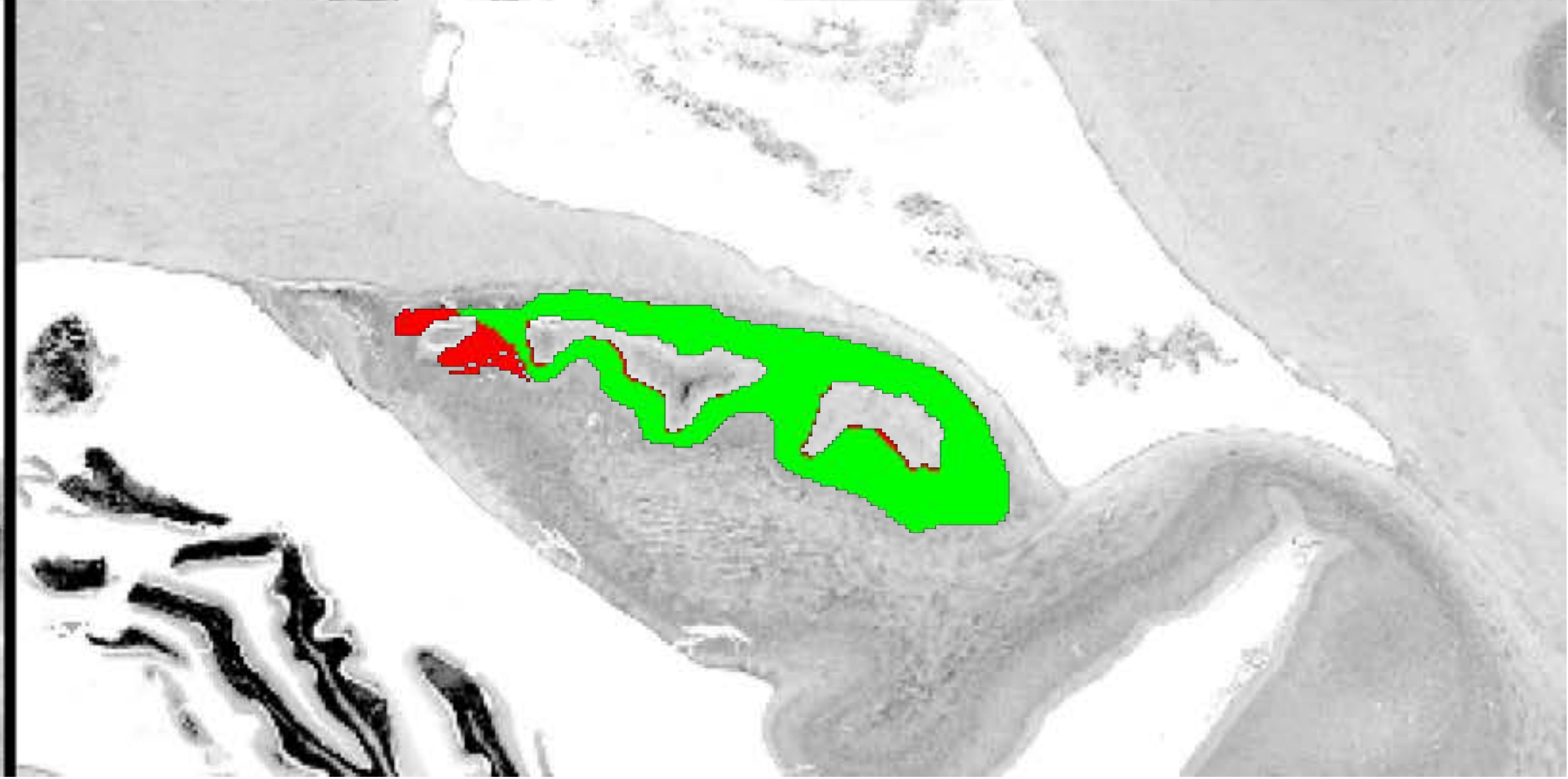

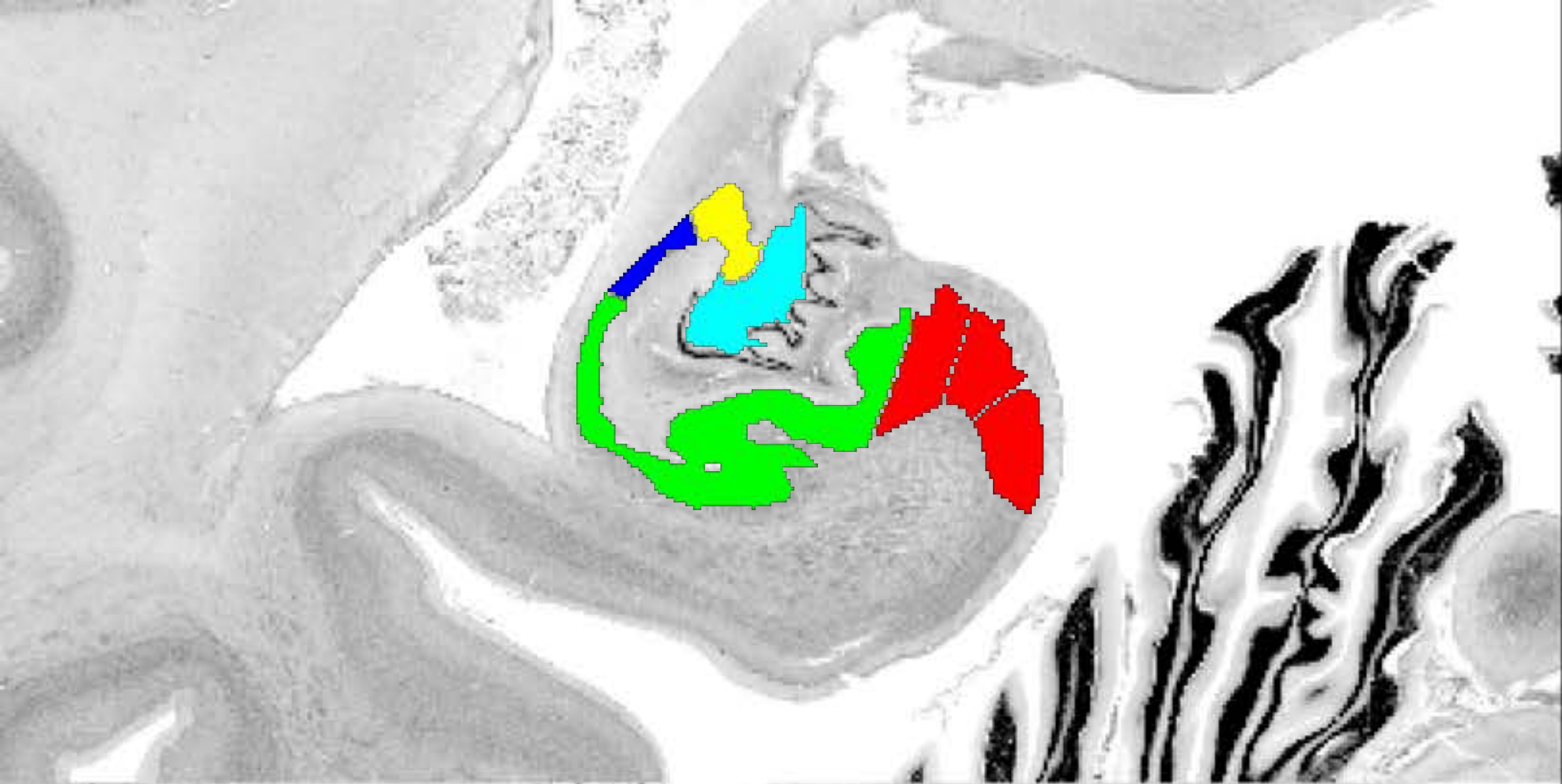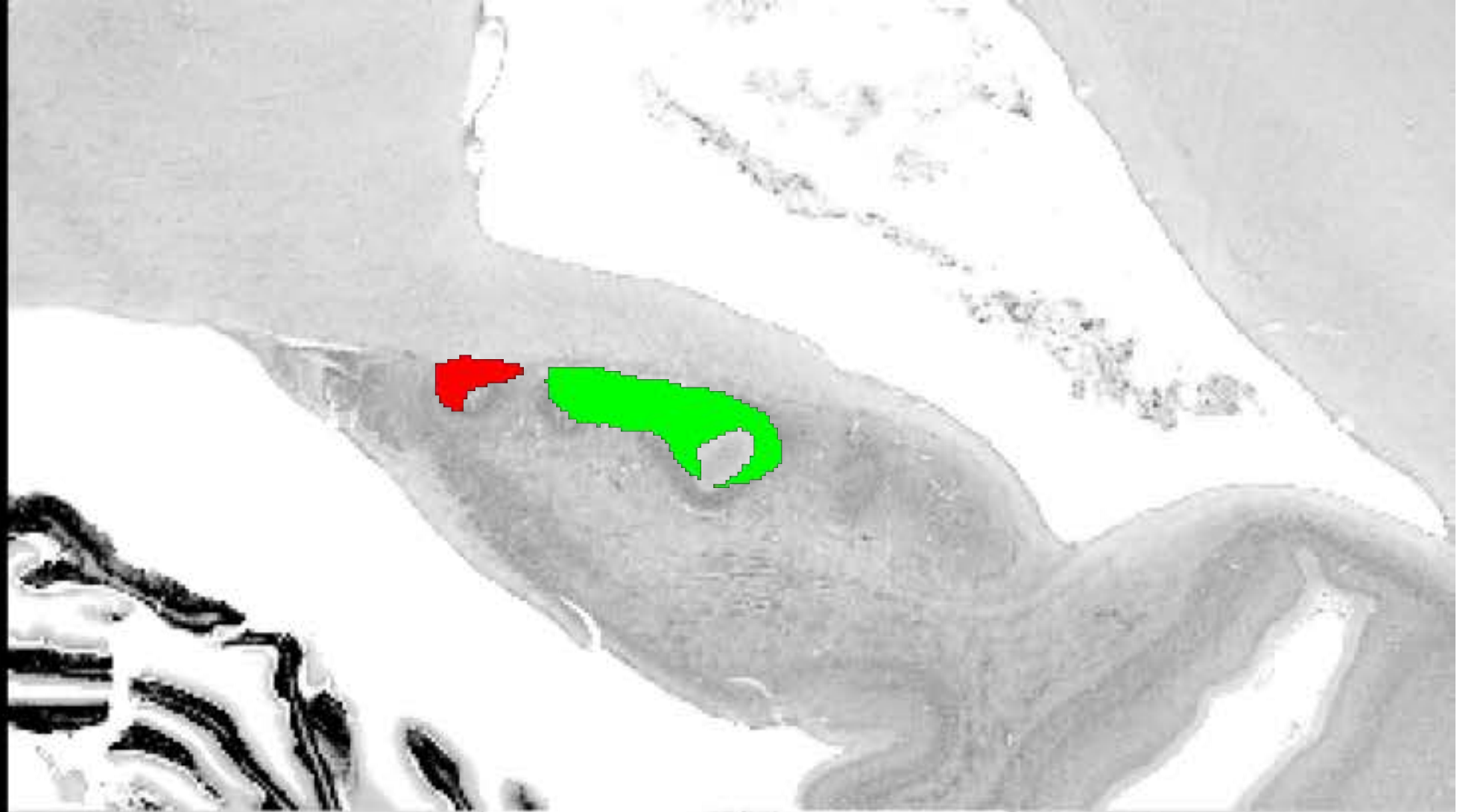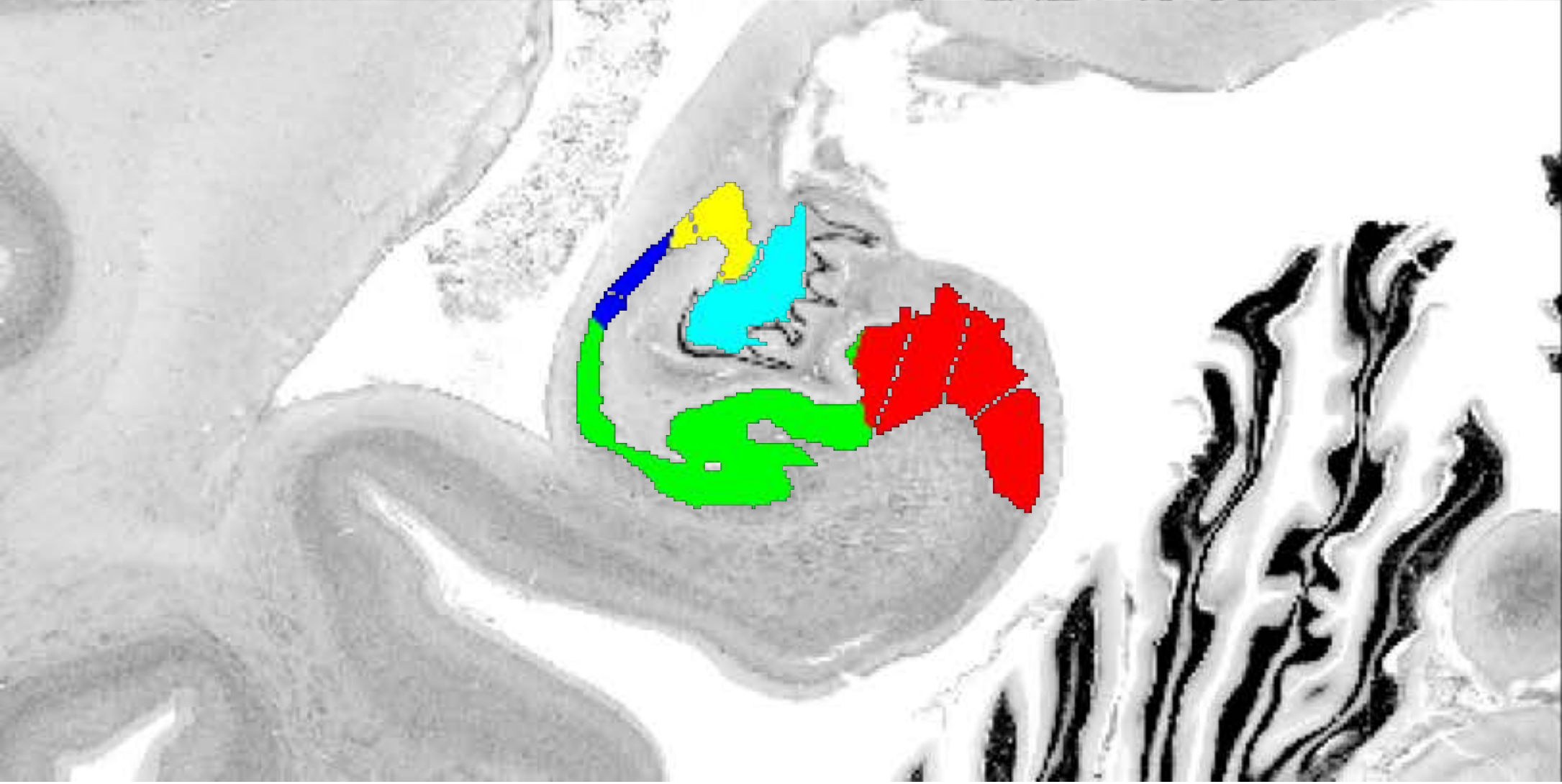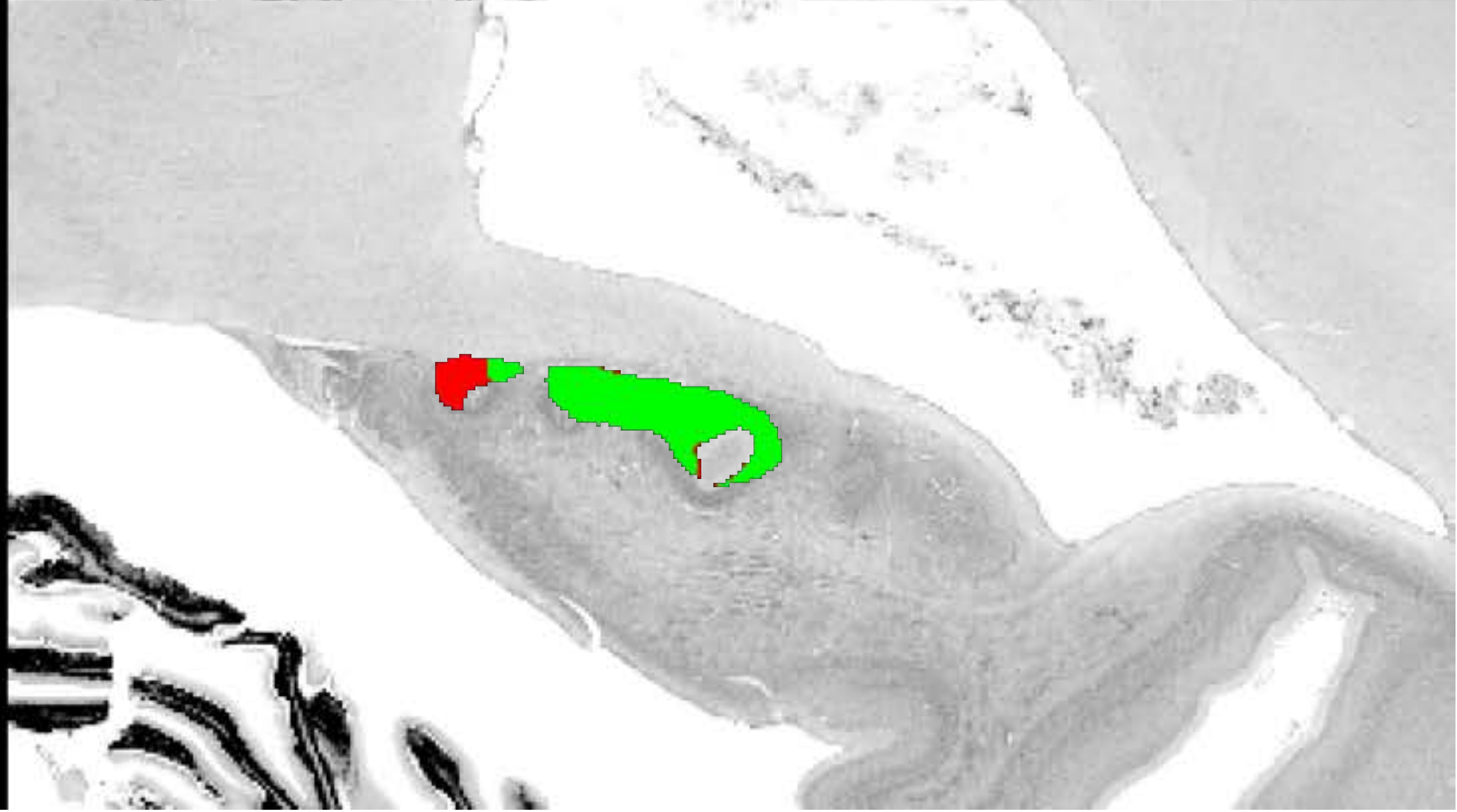

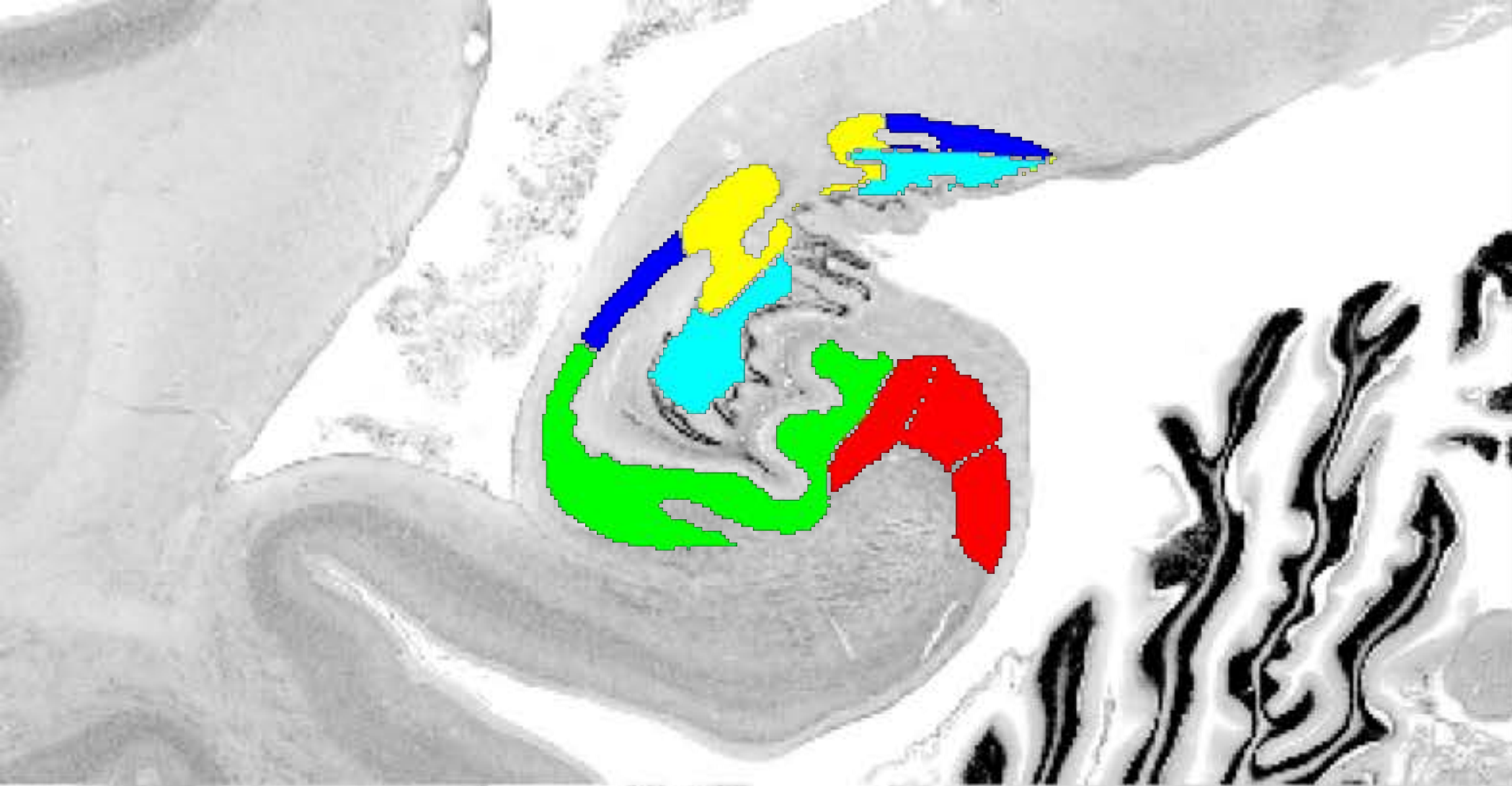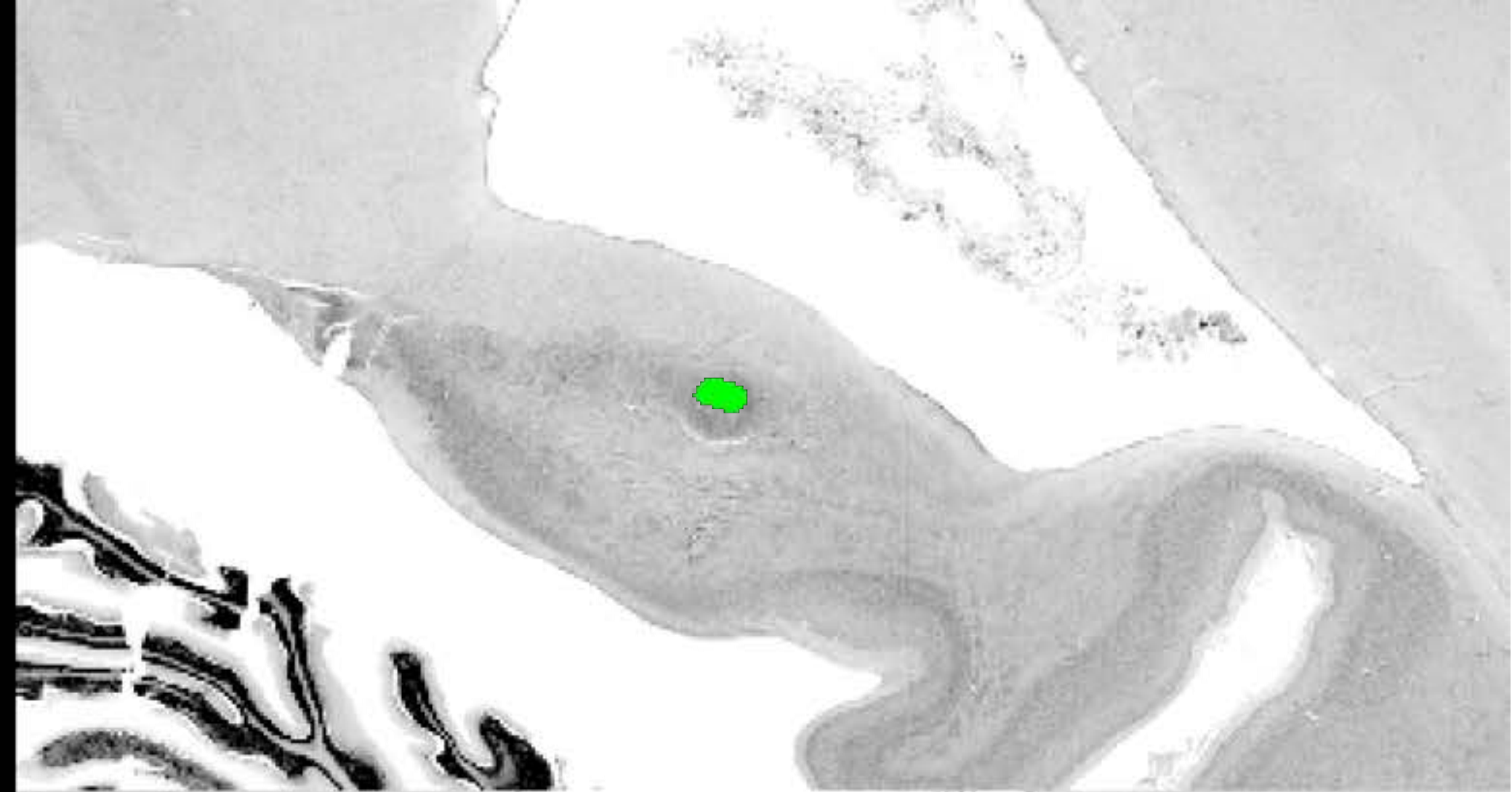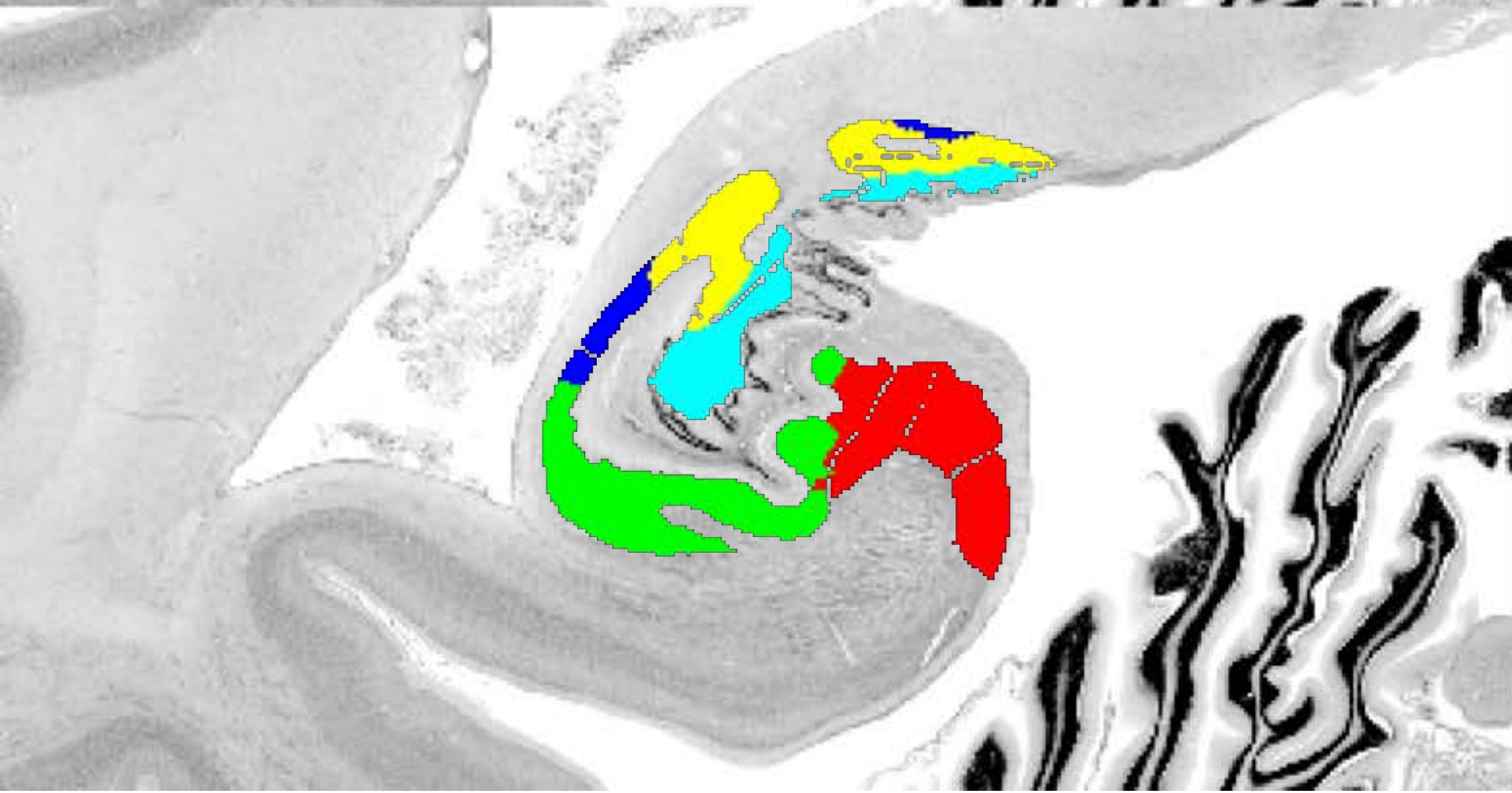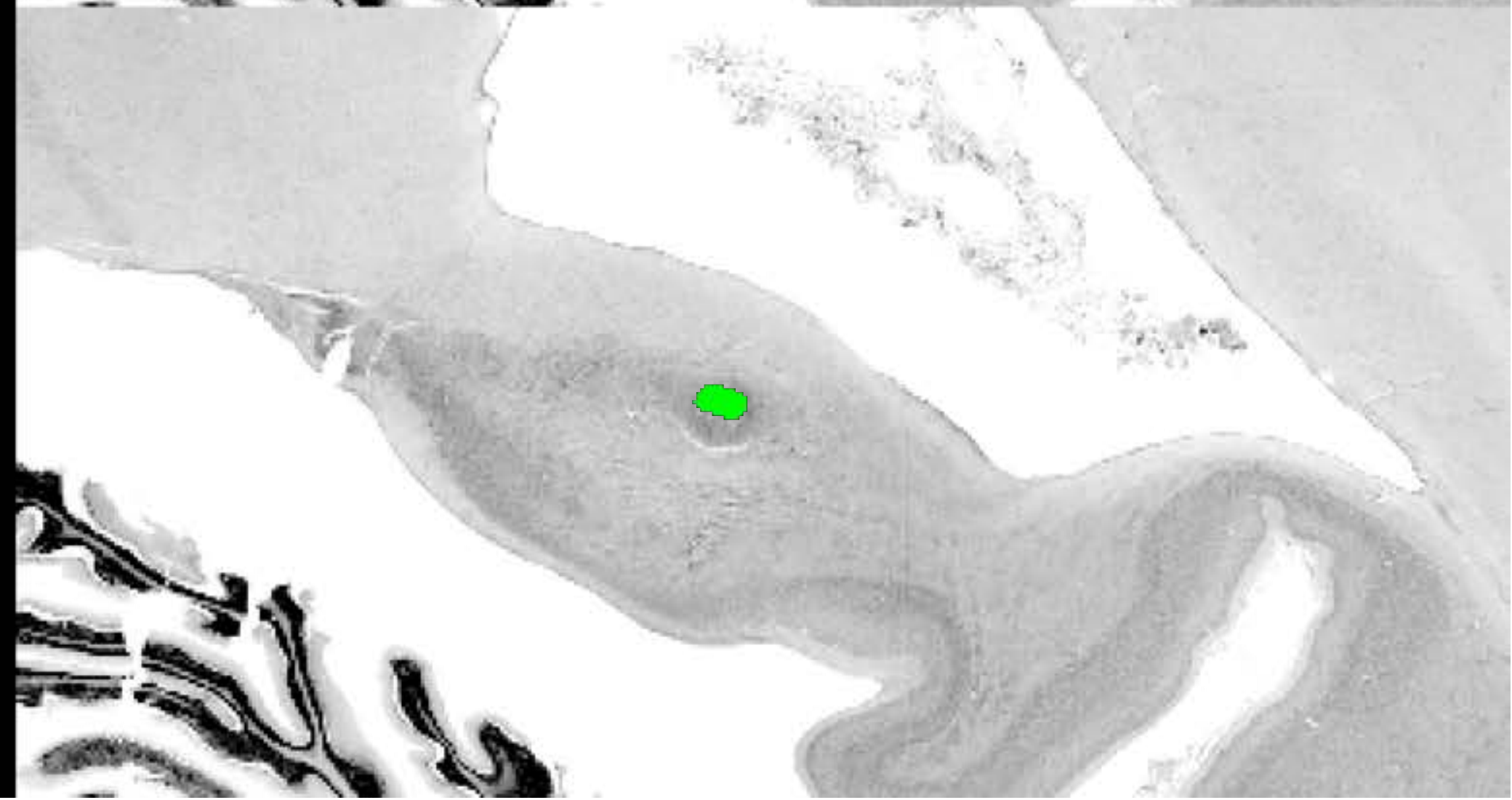

Supplement: Supplementary file 1. — Slices are ordered from anterior-to-posterior. [file elife-88404-supp1.pdf]
